# Communities That HEAL Intervention and Mortality Including Polysubstance Overdose Deaths: A Randomized Clinical Trial

**DOI:** 10.1001/jamanetworkopen.2024.40006

**Published:** 2024-10-21

**Authors:** Bridget Freisthler, Rouba A. Chahine, Jennifer Villani, Redonna Chandler, Daniel J. Feaster, Svetla Slavova, Jolene Defiore-Hyrmer, Alexander Y. Walley, Sarah Kosakowski, Arnie Aldridge, Carolina Barbosa, Sabana Bhatta, Candace Brancato, Carly Bridden, Mia-Cara Christopher, Tom Clarke, James David, Lauren D’Costa, Irene Ewing, Soledad Fernandez, Erin Gibson, Louisa Gilbert, Megan E. Hall, Sarah Hargrove, Timothy Hunt, Elizabeth N. Kinnard, Lauren Larochelle, Aaron Macoubray, Shawn Nigam, Edward V. Nunes, Carrie B. Oser, Sharon Pagnano, Peter Rock, Pamela Salsberry, Aimee Shadwick, Thomas J. Stopka, Sylvia Tan, Jessica L. Taylor, Philip M. Westgate, Elwin Wu, Gary A. Zarkin, Sharon L. Walsh, Nabila El-Bassel, T. John Winhusen, Jeffrey H. Samet, Emmanuel A. Oga

**Affiliations:** 1 Ohio State University College of Social Work, Columbus; 2 RTI International, Research Triangle Park, North Carolina; 3 National Institute on Drug Abuse, National Institutes of Health, Bethesda, Maryland; 4 University of Miami Miller School of Medicine, Miami, Florida; 5 University of Kentucky, Lexington; 6 State of Ohio Board of Pharmacy, Columbus; 7 Boston Medical Center, Boston, Massachusetts; 8 New York State Department of Health, Albany; 9 Substance Abuse and Mental Health Services Administration, Rockville, Maryland; 10 Columbia University, New York, New York; 11 Columbia University Irving Medical Center, New York, New York; 12 University of Cincinnati College of Medicine, Cincinnati, Ohio; 13 Ohio State University Department of Biomedical Informatics and Center for Biostatistics, Columbus; 14 Massachusetts Department of Public Health, Dorchester; 15 Ohio State University College of Public Health, Columbus; 16 Recovery Ohio, Columbus; 17 Department of Public Health and Community Medicine, Tufts University School of Medicine, Boston, Massachusetts; 18 University of Kentucky College of Medicine, Center on Drug and Alcohol Research, Lexington

## Abstract

**Importance:**

The HEALing Communities Study (HCS) evaluated the effectiveness of the Communities That HEAL (CTH) intervention in preventing fatal overdoses amidst the US opioid epidemic.

**Objective:**

To evaluate the impact of the CTH intervention on total drug overdose deaths and overdose deaths involving combinations of opioids with psychostimulants or benzodiazepines.

**Design, Setting, and Participants:**

This randomized clinical trial was a parallel-arm, multisite, community-randomized, open, and waitlisted controlled comparison trial of communities in 4 US states between 2020 and 2023. Eligible communities were those reporting high opioid overdose fatality rates in Kentucky, Massachusetts, New York, and Ohio. Covariate constrained randomization stratified by state allocated communities to the intervention or control group. Trial groups were balanced by urban or rural classification, 2016-2017 fatal opioid overdose rate, and community population. Data analysis was completed by December 2023.

**Intervention:**

Increased overdose education and naloxone distribution, treatment with medications for opioid use disorder, safer opioid prescribing practices, and communication campaigns to mitigate stigma and drive demand for evidence-based interventions.

**Main Outcomes and Measures:**

The primary outcome was the number of drug overdose deaths among adults (aged 18 years or older), with secondary outcomes of overdose deaths involving specific opioid-involved drug combinations from death certificates. Rates of overdose deaths per 100 000 adult community residents in intervention and control communities from July 2021 to June 2022 were compared with analyses performed in 2023.

**Results:**

In 67 participating communities (34 in the intervention group, 33 in the control group) and including 8 211 506 participants (4 251 903 female [51.8%]; 1 273 394 Black [15.5%], 603 983 Hispanic [7.4%], 5 979 602 White [72.8%], 354 527 other [4.3%]), the average rate of overdose deaths involving all substances was 57.6 per 100 000 population in the intervention group and 61.2 per 100 000 population in the control group. This was not a statistically significant difference (adjusted rate ratio [aRR], 0.92; 95% CI, 0.78-1.07; *P* = .26). There was a statistically significant 37% reduction (aRR, 0.63; 95% CI, 0.44-0.91; *P* = .02) in death rates involving an opioid and psychostimulants (other than cocaine), and nonsignificant reductions in overdose deaths for an opioid with cocaine (6%) and an opioid with benzodiazepine (1%).

**Conclusion and Relevance:**

In this clinical trial of the CTH intervention, death rates involving an opioid and noncocaine psychostimulant were reduced; total deaths did not differ statistically. Community-focused data-driven interventions that scale up evidence-based practices with communications campaigns may effectively reduce some opioid-involved polysubstance overdose deaths.

**Trial Registration:**

ClinicalTrials.gov Identifier: NCT04111939

## Key Points

**Question:**

Did a data-driven, community coalition–engaged intervention effectively reduce opioid overdose deaths, total drug overdose deaths, and specific opioid-involved polysubstance overdose deaths?

**Findings:**

In this parallel-group cluster-randomized clinical trial of 67 communities, intervention communities had a nonsignificant 8% lower overdose death rate than control communities and a statistically significant 37% fewer deaths due to an opioid combined with a psychostimulant other than cocaine.

**Meaning:**

These results suggest that community-focused, data-driven interventions that scale up evidence-based practices with a communications campaign may collectively contribute to successes in addressing the evolving nature of some opioid-involved polysubstance overdose deaths.

## Teaser

This randomized clinical trial examines the effectiveness of a coordinated intervention to lower drug overdose deaths at the community level with evidence-based practices, with a specific focus on polysubstance overdoses.

## Introduction

The incidence of drug overdose deaths in the US has steadily increased since 2001.^1^ In 2022, more than 100 000 individuals died from a drug overdose,^2^ of which almost half involved more than 1 substance.^3^ Fentanyl, a synthetic opioid, accounts for over three-quarters of drug overdoses in the US^2^ and is being combined with nonopioid substances (eg, psychostimulants) more frequently.^2^ The ubiquitous presence of fentanyl complicates overdose prevention because: (1) fentanyl’s potency far exceeds that of other opioids (eg, diverted prescription opioids or heroin); (2) fentanyl’s concentration and distribution within the unregulated, illicit drug supply is erratic, so the dose of fentanyl with each use is unpredictable and potentially fatal^4,5^; and (3) the infiltration of fentanyl into the nonopioid drug supply places people lacking opioid tolerance at elevated risk. Fentanyl in the psychostimulant drug supply is one reason for current overdose death rates disproportionately impacting Black and indigenous individuals.^6,7^

Tailored overdose education and naloxone distribution (OEND),^8,9,10,11^ FDA-approved medications for opioid use disorder (MOUD),^12^ and safer opioid prescribing practices by physicians^13^ are recommended evidence-based practices (EBPs) for reducing opioid overdose deaths. The HEALing (Helping to End Addiction Long-term) Communities Study (HCS) was designed to achieve significant reductions in opioid overdose deaths using a community-engaged, data-driven intervention that paired implementing evidence-based practices (EBPs) with communication campaigns in communities highly impacted by opioid overdose deaths across 4 states.^14,15,16,17,18,19,20^ Communities that received the Communities That HEAL (CTH) intervention^19^ had 9% fewer opioid-involved overdose deaths compared with waitlisted control communities.^21^ In contrast to the primary outcome analysis,^19^ we assessed whether the CTH intervention reduced all drug overdose deaths, including those not involving opioids. As secondary a priori objectives, we examined whether the CTH intervention reduced the rate of opioid overdose deaths that co-occurred with psychostimulants other than cocaine (likely methamphetamine), cocaine, or benzodiazepines. Furthermore, we separately described rates of substance-specific drug overdoses, including heroin, any psychostimulants, psychostimulants other than cocaine, cocaine, and benzodiazepines.

## Methods

### Trial Design

HCS was a multisite, parallel group, cluster randomized, unmasked, waitlist-controlled trial in 4 states (Kentucky, Massachusetts, New York, and Ohio).^19^ States implemented the CTH intervention in intervention communities, while the waiting-list comparison communities continued care as usual. The trial was registered on ClinicalTrials.gov (NCT04111939). Advarra, Inc provided institutional review board approval for HCS; the study received a Waiver of Consent and Full Waiver of HIPAA authorization from Advarra for using secondary administrative data. The National Institute on Drug Abuse (NIDA) chartered a data and safety monitoring board comprised of an independent group of individuals to monitor the safety of the trial. We followed the Consolidated Standards of Reporting Trials (CONSORT) guidelines for reporting parallel group randomized trails. The trial protocol appears in Supplement 1.

### Study Population

Sixty-seven communities (counties or municipalities) highly impacted by the opioid crisis were selected from the 4 states based on previously described criteria.^19^ One community declined to participate in the study after randomization (but before intervention activities commenced), reducing the per protocol sample to 66 communities (Figure).

**Figure.**
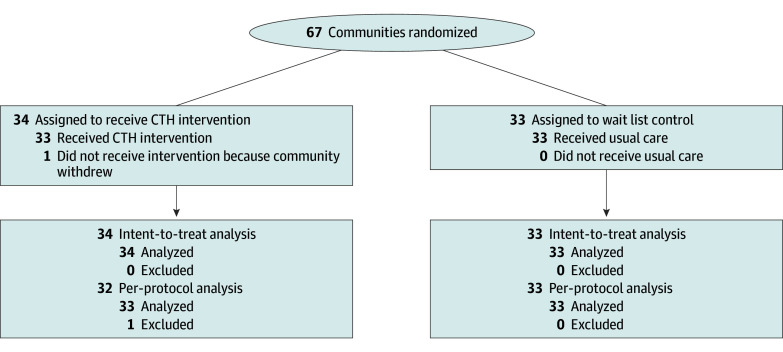
Study Flow Diagram for HEALing Communities in 4 US States

### Study Procedures and Intervention

#### Randomization

Randomization to CTH intervention or wait-list control communities was stratified by state. For each state, covariate constrained randomization was used to ensure balance between the waves on urban or rural classification, community population, and 2016-2017 opioid overdose death rate.^19^

#### Intervention

The intervention included a community-engaged approach with local coalitions to employ data-driven decision-making using community-level data to select and guide the implementation of EBPs, coupled with communication campaigns. The EBPs described in the Opioid-Overdose Reduction Continuum of Care Approach^15^ included OEND, MOUD, and safer opioid prescribing and disposal practices, which were implemented in health care, behavioral health, and criminal legal settings in each community. EBP selection and implementation were tailored to address the unique needs of the communities based on local data.^18^ Communications campaigns were designed to increase demand for EBPs and reduce stigma related to opioid use disorder (OUD) and its treatment.^16^ Activities occurred in intervention communities from January 2020 through June 2022. Comparison of outcomes across study used data from July 2021 through June 2022.

#### Outcomes

In this article, the a priori central outcome was the number of drug overdose deaths among adult community residents (18 years or older). Drug overdose (ie, poisoning) deaths were identified using death certificate records with an underlying cause indicating drug poisoning using the *International Statistical Classification of Diseases and Related Health Problems, Tenth Revision (ICD-10)* (codes X40-X44, X60-X64, X85, and Y10-Y14).^22^ Attribution to a HCS community was based on the decedent’s residence address.

Strata for effect modification of total overdose deaths included state, urban or rural status, age group (18 to 34 years, 35 to 54 years, and 55 years or older), sex (male and female), and race and ethnicity (Hispanic, non-Hispanic Black, non-Hispanic White, and non-Hispanic other [American Indian, Alaskan Native, Asian, Native Hawaiian, other Pacific Islander, other race not previously mentioned]) as provided by an informant. Race and ethnicity were included as study variables because they have been associated with drug overdose deaths in previous work.^6,7^ For communities that represent counties (48 of 67 communities), population estimates are from 2020 Bridged-Race Population Estimates retrieved via the National Vital Statistics System on June 20, 2023^23^; for communities that represent units smaller than counties (19 of 67 communities), population estimates are from 2017-2021 American Community Survey 5-Year Estimates retrieved via US Census data on June 20, 2023.^24^ Urban or rural status was determined by the CDC NCHS urban-rural classification scheme in Kentucky, New York, and Ohio.^25^ Massachusetts assigned rural status to communities that had a population density below 500 residents per square mile.^26^

Three a priori secondary outcomes assessed the number of opioid-involved overdose deaths (*ICD-10* codes T40.0-T40.4 or T40.6) in conjunction with: (1) a psychostimulant other than cocaine (T43.6); (2) cocaine (T40.5); or (3) benzodiazepine (T42.4). Strata included state and urban or rural status. Tertiary outcomes for drug overdose deaths included: (1) heroin (*ICD-10* code T40.1); (2) synthetic opioids except methadone (T40.4); (3) any opioid (T40.0–T40.4 or T40.6) and any psychostimulant (including cocaine) (T40.5 and T43.6); (4) psychostimulant other than cocaine (T43.6); (5) cocaine (T40.5); (6) any psychostimulant (including cocaine) (T40.5 or T43.6); and (7) benzodiazepine (T42.4).

### Statistical Analysis

Descriptive statistics (frequencies with percentages or means with standard deviations) for each outcome were used to summarize the number of events, population, and rates by wave and by subgroup and wave. Rates were calculated per 100 000 adult population.

For central and secondary outcomes, negative binomial regression models with robust, empirical, sandwich standard error estimates were used, and small-sample adjustments were performed per the prespecified statistical analysis plan (Supplement 1).^27^ Small sample adjusted empirical standard error estimates were applied using the average of 2 small-sample corrected empirical estimators^28,29^ as proposed.^30^ Each model was adjusted by trial group (intervention vs control), state (Kentucky, Massachusetts, New York, or Ohio), urban or rural classification, baseline (2019) opioid overdose death rate, and the baseline rate of the specific outcome. Adjusted rates and 95% CIs were calculated using least-squares means. Models were estimated using the intention-to-treat population and sensitivity analyses were performed using the per-protocol population.

Subgroup analyses were performed by including an interaction term between the trial group and a subgroup variable added as a fixed effect in the model and adding community as a random effect to account for clustering within communities to assess effect modifications. For total overdose deaths, effect modifiers included state, urban or rural classification, sex, and race and ethnicity. For the secondary outcomes, state and urban or rural classification were considered due to the constraints in data availability. To control the false discovery rate, the Benjamini-Hochberg procedure^31^ was applied across the complete set of interaction tests with 95% Bonferroni-corrected CIs.

Post hoc analyses were performed to help in the interpretation of the results. To assess the impact of the community size on the analysis of the primary and secondary outcomes, these analyses were conducted using modified Poisson regression. An additional post hoc analysis was performed to describe the change in drug death rate from 2019 through the comparison period using the 2019 and comparison period death counts in a repeated measure generalized estimating equations–type marginal negative binomial model with an unstructured working correlation matrix. This model was adjusted for time period, wave, time period–wave interaction, state, and urban and rural status. For descriptive purposes, a post hoc estimate applied the observed overdose death rate for comparison communities to the intervention communities population to calculate the number of intervention overdose deaths averted. Results were considered statistically significant for 2-sided *P* values <.05. All analyses were conducted by December 2023 using SAS version 9.4 (SAS Institute).

## Results

The total sample included 67 randomized communities and 8 211 506 participants (4 251 903 female [51.8%]; 1 273 394 Black [15.5%], 603 983 Hispanic [7.4%], 5 979 602 White [72.8%], 354 527 other [4.3%]) (Table 1). The intervention consisted of 34 communities, including 8 communities each in Kentucky, Massachusetts, and New York, and 10 in Ohio, with 19 urban communities and a total population of 4 439 170. Controls consisted of 33 communities, 8 communities each in Kentucky, Massachusetts, and New York, and 9 in Ohio, with 19 urban communities and a total population of 3 772 336. In 2019 (baseline), the mean (SD) rates of drug overdose deaths were 44.8 (24.1) vs 44.9 (20.9) per 100 000 adults; 6.4 (10.0) vs 4.4 (5.8) per 100 000 adults for deaths involving opioids and any noncocaine psychostimulant; 7.4 (7.1) vs 10.1 (11.0) per 100 000 adults for opioids combined with cocaine deaths; and 6.7 (5.5) vs 7.7 (5.4) per 100 000 adults for opioid and benzodiazepine deaths, for intervention vs control communities, respectively. Outcomes during the baseline period were similar to the US population affected by overdose deaths (eTable 1 in Supplement 2). Over the course of the trial, intervention communities selected 806 EBP strategies and 615 were implemented^21^; community stigma toward those with OUD was also reduced, as reported in a previous study.^42^

**Table 1.** Baseline Demographic Characteristics of Communities Participating in the HEALing Communities Study by Trial Group

Characteristic	Trial group, No. (%)^a^		Overall, No. (%)^a^
	Intervention communities	Control communities	
No. of randomized communities	34	33	67
Research site			
Kentucky	8 (23.5)	8 (24.2)	16 (23.9)
Massachusetts	8 (23.5)	8 (24.2)	16 (23.9)
New York	8 (23.5)	8 (24.2)	16 (23.9)
Ohio	10 (29.4)	9 (27.3)	19 (28.4)
Urban or rural classification			
Urban	19 (55.9)	19 (57.6)	38 (56.7)
Rural	15 (44.1)	14 (42.4)	29 (43.3)
Population aged ≥18 y^b^			
Total, No.	4 439 170	3 772 336	8 211 506
Mean (SD)	130 563.8 (200 088.0)	114 313.2 (201 417.3)	122 559.8 (199 385.0)
Age^b^			
18-34 y	1 334 880 (30.1)	1 178 210 (31.2)	2 513 090 (30.6)
35-54 y	1 353 341 (30.5)	1 180 392 (31.3)	2 533 733 (30.9)
≥55 y	1 750 949 (39.4)	1 413 734 (37.5)	3 164 683 (38.5)
Sex^b^			
Male	2 133 827 (48.1)	1 825 776 (48.4)	3 959 603 (48.2)
Female	2 305 343 (51.9)	1 946 560 (51.6)	4 251 903 (51.8)
Race and ethnicity^b^			
Hispanic	281 329 (6.3)	322 654 (8.6)	603 983 (7.4)
Non-Hispanic Black	728 037 (16.4)	545 357 (14.5)	1 273 394 (15.5)
Non-Hispanic White	3 229 233 (72.7)	2 750 369 (72.9)	5 979 602 (72.8)
Non-Hispanic other^d^	200 571 (4.5)	153 956 (4.1)	354 527 (4.3)
Rate of opioid overdose deaths^c^			
Mean (SD), No./100 000 adults	38.2 (22.8)	37.1 (20.3)	37.7 (21.4)
No. of communities with missing data due to suppression	0	0	0
Rate of drug overdose deaths^c^			
Mean (SD), No./100 000 adults	44.8 (24.1)	44.9 (20.9)	44.9 (22.4)
No. of communities with missing data due to suppression	0	0	0
Rate of overdose deaths involving any opioid and any psychostimulant (other than cocaine)^c^			
Mean (SD), No./100 000 adults	6.4 (10.0)	4.4 (5.8)	5.4 (8.2)
No. of communities with missing data due to suppression	0	0	0
Rate of overdose deaths involving any opioid and cocaine^c^			
Mean (SD), No./100 000 adults	7.4 (7.1)	10.1 (11.0)	8.7 (9.2)
No. of communities with missing data due to suppression	0	0	0
Rate of overdose deaths involving any opioid and any benzodiazepine^c^			
Mean (SD), No./100 000 adults	6.7 (5.5)	7.7 (5.4)	7.2 (5.4)
No. of communities with missing data due to suppression	0	0	0

^a^ Percentages may not add up to 100 due to rounding.

^b^ For communities that represent counties (48 of 67), population estimates are from 2020 Bridged-Race Population Estimates retrieved via the National Vital Statistics System on June 20, 2023.^23^ For communities that represent units smaller than counties (19 of 67), population estimates are from 2017-2021 American Community Survey 5-Year Estimates retrieved via US Census data on June 20, 2023.^24^

^c^ Rate per 100 000 individuals ages 18 years or older calculated as the observed number of events as measured from January 2019 to December 2019 divided by the observed community population size of individuals 18 years of age or older measured from the 2020 Bridged-Race Population Estimates or the 2017-2021 American Community Survey 5-Year Estimates multiplied by 100 000.

^d^ Other includes American Indian, Alaskan Native, Asian, Native Hawaiian, other Pacific Islander, and other race not previously mentioned.

During the comparison period, the rate of the total sum of drug overdose deaths per population was 58.2 per 100 000 adults in intervention communities and 70.0 per 100 000 adults in control communities with a raw rate ratio of 0.83 (Table 2). Across state, age, sex, and race and ethnicity strata, the raw rate ratios ranged from 0.67 to 0.92. For urban or rural strata, the raw rate ratio for rural communities was 1.01, indicating drug overdose rates in intervention and control communities were similar. For opioid overdose deaths involving psychostimulants other than cocaine, the raw rate ratio was 0.63, 0.81 for an opioid and cocaine overdose deaths, and 0.91 for opioid overdose deaths involving benzodiazepines. Mean event, population, and community-specific rates per 100 000 adult residents among intervention and control communities are included in eTable 2 in Supplement 2.

**Table 2.** Primary and Secondary Outcomes During the Evaluation Period Using the Intention-to-Treat Population

Outcome by group	Intervention communities			Control communities			Overall rate ratio^a^
	Total events, No. (%)	Total population, No. (%)	Overall raw rate, No./100 000 adults^b^	Total events, No. (%)	Total population, No. (%)	Overall raw rate, No./100 000 adults^b^	
**Drug overdose deaths**							
Overall, No.	2582	4 439 170	58.2	2640	3 772 336	70.0	0.83
Research site							
Kentucky	450 (17.4)	617 841 (13.9)	72.8	673 (25.5)	815 764 (21.6)	82.5	0.88
Massachusetts	224 (8.7)	359 314 (8.1)	62.3	265 (10.0)	356 545 (9.5)	74.3	0.84
New York	543 (21.0)	1 101 497 (24.8)	49.3	613 (23.2)	976 069 (25.9)	62.8	0.78
Ohio	1365 (52.9)	2 360 518 (53.2)	57.8	1089 (41.3)	1 623 958 (43.0)	67.1	0.86
Urban or rural classification							
Urban	2202 (85.3)	3 793 353 (85.5)	58.0	2331 (88.3)	3 242 663 (86.0)	71.9	0.81
Rural	380 (14.7)	645 817 (14.5)	58.8	309 (11.7)	529 673 (14.0)	58.3	1.01
Age							
18-34 y	655 (25.4)	1 334 880 (30.1)	49.1	704 (26.7)	1 178 210 (31.2)	59.8	0.82
35-54 y	1260 (48.8)	1 353 341 (30.5)	93.1	1272 (48.2)	1 180 392 (31.3)	107.8	0.86
≥55 y	667 (25.8)	1 750 949 (39.4)	38.1	664 (25.2)	1 413 734 (37.5)	47.0	0.81
Sex							
Male	1772 (68.6)	2 133 827 (48.1)	83.0	1818 (68.9)	1 825 776 (48.4)	99.6	0.83
Female	810 (31.4)	2 305 343 (51.9)	35.1	822 (31.1)	1 946 560 (51.6)	42.2	0.83
Missing	0	NA	NA	0	NA	NA	NA
Race and ethnicity							
Hispanic	154 (6.0)	281 329 (6.3)	54.7	193 (7.3)	322 654 (8.6)	59.8	0.92
Non-Hispanic Black	556 (21.5)	728 037 (16.4)	76.4	620 (23.5)	545 357 (14.5)	113.7	0.67
Non-Hispanic White	1826 (70.7)	3 229 233 (72.7)	56.5	1774 (67.2)	2 750 369 (72.9)	64.5	0.88
Non-Hispanic other^c^	39 (1.5)	200 571 (4.5)	19.4	43 (1.6)	153 956 (4.1)	27.9	0.70
Missing	7 (0.3)	NA	NA	10 (0.4)	NA	NA	NA
**Overdose deaths involving any opioid and any psychostimulant (other than cocaine)**							
Overall	396	4 439 170	8.9	533	3 772 336	14.1	0.63
Research site							
Kentucky	130 (32.8)	617 841 (13.9)	21.0	265 (49.7)	815 764 (21.6)	32.5	0.65
Massachusetts	9 (2.3)	359 314 (8.1)	2.5	9 (1.7)	356 545 (9.5)	2.5	0.99
New York	41 (10.4)	1 101 497 (24.8)	3.7	81 (15.2)	976 069 (25.9)	8.3	0.45
Ohio	216 (54.5)	2 360 518 (53.2)	9.2	178 (33.4)	1 623 958 (43.0)	11.0	0.83
Urban or rural classification							
Urban	287 (72.5)	3 793 353 (85.5)	7.6	452 (84.8)	3 242 663 (86.0)	13.9	0.54
Rural	109 (27.5)	645 817 (14.5)	16.9	81 (15.2)	529 673 (14.0)	15.3	1.10
**Overdose deaths involving any opioid and cocaine**							
Overall	746	4 439 170	16.8	785	3 772 336	20.8	0.81
Research site							
Kentucky	57 (7.6)	617 841 (13.9)	9.2	96 (12.2)	815 764 (21.6)	11.8	0.78
Massachusetts	93 (12.5)	359 314 (8.1)	25.9	108 (13.8)	356 545 (9.5)	30.3	0.85
New York	212 (28.4)	1 101 497 (24.8)	19.2	236 (30.1)	976 069 (25.9)	24.2	0.80
Ohio	384 (51.5)	2 360 518 (53.2)	16.3	345 (43.9)	1 623 958 (43.0)	21.2	0.77
Urban or rural classification							
Urban	708 (94.9)	3 793 353 (85.5)	18.7	724 (92.2)	3 242 663 (86.0)	22.3	0.84
Rural	38 (5.1)	645 817 (14.5)	5.9	61 (7.8)	529 673 (14.0)	11.5	0.51
**Overdose deaths involving any opioid and any benzodiazepine**							
Overall	291	4 439 170	6.6	272	3 772 336	7.2	0.91
Research site							
Kentucky	45 (15.5)	617 841 (13.9)	7.3	90 (33.1)	815 764 (21.6)	11.0	0.66
Massachusetts	44 (15.1)	359 314 (8.1)	12.2	24 (8.8)	356 545 (9.5)	6.7	1.82
New York	86 (29.6)	1 101 497 (24.8)	7.8	71 (26.1)	976 069 (25.9)	7.3	1.07
Ohio	116 (39.9)	2 360 518 (53.2)	4.9	87 (32.0)	1 623 958 (43.0)	5.4	0.92
Urban or rural classification							
Urban	260 (89.3)	3 793 353 (85.5)	6.9	231 (84.9)	3 242 663 (86.0)	7.1	0.96
Rural	31 (10.7)	645 817 (14.5)	4.8	41 (15.1)	529 673 (14.0)	7.7	0.62

Abbreviation: NA, not applicable.

^a^ Overall rate ratio calculated as the overall raw rate in intervention communities divided by the overall raw rate in control communities.

^b^ Raw rate calculated as 100 000 multiplied by the sum of all events in that group and trial group wave divided by the sum of the population in that group and trial group.

^c^ Other included American Indian, Alaskan Native, Asian, Native Hawaiian, other Pacific Islander, and other race not previously mentioned.

An 8% lower rate of overdose deaths in intervention compared with control communities was identified, although not statistically significant (adjusted rate ratio [aRR], 0.92; 95% CI, 0.78-1.07; *P* = .26) (Table 3). Effect modifications by state, urban or rural status, sex, age, or race and ethnicity were not significant. The aRR of overdose deaths involving any opioid and any psychostimulant (other than cocaine) demonstrated a statistically significant 37% reduction in intervention compared with control communities (aRR, 0.63; 95% CI, 0.44-0.91; *P* = .02), with no evidence of effect modification by state or urban or rural status. There was a nonsignificant 6% lower rate of overdose deaths involving an opioid and cocaine in intervention compared with control communities (aRR, 0.94; 95% CI, 0.69-1.30; *P* = .72) with no significant differences by state or urban or rural classification. There was no significant difference in the rate of overdose deaths involving any opioid and any benzodiazepine between intervention and control communities (aRR, 0.99; 95% CI, 0.73-1.35; *P* = .96), with no effect modification by state or urban or rural classification.

**Table 3.** Model-Based Adjusted Rates Within Trial Groups and Adjusted Relative Rates Between Intervention to Control Communities of Outcomes Using the Intention-to-Treat Population

Outcome by group	Adjusted rate (95% CI)^a^		Adjusted rate ratio (95% CI)^b^	*P* value^c^
	Intervention communities	Control communities		
**Drug overdose deaths**				
Overall	55.99 (50.00-62.71)	61.11 (54.25-68.83)	0.92 (0.78-1.07)	.26
Research site^d^				
Kentucky	69.98 (53.48-91.57)	64.75 (42.22-99.30)	1.08 (0.66-1.76)	NR
Massachusetts	55.20 (36.14-84.32)	57.32 (40.70-80.72)	0.96 (0.56-1.65)	
New York	54.05 (38.51-75.87)	61.99 (48.03-80.01)	0.87 (0.58-1.30)	
Ohio	48.01 (41.01-56.19)	58.73 (46.22-74.62)	0.82 (0.62-1.09)	
Urban or rural classification^d^				
Urban	57.05 (49.63-65.58)	60.82 (51.80-71.41)	0.94 (0.76-1.15)	NR
Rural	54.37 (43.50-67.94)	62.11 (49.06-78.63)	0.88 (0.63-1.21)	
Age^d^				
18-34 y	51.73 (44.80-59.72)	57.81 (48.55-68.85)	0.89 (0.72-1.11)	NR
35-54 y	78.54 (65.64-93.98)	87.03 (74.49-101.7)	0.90 (0.74-1.11)	
≥55 y	42.61 (35.72-50.83)	48.22 (36.28-64.10)	0.88 (0.64-1.22)	
Sex^d^				
Male	70.84 (59.40-84.49)	80.87 (70.51-92.75)	0.88 (0.73-1.05)	NR
Female	39.22 (33.88-45.40)	44.53 (37.04-53.53)	0.88 (0.71-1.09)	
Race and ethnicity^d^^,^^h^				
Hispanic	50.69 (33.37-76.99)	53.89 (40.43-71.85)	0.94 (0.57-1.55)	
Non-Hispanic Black	76.83 (60.34-97.83)	93.80 (74.50-118.1)	0.82 (0.59-1.14)	
Non-Hispanic White	52.59 (44.43-62.25)	57.01 (48.13-67.52)	0.92 (0.76-1.11)	NR
Non-Hispanic other	22.37 (11.09-45.12)	29.98 (13.93-64.52)	0.75 (0.27-2.04)	
**Overdose deaths involving any opioid and any psychostimulant (other than cocaine)**				
Overall	6.68 (5.14-8.69)	10.55 (7.75-14.36)	0.63 (0.44-0.91)	.02
Research site^d^				
Kentucky	18.27 (12.57-26.56)	24.61 (14.72-41.16)	0.74 (0.40-1.36)	NR
Massachusetts	2.94 (0.65-13.26)	3.45 (1.03-11.61)	0.85 (0.12-5.87)	
New York	4.26 (2.07-8.76)	10.39 (3.81-28.37)	0.41 (0.11-1.53)	
Ohio	8.86 (6.00-13.10)	12.60 (6.58-24.11)	0.70 (0.34-1.45)	
Urban/rural classification^d^				
Urban	5.83 (4.06-8.39)	8.75 (4.88-15.71)	0.67 (0.37-1.22)	NR
Rural	7.53 (4.97-11.42)	12.88 (8.45-19.61)	0.59 (0.33-1.04)	
**Overdose deaths involving any opioid and cocaine**				
Overall^e^^,^^f^	10.32 (8.32-12.80)	10.94 (8.28-14.47)	0.94 (0.69-1.30)	.72
Research site^d^				
Kentucky	7.51 (3.44-16.38)	6.77 (1.94-23.64)	1.11 (0.26-4.64)	NR
Massachusetts	12.15 (6.44-22.92)	12.52 (4.75-33.01)	0.97 (0.33-2.81)	
New York	15.68 (9.08-27.07)	16.27 (10.40-25.48)	0.96 (0.48-1.92)	
Ohio	8.14 (5.70-11.63)	9.97 (5.88-16.89)	0.82 (0.44-1.53)	
Urban or rural classification^d^				
Urban	13.81 (10.42-18.29)	13.13 (9.15-18.85)	1.05 (0.69-1.61)	
Rural	6.83 (4.44-10.51)	10.10 (6.08-16.80)	0.68 (0.35-1.29)	
**Overdose deaths involving any opioid and any benzodiazepine**				
Overall^e^^,^^g^	6.59 (5.25-8.27)	6.64 (5.00-8.82)	0.99 (0.73-1.35)	.96
Research site^d^				
Kentucky	7.73 (4.90-12.19)	8.30 (4.13-16.67)	0.93 (0.42-2.07)	NR
Massachusetts	7.28 (3.65-14.52)	4.95 (1.41-17.37)	1.47 (0.42-5.09)	
New York	7.31 (4.48-11.94)	7.24 (4.34-12.08)	1.01 (0.48-2.12)	
Ohio	4.95 (2.71-9.05)	6.18 (4.08-9.34)	0.80 (0.40-1.63)	
Urban or rural classification^d^				
Urban	7.07 (5.54-9.01)	6.44 (4.49-9.22)	1.10 (0.72-1.68)	NR
Rural	5.46 (3.26-9.14)	7.60 (4.48-12.89)	0.72 (0.35-1.46)	

Abbreviation: NR, not reported.

^a^ Adjusted rate per 100 000 residents aged 18 years and older.

^b^ Adjusted relative rate of outcome between intervention and control communities. For subgroups (research site, urban or rural classification, age, sex, race and ethnicity), the 95% CIs reported used a Bonferroni correction.

^c^ For subgroups (research site, urban or rural classification, age, sex, race and ethnicity), false discovery rate–adjusted *P* value for the interaction between stratification variable and trial group were not significant.

^d^ A separate negative binomial model was fit for each test of effect modification. Each model included the following fixed effects: trial group, research site (Kentucky, Massachusetts, New York, Ohio), urban or rural classification, baseline opioid overdose death rate, baseline rate of outcome, stratification variable (age, sex, race and ethnicity, if needed), and a 2-way interaction between trial group and the stratification variable.

^e^ Results obtained from a negative binomial model adjusting for research site (Kentucky, Massachusetts, New York , Ohio), urban or rural classification, baseline opioid overdose death rate, and baseline rate of the outcome. The natural log of the observed community population size of individuals 18 years of age or older measured from the 2020 Bridged-Race Population Estimates or the 2017-2021 American Community Survey 5-Year Averages is used as an offset.

^f^ Estimated dispersion parameter: k, 0.1472; 95% CI, 0.0774-0.2798.

^g^ Estimated dispersion parameter: k, 0.0925; 95% CI, 0.0298-0.2866.

^h^ Other included American Indian, Alaskan Native, Asian, Native Hawaiian, other Pacific Islander, and other race not previously mentioned.

The rates of death associated with each of the drug categories in intervention relative to control communities varied from 0.63 for deaths involving heroin (37% lower in intervention communities) to 0.89 (11% lower in intervention communities) for deaths involving benzodiazepines (Table 4). Stratifications by state and urban or rural status are presented in eTable 3 in Supplement 2.

**Table 4.** Descriptive Sums of Tertiary Outcomes During the Evaluation Period Using the Intention-to-Treat Population

Drug categories involved in overdose death	Intervention communities			Control communities			Overall rate ratio^a^
	Total events, No.	Total population, No.	Overall raw rate per 100 000 population	Total events, No.	Total population, No.	Overall raw rate per 100 000 population	
Heroin	93	4 439 170	2.1	125	3 772 336	3.3	0.63
Synthetic opioids except methadone	2075	4 439 170	46.7	2165	3 772 336	57.4	0.81
Any opioid and any psychostimulant (including cocaine)	1059	4 439 170	23.9	1218	3 772 336	32.3	0.74
Any psychostimulant (other than cocaine)	482	4 439 170	10.9	638	3 772 336	16.9	0.64
Cocaine	891	4 439 170	20.1	924	3 772 336	24.5	0.82
Any psychostimulant (including cocaine)	1277	4 439 170	28.8	1449	3 772 336	38.4	0.75
Benzodiazepine	317	4 439 170	7.1	304	3 772 336	8.1	0.89

^a^ Overall rate ratio calculated as the overall raw rate in Intervention Communities divided by the overall raw rate in Control Communities.

Unadjusted rates of total overdose deaths increased during COVID-19 in both waves during the comparison period; however, increases were greater in control communities (Table 1; eTable 1 in Supplement 2). Deaths involving heroin, either alone or in combination with other drugs, represented only 4.2% of opioid overdose fatalities over the study period. Over 80% of overdose deaths due to psychostimulants also involved an opioid. However, only about 50% of opioid-involved deaths also included a psychostimulant (eTable 4 in Supplement 2).

### Additional Analyses

Sensitivity analyses using the per protocol population (eTable 5 in Supplement 2) were similar to those using intention-to-treat. Exploratory post hoc sensitivity analysis assessing the impact of community size using Poisson distribution was significant for total drug overdose deaths, indicating a 15% reduction (aRR, 0.85; 95% CI, 0.72-1.00; *P* = .045) (eTable 6 in Supplement 2). The post hoc repeated measure marginal model using a negative binomial distribution was not significant (aRR, 0.93; 95% CI, 0.79-1.09; *P* = .37), but was significant when using a Poisson distribution (aRR, 0.85; 95% CI, 0.72-1.00; *P* = .046). The estimated number of drug overdose deaths avoided in intervention communities during the comparison period was 525.

## Discussion

Intervention communities had a nonsignificant 8% lower rate of drug overdose deaths after implementation of the CTH intervention, which represents an estimated 525 drug overdose deaths averted in intervention communities. Communities receiving the CTH intervention had a 37% reduction in the adjusted rate of polysubstance use involving an opioid and psychostimulant other than cocaine (likely methamphetamines) overdose deaths, a statistically significant reduction. Rates of overdose deaths involving an opioid and cocaine and those involving an opioid and benzodiazepines showed 6% and 1% reductions, respectively, which did not reach statistical significance. We found high prevalence of polysubstance use, consistent with prior research on overdose deaths in the US. Deaths from polysubstance use are likely more reflective of the current reality of the opioid crisis than deaths from opioids alone.^32,33^ Cases with at least 1 opioid and a psychostimulant represented more than 40% of opioid overdose fatalities in this study. Fentanyl in combination with psychostimulants is a major reason for the continued rise in overdose deaths, suggesting a need to better understand and treat polysubstance use.^34^

Naloxone, as an opioid antagonist, is likely to significantly reduce deaths related to an opioid and other substances, including opioid-psychostimulant combinations. MOUD, through occupying the μ receptors, reduces risk of opioid overdose deaths (whether alone or in combination with stimulants) by decreasing the number of times an individual uses opioids and risk exposures.

In response to community concern about overdose deaths associated with opioid-involved polysubstance use, a communications campaign was developed focusing on the potential benefit of using naloxone for all overdoses. Materials were created specifically addressing the widespread presence of fentanyl in the drug supply as a risk to people using stimulants and counterfeit prescription pills with no opioid tolerance. All intervention communities implemented these messages. This campaign, along with a focus on OEND, may have increased awareness of the importance of naloxone availability for any nonprescribed substance use. Thus, the CTH’s focus on increasing OEND through EBP strategies and communication campaigns, regardless of substance used, may be a mechanism for reducing overdose deaths from opioid-involved polysubstance use.^35^

The increased use of naloxone and OEND messaging should have lowered overdose deaths involving a stimulant and opioid combination. However, the reductions in overdose deaths noted for opioid and cocaine (6%) were much smaller than those noted for opioid and psychostimulants other than cocaine (37%). The rates also varied considerably by state. The adjusted rate for cocaine-involved deaths were highest in New York and lowest in Kentucky, while psychostimulant other than cocaine-involved deaths were highest in Kentucky where rates were 7-fold higher than those in Massachusetts (which had the lowest rates) (Table 3). These deaths are likely to be due to methamphetamine use. Historically, in the US, methamphetamine use has been more prevalent in rural areas, whereas cocaine tends to be more common in urban areas,^36^ although this may be changing.^37^ Subgroup analyses of the effects on urban and rural communities consistently show that rural HCS communities had greater, nonsignificant reductions in death rates for the central and secondary outcomes. Thus, the differential effects might be due to a larger reach of OEND and the media messaging in rural areas with smaller populations. The lack of a national real-time surveillance system for drug supply and overdose deaths makes it more difficult for communities to respond efficiently to the changing nature of the drug crisis.^38^ This is evident in the evolution of the opioid crisis, which now includes psychostimulants, and a continuing rise in opioid-involved polysubstance overdose deaths.^39,40,41^

### Limitations

This study had several limitations. External factors may have limited our ability to detect differences in some of our outcomes. First, death rates from overdoses increased substantially during the early stages of the COVID-19 pandemic across all the communities, with no state returning to their 2019 baseline during the study period. Second, the expansion of fentanyl in the drug supply of psychostimulants, counterfeit pills, and other drugs during this period increased risk of an overdose fatality even when there was no intent to use an opioid.^3,4^ Third, COVID-19 overwhelmed public health departments responding to both COVID-19 and the increase in overdose deaths. This led to a delay in EBP implementation so that after selection of EBPs, only 10 months remained for implementation.^21^ Only 38% of EBPs were implemented before the comparison period. Had EBP strategies been implemented earlier, they might have gained greater penetration, reached more people in need, and shown a stronger effect on overdose deaths. Assessing the effects of the intervention over a longer postintervention period might have resulted in a greater reduction in deaths. Additionally, the HCS was implemented as a randomized clinical trial with wait-list comparison communities serving as control communities. However, during the study, significant outside funding became available to communities via various federal and state initiatives to reduce opioid and polysubstance overdose deaths. The extent to which funding was successfully obtained and used to implement similar EBPs in control communities during the intervention period is unknown and could be a confounding variable.

## Conclusions

In this clinical trial examining the effects of the CTH intervention, the intervention communities had a lower adjusted rate of overdose fatalities by a nonsignificant 8% and a statistically significant 37% reduction in the adjusted rate of deaths among those using an opioid and psychostimulant other than cocaine. In addition to reducing opioid and psychostimulant deaths other than cocaine, the CTH was successful at helping communities identify and implement intervention components that they felt would be most feasible and impactful for their communities. Population-level interventions that utilize local community coalitions to help implement proven EBPs to reduce opioid-involved overdose deaths can also be effective at reducing polysubstance overdoses that include an opioid. Resources that help local communities identify their needs and stand-up practices that are known to reduce opioid overdose deaths would be worthy of resources. Continued research of the model that is powered for smaller reductions in opioid overdose deaths is also warranted.

## Notes

Supplement 1.
HEALing Communities Study Protocol and Statistical Analysis Plan

Supplement 2. **eTable 1.** Descriptive Sums of Primary and Secondary Outcomes During the Baseline Period (January 2019-December 2019) Using the Intention-to-Treat Population **eTable 2.** Descriptive Means of Primary and Secondary Outcomes During the Evaluation Period (July 1, 2021-June 30, 2022) Using the Intention-to-Treat Population **eTable 3.** Descriptive Sums of Tertiary Outcomes During the Evaluation Period (July 1, 2021-June 30, 2022) Using the Intention-to-Treat Population **eTable 4.** Calculation of Proportions of Opioid Deaths Involving Specific Drugs **eTable 5.** Adjusted Rate of Each Outcome Within Trial Groups and Adjusted Relative Rate of Each Outcome Between Intervention to Control Communities During the Evaluation Period Using the Per-Protocol Population **eTable 6.** Sensitivity Analyses

Supplement 3.
Data Sharing Statement
