# Error in Byline

**DOI:** 10.1001/jamanetworkopen.2025.15507

**Published:** 2025-05-09

In the Original Investigation titled “Communities That HEAL Intervention and Mortality Including Polysubstance Overdose Deaths: A Randomized Clinical Trial,”^1^ published October 21, 2024, the name of coauthor Mia-Cara Christopher appeared incorrectly in the byline. This article has been corrected.^1^

## References

[1] Freisthler B, Chahine RA, Villani J, . Communities that HEAL intervention and mortality including polysubstance overdose deaths: a randomized clinical trial. JAMA Netw Open. 2024;7(10):e2440006. doi:10.1001/jamanetworkopen.2024.40006 39432308 PMC11581668

