# Supplementary material for: Communities That HEAL Intervention and Mortality Including Polysubstance Overdose Deaths: A Randomized Clinical Trial
Source: JAMA Netw Open. 2024 Oct 21;7(10):e2440006. doi: 10.1001/jamanetworkopen.2024.40006 (PMC11581668; doi:10.1001/jamanetworkopen.2024.40006)
Supplement: Supplement 1. — HEALing Communities Study Protocol and Statistical Analysis Plan [file jamanetwopen-e2440006-s001.pdf]

The HEALing Communities Study

Protocol Number: Pro00038088

National Clinical Trial (NCT) Identified Number: NCT04111939

Principal Investigators

DCC: Emmanuel Oga, MD, MPH; Gary Zarkin, PhD; LaShawn Glasgow, DrPH, MPH

KY: Sharon Walsh, PhD

MA: Jeffrey H. Samet, MD, MA, MPH

NY: Nabila El-Bassel, PhD

OH: Bridget Freisthler, PhD

NIDA: Redonna Chandler, PhD

SAMHSA: Yngvild Olsen, MD, MPH

Sponsor: RTI International

Grant Title: Data Coordinating Center

Grant Numbers

DCC: 1 UM1 DA049394

KY: 1 UM1 DA049406

MA: 1 UM1 DA049412

NY: 1 UM1 DA049415

OH: UM1DA049417

Funded by the National Institute on Drug Abuse

Version 1.21

March 6, 2024

CONFIDENTIALITY STATEMENT

This document is confidential communication. Acceptance of this document constitutes agreement by the recipient that no unpublished information contained herein will be published or disclosed without prior approval of the Principal Investigators or other participating study leadership and consistent with the National Institutes of Health terms of awards.

Contents

Contents

STATEMENT OF COMPLIANCE 6

1. PROTOCOL SUMMARY 6
   - 1.1 Synopsis 6
   - 1.2 Timeline 8
2. INTRODUCTION 9
   - 2.1 Study Rationale 9
   - 2.2 Background 10
   - 2.3 Risk/Benefit Assessment .... 11~~12~~
     - 2.3.1 Known Potential Risks .... 11~~12~~
     - 2.3.2 Known Potential Benefits .... 13~~14~~
     - 2.3.3 Assessment of Potential Risks and Benefits 14
3. OBJECTIVES AND OUTCOMES 14
4. STUDY DESIGN 20
   - 4.1 Overall Design 20
     - 4.1.1 Trial Duration 20
     - 4.1.2 Wave 1 Communities .... 20~~21~~
     - 4.1.3 Wave 2 Communities .... 20~~21~~
     - 4.1.4 Assignment to Study Wave 1 and Wave 2 Communities .... 20~~21~~
   - 4.2 Scientific Rationale for Study Design 21
   - 4.3 Justification for Intervention .... 21~~22~~
   - 4.4 End-of-Study Definition .... 21~~22~~
5. STUDY POPULATION 22
   - 5.1 Inclusion Criteria 22
   - 5.2 Exclusion Criteria 23
   - 5.3 Strategies for Recruitment and Retention 23
6. STUDY INTERVENTION(S) 24
   - 6.1 Study Intervention(s) Administration 24
     - 6.1.1 Study Intervention Description .... 24~~25~~
   - 6.2 Fidelity .... 55~~56~~

6.2.1 Fidelity Measures for CTH Community Engagement Intervention 5556

6.3 Implementation Science Measures for Communities That HEAL 6061

6.3.1 Study Participants 6162

6.3.2 Recruitment Procedures 6263

6.3.3 Compensation 6465

6.3.4 Instruments 6465

6.4 Toxicology Survey 6869

6.5 Measures to Minimize Bias: Randomization and Blinding 6869

7. STUDY INTERVENTION DISCONTINUATION AND PARTICIPANT DISCONTINUATION/WITHDRAWAL 6970

7.1 Discontinuation of Study Intervention 6970

7.2 Participant Discontinuation/Withdrawal From the Study 7071

7.3 Lost to Follow-Up 7071

8. INSTRUMENTS AND PROCEDURES 7071

8.1 Instruments 7071

8.2 Safety Assessments 129130

8.3 Adverse Events 131132

8.3.1 Definition of Adverse Events and Serious Adverse Events 131132

8.3.2 Time Period and Frequency for Event Assessment and Follow-Up 132133

8.4 Unanticipated Problems 133134

8.4.1 Definition of Unanticipated Problems 133134

8.4.2 Unanticipated Problem Reporting 133134

8.4.3 Reporting Unanticipated Problems to HCS Communities 134135

9. STATISTICAL CONSIDERATIONS 134135

9.1 Statistical Hypotheses 134135

9.2 Sample Size Determination 134135

9.3 Populations for Analyses 136137

9.4 Statistical Analyses 136137

9.4.1 General Approach 136137

9.4.2 Analysis of the Primary Outcome 137138

9.4.3 Analysis of the Secondary Outcomes 138139

9.4.4 Baseline Descriptive Statistics 138139

9.4.5 Subgroup Analyses .... 138~~139~~

9.4.6 Exploratory Analyses .... 138~~139~~

10. SUPPORTING DOCUMENTATION AND OPERATIONAL CONSIDERATIONS .... 139~~140~~

10.1 Regulatory, Ethical, and Study Oversight Considerations .... 139~~140~~

10.1.1 Informed Consent .... 139~~140~~

10.1.2 Certificate of Confidentiality .... 140~~141~~

10.1.3 Confidentiality and Privacy .... 141~~142~~

10.1.4 Future Use of Stored Specimens and Data .... 141~~142~~

10.1.5 Key Roles and Study Governance .... 141~~142~~

10.1.6 Safety Oversight .... 143~~144~~

10.1.7 Quality Assurance and Quality Control .... 144~~145~~

10.1.8 Data Handling and Record Keeping .... 145~~146~~

10.1.9 Protocol Deviations .... 149~~150~~

10.1.10 Publication and Data Sharing Policy .... 149~~150~~

10.2 Abbreviations and Special Terms .... 151~~152~~

10.3 Protocol Amendment History .... 153~~154~~

11. REFERENCES .... 158~~159~~

12. APPENDICES .... 165~~166~~

12.1 Appendix A: Study Outcomes .... 165~~166~~

12.1.1 Primary Outcomes .... 165~~166~~

12.1.2 Secondary Outcomes .... 166~~167~~

12.1.3 Structural Outcomes .... 178~~179~~

12.2 Appendix B: Health Message Testing Service Questions—Centers for Disease Control and Prevention .... 188~~189~~

12.3 Appendix C: Justice Community Opioid Innovation Network (JCOIN) and HCS Annual Jail Survey .... 204~~205~~

12.4 Appendix D: Additional Site-Led HCS Research Activities .... 208~~209~~

12.4.1 Group Model Building Workshop .... 208~~209~~

12.4.2 GetNaloxoneNow .... 208~~209~~

12.4.3 Brandeis University Collaboration to Conduct Interviews with Health Care Payers .... 209~~210~~

12.4.4 Academic Detailing and CECentral Online Modules .... 210~~211~~

12.4.5 MOUD Organization Interview Guide .... 211~~212~~

12.4.6 Pharmacy Study Interview Guide & Survey ..... 212~~213~~

12.4.7 CE Facilitator Community Genogram Model ..... 213~~214~~

12.4.8 Policy Barriers and Facilitators Tracking ..... 214~~215~~

12.4.9 Costing the Evidence Based Practices ..... 215~~216~~

12.4.10 Identifying the Municipal Policies that Impact the Implementation of the EBP Strategies ..... 216~~217~~

12.4.11 Photovoice Participatory Research Technique ..... 217~~218~~

12.4.12 Race and Ethnicity Data Collection Readiness Survey ..... 221~~222~~

12.4.13 PARTNER Tool ..... 221~~222~~

12.4.14 Medication Disposal Program Pharmacy Interviews ..... 222~~223~~

12.4.15 Mobile Interventions for Increasing Access to Medications for Opioid Use Disorder (MOUD) ..... 223~~224~~

12.4.16 A Cost Analysis of Peer Recovery Support Services ..... 224~~225~~

12.4.17 Wave 1 Partner Organization Sustainability Interview ..... 226~~227~~

TABLES

Table 1: Primary and Key Secondary Study outcomes 15

Table 2: Other Secondary Study outcomes 16

Table 3: Additional RS-specific inclusion criteria for communities enrolled in the HCS ..... 22~~23~~

Table 4: Overview of communication campaigns ..... 44~~45~~

Table 5: Campaign Prepare-Plan-Implement process steps ..... 47~~48~~

Table 6a: Timeline for Stages of Communication Campaigns (Wave 1) ..... 48~~49~~

Table 6b: Timeline for Stages of Communication Campaigns (Wave 2) ..... 49~~50~~

Table 7: Measures by data collection point ..... 53~~54~~

Table 8: Fidelity measures and implementation strategies ..... 56~~57~~

Table 9: Instrument name and associated informed consent form name ..... 71~~72~~

Table 10: Instrument Summaries ..... 76~~77~~

Table 11: Metrics Used to Declare a Safety Signal in AE/SAE Review Process ..... 132~~133~~

Table 12: Power calculation for underlying reduction in risk of opioid overdose fatalities between Wave 1 and Wave 2 communities ..... 135~~136~~

Table 13: Key roles and study governance ..... 141~~142~~

Table 14: Data and Safety Monitoring Board members ..... 144~~145~~

Table 15: Protocol Amendment History ..... 153~~154~~

FIGURES

Figure 1: HEALing Communities Study timeline 9
Figure 2: Three components of the CTH intervention 2425
Figure 3: Conceptual framework of the CTH intervention 2526
Figure 4a: CTH intervention phases (Wave 1) 2627
Figure 4b: CTH intervention phases (Wave 2) 2728
Figure 5: Phase 3 parallel processes for developing community profiles and data dashboards (Wave 1) 3334
Figure 6: Schedule of Activities for Maintaining the CTH Beyond Year 1 (Wave 1) 3738
Figure 8: The Plan-Prepare-Implement model for campaign development and community engagement........ 4647
Figure 9: RE-AIM/PRISM model adapted for HCS 6162
Figure 10: Two-Step AE/SAE Review Process........ 130131

STATEMENT OF COMPLIANCE

The trial will be carried out in accordance with International Conference for Harmonisation of Technical Requirements for Pharmaceuticals for Human Use (ICH) Good Clinical Practice (GCP).

National Institutes of Health (NIH)-funded investigators and clinical trial site staff who are responsible for the conduct, management, or oversight of NIH-funded clinical trials have completed human subjects protection and ICH GCP training.

The protocol, informed consent form(s), recruitment materials, and all participant materials will be submitted to the Institutional Review Board (IRB) for review and approval. Approval of the protocol and the consent forms must be obtained before the communities are randomized or any participant is consented. Any amendment to the protocol will require review and approval by the IRB before the changes are implemented in the study. All changes to the consent forms will be IRB approved; a determination will be made regarding whether a new consent needs to be obtained from participants who provided consent.

1. PROTOCOL SUMMARY

1.1 Synopsis

**Title**	The HEALing Communities Study (HCS)
**Grant Numbers**	UM1-DA049415, UM1-DA049417, UM1-DA049412, UM1-DA049406, UM1-DA049394
**Study Description**	The HCS is a multi-site trial evaluating the impact of the Communities That HEAL (CTH) intervention compared with usual care in wait-list communities. The CTH is a community-engaged intervention that provides a comprehensive, data-driven community response plan to deploy evidence-based practices (EBPs) across multiple sectors to reduce opioid-related overdose deaths and associated outcomes.<br><br>The HCS refers to the communities randomized to receive the CTH intervention first as “Wave 1 communities” and those in the wait-list comparison arm as “Wave 2 communities.” Wave 1 communities will implement the CTH intervention for 30 months, during which time Wave 2 communities will provide usual care. At month 31, Wave 2 communities begin to implement the CTH intervention.
**Objectives**	The HCS will test the impact of the CTH intervention on opioid overdose deaths and associated outcomes in 67 highly affected communities in Kentucky, Massachusetts, New York, and Ohio. The goal is to reduce opioid overdose deaths by 40%.

**Outcomes**	The primary outcome is the number of opioid overdose deaths. Secondary outcomes include (1) the number of naloxone units distributed, (2) the number of individuals with opioid use disorder who receive buprenorphine for opioid use disorder, and (3) incident high-risk opioid prescribing. The primary comparison of interest is between Wave 1 communities compared with Wave 2 communities during the second 12-month period after Wave 1 communities are activated. The primary analysis will use an intention-to-treat design with the population defined as any community randomized into the HCS.<br><br>The HCS will measure additional outcomes for secondary data-analytic purposes. A full list of outcomes appears in Appendix A.
**Study Population**	A total of 67 communities across four states (Kentucky, Massachusetts, New York, Ohio) were selected to participate in the HCS. At least 30% of the communities selected in each state were required to be rural. Collectively, the communities in each state were required to have at least 150 opioid-related overdose fatalities (at least 15% occurring in rural communities) and a rate of 25 opioid-related overdose fatalities per 100,000 people or higher in 2016. Kentucky selected 16 counties to participate, Massachusetts selected 16 cities/towns, New York selected 13 counties and 3 cities/towns, and Ohio selected 19 counties.
**Study Design**	The HCS is a multi-site, parallel arm, cluster randomized, wait-list controlled trial evaluating the impact of the CTH intervention compared with usual care in wait-list communities.
**Description of Study Intervention**	The CTH is a community-engaged intervention that provides a comprehensive, data-driven community response plan to deploy EBPs across multiple sectors to reduce opioid overdose deaths and associated outcomes across HCS communities. The CTH intervention seeks to promote a common vision, shared goals, and tailored strategies to mobilize HCS communities to adopt EBPs using a stepwise community change process that integrates three components. The first CTH component, community engagement, includes seven phases to assist communities in developing a response specific to their opioid crisis: (0) Preparation (pre-intervention), (1) Getting Started, (2)

	Getting Organized, (3) Community Profiles and Data Dashboards, (4) Community Action Planning, (5) Implement and Monitor, and (6) Sustainability Planning (ongoing). The second CTH component, the Opioid-Overdose Reduction Continuum of Care Approach (ORCCA), facilitates each community's implementation of system- and practice-level changes to increase adoption of EBPs and reduce opioid overdose deaths. The ORCCA provides a menu of strategies for implementing three required EBPs: (1) opioid overdose prevention education and naloxone distribution (OEND) in high-risk populations; (2) effective delivery of MOUD maintenance treatment, including agonist/partial agonist medication, and including outreach and delivery to high-risk populations; and (3) safer opioid prescribing and dispensing. The third CTH component is a communication campaign. Community-based communication campaigns in each year will focus on different messages and priority groups in the communities. Campaign objectives support CTH implementation and outcomes and include (1) increasing demand for OEND and MOUD EBPs, (2) increasing availability and access to MOUD and naloxone, (3) increasing treatment retention, (4) increasing recovery support, (5) reducing stigma, and (6) reducing high-risk prescribing.
**Study Duration**	72 months

1.2 Timeline

The duration of the HCS is April 2019 through March 2025 (Figure 1). During this 6-year period, the CTH intervention will be carried out in two waves. Startup activities for the four academic Research Sites and the Data Coordinating Center began in April 2019 and continued through December 2019. Wave 1 communities have begun implementing the CTH intervention for 30 months (January 2020 through June 2022), during which time Wave 2 communities are providing usual care. The primary outcome measurements will be assessed in Wave 1 communities compared with Wave 2 communities from July 2021 through June 2022. At month 31 (July 2022), Wave 2 communities will begin to implement the CTH intervention for 18 months (July 2022 through December 2023). After the intervention period, Wave 1 communities will be observed for sustainment of the intervention for an 18-month period (July 2022 through December 2023). Data analysis and project closeout will occur from July 2023 through March 2025.

Figure 1: HEALing Communities Study timeline

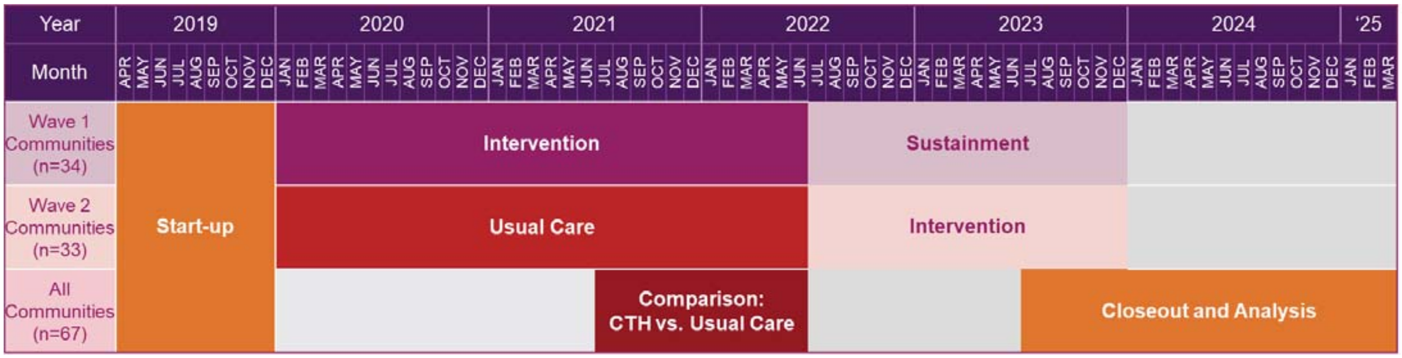

2. INTRODUCTION

2.1 Study Rationale

Communities across the United States are dealing with the catastrophic consequences of excessive availability and use of prescription opioids and illicit opioids such as heroin and illicitly manufactured fentanyl (and related analogs). Millions of Americans are struggling with inappropriate use of opioids and opioid use disorder (OUD). The consequences of this crisis are grave with tens of thousands continuing to die each year in the United States from opioid overdose. In addition, rates of OUD, injection drug use, acute hepatitis C virus infections, localized outbreaks of the human immunodeficiency virus, and other serious health conditions, such as endocarditis and neonatal abstinence syndrome, continue to rise.

One driver of the opioid crisis is the recognized gap between the number of individuals who could benefit from evidence-based treatment and prevention interventions to reduce opioid misuse and OUD versus those actually engaged in care. The National Survey on Drug Use and Health (NSDUH) estimates that 2.1 million Americans have OUD, yet fewer than 20% of those individuals receive specialty care in a given year. A menu of evidence-based practices (EBPs) exists, including opioid overdose education and naloxone distribution (OEND) programs; prescription drug monitoring programs and other strategies to reduce inappropriate opioid prescribing; Food and Drug Administration–approved medication for opioid use disorder (MOUD) including methadone, buprenorphine, and naltrexone; behavioral therapies; and recovery support services. Unfortunately, these EBPs have largely failed to penetrate community settings including addiction treatment, general medical care, social support services, schools, and the justice system. This failure is in part due to a lack of evidence-based approaches for assisting communities in the development and deployment of a data-driven, customized response strategy to adopt, deliver, and use comprehensive integrated EBPs.

The HEALing Communities Study (HCS) is a 5-year multi-site, parallel arm, cluster randomized, wait-list controlled trial to test the impact of a community-engaged intervention designed to increase the adoption of an integrated set of EBPs delivered across health care, behavioral health, justice, and other community-based settings. The primary goal is to reduce opioid-related overdose deaths by 40% in highly affected communities. A total of 67 communities in the states of four RSs (Kentucky, Massachusetts, New York, and Ohio) will be enrolled in the study to measure the impact of the intervention.

The HCS will test the Communities That HEAL (CTH) intervention, a conceptually driven framework based on the Communities That Care model, for its effectiveness in organizing evidence-based overdose prevention efforts in communities. The CTH intervention seeks to promote a common vision, shared goals, and tailored strategies to mobilize HCS communities to adopt EBPs using a stepwise community change process that integrates three components. The first CTH component, community engagement, includes a seven-phase coalition-driven process designed to promote community-specific approaches. The second component of the CTH intervention entails facilitating each community's implementation of system- and practice-level changes to rapidly reduce the rate of opioid-related overdose fatalities. The conceptual and operational framework guiding this component is titled the Opioid-Overdose Reduction Continuum of Care Approach (ORCCA). The ORCCA provides a menu of strategies for implementing EBPs designed to help communities reduce opioid overdose deaths and includes three mandatory EBPs: (1) OEND in high-risk populations; (2) effective delivery of MOUD maintenance treatment, including agonist/partial agonist medication, and including outreach and delivery to high-risk populations; and (3) safer opioid prescribing and dispensing. As noted in required EBPs 1 and 2, identification of, and intervention with, high-risk populations is an ORCCA requirement. The third CTH component utilizes the Prepare-Plan-Implement (PPI) model to design five community-based communication campaigns that focus on different messages and priority groups in the HCS communities (e.g., community leaders; criminal justice, public health, public safety, and medical staff; people with an OUD and their families; at-risk patients and their families). Campaign objectives include (1) obtaining and carrying naloxone; (2) decreasing MOUD stigma; (3) raising awareness of MOUD treatment; (4) initiating MOUD treatment; and (5) staying in MOUD treatment. Another overarching objective is for the campaigns to set a public agenda to increase community efforts to reduce opioid OD deaths.

2.2 Background

The U.S. opioid overdose epidemic has been declared a national emergency.[1,2] Overdose deaths from prescription opioids, illicit synthetic opioids, and heroin continue to increase. There were more than 350,000 deaths from 1999 to 2016[3] and 47,600 deaths in 2017.[4] In recent years, the deadly surge of the availability and use of illicit fentanyl and fentanyl analogs has been driving increases in overdose deaths.[5]

These opioid-related overdose deaths reflect, in large part, a lack of treatment of OUD.[6] As mentioned, the most recent NSDUH indicates that 2.1 million Americans have OUD, although other sources suggest the number is closer to 5 million.[7-9] Fewer than 20% of individuals with OUD have received any form of OUD treatment in the past year.[10] In a cohort of opioid overdose survivors, fewer than one-third received any MOUD treatment within a year of the overdose event.[11]

The treatment gap reflects three major challenges: (1) many individuals with OUD do not perceive a need for treatment, (2) there is insufficient treatment capacity, and (3) treatment retention is suboptimal. National data indicate that among individuals with OUD who are not in treatment, a lack of recognition of the disorder is a major impediment to seeking treatment.[12,13] Furthermore, many individuals have internalized stigma about OUD that prevents them from seeking treatment.[14] In addition to these challenges, capacity for delivering MOUD is an ongoing problem in many areas. Most of the nation's 1,100 opioid treatment programs (i.e., federally licensed methadone programs; OTPs) are located in urban centers, and growth in the number

of OTPs has been modest in the past decade.[15] Buprenorphine, in contrast to methadone, is more widely delivered in office-based addiction treatment in the United States.[16] The number of U.S. buprenorphine-waivered physicians[17] and buprenorphine dispensing have increased[18] but remain insufficient to meet the national need for treatment. Even with increasing numbers of patients receiving MOUD, treatment retention is poor, and the percentage of coverage has declined from 2010 (25%) to 2014 (16%) due to increasing numbers of affected individuals.[19]

In addition to treatment-related challenges that are fueling the opioid epidemic, two other factors—the suboptimal uptake of overdose prevention and the opioid-prescribing behaviors that enhance overdose risks—are significant drivers of the national epidemic. Naloxone effectively reverses opioid overdose, thus preventing fatalities. Although demonstration projects have shown that community distribution of naloxone can reduce the rate of opioid fatalities, national data show very limited prescribing of naloxone (including distribution through standing orders at pharmacies).[20] Opioid overdose deaths also reflect continuing patterns of risky prescribing, such as the co-prescription of benzodiazepines with opioids, the prescription of high doses of opioids (i.e., >90 morphine milligram equivalents per day), and long-term prescriptions of opioids. These prescribing behaviors increase the risk of overdose even among individuals without OUD and heighten the risk of developing an OUD.

For these reasons, it is necessary to change the course of the epidemic using a data-driven, multi-pronged approach. The HCS will test the immediate impact of implementing the CTH intervention on opioid overdose deaths in highly affected communities with the goal of reducing opioid overdose deaths by 40%. A recent modeling study considered multiple interventions across the spectrum of prevention to treatment and concluded that the most effective policy interventions for reducing overdose deaths are the expansion of naloxone availability and access to MOUD.[2] Therefore, the HCS will engage communities to reach individuals who are at highest risk of overdose death (e.g., out of treatment, leaving jail) and (1) expand access to and receipt of MOUD and behavioral treatment, (2) increase the number of individuals retained in treatment beyond 6 months, (3) reduce the risk of fatal overdose through expansion of OEND, and (4) improve prescription opioid safety.

2.3 Risk/Benefit Assessment

2.3.1 Known Potential Risks

This section considers immediate potential risks that affect the following categories of participants:

- Communities enrolled in the study
- Individual study participants: Research Site (RS) Community Advisory Board members, community coalition members, and professionals working in service venues in a community who will provide data
- People with lived experiences in the communities
- Individuals whose records are included as part of the secondary data analyses

2.3.1.1 Communities Enrolled in the Study

The reputation of the participating communities could be put at risk through identification as areas where opioid use and misuse have had significant impact. This could affect such things as tourism, desire for workers or employers to relocate to a community, housing and other real estate values, and the general sense of well-being in a community. However, the information on the level of impact of opioid misuse and OUD on communities is already widely available to the public. News coverage on opioid-related information, such as overdose deaths, by county or other local geographic area, is regularly publicized. Information about the number of opioid deaths by county is also widely available. Thus, community participation in the HCS is unlikely to affect the reputation of a community beyond what is known based on currently available information. In fact, a community will likely consider participation in the HCS a positive action. Stigma reduction is also embedded in messaging campaigns in all participating communities. These messaging campaigns will raise public awareness of participating communities' commitment to mobilizing an effective response that will address the opioid overdose epidemic with the potential to strengthen the delivery of health care and behavioral health services overall. Furthermore, participation in the HCS may result in a reduction of opioid-related morbidity and mortality, thereby yielding long-term economic benefit.

2.3.1.2 Individual Study Participants

Individual stakeholders in a community will be asked to provide information about the opioid-related services currently being offered, their ability to offer new services, attitudes that might affect the success of service offerings, and the cost of offering services. It is expected that risks with such data collection will be minimal. Participants will not be asked to provide personal information other than basic demographic information and perceptions of access and barriers to community services. Participants will also not be asked questions that would put them at risk of criminal or civil liability or cause damage to financial standing, employability, or reputation. Some questions, although not structured or intended to do so, could invite feelings of discomfort for respondents. Informed consent will acknowledge this risk of discomfort, consent documents will clearly state that participation is entirely voluntary, and individuals may refuse to answer any or all questions and stop participating at any time without penalty.

As with all research, there is a risk of breach of confidentiality. To minimize this risk, RSs will use practices to securely store data and to separate identifying information from research data. Research staff will make every effort to protect participant privacy and confidentiality of the Community Advisory Board, community coalition members, communication research participants, and other key stakeholders, who will be providing data. Involvement in the study or data collected by the study could become known to other participants. The following steps will be taken to protect against a breach of confidentiality. First, all data, including quantitative assessments, digital recordings, and qualitative interview transcripts will be labeled only with study/subject identification numbers, and no participant names or other identifying information will be attached to study data. Second, any document with study coding that assigns an identification number to a participant name will be kept by project directors or study staff designated by the Principal Investigator in locked file cabinets separate from research data or in a password-protected file on an encrypted endpoint device. Third, electronic data files or databases will be stored in secure, password-protected systems that use appropriate safeguards against unauthorized access. Data sent from the research sites to the Data

Coordinating Center (DCC) at RTI International will be securely transferred and encrypted (see *10.1.8 Data Handling and Record Keeping* for additional information). Fourth, all research data on paper will be kept in locked file cabinets and will be available only to research staff directly involved in this project and National Institute on Drug Abuse and Substance Abuse and Mental Health Services Administration staff, or their designees. Fifth, all study staff will receive training on procedures to protect participant confidentiality and will take all required courses and certification tests (e.g., human subjects protection and International Conference for Harmonisation of Technical Requirements for Pharmaceuticals for Human Use Good Clinical Practice training). Sixth, participants will be informed that all data are confidential within the limits of the law.

2.3.1.3 People With Lived Experiences in the Communities

The most affected people living in a community are those with OUD and their friends and relatives. The risks to these individuals are low because the CTH intervention is a process of community engagement designed to help communities select and implement EBPs to mitigate the impacts of the opioid epidemic in the community. The HCS will not be testing new programs where the risks and potential adverse effects are unknown. Previously proven EBPs will be selected by the communities where the risks and benefits have been previously studied and found to be properly balanced.

2.3.1.4 Individuals Included in Secondary Data

RSs will have access to identified data and protected health information from secondary administrative data sources. The main risk to individuals is related to data security; there are no health risks to participants. All individual-level data will be stored on local secured servers, with dedicated Information Systems personnel managing data security. Access to the data will only include Information Systems personnel responsible for their security and specified HCS personnel. No hard copies of raw data will be made. To protect participant welfare, all data from secondary sources will be generalized and de-identified before publication. As required by the Health Insurance Portability and Accountability Act of 1996 (HIPAA), if participants request, they would be allowed to know that their records were used in this study, as their rights allow. Any secondary data transmitted to the Data Coordinating Center will be de-identified; no individually identifiable data will be released outside of the RS's secured data environment.

2.3.2 Known Potential Benefits

Participation in the HCS has the potential to assist communities by developing an effective response to reduce opioid-related overdose fatalities and by improving the delivery of health and behavioral health treatment. HCS participation may also yield long-term economic benefits due to the reduction of opioid-related morbidity and mortality. Communities will receive resources from the HCS (e.g., personnel) to assist in the development of a data-driven response strategy to reduce opioid-related overdose fatalities. Resources will also be provided to assist in the selection and implementation of EBPs. These will include increasing the delivery of OEND and MOUD and identifying people who may have lost tolerance for opioids and are at a high risk for overdose in order to engage them in care. Communities will also receive support for the implementation of messaging campaigns. Communities participating in the HCS may also benefit from greater coordination and integration of efforts among agencies and

stakeholders in the community. As part of their involvement with the HCS, communities will have not only better monitoring and decision tools and processes for the community, but also better coordination and partnerships across different sectors of the community to address the opioid crisis and related problems.

2.3.3 Assessment of Potential Risks and Benefits

Communities participating in the HCS experience higher-than-average opioid-related overdose fatalities. The potential benefits of participating in the HCS outweigh the potential risk of negative community perception. Furthermore, some individuals participating in interviews, focus groups, or surveys may experience psychological distress; however, these risks are minimal and not long lasting.

3. OBJECTIVES AND OUTCOMES

The HEALing Communities Study (HCS) will test the impact of the Communities That HEAL (CTH) intervention on opioid overdose deaths and associated outcomes in 67 highly affected communities in Kentucky, Massachusetts, New York, and Ohio. The goal is to reduce opioid overdose deaths by 40%.

The HCS is a multi-site, parallel arm, cluster randomized, wait-list controlled trial evaluating the impact of the CTH intervention compared with usual care in wait-list communities. The CTH is a community-engaged intervention that provides community-based communication campaigns along with a comprehensive, data-driven community response plan to deploy evidence-based practices across multiple sectors to reduce opioid-related overdose deaths and associated outcomes.

As described, the HCS refers to the communities randomized to implement the CTH intervention first as Wave 1 communities and those in the wait-list comparison arm as Wave 2 communities. Wave 1 communities will implement the CTH intervention for 30 months, during which time Wave 2 communities will provide usual care. At month 31, Wave 2 communities will begin to implement the CTH intervention.

The HCS has one primary hypothesis (H1) and three secondary hypotheses (H2, H3, H4). Compared with Wave 2 communities, we hypothesize that Wave 1 communities will accomplish the following:

- H1: Reduce opioid overdose deaths.
- H2: Increase naloxone distribution.
- H3: Expand use of buprenorphine for opioid use disorder.
- H4: Reduce high-risk opioid prescribing.

The primary analysis will compare Waves 1 and 2 during the 12-month period of July 2021 through June 2022. This comparison period begins 18 months after Wave 1 communities implement the CTH intervention and before Wave 2 communities implement the CTH intervention.

The primary outcome is the number of opioid overdose deaths. Key secondary outcomes include (1) the number of naloxone units distributed in the community, (2) the number of

individuals with opioid use disorder (OUD) who receive buprenorphine, and (3) incidents of high-risk opioid prescribing. The primary comparison of interest is between Wave 1 and Wave 2 communities during the 12-month parallel-arm period. The primary analysis will use an intention-to-treat design with the population, defined as any community randomized into the HCS.

In addition to testing these hypotheses, the HCS seeks to determine (1) the factors that contribute to or impede successful implementation of the CTH intervention, (2) the factors that contribute to or impede sustainment of CTH intervention, and (3) the incremental costs and cost effectiveness of the CTH intervention.

The HCS will measure additional outcomes for secondary data-analytic purposes. Lists of primary and secondary study outcomes appear in Tables 1 and 2, respectively. A more complete description of each outcome and its data source appears in Appendix A.

Table 1: Primary and Key Secondary Study outcomes

Hypothesis	Associated Outcomes	Appendix A Reference Number	Data Source
1	Number of opioid overdose deaths (Primary Outcome)	1	Death certificates, supplemented (if needed) with medical examiner, coroner, and toxicology data
2	Number of naloxone units distributed in communities (Secondary Outcome)	2.14.3	Combination of dispensed prescriptions data purchased from IQVIA and state administrative sources on naloxone distribution
3	Number of individuals receiving buprenorphine products that are approved by the Food and Drug Administration (FDA) for treatment of OUD (Secondary Outcome)	2.5.1	Prescription drug monitoring programs
4	Incidents of high-risk opioid prescribing (Secondary Outcome)	2.13	Prescription drug monitoring programs

Table 2: Other Secondary Study outcomes

Hypothesis	Associated Outcomes	Appendix A Reference Number	Data Source
N/A	Number of drug overdose deaths	2.1	Death certificates, medical examiner, coroner, and toxicology data
N/A	Number of non-fatal drug overdose events	2.2	Hospital inpatient and emergency department (ED) billing claims
N/A	Number of non-fatal opioid overdose events	2.3	Hospital inpatient and ED billing claims
N/A	Number of individuals with OUD (prevalence)	2.4	Medicaid administrative data, including claims and eligibility files
N/A	Number of individuals receiving methadone	2.5.2	Medicaid administrative data, including claims and eligibility files
N/A	Number of individuals receiving naltrexone	2.5.3	Medicaid administrative data, including claims and eligibility files
NA	Number of individuals with OUD receiving MOUD	2.5.4	Medicaid administrative data, including claims and eligibility files
N/A	Number of individuals with OUD receiving behavioral health treatment	2.6	Medicaid administrative data, including claims and eligibility files, and all-payer claims data if available
N/A	Number of individuals receiving buprenorphine/naloxone retained beyond 6 months	2.7.1	Prescription drug monitoring programs
N/A	Number of individuals receiving methadone retained beyond 6 months	2.7.2	Medicaid administrative data, including claims and eligibility files
N/A	Number of individuals receiving naltrexone retained beyond 6 months	2.7.3	Medicaid administrative data, including claims and eligibility files
N/A	Number of individuals with MOUD retained in treatment beyond 6 months	2.7.4	Medicaid administrative data, including claims and eligibility files
N/A	Person-months in MOUD	2.7.5	Medicaid administrative data, including claims and eligibility files

Hypothesis	Associated Outcomes	Appendix A Reference Number	Data Source
N/A	Number of emergency medical services (EMS) naloxone administration events	2.8.1	State EMS data collected for national reporting to the National Emergency Medical Services Information System (NEMSIS)
N/A	Number of EMS runs for opioid-related incidents/overdoses	2.8.2	State EMS data collected for national reporting to the National Emergency Medical Services Information System (NEMSIS)
N/A	Number of individuals linked to MOUD after opioid overdose	2.9	Medicaid administrative data, including claims and eligibility files
N/A	Number of individuals linked to MOUD after release from prison	2.10	Medicaid administrative data, including claims and eligibility files linked to incarcerated individual files from state departments of corrections
N/A	Number of individuals provided MOUD while in jail	2.11	Primary data collection—survey<br>*(Justice Community Opioid Innovation Network [JCOIN]* and *HCS Annual Jail Survey)*
N/A	Number of individuals linked to MOUD after an opioid-related ED visit	2.12	Medicaid administrative data, including claims and eligibility files
N/A	Number of individuals with OUD who are screened, diagnosed, and treated for hepatitis C	2.15	Medicaid administrative data, including claims and eligibility files
N/A	Number of newly diagnosed HIV cases	2.16	State registry for HIV/AIDS reporting
N/A	Number of opioid-related overdoses treated in EDs and captured by syndromic surveillance data	2.17	Syndromic surveillance records (accessed via the Centers for Disease Control and Prevention's National Syndromic Surveillance Program Electronic Surveillance<br>System for the Early Notification of Community-based Epidemics (NSSP-ESSENCE) application or other state-based platforms)

Hypothesis	Associated Outcomes	Appendix A Reference Number	Data Source
N/A	Number of new acute opioid prescriptions limited to a 7-day supply	2.18	Prescription drug monitoring program
N/A	Opioid prescriptions from multiple prescribers or pharmacies	3.1	Prescription drug monitoring programs
N/A	Number of providers with a waiver under the Drug Addiction Treatment Act of 2000 (DATA 2000)	3.2	U.S. Drug Enforcement Administration's (DEA) Active Controlled Substances Act (CSA) Registrants Database
N/A	Number of providers with a DATA 2000 waiver who actively prescribe buprenorphine products that are FDA approved for OUD	3.3	DEA's Active CSA Registrants Database linked to prescription drug monitoring program data
N/A	Number of providers who actively prescribe buprenorphine products that are FDA approved for OUD	3.4	Prescription Drug Monitoring Program (PDMP) data
N/A	Number of jails initiating and linking people to MOUD	3.5	Primary data collection—survey *(Justice Community Opioid Innovation Network [JCOIN]* and *HCS Annual Jail Survey)*
N/A	Number of take-back drug drop boxes and events	3.8	State administrative data and DEA's Active CSA Registrants Database
N/A	Number of ED visits for BH (count visits)	4.1.1	Medicaid administrative data, including claims and eligibility files
N/A	Number of ED visits for non-BH (count visits)	4.1.2	Medicaid administrative data, including claims and eligibility files
N/A	Number of hospital/inpatient nights for non-detox BH (count nights)	4.2.1	Medicaid administrative data, including claims and eligibility files

Hypothesis	Associated Outcomes	Appendix A Reference Number	Data Source
N/A	Number of hospital/inpatient nights for detox (count nights)	4.2.2	Medicaid administrative data, including claims and eligibility files
N/A	Number of hospital/inpatient nights for non-BH (count nights)	4.2.3	Medicaid administrative data, including claims and eligibility files
N/A	Number of non-detox BH residential nights (count nights)	4.3.1	Medicaid administrative data, including claims and eligibility files
N/A	Number of BH detox residential nights (count nights)	4.3.2	Medicaid administrative data, including claims and eligibility files
N/A	Number of intensive BH outpatient visits (count nights)	4.4.1	Medicaid administrative data, including claims and eligibility files
N/A	Number of outpatient visits BH (count visits)	4.5.1	Medicaid administrative data, including claims and eligibility files
N/A	Number of outpatient visits non-BH (count visits)	4.5.2	Medicaid administrative data, including claims and eligibility files
N/A	Number of non-pain buprenorphine days supplied (count days supply)	4.6.1	Medicaid administrative data, including claims and eligibility files
N/A	Number of non-pain buprenorphine injections (count injections)	4.6.2	Medicaid administrative data, including claims and eligibility files
N/A	Number of opioid-related oral naltrexone days supplied (count days supply)	4.6.3	Medicaid administrative data, including claims and eligibility files
N/A	Number of opioid-related naltrexone injections (count injections)	4.6.4	Medicaid administrative data, including claims and eligibility files
N/A	Number of methadone days supplied (count calculated days supply)	4.6.5	Medicaid administrative data, including claims and eligibility files

Hypothesis	Associated Outcomes	Appendix A Reference Number	Data Source
N/A	Number of opioid pain medication days supplied (count days supply)	4.7.1	Medicaid administrative data, including claims and eligibility files
N/A	Number of non-opioid pain medication days supplied (count days supply)	4.7.2	Medicaid administrative data, including claims and eligibility files

4. STUDY DESIGN

4.1 Overall Design

The HEALing Communities Study (HCS) is a multi-site, parallel arm, cluster randomized, wait-list controlled trial evaluating the impact of the Communities That HEAL (CTH) intervention compared with usual care in wait-list communities.

4.1.1 Trial Duration

The HCS goes from April 2019 through March 2025. During this 6-year period, the CTH intervention will be carried out in two waves.

4.1.2 Wave 1 Communities

The HCS refers to the communities randomized to receive the CTH intervention first as Wave 1 communities. Wave 1 communities will implement the CTH intervention for 30 months (January 2020 through June 2022). After the intervention period, they will be observed for sustainment of the intervention for 18 months (July 2022 through December 2023).

4.1.3 Wave 2 Communities

The HCS refers to the communities randomized to receive the CTH intervention second (in the wait-list comparison arm) as Wave 2 communities. During the 30 months that the Wave 1 communities are receiving the intervention, Wave 2 communities will provide usual care (but not the CTH intervention). At month 31 (July 2022), Wave 2 communities will begin to implement the CTH intervention for 18 months (July 2022 through December 2023).

4.1.4 Assignment to Study Wave 1 and Wave 2 Communities

The 67 HCS communities will be randomly assigned to Wave 1 communities or Wave 2 communities. Randomization will be stratified by Research Site (RS) (i.e., Kentucky, Massachusetts, New York, and Ohio). In each RS, we will use covariate-constrained randomization[21,22] to ensure balance between Wave 1 and Wave 2 communities on three key

community characteristics at baseline: (1) opioid overdose death rate averaged over the prior 2 years, (2) population size, and (3) urban versus rural status. Covariate-constrained randomization sets limits on the differences in select variables between arms in a trial. For the HCS, we will cluster-randomize communities in each site (state) and constrain randomization to balance three community-level factors at baseline: (1) rural/urban status (equal for even numbers; no more than a difference of 1 for odd numbers), (2) less than 0.2 standard deviation difference in community population, and (3) opioid death rate. Given the nature of the research, there will be no blinding in this study.

4.2 Scientific Rationale for Study Design

The effectiveness of the CTH intervention is unknown but is designed to significantly reduce opioid-related mortality. Therefore, we chose to adopt the proposed parallel arm, cluster randomized, wait-list controlled trial design because it is feasible, ethically justified, and scientifically sound. We expect that there will be a lag (approximately 18 months) between the time that a community introduces the CTH intervention and when its effect on opioid overdose deaths will be observed; time is needed to ramp up programs and deliver services in order to accrue the benefit of the components of the CTH intervention. Furthermore, our calculations indicate that we have high power to detect the expected impact of the CTH intervention. Our trial design is ethically sound as Wave 2 (waitlist) communities will receive the CTH intervention after the primary outcome assessment period is completed in June 2022. The CTH intervention will be delayed for communities randomized to Wave 2, but Wave 2 communities will continue to provide usual care and will not be prohibited from using their own resources to adopt, enhance, or implement new methods of prevention and treatment during this time.

4.3 Justification for Intervention

One driver of the opioid epidemic is the recognized service access gap for individuals who could benefit from an evidence-based practice (EBP) to reduce opioid-related overdose fatalities. Unfortunately, the penetration of these EBPs into community settings has been insufficient. This inadequacy is due, in part, to a lack of evidence-based implementation approaches to assist communities in the development and deployment of a data-driven customized response strategy to comprehensively integrate and implement EBPs.

The CTH intervention is intended to assist communities in identifying community leaders, champions, and stakeholders willing to work collaboratively (through a local coalition) and develop a community response strategy to implement communication campaigns and EBPs with the goal of reducing opioid-related overdose mortality.

4.4 End-of-Study Definition

Study completion for Wave 1 communities is defined for the intervention and sustainment stages. The intervention ends when Wave 1 communities complete 30 months of CTH; the sustainment stage ends 18 months after completion of CTH. The primary outcome will be assessed in months 19–30, and sustainment will be measured in months 31–42. Study completion for Wave 2 communities will occur at the end of the 18-month CTH intervention.

5. STUDY POPULATION

The HEALing Communities Study (HCS) will enroll communities in Kentucky, Massachusetts, New York, and Ohio. In addition, interviews, surveys, and focus groups will be conducted with the Community Advisory Board and community coalition members, service providers, and individuals and families affected by the opioid crisis.

5.1 Inclusion Criteria

The National Institute on Drug Abuse (NIDA) selected four Research Sites (RSs) (in Kentucky, Massachusetts, New York, Ohio) for the HCS. In these four states, 67 communities were selected. To be selected for this study, a community must meet all the following criteria established by NIDA:

- The community must be located in one of the four participating states: Kentucky, Massachusetts, New York, or Ohio.
- Of the communities selected in each state, 30% or more must be rural.
- Across all the HCS communities in each state, there must be a minimum of 150 opioid-related overdose fatalities (at least 15% of which come from rural communities) and a rate of at least 25 opioid-related overdose fatalities per 100,000 people, based on 2016 data.
- The community must express willingness to address in its response strategy the implementation of medication for opioid use disorder, overdose prevention training, and naloxone distribution across the community.
- The community must express willingness to develop partnerships across health care, behavioral health, and justice settings for evidence-based practices to address opioid misuse, opioid use disorder, and overdoses.

In addition to the NIDA-defined eligibility criteria listed earlier, the RSs used additional eligibility criteria to further refine their site selection (see Table 3).

Table 3: Additional RS-specific inclusion criteria for communities enrolled in the HCS

Criteria	Kentucky	Massachusetts	New York	Ohio
**Number of communities**	16 counties	16 cities/towns	13 counties, 3 cites/towns	19 counties
**Additional criteria**	Selected counties that had (1) a syringe service program (marker of community readiness), (2) a jail, (3) ≥1 buprenorphine-waivered provider, and (4) ≥5 opioid	Selected to minimize proximity and contamination, favored communities with an anchor office-based addiction treatment (OBAT) program and a pre-existing	Selected the communities of Buffalo in Erie County, Rochester in Monroe County, and the Brookhaven township in Suffolk County to keep size comparable	Randomly selected counties stratified by urban/rural that (1) were not contiguous and (2) did not share an alcohol, drug

	overdose deaths in 2017	substance use coalition		and mental health board

5.2 Exclusion Criteria

Communities that did not meet the aforementioned inclusion criteria were excluded from the HCS. Massachusetts and New York did not have any further exclusion criteria. Kentucky excluded three counties because they are actively engaged in two National Institutes of Health–funded community-level interventions; inclusion would confound respective outcomes, and these communities could not be randomized. Ohio excluded 10 counties for lack of available opioid-related data.

5.3 Strategies for Recruitment and Retention

Each of the four RSs conducted its own recruitment process for engaging communities.

Kentucky identified possible counties based on the aforementioned inclusion criteria, then partnered with the Kentucky Agency for Substance Abuse Policy (KY-ASAP) and the local KY-ASAP boards located in the counties that met the inclusion criteria. The mission of the KY-ASAP and its local boards is to develop a long-term strategy designed to reduce the incidence of youth and adult smoking and tobacco addictions, promote resistance to smoking, reduce incidence of substance use disorders, and promote effective treatment of substance use disorders. Local KY-ASAP boards signed a letter of support indicating their willingness to participate.

Massachusetts identified towns/cities with high opioid mortality rates, then sought geographic diversity by convenience sampling, mindful of community proximity and its potential contamination. Communities were prioritized with an “anchor” community health center in which an OBAT program could be established or expanded, as well as communities with a pre-existing substance use coalition. An explicit expectation for inclusion was willingness to participate fully in the study as a Wave 1 or Wave 2 community. We excluded Boston and adjacent communities because many intervention components (e.g., OBAT, addiction consult service, bridge clinic) were already well developed in this metropolitan area.

New York identified counties with high opioid overdose death rates, then worked through the existing research team networks to reach out to the local health or mental health department of each county. Counties signed a letter of support indicating their willingness to participate.

Ohio identified highly affected communities in two steps. Step 1 involved a comprehensive environmental scan of ongoing interventions funded by federal, state, and local entities and community organizations, containing latest available data for the primary and secondary outcomes. In step 2, the final set of communities was identified via a sampling design that minimized spillover effects. All randomly selected communities committed to partnering in the HCS as demonstrated by letters from leadership from the local county opioid coalitions, including representation from health care, behavioral health, and justice settings.

6. STUDY INTERVENTION(S)

6.1 Study Intervention(s) Administration

This section describes in more detail the three components of the Communities That HEAL (CTH) intervention: (1) a community-engaged change process that forms the backbone of the HEALing Communities Study (HCS); (2) the Opioid-Overdose Reduction Continuum of Care Approach (ORCCA), a menu of strategies to implement evidence-based practices (EBPs) as a key component of the CTH intervention; and (3) community-based health communication campaigns designed to increase community engagement (CE), reduce stigma, and increase demand and utilization of EBPs. See Figure 2 for a depiction of the three components of the CTH intervention.

Figure 2: Three components of the CTH intervention

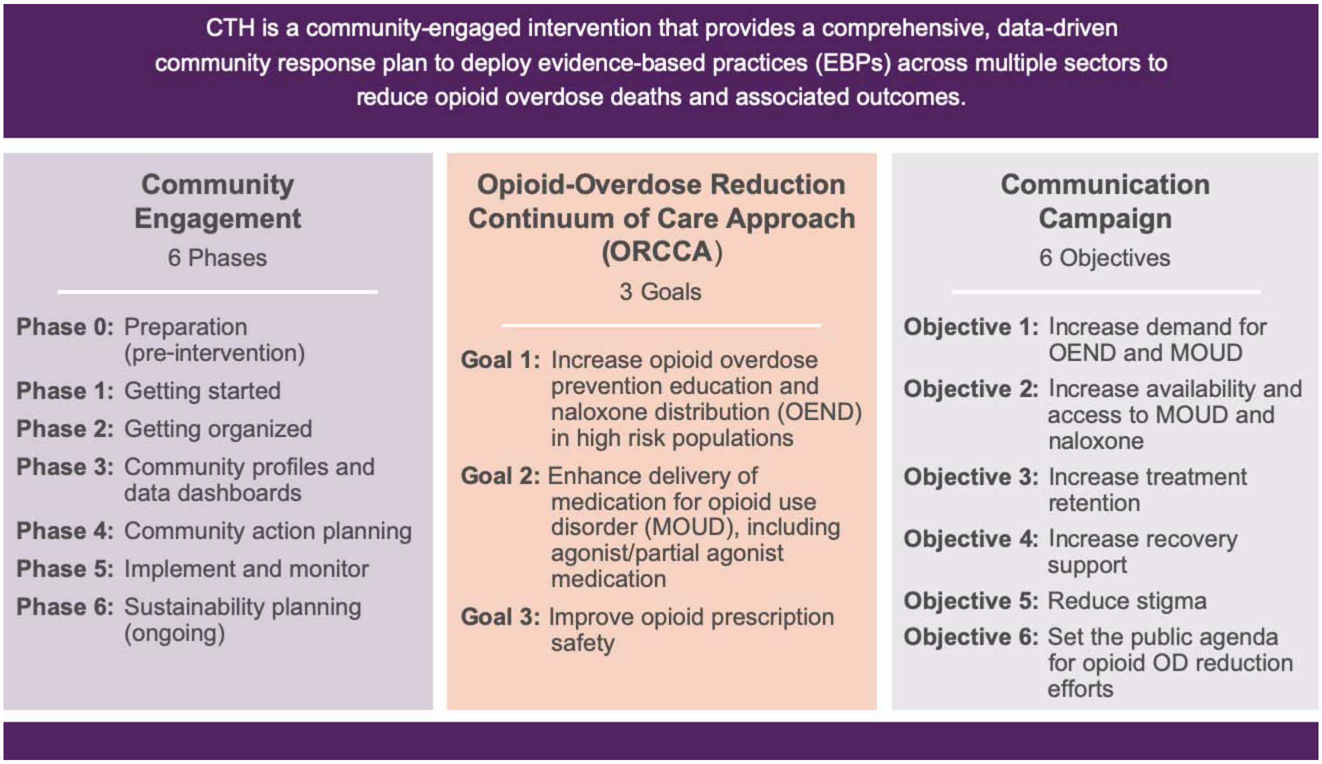

6.1.1 Study Intervention Description

6.1.1.1 Communities That HEAL Intervention

The HCS investigators hypothesize that the fastest and most sustainable way to achieve a relative 40% reduction in opioid-related overdose deaths is to support local community coalitions and their stakeholders in building and enhancing a comprehensive, data-driven community response to the opioid crisis in their community. The CTH intervention seeks to promote a common vision, shared goals, and tailored strategies to mobilize HCS communities to implement communication campaigns and adopt EBPs using a stepwise community change process. Drawing on community-based participatory research principles, the CTH intervention

will partner HCS researchers with multi-sector coalitions to develop a community-driven change process that will enable communities to be more effective in preventing deaths from opioids.

The conceptual framework of the CTH intervention is shown in Figure 3. Briefly, the CTH includes co-creation, or participatory, approaches that lead to coalition-driven community change, enhanced decision making with data dashboards, and the design and implementation of communication campaigns that focus on increasing demand for EBPs and reducing stigma. The implementation of EBPs in each community is guided by the ORCCA that prioritizes EBPs, populations, and venues most likely to reduce opioid overdose fatalities. The ORCCA approach is supported by data from implementation science research and will be a focus of our cost-effectiveness research. These intervention components are then hypothesized to lead to the primary outcomes described earlier.

Embedded in this work is the belief that the primary responsibility for practice change lies in the community. Further, it is recognized that communities are complex and that distinct priorities exist across and within them. Thus, community members, particularly those who are most affected, have a nuanced understanding of the best ways to implement and promote EBPs locally. This dynamic interplay of theories of change, EBPs, and the realities in each community will establish a data-driven learning system to facilitate a greater understanding of principles needed for community change.

Figure 3: Conceptual framework of the CTH intervention

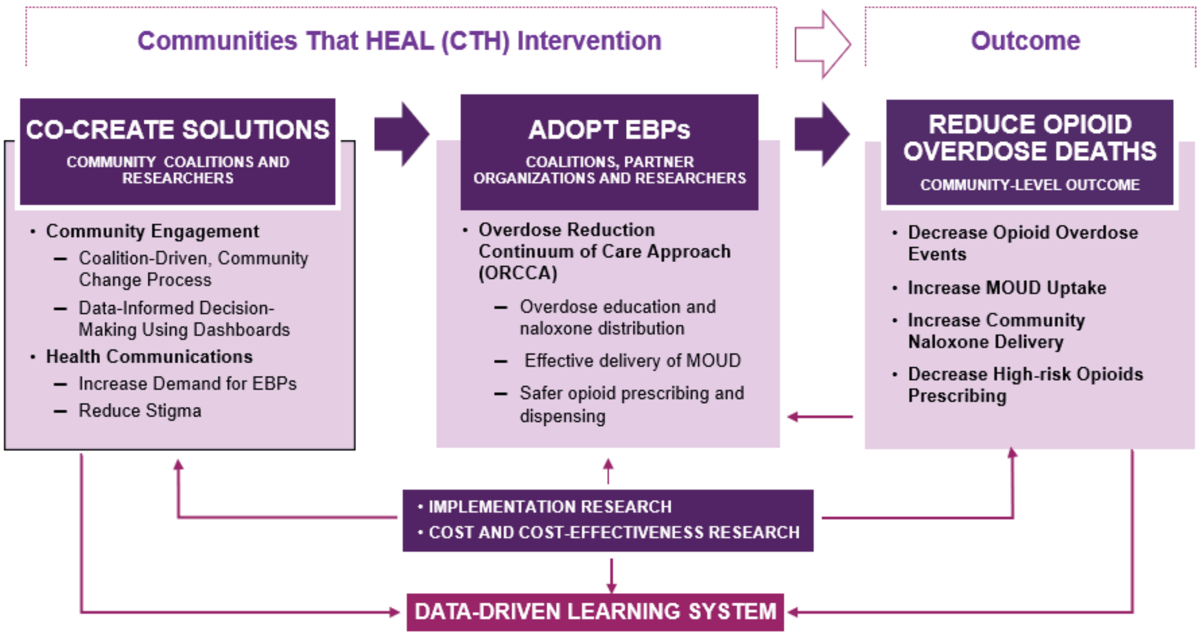

6.1.1.2 The Community Engagement Process

The CTH intervention is an adaptation of the Communities That Care model.[23-25] CTH is a stepwise but also iterative learning process for engaging communities in a partnership that

enables the adoption of EBPs chosen collaboratively to address the opioid crisis. The CTH is a non-linear, dynamic, and co-learning CE process that incorporates principles of systems and implementation science, health communications targeting stigma reduction and demand creation, and sustainability planning. The CTH CE process will involve seven phases, within which the ORCCA implementation and communication campaigns are operationalized, as shown in Figure 4a for Wave 1 and Figure 4b for Wave 2.

Figure 4a: CTH intervention phases (Wave 1)

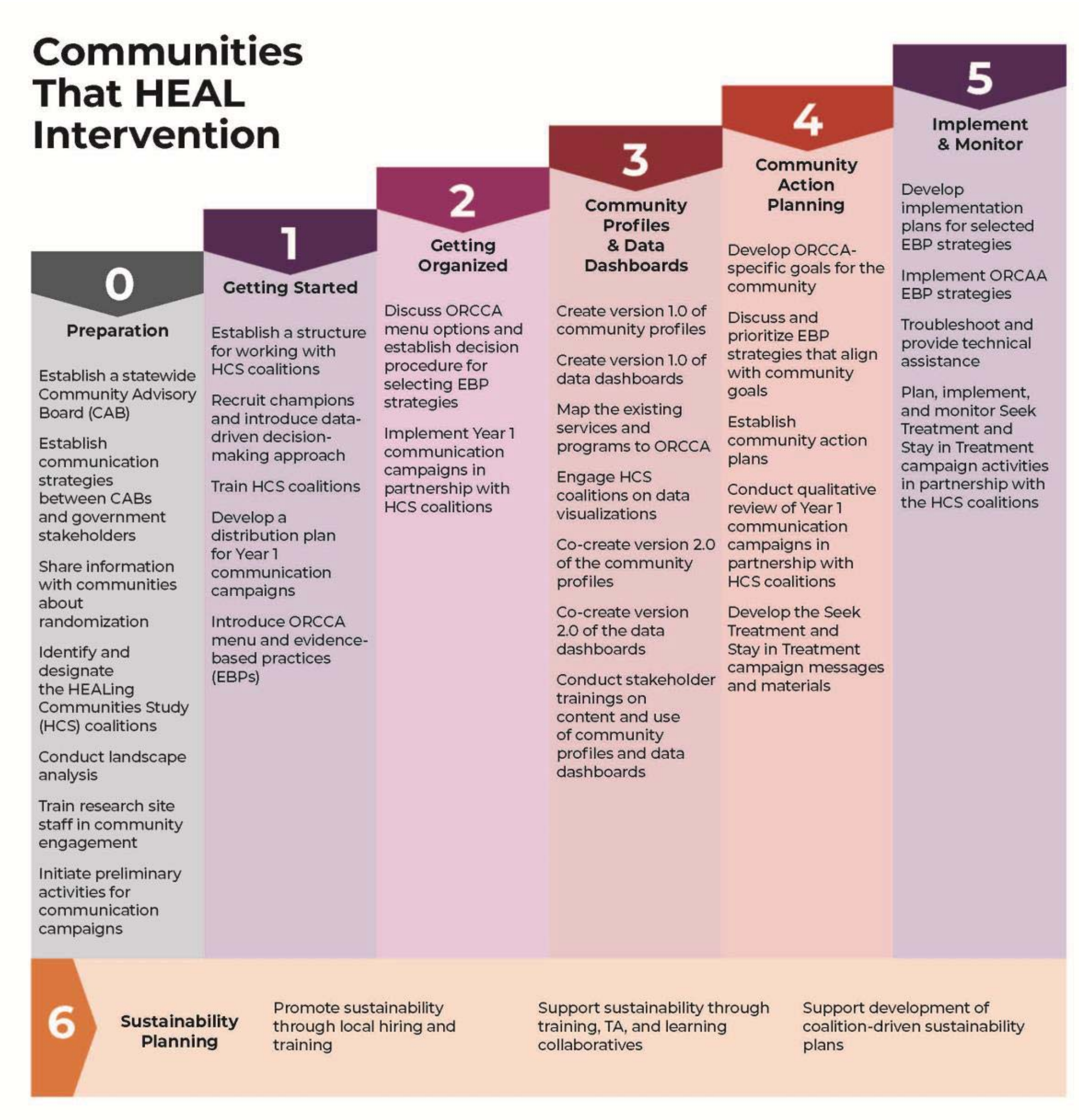

Figure 4b: CTH intervention phases (Wave 2)

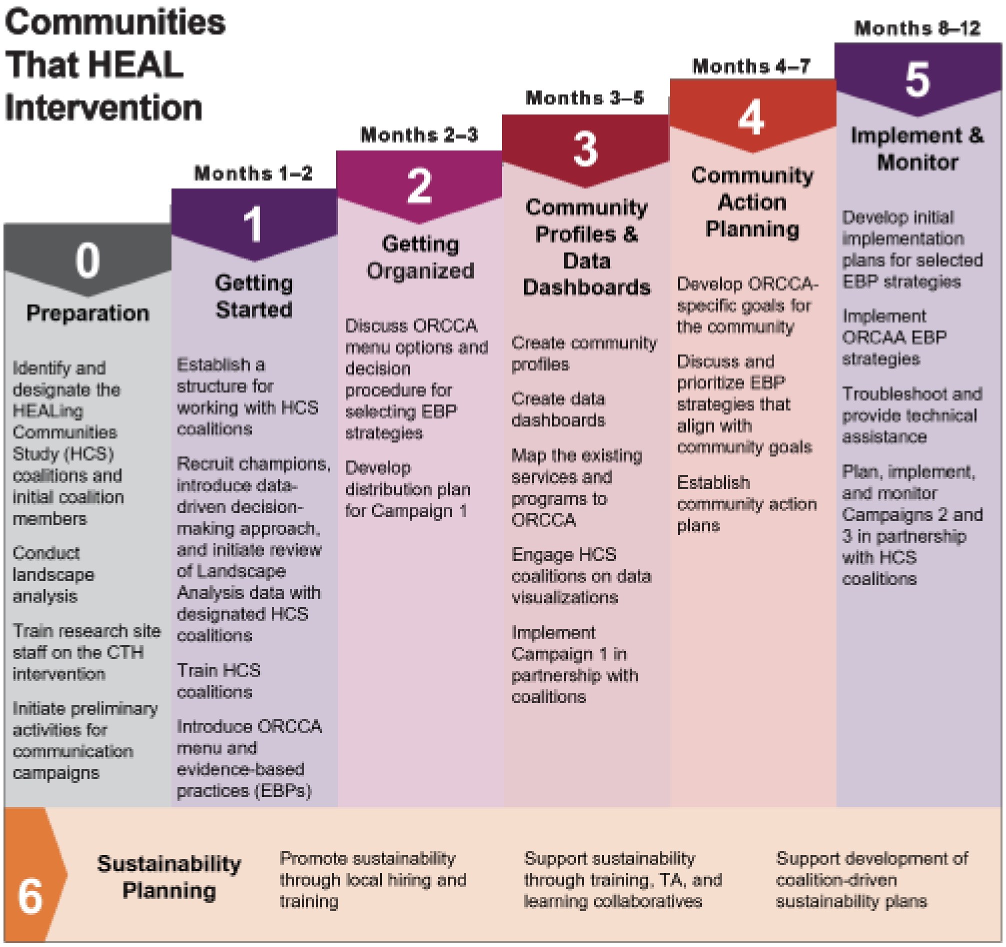

Phase 0: Preparation (Pre-Intervention)

This phase refers to the period of the study before the launch of the CTH intervention (as described in the ensuing phases 1–6). Tasks 1-3 below cover Wave 1 and Wave 2 communities and are completed at the start of the study.

1. Establish a statewide Community Advisory Board (CAB): CABs will serve as leadership bodies for community-based participatory research partnerships, ensuring that research activities are reflective of community priorities. As such, CAB composition typically reflects the communities of interest. CABs serve as a mechanism for community members to voice concerns and priorities that otherwise might not be on the researchers' agenda. The CAB may also advise on the research process to help ensure that methods and procedures are acceptable to community members and aligned with local norms and values.[26]

2. Establish communication strategies between CABs and government stakeholders: Possible options may include progress reports, sharing of CAB meeting minutes, and structured or unstructured opportunities for participation by federal officials in CAB meetings.

3. Share information with communities about randomization: This activity seeks to develop and implement a strategy to communicate effectively and sensitively about randomization in a way that is acceptable to Wave 1 and Wave 2 communities, state officials, and other stakeholders. In particular, we want to avoid dropout and maintain cooperation of Wave 2 communities.

4. Identify and designate the HCS coalitions: During this pre-intervention phase, HCS teams will identify existing coalitions in their communities to inform designation of HCS coalitions and to facilitate collaboration among local alliances and stakeholders doing CTH-relevant work. In some communities, HCS coalitions may need to be created or modified to ensure representation of diverse groups, including those with lived experience, as well as racial and ethnic minorities and other groups disproportionately burdened by the opioid crisis. In other communities, existing coalitions or other established community advisory groups will be designated as HCS coalitions. Additionally, select members from different existing community coalitions may be brought together to form the new HCS coalition. In Wave 2, work under this task may also involve RSs sharing published HCS protocol papers, sample coalition charters, and sample champion descriptions with community stakeholders, as part of providing an overview of the HCS.

5. Conduct Landscape Analysis (LA) and Baseline Assessments: An LA will be conducted to capture prevention, treatment, recovery support services, and infrastructure in health care, behavioral health, and criminal justice organizations, along with other key community features, such as local political context, that can be gathered from publicly available data sources. Specifically, the LA is designed to (1) describe the assets and gaps in the community that are relevant to HCS and CTH; (2) support collaboration with the coalitions as they make decisions about which EBPs are needed or could be enhanced in their communities and what venues may need to be engaged in the delivery of EBPs; and (3) help each HCS coalition and RS staff supporting the coalition to generate a list of potential key agencies and groups with which to partner for EBP delivery. Scientific team members will search publicly available, online and secondary data sources to identify relevant assets that exist in Wave 1 and Wave 2 communities. The LA report generated at the end of this exercise will provide key community context and directly inform CE efforts of the HCS. The LA for Wave 1 has two phases: **LA Phase 1**, which involves research staff searching publicly available information (primarily online) to identify assets in the communities; and **LA Phase 2**, which involves research teams contacting a subset of assets by email, by telephone, and/or in person to administer follow-up questions about the agencies' services. Also, in Phase 0, RSs will collect pre-intervention (baseline) data from coalition members, CABs, and key stakeholders and community service providers to gather their input on the opioid crisis in their communities, ongoing efforts to address the crisis, and community or coalition factors that may affect the implementation of EBPs in HCS communities.

   Based on lessons learned and efficiencies gained in Wave 1, the LA for Wave 2 communities consists of three steps. During Phase 0, two steps of the LA will occur either sequentially or concurrently. In step 1, RS staff will enter/update preliminary data in the REDCap tool from existing reports or data sources and from web searches in

the REDCap tool and identify & engage key community informants to elicit information on additional assets and gaps. In step 2, RS staff will refine, complement, and synthesize existing data by conducting systematic searches of publicly available data (e.g., using Google) and using this data to support interactive asset mapping. Organizational questionnaires, equivalent to the LA Phase 2 conducted in Wave 1 communities, may be conducted as an optional activity.

6. Train research site staff on the CTH intervention: RS staff will be trained on the CTH intervention, including principles of CE. Based on suggested competencies for implementation scientists doing community-engaged research, trainings will focus on skill development. Educational strategies may include in-person workshops, web-based didactics, and longitudinal debriefing sessions with CE staff.

7. Initiate preliminary activities for communication campaigns: This activity aims to lay the foundations for a health communication campaign (third component of the CTH). During this process, HCS teams will develop campaign playbooks and messages, conduct interviews with media gatekeepers, and perform message testing. Wave 2 communities will tailor campaign materials developed with Wave 1 communities.

Phase 1: Getting Started

This phase commences the CTH intervention. According to Figure 1, Wave 1 communities will begin Phase 1 in January 2020, and Wave 2 communities will begin Phase 1 in July 2022.

Stakeholder identification and engagement is an iterative process of relationship development with community stakeholders to foster effective collaboration to support HCS aims. This includes (1) partnering with and strengthening local coalitions to provide leadership and contextualize the opioid crisis for the EBPs; and (2) collaborating with state and local governments to strengthen the policy environment, expand resources, and support communities' capacity to accelerate and expand delivery of EBPs to prevent opioid overdose deaths.

In Wave 1, coalitions developed a distribution plan for the Year 1 communication campaign during Phase 1. However, this task was moved to Phase 2 for Wave 2 to allow more time for building communities' capacity for community campaign work.

1. Establish a structure for working with HCS coalitions: Each community participating in the HCS will develop or engage a standing body of stakeholders to support this study, referred to as the HCS coalitions. Coalitions are the primary locus for CE in this study. In some communities, pre-existing local coalitions established through state policy will be designated HCS coalitions. In other communities, new coalitions will be formed. Given the anticipated variation in HCS coalitions' structures and practices, RS staff will need to work with coalitions to determine and document feasible protocols and procedures for partnering on CTH. Documenting protocols and procedures can help bolster RS–coalition partnerships and promote efficient implementation of the CTH intervention. Another key tool for bolstering the RS–coalition partnership through effective communication is the CTH portal, an online platform RSs will develop and customize for each HCS community. In Wave 2, HCS researchers agreed that RSs will work with coalitions to determine whether members, champions, or chairs should be reimbursed for their time, travel, or other resources committed to the CTH intervention.

Reimbursement is a community-driven decision, so may vary across sites. In Wave 2, HCS researchers also decided to include explicit guidance in the CTH implementation manual around meaningfully engaging people who use drugs or are in recovery. People with lived or living experience may be engaged through membership in the coalition, as champions, or through one-on-one consultation. The approach to engaging people who use drugs or are in recovery may vary across HCS communities, but HCS researchers have committed to the following principles:

- Respect the engagement preferences of people who use drugs, including ensuring anonymity, if requested
- Protect the safety of people who use drugs and address any concerns related to criminalization
- Address any barriers to participation, such as limited transportation, mobility, schedule, or internet access
- Aim for diverse representation of people who use drugs (e.g., age, gender, race and ethnicity, and sexual orientation diversity)
- Include protocols for addressing any stigma or related conflict within the coalition
- Comply with sIRB requirements

2. Recruit champions introduce data-driven decision-making approach, and initiate review of community assets data from the Landscape Analysis: The objective of this activity is to identify coalition members who can facilitate communication and activities between the coalitions, the HCS research team, and partner organizations. This process involves orienting coalitions to data-driven decision-making approaches and identifying and engaging multi-sector coalition members who are willing and able to serve in leadership roles.[27] For Wave 2 communities, the third step of the Landscape Analysis consists of inviting coalition members to review data collected from the Landscape Analysis.

3. Train HCS coalitions: It is important to ensure that designated coalition members have the necessary background knowledge and skills to carry out their roles and fully participate in meetings. Specialized training modules for designated coalition members will ensure that they have a foundation of information that will allow them to participate meaningfully in discussions and decisions. These modules serve as a prelude to more in-depth discussions of surveillance, treatment, implementation in practice settings, and evaluation in subsequent steps.

   The development of an HCS-related coalition training plan is critical to facilitating coalition-led HCS activities. Coalition members should be provided with an overview of the HCS. This may include grant materials describing the HCS design and the overall leadership structure including the role of the CAB. The orientation should also include a detailed overview of the CTH process and planned menu items associated with the ORCCA menu of EBPs. Additionally, community stakeholders should understand the rationale underlying study activities, including constraints dictated by research requirements. It should be made clear how action-oriented implementation research

differs from routine service delivery on the one hand and traditional clinical research on the other. To be most effective in their roles, members of coalitions must be able to understand and challenge data that are presented, to inform their decisions and strategies.

4. Introduce the ORCCA menu and EBPs: In this phase, community coalitions begin to convene and engage in the HCS. The HCS team will introduce the ORCCA-required objectives and the menu of EBP strategies. Community champions, one for each of the three required objectives, would have been identified and recruited. In the context of the community-specific LA, they will begin developing a shared understanding of the local epidemic, current services to address overdose, and relevant settings.

Phase 2: Getting Organized

In Phase 2, community coalitions and HCS teams will review and discuss the menu of EBP strategies and the strategy selection process. Through review and discussion of the EBP strategies, coalitions will begin to develop a shared vision for implementing the EBPs, which will facilitate data review and action planning in subsequent CTH phases. During Phase 2, HCS teams will also develop a distribution plan for Campaign 1 in partnership with the coalitions.

1. Discuss ORCCA menu options and decision procedure for selecting EBP strategies: The purpose of this activity is to ensure that designated coalition members understand the rationale for emphasizing EBPs, the range of differences among ORCCA menu options, and how to evaluate anticipated risks and benefits of different approaches with different populations in different settings. Coalitions will review options and make recommendations to guide partner organizations' decisions about selection of options. Deliberations about choices should address appropriateness, preferences, and feasibility, as well as the ability to monitor implementation and fidelity. The process here will focus on coalitions' identification and selection of EBPs that best suit their community needs.

2. Develop a distribution plan for Campaign 1: RS staff will collaborate with community coordinators, CAB members, program managers, community engagement facilitators, communication champions, subcommittee or coalition members, and partner organizations to develop a plan for disseminating materials for Campaign 1.

Phase 3: Community Profiles and Data Dashboards

This phase will focus on the collaborative improvement or development of data systems and surveillance infrastructure to allow systems integration and inform decision making. Surveillance will support establishment of community-facing dashboards that will provide multi-level data necessary to support action planning and improvement in practice and outcomes. This phase will involve sequential and parallel processes as depicted in Figure 5 and described below for Wave 1.

1. Create version 1.0 of the community profile: The purpose of this activity is to collaborate with state and local stakeholders to collect or review data that describe the

state of the opioid epidemic and treatment and opioid overdose prevention resources across sectors of participating communities. Data for the community profiles will include existing assessments completed by the community, existing or updated LAs, and new data collection to supplement existing profiles or create new profiles. Also, in this phase, the research teams will present the results of prior LAs conducted in Phase 0 for their feedback to solicit input on missing elements and make corrections in the data. Data to be collected may vary across sites but could include epidemiological data on the epidemic, contextual information on county conditions, assessment of stigma in the county, network analysis of organizations, and an inventory of providers and services across the continuum of care and in multiple settings.

2. Create version 1.0 of the data dashboard: Access to integrated data from different sectors can improve communities' capacity to plan, monitor, innovate, respond, and support community health improvement. At this step, we will facilitate discussion in the CAB, coalitions, or across organizations to define what data sources are important to profile the crisis in their community and develop a system for sharing and displaying data that meet community needs. To develop and/or improve data sharing relationships across community organizations, study teams will facilitate discussions about data sharing, including identifying key stakeholders and community partners that collect and use data, identifying enablers or barriers to using and sharing data, and developing a shared understanding of the potential benefits of data sharing and data visualization. Once this shared understanding is developed, researchers will assist in assessing the types of data they collectively like to share and will assist in developing a data visualization tool or dashboard that meets identified community needs.

3. Map the existing services and programs to ORCCA: Working together, the community coalitions and HCS teams will review the community's inventory of existing resources, identify gaps in services for people at high risk for opioid overdose, and identify barriers to addressing service gaps. This process will be completed for all currently available services that address the three ORCCA-required EBPs.

4. Engage HCS coalitions on data visualizations: In this phase, HCS researchers will engage coalitions and other key stakeholders on the visualization of the community profiles and the use of dashboards.

5. Co-create version 2.0 of the community profile: RS staff and coalitions collaborate to develop version 2.0 of the community profile. Version 2.0 of the community profile is a written document that demonstrates shared understanding of the existing ORCCA services, who is currently engaged, and who can and should be engaged in implementing EBPs. The development process for this final document will likely include iterative drafts of a written profile that is shared with the community coalition for feedback and final approval.

6. Co-create version 2.0 of the data dashboard: RS staff will collaborate with coalitions to develop version 2.0 of the community-tailored dashboard. This task builds on discussions from version 1.0, where coalition members and stakeholders discuss community-specific data issues and recommendations. Co-creating version 2.0 of dashboard involves ensuring that version 2.0 dashboards are responsive to the

community needs identified by coalitions, and this may require adding new metrics or visualizations to the dashboards.

7. <u>Conduct Stakeholder Trainings on Content and Use of Community Profiles and Data Dashboards:</u> After community profiles are developed, coalitions will be offered training and support to work with and understand data, in order to consider this information in setting goals and action plans. The purpose of this activity is to ensure that designated coalition members understand how to interpret and work with community profile data.

Figure 5: Phase 3 parallel processes for developing community profiles and data dashboards (Wave 1)

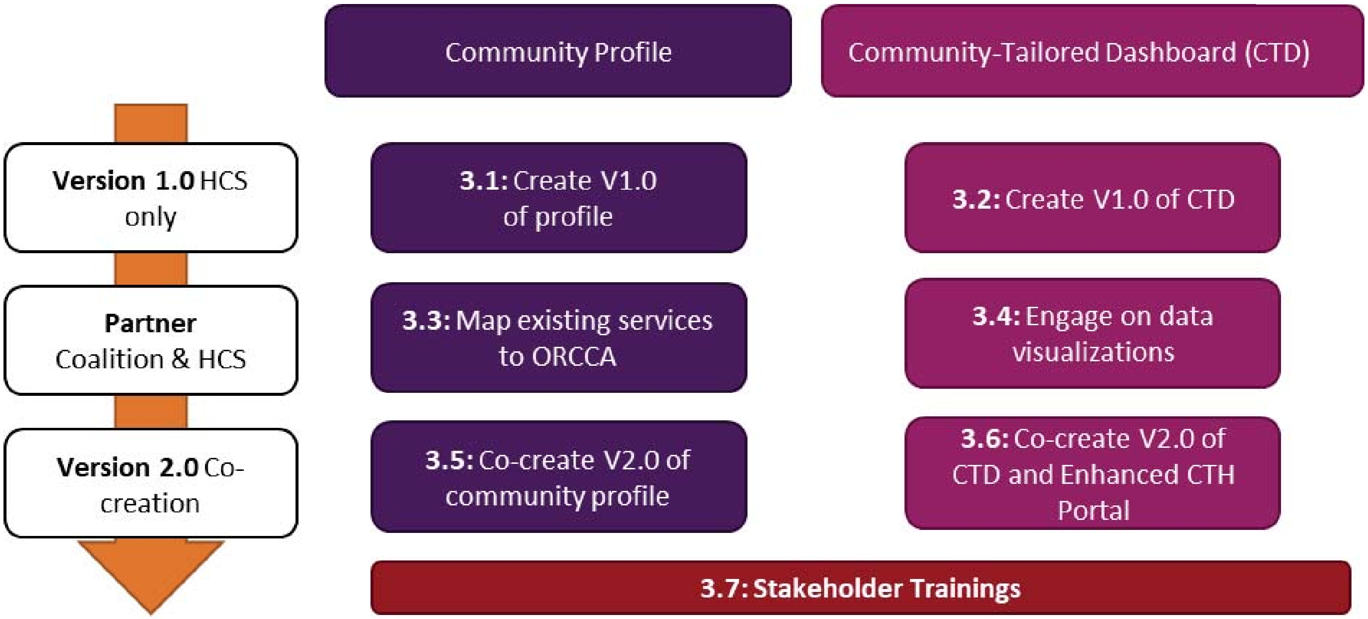

For Wave 2, research sites will draw on lessons from the development of Wave 1 community profiles and dashboards, and only one version of the profile and dashboards will be developed for Wave 2 communities. Additionally, the training conceptualized as a separate task in Wave 1 (task 7 above) has been incorporated into the ”Engage HCS coalitions on data visualizations” task (4 above), as research sites experienced that engagement on visualizations and training often went hand-in-hand during Wave 1. In Wave 2, the implementation of Campaign 1 will occur in Phase 3.

Phase 4: Community Action Planning

In collaboration with stakeholders and coalitions, we will use community profiles and data dashboards to identify key gaps and areas of unmet need evident in the data. Planning will entail a data-driven facilitation process during which EBPs are discussed, considered, and chosen (as applicable). Further, planning will detail how EBPs will be implemented and how progress will be monitored and reported to others and by whom. These plans will draw on the best available scientific evidence and stakeholder experience regarding individual, organizational, and community change. Leaders of organizations involved in implementing the designated EBPs will be encouraged to be fully engaged in this process.

1. Develop ORCCA-specific goals for the community: Coalitions will drive goal setting with support from RS staff. Community ORCCA-specific goals must align with the HCS goal to reduce opioid overdose mortality. Coalitions can also consider whether it is beneficial to adapt goals from previous plans or related efforts to address the opioid crisis. HCS coalitions can select goals using ORCCA-specific resources.

2. Discuss and prioritize EBP strategies that align with community goals: Coalitions will work with RS staff to discuss and prioritize EBP strategies that (1) align with the ORCCA-specific goals developed under the previous task, (2) address the gaps identified through review of the community profile and data presented on dashboards, and (3) are high impact and highly feasible. To the extent possible, coalitions should engage potential partner organizations in this selection process. Potential partner organizations can help assess the feasibility of implementing the EBP strategies under consideration. In some cases, these organizations will be members of the coalition. However, if potential partner organizations are not also coalition members, they can be invited to participate in strategy setting meetings or invited to provide timely input via email or separate meetings.

3. Establish community action plans: The format of action plans may vary across RSs and communities, but, at a minimum, action plans across all sites will capture the prioritized EBP strategies under each of the three required ORCCA components (OEND, MOUD, and prescription opioid safety). Recommended steps for completing this task include reviewing the priorities set in the previous task with the coalition, then drafting action plans and identifying high-priority strategies to move forward for development of implementation plans. When possible, coalitions and research teams should engage partner organizations in the action planning process. Of note, as communities move through Phase 5, action plans may need to be revisited and revised in response to challenges encountered and lessons learned during the implementation process or evolving community needs and resources. Starting in Phase 4, RSs will complete the ORCCA Tracker (ORCCAT) monthly for each community to document the selection of EBPs and any changes to action plans over time. As communities progress to Phase 5, the ORCCAT will also be used to track EBP implementation. See Table 10 for additional instrument details.

In Wave 1, this phase included two communication campaign tasks (4 and 5 below). However, as noted above, the cadence for the planning, implementation, and monitoring of communication campaigns was refined for Wave 2 based on lessons from Wave 1 and in response to the shorter timeline Wave 2 communities have for implementing the CTH. Tasks 4 and 5 below are not included in Phase 4 for Wave 2, rather a combined task to plan, implement, and monitor Campaigns 2 and 3 was added to Phase 5 for Wave 2.

4. Conduct qualitative review of Year 1 communication campaigns in partnership with the HCS coalition: Coalitions will review their dissemination activities to date for each of the three Year 1 communication campaigns (1- Naloxone, 2- Stigma, and 3- MOUD) with RS staff. As part of this review, RS staff will guide coalitions in a qualitative assessment to discuss what is working, what can be improved, and what additional resources can be leveraged to improve the reach, frequency, and effectiveness of Year 2 campaign activities.

5. Develop the Stay in Treatment Campaign Messages and Materials: RS staff and coalitions will apply the Prepare-Plan-Implement steps in Figure 28 to develop additional messages and materials for the Stay in Treatment campaign. Completing the Prepare-Plan-Implement steps involves RS staff working with coalitions to answer essential questions at each step in the process, including questions about the resources needed for success.

Phase 5: Implement and Monitor

This phase involves developing implementation plans for selected EBP strategies, implementing EBP strategies, and planning, implementing, and monitoring Campaigns 2 and 3 in partnership with HCS coalitions (Wave 2).

1. Develop initial implementation plans for selected EBP strategies: This task involves building on the action plans developed under Phase 4 to create plans that will detail how partner organizations will execute the specific EBP strategies they agree to implement, with support from RS staff and coalitions.

2. Implement ORCCA EBP strategies: Using implementation plans as a guide, partner organizations will implement selected EBP strategies with support from RS staff and HCS coalitions. As partner organizations monitor and learn from implementation efforts, they may identify opportunities to improve the implementation of EBP strategies and can work with RS staff and coalitions to modify implementation plans as needed.

3. Troubleshoot and provide technical assistance (TA): RS staff supporting EBP implementation will troubleshoot implementation problems with partner organizations and provide TA as needed to support optimal implementation of EBPs. Coalition members may also assist with troubleshooting and TA. Learning collaboratives (LCs) or communities of practice may be launched under this task to help address coalitions' and partner organizations' training and TA needs and to provide a forum for sharing successful strategies and lessons learned from EBP implementation.

4. Plan, implement and monitor Stay in Treatment campaign activities and a "Community Choice' campaign (a refresh and repeat of one of the previous four campaigns) in partnership with HCS coalitions (Wave 1): After completing the Prepare step in Phase 4 that concludes with the draft campaign plan, RS staff will partner with coalitions to plan, implement, and monitor Year 2 activities for: (1) Stay in Treatment and (2) Community Choice. Stay in Treatment materials will be distributed in communities starting in June 2021. Community Choice campaign materials will be distributed starting no later than January 2022.This Wave 1 task was changed to plan, implement, and monitor Campaigns 2 and 3 in partnership with HCS coalitions for Wave 2.

Phase 6: Sustainability Planning

Training and data-driven decision making are key CTH elements that help position coalitions and partner organizations to sustain EBP implementation after HCS ends. Phase 6 involves setting up training and data tools and resources used in previous phases to be available to coalitions over the course of the intervention period and beyond. RSs will also assist coalitions and partner organizations with developing a sustainability plan.

1. Promote sustainability through local hiring and training: Over the course of the intervention, RSs will recruit and train local community members for key CTH roles (see Phases 0, 1, and 2). Hiring and training community members to implement the CTH intervention helps build local expertise related to data-driven action planning and EBP selection, which can support long-term EBP implementation that in turn leads to improved health outcomes.

2. Support sustainability through training, TA, and learning collaboratives: RSs provide training and TA for HCS communities over the course of the intervention to support data-driven planning, EBP implementation, and communication campaigns. As communities make progress on these key CTH components, the focus of training and TA shifts to sustainability. Areas of focus for sustainability TTA may include obtaining financial resources to continue CTH efforts beyond HCS, maintaining the data infrastructure (e.g., data dashboards and data sharing protocols) to continue data-driven planning, and leveraging HCS communication campaign strategies and materials for dissemination beyond HCS.

3. Support development of coalition-driven sustainability plans: A coalition-driven sustainability plan is required for all HCS communities. RSs will assist coalitions and partner organizations with developing a sustainability plan. Sustainability planning is a community-driven process; therefore, planning activities and plans are expected to vary within and across communities and RSs. RSs will encourage the adoption of sustainability planning best practices, including developing plans that specify measurable sustainability goals for core CTH components—community-engaged, data-driven action planning; EBP implementation; and a communication strategy to support the uptake of EBPs—and markers of success.

6.1.1.3 Maintaining CTH Beyond Year 1 (Wave 1)

Wave 1 communities are expected to maintain and deepen the core elements of the CTH—community engagement, ORCCA, and health communications—in an integrated manner throughout the intervention period, which ends June 30, 2022. RSs, coalitions, and partner organizations will continue collaborating to maintain and expand EBP implementation and other elements listed in Figure 6. As needed, RSs may support coalitions in cycling back to any CTH phases to address emerging community concerns related to the opioid crisis. Note that this work to maintain CTH beyond Year 1 only applies to Wave 1 communities. Wave 2 communities will be implementing CTH on a truncated timeline and therefore are not required to complete these maintenance activities.

Figure 6: Schedule of Activities for Maintaining the CTH Beyond Year 1 (Wave 1)

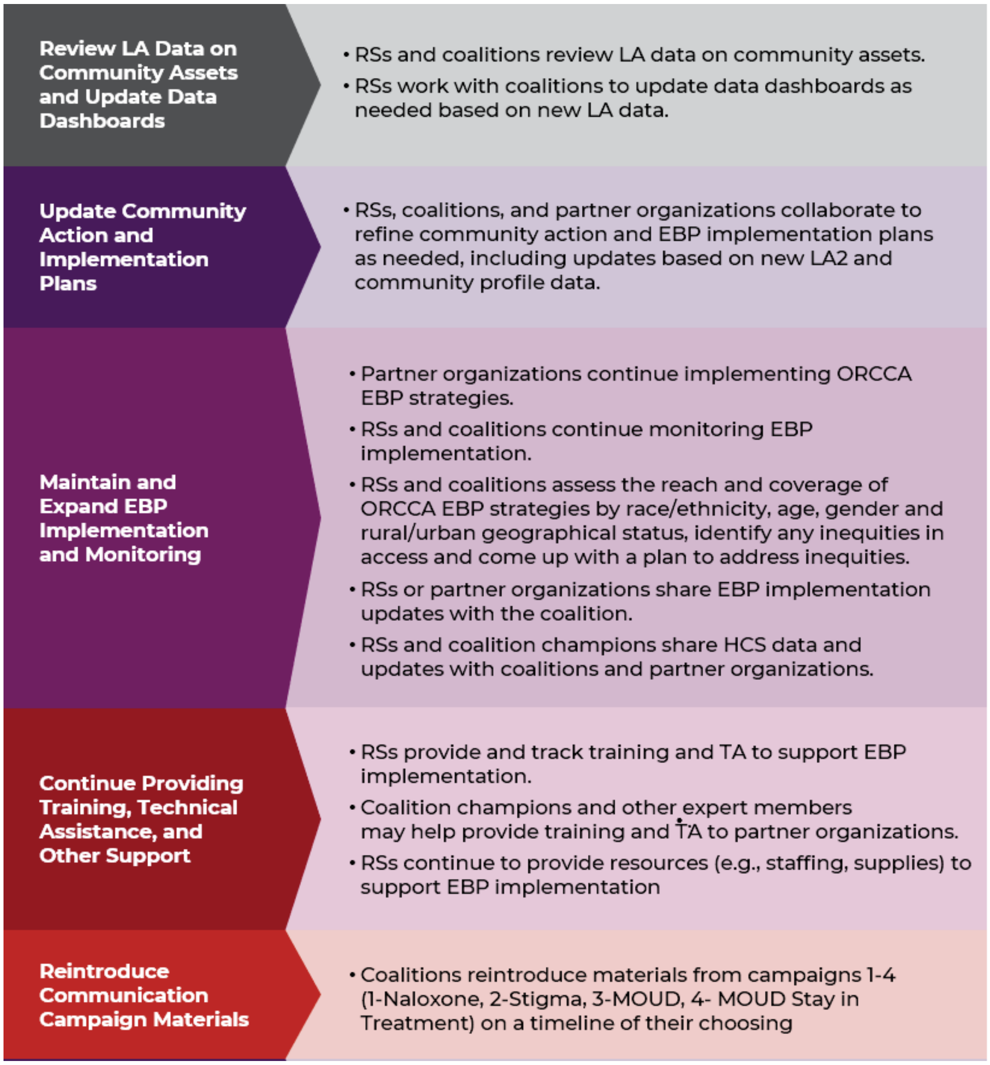

6.1.1.4 The Opioid-Overdose Reduction Continuum of Care Approach (ORCCA)

The ORCCA prioritizes practices, populations, and venues most likely to reduce opioid-related overdose fatalities (see Figure 7) and can be mapped to the OUD cascade of care model.[28] This model describes the stepwise progression from initial opioid exposure to a diagnosis of OUD, initiation, engagement, and retention in treatment. The ORCCA provides strategies to reduce overdose deaths along this OUD cascade of care.

Figure 7: The HCS ORCCA with sample strategies

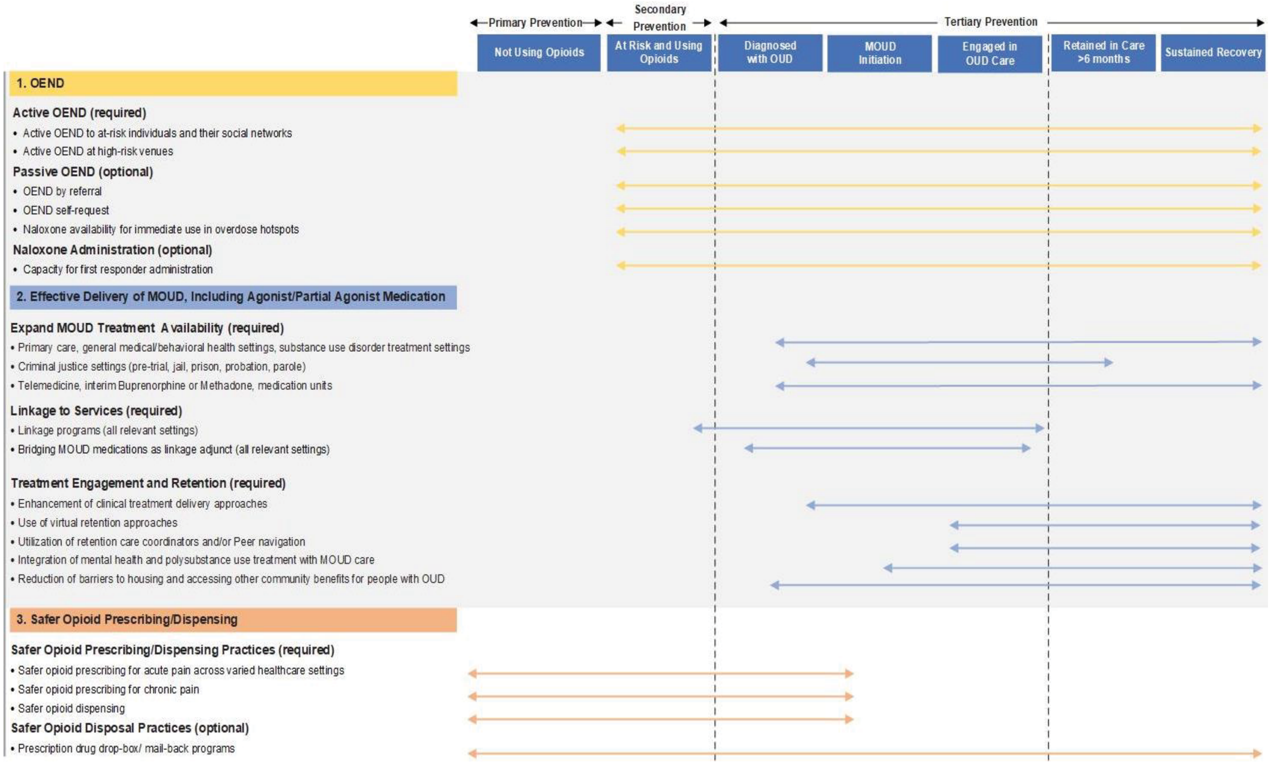

Source: Figure and conceptual model adapted from Williams and colleagues.[28]

ORCCA Requirements

The ORCCA menu of strategies will evolve over time based on emergent community needs as new evidence and resources are collected. Through the CTH intervention, each coalition will use the ORCCA menu of strategies to facilitate community adaptation and implementation of the three required EBPs.

ORCCA EBP Requirement 1: OEND in high-risk populations

Opioid overdose victims die when they do not receive an antidote in time. In many cases, naloxone can completely reverse opioid overdoses. To implement ORCCA EBP Requirement 1, community action plans will need to include strategies that actively distribute naloxone and provide overdose prevention education to high-risk individuals and their social networks at high-risk venues. Optional strategies that can be included in the community action plan are OEND by referral, OEND by self-request, naloxone availability for immediate use in overdose hotspots, and capacity for first responder administration.

ORCCA EBP Requirement 2: Effective delivery of MOUD, including agonist/partial agonist medication and outreach and delivery to high-risk populations

Reducing opioid overdose fatalities and improving secondary study outcomes will require increasing the availability of MOUD.[6] Improved access to evidence-based MOUD treatment, particularly agonist/partial agonist treatment, can significantly reduce the risk of overdose death.[11] The most effective treatment for OUD is MOUD, including methadone maintenance, buprenorphine maintenance, and naltrexone. MOUD significantly reduces the likelihood of opioid overdose,[29-32] human immunodeficiency virus transmission,[33,34] and hepatitis C virus transmission.[33,35,36] MOUD is also associated with increased employment rates,[37] increased quality of life,[38] decreased crime, and decreased utilization of high-cost health care services.[39]

Expanding MOUD treatment availability

In the absence of evidence that MOUD treatment is readily available on demand and with few or no barriers (i.e., multiple settings with no waitlists for MOUD, adequately covered by insurance), efforts to expand MOUD treatment (i.e., capacity building) are required as part of the CTH intervention. These efforts could include making MOUD treatment available in settings currently lacking MOUD, such as criminal justice, general medical, and addiction treatment settings, and expanding the capacity of settings that already offer MOUD to treat more individuals.

Linking to MOUD services

Techniques to target individuals at heightened risk and link them to services are required. In particular, providing MOUD treatment (e.g., buprenorphine) on a short-term basis outside addiction treatment settings is an effective adjunct to linkage-to-care practices that significantly increase subsequent engagement in structured and sustained MOUD treatment programs.[40,41]

Enhancing MOUD treatment retention

Although MOUD is effective for those engaged in treatment, retention rates in MOUD are disappointing.[42-45] Thus, strategies to increase treatment engagement and retention are required as part of the CTH and are shown in Figure 7.

ORCCA EBP Requirement 3: Safer opioid prescribing and dispensing

Pharmaceutical opioid supply is a key source of opioid exposure, contributing to OUD and opioid overdose. Specific prescribing practices, including excessive prescribing for acute or postoperative pain, prescribing ≥90 morphine milligram equivalents per day for chronic pain, or co-prescribing opioids and benzodiazepines, increase the risk of opioid overdose. Promoting safer, more judicious opioid prescribing, dispensing, storage, and disposal practices can increase opioid safety, reduce the excess opioid supply in communities, and decrease the risk of overdose.

Identifying and engaging high-risk populations

As noted in Required EBPs 1 and 2, identification of and intervention with high-risk populations is an ORCCA requirement.

- *Definition of populations at substantially heightened risk for opioid overdose death:* Any individual with OUD is at risk for opioid overdose death, particularly if he or she is not engaged in MOUD. Characteristics that further elevate risk of overdose and death in individuals who use opioids include use of opioids and (1) a prior opioid overdose,[1,2] (2) reduced opioid tolerance[1,3-5] (e.g., completing detox or release from an institutional setting such as jail, residential treatment, or hospital), (3) use of other substances[6] (e.g., alcohol, benzodiazepines, cocaine, and amphetamine-like substances), (4) concomitant major mental illness[1] (e.g., major depression, bipolar disorder, schizophrenia, anxiety disorders), (5) concomitant major medical illness[1] (e.g., cirrhosis, chronic renal insufficiency, chronic obstructive pulmonary disease, asthma, sleep apnea, congestive heart failure; infections related to drug use), and/or (6) injection of drugs.[1,7]
- *Venues with populations at heightened risk for opioid overdose death:* These venues include (1) criminal justice settings[8,9] (e.g., pre-trial, jails, probation, parole, drug and problem-solving courts, police and narcotics task forces, halfway houses and community-based correctional facilities, departments of youth services); (2) syringe service programs,[10] (3) health care facilities[11-14] (e.g., emergency departments; safety net clinics; health departments, pharmacies, and hospitals), (4) first responder stations[15] (e.g., police and fire stations), (5) addiction treatment and recovery facilities, (6) mental/behavioral health treatment facilities, (7) community-based social service agencies (e.g., homeless shelters or other temporary housing, services agencies for transactional sex workers, halfway houses and/or other sober living facilities), and (8) hotlines (telephone or Internet) responding to service requests.

Process of EBP Strategy Selection

The general process for coalitions to review and select specific EBP implementation strategies was described in the CE phases. The process for determining what system and practice changes will be pursued can be divided into two levels: (1) the overall community level (stakeholder coalitions, etc.) and (2) the care delivery system level (e.g., system administrators and service providers), detailed as follows.

Community Level

Community action planning efforts will involve a bi-directional and iterative exchange in which community needs and capacity are matched to evidence about which approaches are available to rapidly affect opioid overdose mortality. Using decision aids and a shared decision-making process can facilitate this approach.[46,47] The goal of this exchange is to efficiently identify the most promising approaches, settings, and populations for a given community and to create a rationale from the community-wide perspective that can be helpful in motivating individual systems, administrators, and providers to implement the EBPs.

Care Delivery System Level

Ideally, the majority of interaction at the system administrator and service provider level will involve facilitation and operationalization of selected EBP strategies. This will be more likely if (1) setting-specific administrator and provider perspectives are adequately represented in coalitions, and (2) there is collaboration between service settings and the communities they serve.

Nonetheless, there is the potential for disconnect in which the coalition selects EBP strategies that are not feasible or acceptable to the administrators or providers of a given service setting. If this disconnect were to occur, it would entail three complementary responses. The first level of response would be to reiterate the coalition’s rationale and EBP implementation selection process with individual administrators and providers. The second level of response would be to explore the barriers within that setting or service to determine whether there is a possible solution. In many cases, overall concern about a change in practice is specific to components that, once defined, can be addressed to mutual satisfaction. The third level of response would be returning to the coalition with the administrator or provider concerns to refine the community action plan, search for solutions, or define alternate EBP implementation strategies.

Expected Changes in Systems and Practices

Several factors mediate the relationship between the CTH intervention and the expected changes in systems and practices that will be facilitated and measured as part of the study protocol.

1. Decisions and actions to implement system and practice changes as well as the actual services provided at the individual level are a clinical (non-experimental) matter that is not explicitly determined by the research protocol, so long as the implemented EBPs are aligned with the requirement and priority populations and settings. Although the CTH will work to facilitate the communities’ focus on effective practices and high-fidelity implementation that are evidence based, there is uncertainty as to whether this

facilitation will be successful. It is possible that communities will adapt existing EBP strategies, embrace approaches that were not initially selected for the list of EBP strategies, or even withdraw from the study altogether. Nonetheless, at no point will study resources be allocated to the testing of novel practices that are deemed experimental.

2. The premise of the CTH intervention is that with facilitation and technical support, it is possible for communities to modify existing system structure and practice to reduce opioid overdose deaths. This approach will necessarily involve helping coalitions devise ways to do more with existing resources and pursue additional resources. However, although direct service support by study funds (paying for treatment or other service delivery personnel, purchasing medications or supplies, etc.) is not inherently sustainable beyond the duration of the study, such service support is allowed.

3. Although we anticipate that there may be RS-to-RS and community-to-community variability in the system by which CTH facilitation is delivered, there will be a component of central, common, and more content-based leadership that is extended throughout the communities by a complementary component that is more local, more clinically experienced, and more focused on operations and TA. Although content expertise of centrally located academic experts is essential, effective facilitation at the level of individual settings and EBPs requires an intimate and sustained understanding of the personnel, practices, and systems in each EBP location. This technical and operational insight is most reliably found within individuals who have relevant practical and clinical experience for the setting and/or EBP. More importantly, these individuals are most likely to be viewed as credible by local administrators and practitioners.

Community-Based Communication Campaigns

The CTH intervention includes a series of communication campaigns that build on the empirical foundations of health communication and mass communication for behavior change.[48-51] The development and implementation of these campaigns have the following objectives:

1. Enhancing the adoption of EBPs in each community and heightening CE;
2. Developing a cohesive set of communication objectives, priority groups, strategies, and tactics that can be applied across all communities in the HCS;
3. Providing message materials for coalitions that can be tested and tailored to reflect the unique assets and characteristics of each community; and
4. Integrating approaches to reduce stigma.

Integrating health communication EBPs, social-behavioral theories, and insights from people we seek to serve, the campaigns described here are a common set of activities, using a campaign guidebook and messages developed and vetted by the RSs, that will be implemented in each of the Wave 1 communities. The campaigns are designed to use only locally accessible media resources to avoid potential spillover effects into Wave 2 communities before their activation. The structure of the campaigns is designed to provide a minimum protocol for communities to follow. Individual community coalitions may elect to add other components onto their campaign for local tailoring (e.g., communities may create materials for specific population groups based

on socio-demographic characteristics). Some communities may be able to use television and radio, whereas others may not, due to potential spillover effects. Communities may choose to focus on specific social media platforms, such as Facebook, Instagram, Snapchat, Reddit, or Nextdoor, or use paid targeted advertising on such social media and other outlets.

Each campaign provides the opportunity to leverage communication and media assets in the local community to reach a larger and broader part of each community to engage in the study and to spur demand for EBPs such as (1) increasing OEND; (2) enhancing delivery of MOUD; and (3) identifying, reaching, and engaging high-risk populations in OEND and MOUD.

An overview of the approach for the Wave 1 communities' campaigns is shown in Table 4. Briefly, an HCS website with a dedicated page for each Wave 1 community is updated as new campaigns are launched in the communities. Planning activities for the first three campaigns are carried out by the HCS campaign team, including message concept testing for each campaign among community representatives of the priority groups. A similar planning and formative research process will be taken for the final two campaigns that are intended to focus more deeply on increasing MOUD retention and a refresh and repeat of a previous campaign (Community Choice). Each campaign includes a Prepare phase that involves co-creation of distribution plans with coalitions that leads to the successive launch of each campaign with core digital and print assets (e.g., digital advertisements, social media posts, and print posters and handouts) along with a campaign message guidance document for coalitions that wish to create additional materials to support the campaign. The Implement phase is coalition led with technical assistance and support provided by each RS and communications staff.

In a process identical to that conducted with the Wave 1 communities, Wave 2 communities will also conduct campaigns using the same campaign guidebook and messages as originally presented to the Wave 1 communities. These coalitions will go through a tailoring process to allow for potential targeting of high-priority groups, focusing on specific objectives or EBPs, and include revised messages and/or new media distribution channels.

Each campaign has pre-specified relevant priority groups of community leaders, health care providers, and people with lived experience (PwLE). Communication objectives include: (1) obtaining and carrying naloxone; (2) decreasing MOUD stigma; (3) raising awareness of MOUD treatment; and (4) staying in MOUD treatment. We will incorporate messages in each campaign directed toward stigma reduction as they relate to OUD, MOUD, OEND, and recovery. The key issues as they relate to each campaign are identified under the stigma targets column in Table 4.

Table 4: Overview of communication campaigns

Campaign	Theme	Priority Groups	Objectives	Stigma Targets
April 2020-July 2020	Naloxone	Community leaders, public safety, criminal justice,<br>Health care providers<br>People with OUD and their families	Increase demand and access to naloxone.<br><br>Carry naloxone.	OUD is a medical disease<br>People with OUD deserve the best medical care possible
July 2020-November 2020	Stigma	Community leaders, public safety, criminal justice,<br>Health care providers<br>People with OUD and their families	Reduce stigma	OUD is a medical disease<br>People with OUD deserve the best medical care possible<br>Anyone could develop an OUD<br>MOUD is a safe and effective path to recovery for many people
October 2020–March 2021	MOUD – Seek Treatment	Community leaders, public safety, criminal justice,<br>Health care providers<br>People with OUD and their families	Increase demand for MOUD<br>Increase MOUD prescribing<br>Increase access to, and availability of, MOUD	OUD is a medical disease<br>People with OUD deserve the best medical care possible<br>Anyone could develop an OUD<br>MOUD is a safe and effective path to recovery for many people
June 2021 – Dec 2021	MOUD - Stay in treatment and recovery	High-risk patients and families, their health care providers, friends, religious leaders, employers, and co-workers<br>Community agencies, businesses, and organizations that can support treatment referral and retention	Increase access and coordination for seeking and being referred to treatment for OUD<br>Increase treatment retention	Treatment is effective<br>Increase support for people with OUD and their families<br>Increase public acceptance and support for non-discrimination of people with OUD in employment, health care, and housing

Jan 2022 – June2022	Community Choice	Selected by a coalition	Consistent with objectives of the selected campaign	Consistent with targets of the selected campaign

Communication Campaign Development and Implementation Protocol

The LA conducted in CTH Phase 0 and the study baseline periods will identify local media outlets. Interviews with up to 10 media representatives in each Wave 1 community will provide more context for understanding those communities and their unique media environments that can be used in the CTH efforts.

The key steps for the implementation of each campaign as described in Table 4 are outlined in Figure 8. These steps will be codified in a campaign guidebook that the RS staff and coalitions will use to facilitate discussion of each campaign's priority groups, objectives, message maps, communication strategies and tactics, monitoring metrics, and milestones. As we expect, there will be limited capacity within coalitions to develop and launch campaigns along with other implementation goals (e.g., adoption of EBP strategies by community organizations and groups), and the compressed time frame in which to develop and field campaigns to affect study outcomes, the guidebook, and messages are a way to present options for quickly deciding the direction and content of the campaigns. We will also conduct message testing for the first three campaigns followed by more extensive concept and message testing for the Stay in Treatment campaign.

Figure 8: The Plan-Prepare-Implement model for campaign development and community engagement

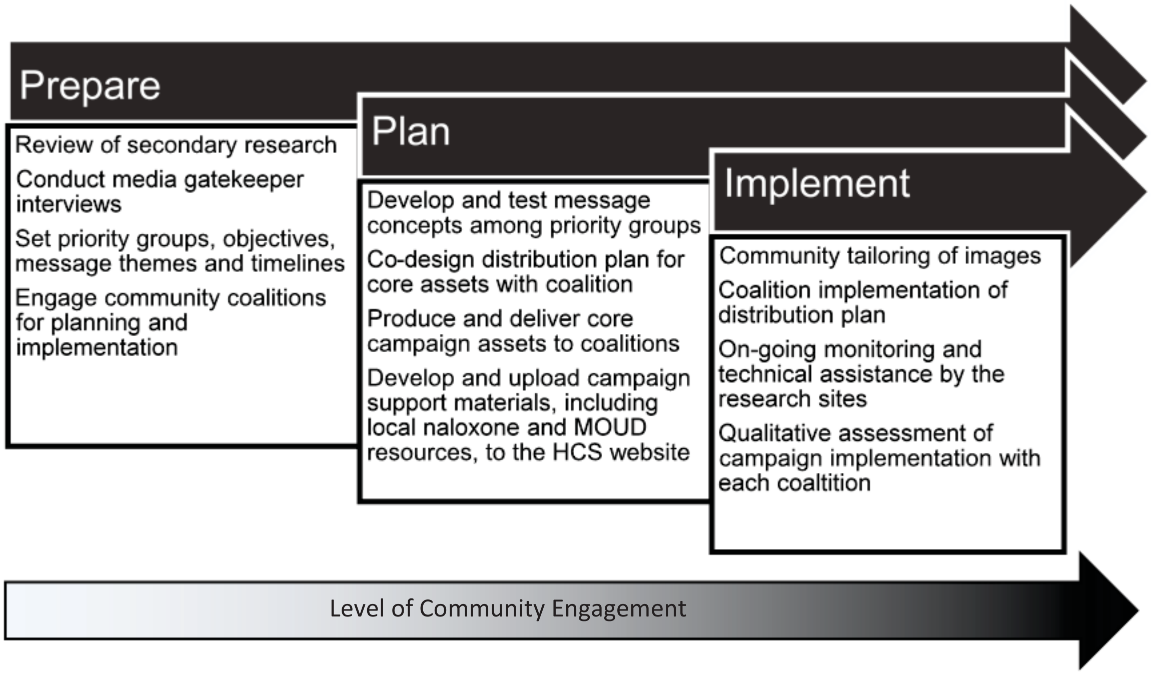

Formative research activities for each campaign will follow a standard set of methods and procedures that also allow for each community to adopt campaign practices and messages that are best suited to their local context, the capacities and assets of their coalition, current perceptions of the opioid overdose challenges in the community by different priority groups, and any unique preferences for messages and communication channels. In at least three Wave 1 communities, the RS will conduct up to 5 focus groups or up to 25 individual interviews for message testing per campaign. The number of groups or interviews in each community will be dependent on the size of the community (i.e., smaller communities may require fewer focus groups than larger ones), the desired priority groups to recruit (i.e., some communities may choose to focus on one group; others may choose to focus a campaign on more), and how many groups or individuals per priority group are judged as necessary to validate results (one may be enough in some circumstances such as with coalition members or stakeholders; two to three may be needed for people with OUD or high-risk users, especially in larger communities). Each of these groups will be composed of up to 10 representatives from a stakeholder group (e.g., community leaders, public safety or criminal justice, health care providers, treatment centers) or priority population (e.g., people with OUD, family members, heroin users, high-risk patients or people from their social networks, people currently taking prescription opioids). We anticipate that up to 1,800 people across all HCS communities may be included in these focus groups, individual interviews, or research design workshops for each campaign (see the following moderator and facilitator guides: *Focus Group CAB, Coalition, and Partner*

Organizations Guide; *Focus Group for Persons with OUD*; *Focus Group Testing of Launch Messages*; *Communication Design Workshop Guide*; *Interview Guide Message Testing*; *Interview Guide Message Testing Persons with OUD*), for an average of 54 participants per community.

Throughout the campaign development process, we will test specific concepts and messages to express the theme in a way that is most relevant, compelling, and useful for the priority groups in each community. Appendix B contains the pool of items that we will pull from for these message testing and formative research activities. The community coalition, local partners, and their respective communication assets (e.g., community-hosted websites, social media pages, newsletters) will be a critical part of the campaign message dissemination process. In Table 5, we present more details about the key steps in the Prepare-Plan-Implement process.

Table 5: Campaign Prepare-Plan-Implement process steps

**Prepare**
1. Develop campaign guidebooks, campaign resources for coalitions, and messages materials.<br>2. Conduct a literature review and environmental scan of previous communication efforts around opioids and EBPs.<br>a. Conduct a literature review and environmental scan of previous campaigns.<br>b. Extract data collected in the LA and qualitative interviews with coalition members to understand what communication assets and capabilities are available in communities to design, produce, and implement a communication campaign.<br>c. Conduct interviews with media representatives.<br>d. Perform online searches and seek information from the LA (asset mapping) and interviews with coalition leaders to learn about state or local communication efforts (previously conducted and currently on the ground); capture lessons learned from previous campaigns and note potential “competing messages” from current efforts.
**Plan**
1. Conduct up to five focus groups or up to 25 individual interviews for message testing groups in at least three Wave 1 communities with key stakeholders and/or representatives of priority groups for these interviews, focus groups, and design workshops (see *Focus Group for Persons with OUD*; *Focus Group CAB, Coalition, and Partner Organizations Guide*; *Focus Group Testing of Launch Messages*; *Communication Design Workshop Guide*; *Interview Guide Message Testing Persons with OUD*; and *Interview Guide Message Testing*).<br>2. Introduce campaign objectives, proposed priority groups, message materials, and findings from message testing and development timeline to community coalitions (via in-person or virtual meetings/presentations); solicit input and refine.<br>3. Introduce communication product ideas to each coalition; solicit input and refine ideas (e.g., social media copy, advertisements, sample op-eds, infographics).<br>4. Decide on message dissemination strategies and tactics with coalitions.<br>5. Articulate the implementation plan and review collaboration and integration touchpoints with CE and ORCCA teams.

6. Develop an implementation plan with the coalition including timelines, responsibilities, and milestones.	
**Implement**	
1. Coordinate release of first messages in the community (media choices to be determined via Landscape Analyses and media asset analyses) with kickoff events in each community.<br>2. Assist (where necessary) each community with selecting, using, and placing messages via previously selected/agreed-upon channels.<br>3. Engage partners to release campaign social media posts.<br>4. If placing paid advertisements (not required across communities), monitor advertisement placement and reach.<br>5. Hold monthly monitoring and feedback meetings of coalition/community implementation staff and RS.<br>6. Monitor social media and traditional media coverage of stories/trends surrounding naloxone, OUD, and MOUD.<br>7. Use monitoring data to make corrections to campaign implementation strategies/protocol and messages in each community (measures of campaign outputs including primary content, number and frequency of social media posts, advertisements, and earned media placements; feedback from coalition members and other community stakeholders).	

In summary, the communication campaign will start from an HCS-produced campaign guidebook and message maps that will be taken to each community coalition for discussion, refinement, testing, and implementation. Although core objectives and messages will be common across Wave 1 communities, there are opportunities for coalitions to refocus priority groups and co-create the strategies and tactics later in the campaign that will be empty in the final implementation plan. The campaign schedule for the entire intervention period, across both waves of communities and including each phase of the Prepare (for Wave 1), Plan, and Implement process, is summarized in Table 6a for Wave 1 and 6b for Wave 2.

Table 6a: Timeline for Stages of Communication Campaigns (Wave 1)

Stage	Naloxone Campaign Wave 1	Stigma Campaign Wave 1	MOUD Campaign Wave 1	Stay in Treatment Campaign Wave 1	Community/ Coalition Choice Wave 1	
**Prepare**	Sep 2019–Feb 2020	Sep 2019–Mar 2020	Sep 2019–Apr 2020	Nov 2020 – Apr 2022	Aug 2021 – Oct 2021	
**Plan with coalition**	Feb–Apr 2020	Apr–Jun 2020	Sep–Nov 2020	Apr 2021 – Jun 2021	Oct 2021 – Dec 2021	
**Implement**	Apr–Jul 2020	Jul–Nov 2020	Oct 2020 – May 2021	Jun 2021 – Dec 2021	Jan 2022 – May 2022	

Table 6b: Timeline for Stages of Communication Campaigns (Wave 2)

Stage	Campaign 1	Campaign 2	Campaign 3
**Plan with coalition**	Aug-Sept 2022	Jan-Feb 2023	Jun-Jul 2023
**Implement**	Oct 2022 – Feb 2023	Mar-Jul 2023	Aug-Dec 2023

Note, all campaigns were prepared during Wave 1. Each Wave 2 community will choose three of the four campaigns developed during Wave 1 (Naloxone, Stigma, MOUD, Stay in Treatment) to implement in three of separate campaign periods.

Overview of the HCS Communication Campaign Evaluation Study (HCS-CES)

Given the investment in health communication campaigns as a pillar of the HCS, a rigorous scientific evaluation is proposed across the 67 HEALing communities of the four RSs. Central to this evaluation is a cross-sectional, repeated-measure, longitudinal community-level measurement of the attitudes, intentions, and perceptions of EBPs to prevent and treat OUD among the recipients of the campaign in HCS communities. Furthermore, the evaluation will measure the impact of the health communication messages for persuading individuals to seek out EBPs for OUD and the campaign's impact on reducing stigma surrounding OUD, OEND, and MOUD. All data collection points in the evaluation will coincide directly with the campaign activities for each wave of the HCS, allowing for rigorous measurement of impacts at the community level and among the recipients of the campaign in the HCS communities. Campaign evaluations are also necessary to guide mid-course adjustments of strategies and tactics and arrive at sound, evidence-based recommendations for the use of campaigns to address opioid and other substance use epidemics.

The **HCS-CES** therefore has three primary components:

1. A tracking study of the impact of HCS messages through daily monitoring and analysis of message dissemination activities and citizen exposure to them. The tracking study will also measure responses to action steps contained in the messages (i.e., community-specific URLs directing users to the HCS campaign webpage on the HCS website). Time-series analyses will be used to detect dose-response associations between the level of campaign activity and audience information-seeking on the HCS campaign webpage.
2. Monitoring of other opioid-related communication campaigns sponsored by state and national organizations will document other sources of information that residents of HCS communities may have been exposed to during the CTH intervention. Salient and frequent messages from one or more of these external campaigns may be incorporated into the longitudinal surveys to assess their recognition relative to the HCS campaign materials.
3. Survey data collection of community residents to assess changes in specific components of stigma toward individuals with OUD, the acceptability of naloxone

(OEND), and the acceptability of MOUD treatment in the Wave 1 and Wave 2 communities (see Figure 9 for a timeline and visual representation of data collection points for the HCS-CES community surveys). Survey data will be collected from a sample generated from social media recruitment (Facebook). In addition, participants may opt in to be part of a longitudinal panel in which they are surveyed at each time point. These changes will be assessed across communities using differences between treatment and control communities and within communities over time by dose-response effects associated with exposure to campaign messages and other messages in the community environment. See *Campaign Evaluation Questionnaire.*

HCS-CES Research Hypotheses

H1: Time-series analyses of tracking data will indicate positive associations between higher levels of message dissemination activities and audience information-seeking on the website.

H2: The number of campaign messages correctly recognized, and the reported frequency of exposure to these messages, will be positively associated with desired attitudinal outcomes regarding stigma, naloxone distribution, and MOUD treatment at the individual respondent level.

H3: In intervention communities receiving the health communication campaigns, self-reported stigma toward individuals with OUD will decline, and acceptance of EBPs will increase significantly over time at the community level.

H4: In intervention communities receiving the health communication campaigns, self-reported awareness and acceptance of MOUD and OEND at the community level will significantly increase over time.

Because components 1 and 2 of the HCS-CES do not involve human subjects or collection of personally identifiable information (PII), the rest of the discussion of the HCS-CES will focus on the third component: the survey data collection of community residents. There will be seven data collection phases:

(1) CEQ1: March-April, 2020, Wave 1 and 2 communities (baseline)

(2) CEQ2: September-October, 2020, Wave 1 only (post-Campaign 1)

(3) CEQ3: January-February, 2021, Wave 1 only (post Campaigns 2 and 3)

(4) CEQ4: May-June, 2021, Wave 1 and Wave 2 (pre-Campaign 4 and beginning of CTH evaluation phase, secular trends comparison in Wave 2)

(5) CEQ5: November-December, 2021, Wave 1 only (post-Campaign 4)

(6) CEQ6: May-June, 2022, Wave 1 and Wave 2 (post Campaign 5, CTH evaluation phase comparison)

(7) CEQ7: May-June, 2023, Wave 1 and Wave 2 (Campaign effects in Wave 2, sustainability comparison in Wave 1)

Community Survey Participants

The study population for the HCS-CES community surveys will include any community member older than age 18 who resides in one of the 67 HCS communities. These individuals will be recruited for the campaign evaluation surveys via a series of Facebook/Instagram advertisements targeted to people aged 18 or older who reside within the ZIP Codes of the 67 communities. Data from the Health Information National Trends Survey demonstrates that social media is an effective tool for health communication research.[52] Specifically, researchers have found Facebook to be a viable platform for recruiting participants from lower socio-economic backgrounds,[53] recruiting representative samples,[53] and contacting hard-to-reach populations.[54] Using Facebook is also a cost-effective recruitment strategy.[53,55] Additionally, Healthy People 2020[56] has demonstrated the reach and penetration of mobile device data plans and found no significant difference in accessing Internet via cellular networks on the basis of rural versus urban communities, gender, Hispanic and non-Hispanic populations, and educational attainment.[57] Recruiting via social media has become an increasingly popular method for conducting surveys, because recruitment for telephone surveys has become increasingly challenging.

Prior to completing the pre-test survey (more details below in the HCS-CES procedures section), participants from Facebook/Instagram samples will be asked to complete a brief two-minute screening survey. Respondents will be routed to a screening survey that RTI has programmed in the Qualtrics survey system. RTI's license includes the premium data isolation feature, meaning the data are encrypted at rest. As a part of the screener, participants will be asked to provide their email address. For this question, the participant will be directed to a separate instrument so that the email address is stored within the Qualtrics system but separately from the other screener questions. Responses to screening questions will also be kept separate from the pre-test, intermediate, and post-test survey data since these data will be hosted on REDCap (more details below). This screening survey will be completed by each new sample obtained for subsequent campaigns.

The Qualtrics survey system has advanced capabilities to monitor the screening success rates, and to prevent fraudulent activity that is pervasive during social media survey recruitment. Using Qualtrics allows the study to implement best practices for social media survey respondent recruitment.

Respondents deemed eligible for the survey based on responses to the screener will be routed to a REDCap survey. Upon completion of the pre-test, intermediate, or post-test surveys, participants will be offered the opportunity to enter a drawing to win a $100 Amazon electronic gift card. One electronic gift card will be distributed to one winner in each of the 67 communities per data collection period. Those individuals wishing to enter the drawing will be offered the opportunity to voluntarily enter their name and email address at the end of the survey, so they can be contacted if they win the gift card.

The primary analysis is to assess the average stigma change in communities with the campaign. We expect to see a mean change score of at least 0.05. Furthermore, based on pilot data from Maulik, Siddhardha, Sudha, Abha, Shailaja, Miria, and Thornicroft (2017), we assume a SD of 0.09 for paired changes. However, our data are collected cross-sectionally, and therefore the SD corresponding to changes from pre to post will be larger. To be conservative with our sample size calculations, we assume a very large correlation of 0.95 for

pairs, which maps the 0.09 SD to a conservative SD of 0.40 for our cross-sectional study. Furthermore, we calculate required numbers of surveys per community corresponding to intra-cluster correlation coefficient (ICC) values ranging from 0.01 to 0.05, which statistically account for differences in mean changes across the communities. Based on these conservative assumptions, we will need approximately 20 to 80 surveys per community, on average, to be completed at each time point for each of the 33 Wave 1 communities in order to have at least 80% power for a two-sided test at the 0.05 significance level. With respect to baseline data for Wave 2, if we collect the same number of surveys across these 33 other communities, then these assumptions imply we will be able to estimate the true mean stigma score within +/- 0.024, based on a 95% confidence interval.

Data collection for each time point in the REDCap survey will be programmed to continue until this number of desired completions has been achieved. If a participant elects to not volunteer for future surveys or enter the raffle, all surveys will be anonymous. We will not be collecting any PII other than basic demographics (age, gender, race/ethnicity, educational attainment, and whether they self-identify as a health care provider or community leader). If participants elect to volunteer for future surveys, the raffle, or both, their information (name and email address) will remain confidential and will be used only for purposes of contacting for future surveys or informing them that they won a raffle.

HCS-CES Procedure

As discussed previously, data will be collected across the five campaigns in Wave 1 communities with annual inclusion of Wave 2 communities for comparison purposes. Wave 1 communities will also be included in a follow-up CEQ to measure sustainability of knowledge, attitudes and behaviors and Wave 2 communities will receive a CEQ to measure the overall impact of the campaigns across the year of implementation. These data collection points represent our cross-sectional study design. The following sections describe the data collection process for the first campaign for Wave 1 communities; these procedures will then be repeated for the second campaign for Wave 1, as well as for campaigns 3, 4, and 5.

Details of proposed survey of attitudes regarding stigma, naloxone availability, and MOUD for each discrete campaign

Campaign Evaluation Questionnaire: For the proposed community survey, the Facebook advertisements will target individuals living in the geographic regions of all 67 HCS communities. The survey will be programmed to continue until the number of desired completions has been achieved. Each discrete CEQ will be staggered to serve as both pretest for the beginning campaign and posttest for each previous campaign as test messages from all previous campaigns will be iteratively added to each subsequent CEQ. This will provide an overall impression of message recognition from each previous campaign (via cued recall of messages used in each previous campaign) as well as the current campaign.

The CEQ includes measures of stigma toward individuals with OUD and EBPs to prevent or treat OUD, as well as awareness and intentions to seek MOUD and OEND. Using a cued-recall survey methodology (discussed as follows), respondents will also be asked questions about recognition and attitudes toward other competing campaign messages being disseminated in their community. They will not be shown HCS messages at baseline. Survey completion is estimated to require approximately 20 minutes. Individuals will be asked if they are willing to be

re-contacted for the second and third time points of the campaign currently running in their community, and if willing, to provide an email address to help construct the cohort panel discussed previously. This process will be duplicated for all subsequent Campaign Evaluation Questionnaires.

The basic procedure for the cued-recall survey methodology involves two steps. Participants will first answer a set of demographic questions and items that assess their beliefs about evidence-based treatments for OUD, stigma surrounding OUD and treatments for it, and attitudes toward individuals with OUD. After completing these sections. each participant will be shown the current HCS messages being disseminated in their community, an additional message from non-HCS campaigns currently running in their state (e.g., sponsored by a federal, state, or non-profit organization), and a foil (or fabricated) message that will aid with calculating alternative explanations for message exposure effects.[58] After each message, the participants will complete brief measures using the Message Impact Framework (MIF)[59] to assess the perceived effectiveness of the messages, recognition of the messages, and frequency of self-reported exposure to the messages. We intend to submit these test messages to the sIRB as they are finalized and produced.

The data will be aggregated to the community level and aggregated for all individuals recruited in a given community. RSs have the option to conduct site-specific analyses so that they can share and give back non-identifying CEQ data to their HCS communities.

Measures

The measures for the proposed surveys (see Table 7) cover 10 areas related to the specific aims and hypotheses of the CES.

Table 7: Measures by data collection point

Measure	Baseline Surveys and Initial CEQ	All Subsequent CEQs	Campaign CEQ Post-Tests
HCS Study and Campaign Awareness	X	X	X
Barriers and Knowledge	X	X	X
Personal Stigma	X	X	X
Provider Stigma	X	X	X
Self-Efficacy	X	X	X
Social Distance/Community Stigma	X	X	X
HCS Message Impact		X	X
Behavior	X	X	X
Personal Experience	X	X	X
Demographics	X	X	X

To meet the aims of the CES and test the proposed hypotheses, the following measures are included: (1) initial measures of *recognition* of the HCS and HCS campaign (adaptation[60]), as well as of other major communication campaigns that may be circulating in the treatment and control communities; (2) measures of *barriers* to uptake of MOUD and OEND and *knowledge* about MOUD/OEND (developed and refined by the Communications Workgroup); (3) measures of *personal stigma* (adaptation from work on predictors of depression stigma[61,62]); (4) measures of *stigma toward MOUD providers* (adaptation[62]); (5) measures of *self-efficacy*;[62,63] (6) measures of *social distance (community stigma)* as currently approved for the HCS Coalition Baseline Survey, (7) a *message impact scale* that tests perceived message effectiveness (adaptation[59]), (8) measures of *behavior* surrounding OUD, naloxone, and treatment (adaptation[60]) and from the Communications Workgroup, (9) items measuring *personal experience with OUD* (adaptation[60]), and (10) a series of *demographic questions* that directly align with those from the HCS Coalition Baseline Survey. See Figure 9 for a graph of measures by data collection point and instrument.

Overview of Health Economics Plan for Intervention Protocol

We will conduct health economics research to determine the incremental costs of startup and ongoing implementation of the CTH. We will combine these cost estimates with data on the reduced number of opioid overdose deaths attributable to the CTH intervention to estimate the cost effectiveness of reducing opioid overdose deaths, yielding an estimate of the additional cost per averted opioid overdose death. We will also develop a microsimulation model that simulates the natural history of OUD and the effects of prevention and treatment methods, and the CTH intervention. Using the inputs and outcomes of the microsimulation model, we will develop an interactive online tool for policy makers and the scientific community that will allow users to select a geographic area and to evaluate the costs and effectiveness of alternative interventions for that particular area.

The initial phase of the project is focused on estimating the costs of the CTH intervention. To estimate these costs, we use an activity-based costing approach that captures the time spent by staff, space and material resources associated with implementing the CTH intervention components, and other direct costs such as medication, transportation, and staff training.

We will estimate the cost of the core components of the CTH intervention: the CE process, the communication campaign, and the ORCCA EBPs. Health economics measures include instruments that estimate the CE costs of the CAB meetings and activities performed by CAB members; the cost of the community coalition meetings; the cost of activities performed by project staff to facilitate the CTH intervention in communities; and the cost of the preparation, planning, and implementation phases of the communication campaign.

The simulation model will draw on cost, outcome, and transition probability data from the literature and the HCS; no additional primary data collection is anticipated for the simulation model.

6.2 Fidelity

6.2.1 Fidelity Measures for CTH Community Engagement Intervention

We will use several indicators and sources of data to assess the fidelity of how we deliver the CTH intervention and implementation strategies across the Wave 1 communities and Wave 2 communications when they start implementing the CTH. Fidelity broadly defined is “the extent to which an intervention is implemented as intended”. Fidelity constructs include (1) dosage or exposure to CTH using attendance data (2) adherence using the CTH fidelity checklist and meeting minutes (3) quality of delivery using meeting minutes and ethnographic observation (MA only); (4) output of coalitions as measured by production of such activities as a community profile, community action plan, community dashboard and implementing ORCCA-related trainings (5) participant responsiveness to CTH as measured by feedback from coalition members in annual surveys and qualitative interviews and participant feedback forms and (6) any adaptations or modifications to CTH intervention that communities make. Collectively these different measures will help us assess the extent to which CTH intervention was implemented as intended, the quality of implementation and what adaptations/modifications were made to inform the refinement and dissemination of the intervention if it is found to be effective on study outcomes. If the CTH intervention is for some reason not found to be effective, the fidelity measures will help us understand to what extent the lack of fidelity to CTH may have contributed to the failure to detect significant effects.

The primary source of data will be the CTH Milestone and Benchmark Checklist, which asks the level to which each activity of CTH CE is completed successfully. Responses are coded on a five-point Likert scale: 4=completed, 3=mostly achieved, 2=somewhat achieved, 1=just began, and 0=not started yet. The checklist also asks participants to indicate whether they completed the activity before the CTH CE intervention or during the CTH intervention, what barriers and facilitators hindered or helped their ability to complete the activity, and whether they made adaptations or modifications to the activity. During qualitative interviews, community coalition members will also be asked about challenges they are experiencing adhering to certain CTH CE activities and whether they have made adaptations to certain activities. Another key source of data includes regular review by the study team of coalition meeting minutes and documents of key outputs from CTH CE activities to identify the level to which certain activities are completed; barriers and facilitators to completing certain activities and the extent to which different members of the coalition attend meetings and contribute to the completion of activities; and whether participation in certain activities varies by gender, race, age, educational attainment, or geographic status. The study team will review qualitative interviews, meeting minutes, attendance records of meetings, and anonymous coalition participant evaluation forms to examine the extent to which all members are engaged in decision making, share common goals and objectives, engage in mutually reinforcing activities, and can identify and prioritize activities of the coalition that align with the CTH CE intervention. Table 8 specifies the fidelity measures that will be used to assess adherence to the specific activities of the seven phases of CTH and the implementation strategies that will be used to implement CTH.

Table 8: Fidelity measures and implementation strategies

Processes and Activities Within Each Phase	Fidelity Measures	Type/Sources of Data	Time Points for Data Collection
Phase 0: Pre-Intervention<br>Formation of Study CAB, Share information with communities regarding randomization; Landscape Analysis Phase 1 and 2 and Reporting, Training HCS staff on community engagement; Formation of Coalitions<br><br>Phase 1: Getting Started<br>Establish a structure for working with coalitions (charter); Recruit Champions, Train Community Coalitions, Plan Communication Campaign; Select Priority groups for Campaign; Introduce ORCCA Menu: Discuss Guidebook and Maps with Coalition, Coalition Review of LA Community Assets Data<br><br>Phase 2: Getting Organized<br>Discuss ORCCA Menu Options<br>Develop a shared vision; Commence and Evaluate Communications Campaign<br><br>Phase 3: Community Profiles and Dashboards<br>Creation of Community Profile 1.0; Creation of Data Dashboard Template; Map existing services to ORCCA Menu<br><br>Phase 4: Community Action Plan<br>Develop Implementation plan and Implement ORCCA EBP Strategies; Start Implementing EBPs<br><br>Phase 5: Implement and Monitor EBPs<br>Continue to implement and evaluate ORCCA EBPs, troubleshoot and provide technical assistance	CTH milestone and benchmark checklist for all Phase 1–6 activities<br><br>Meeting minutes and attendance records for all Phase 1–6 activities<br><br>Qualitative interviews to identify barriers and facilitators of completing Phase 1–6 activities<br><br>Repeated partner tool survey<br><br>Document review and upload:<br>Landscape reporting from Phases 1 and 2;<br>Charter;<br>Communication campaign plan and list of priority groups; Community profile, Dashboard template; Mapping of existing sources to ORCCA menu: ORCCA EBP strategy selection; Evaluation of 1st and 2nd communication campaigns: Implementation Plan for ORCCA EBPs<br><br>Coalition member participant evaluation forms	HCS CE facilitators<br><br>HCS staff<br><br>Coalition participants and key stakeholders	Monthly<br><br>Continuous<br>After every Meeting<br><br>Annually<br><br>Annually<br><br>Continuous after document completion

Phase 6: Sustainability Planning<br>Build capacity and align resources, training of coalitions through Learning Health Collaborative			
**Implementation Strategies**	**Fidelity Measures**	**Type/Sources of Data**	**Time Points for Data Collection**
<u>CTH CE implementation strategies</u><br>• CTH training of coalition members on CE strategies<br>• CTH technical assistance and supervision on CE strategies<br>• Utilization of Dashboard for planning and implementing CTH CE activities<br>• Group Model Building Implementation Strategy (NY Site Specific)<br>• Use of dashboard and portal data to guide CTH and ORCCA activities	Number and type of CE and ORCCA training and TA activities completed using training and TA forms, including CE supervision via learning health collaborative meetings<br><br>Number and type of CTH supervision meetings held<br><br>Attendance records of community coalition members and organizations at CTH supervision meetings	Designated community coalition chair or representative	Monthly<br><br>Annual<br><br>Continuous
	Qualitative interviews and repeated surveys	Coalition members	Annual
	Participant evaluation forms on training, education (site optional)<br><br>Participant evaluations of group model building (GMB) Implementation Strategy (NY site specific)<br><br>GMB Field Notes (NY site specific)	CTH/CE training, technical assistance and supervision coordinators<br><br>Training and TA participants<br><br>GMB participants<br><br>HCS research staff	Continuous
	Dashboard and/or portal	Dashboard or community portal search and utilization logs	Continuous

Following is a description of the administration of the fidelity measures listed in Table 8.

CTH milestone and benchmark checklist: HCS CE staff hired by the study will be asked to complete the checklist using a REDCap computerized survey on a monthly basis throughout the CTH intervention period (Phase 0 through Phase 6). Staff will log into REDCap every month to report on intervention fidelity. Each RS will send checklist data for their communities that are in the active intervention phase.

Attendance records: HCS staff will take attendance at every regular HCS designated coalition meeting or conference call in each community implementing the CTH intervention. The attendance sheet will list names of participants, their organizations, and their contact information. The project director (PD) or other designated HCS research staff in each community will enter the participant into the REDCap program and report the total number of participants who attended the meeting, the total number invited to the meeting, the total number of organizations represented at the meeting, and the total number of organizations invited to the meeting. After the PD or designated HCS research staff member finishes entering this attendance information into REDCap, they will store the attendance sheet in a folder in a separate locked file cabinet without any research data in their office, or they will scan a PDF of this sheet and save it in a password-protected, encrypted file on their computer. Hard copies and electronic files of attendance sheets with identifying information will be destroyed at the end of the study after quality assurance is completed. Attendance information will also be collected from subcommittee and work group meetings or conference calls that occur outside the regular HCS designated HCS coalition meeting that will include (1) date of meeting/conference; (2) number of attendees at the meeting/conference call; (3) topics covered in the meeting; and (4) length of meeting in minutes.

Meeting minutes: HCS staff will take minutes of every coalition meeting using a template or an existing meeting minute form. Meeting minutes are critical to HCS study outcomes; thus, the research sites have the option to audio and/or video-record coalition meetings held either in-person, web-based/remotely, or via a hybrid approach where some members are in-person and some join remotely. At the beginning of every meeting, HCS staff will apprise coalition members and attendees that they will be taking meeting minutes that will be used for research purposes to better understand how the coalitions are implementing the CTH intervention, to identify barriers and facilitators of implementing CTH, and to identify any adaptation. If applicable, coalition members will also be informed that the meeting will be audio and/or videorecorded for the sole purpose of creating detailed meeting minutes. At the coalition's request, meeting recording(s) can be shared with coalition members. Coalitions may also choose to take their own meeting minutes and/or record their own meetings for internal purposes. HCS staff will let coalition members know that they can ask to have any comments off the record, and those discussions will not be included in the minutes. The coalition will receive a copy of the minutes by email, and coalition members may ask to remove or edit any portion of the minutes. Coalitions have the final say in what type of information is included in their meeting minutes (some do not want members' names associated comments and questions made during the meeting, while other coalitions do). HCS staff will upload meeting minutes into a REDCap program or a similar secure, electronic environment along with information on the community and the date of the meeting. If the meeting is audio and/or video-recorded, recorded files will be transcribed, and audio and/or video files will be destroyed within 6 months of being transcribed.

The procedures of taking meeting minutes and using them for research purposes will also be described in coalition charters.

<u>CTH document upload and review:</u> Coalitions will be asked to provide electronic copies of selected documents they produce during CTH activities, including community-specific LA and reporting beyond standard format, the coalition charter, the communication campaign plan and list of priority groups, community profile, data dashboard template, mapping of existing sources to the ORCCA menu, ORCCA EBP strategy selection, evaluation of the first and second communication campaigns, and the implementation plan for ORCCA EBPs. HCS research staff will upload documents into the REDCap program or a similar secure, electronic environment. For document upload, HCS research staff will be asked to indicate what type of document, the community coalition, and the date of upload. HCS research staff may later review and evaluate documents to better understand how CTH activities were implemented and to monitor progress of coalitions and provide feedback for quality improvement. The procedures of collecting and using CTH documents for research purposes will also be described in coalition charters.

<u>Coalition member participant evaluation forms:</u> HCS research staff may administer paper copies (or electronic copies if the meeting is held remotely/web-based) of anonymous brief participant evaluation forms to coalition members after meetings to elicit feedback on their level of satisfaction with different aspects of the CTH intervention and conduct of the meeting, as well as their suggestions for improving the meetings in the future. If the meeting is held in-person, participants will be asked to complete forms and place them in a large envelope when they are done. No identifying information or participant ID numbers will be included on the forms. Data from the forms will be entered into a secure REDCap program. After quality assurance is conducted, paper forms will be destroyed.

<u>Training and TA tracking:</u> RSs will be asked to fill out training or TA service forms for CE or ORCCA training and TA activity in Wave 1 communities on a monthly basis. Data from the forms will be entered into a secure REDCap program. After quality assurance is conducted, paper forms will be destroyed. The collected information will support analyses of ORCCA implementation and costing activities.

<u>Coalition dashboard and portal website logs:</u> HCS RSs may download and review logs or archives of data searches and visualizations conducted by coalition members or key community stakeholders to better understand what type of data coalitions are requesting and how they are using data to guide their planning of CTH and ORCCA activities. These logs will be coded by community site ID number and date. Coalition members will be apprised that their data logs and searches will be saved and used for research purposes on search pages of dashboards and website portals. The procedures of collecting and using these logs for research purposes will also be described to dashboard and portal users when they request access.

<u>Portal Functionality Group Interviews:</u> HCS RSs will conduct group interviews with community members and research staff who actively utilize the community portals and dashboards as part of the HCS. These group interviews will inform the RSs about whether and how dashboards have been useful in CTH decision making, whether they are easy to use and understand, whether they will continue to be used and for what purpose(s), and whether and in what capacity the landscape data will continue to be used to populate the HCS dashboards. A short questionnaire will also be administered electronically via REDCap to gather basic

sociodemographic information and assess the respondent's satisfaction with using the CTH portal and dashboard.

The Group Model Building (GMB) participant feedback form: This is a brief, semi-structured instrument intended to evaluate GMB participants' ratings of the quality of the workshop's facilitation, content, clarity, and perceived utility. The instrument includes 13 six-point Likert-type items (6=excellent, 5=very good, 4=good, 3=fair, 2=poor, and 1=very poor) and three short-answer items (to capture specific comments about their experience as a participant). The form was adapted from its original version, which was developed by Zimmerman and colleagues.[64] GMB participants will be asked to complete this form by hand after a workshop session. Completed forms will be placed in a large envelope. Forms will be completed anonymously. No identifying information or participant ID numbers will be used. After quality assurance is conducted, paper forms will be destroyed.

Applying lessons learned from group discussions: HCS research sites may hold group discussions with members from the HCS research cores, community coordinators, fiscal agent representatives and others involved in the CTH intervention to gather feedback and lessons learned for process improvement. These discussions may help inform approaches used with HCS communities and will ensure an intentional and inclusive approach. Lessons learned and process improvement strategies may be shared in presentation and publications and may be shared with community organizations for their own program improvement.

6.3 Implementation Science Measures for Communities That HEAL

Although the primary aim of the HCS is to evaluate the effectiveness of a community-engaged intervention on reducing opioid overdose fatalities, the HCS provides a unique opportunity to extend knowledge regarding the factors that mediate or moderate the impact of the intervention on this critically important public health outcome in 67 communities in four states. To conduct a scientifically rigorous study of the implementation process, the HCS is informed by an adaption of the RE-AIM/PRISM model.[65] Similar to other frameworks in implementation science,[66-71] RE-AIM/PRISM emphasizes the inter-relationships between inner and outer context, the interventions to be implemented, implementation strategies, and implementation outcomes. The adapted RE-AIM/PRISM model guiding the HCS is presented in Figure 10. The primary adaptation is represented in the circle, where the generic "evidence-based intervention (components)" and "implementation strategies" have been replaced by the CTH intervention (i.e., the CE strategy focused on community coalitions, the communication campaigns, and the ORCCA), which emphasizes the scaling up of EBPs across a range of organizational settings that serve individuals who are at elevated risk of opioid-related overdose (e.g., criminal justice, behavioral health, medical organizations).

Figure 9: RE-AIM/PRISM model adapted for HCS

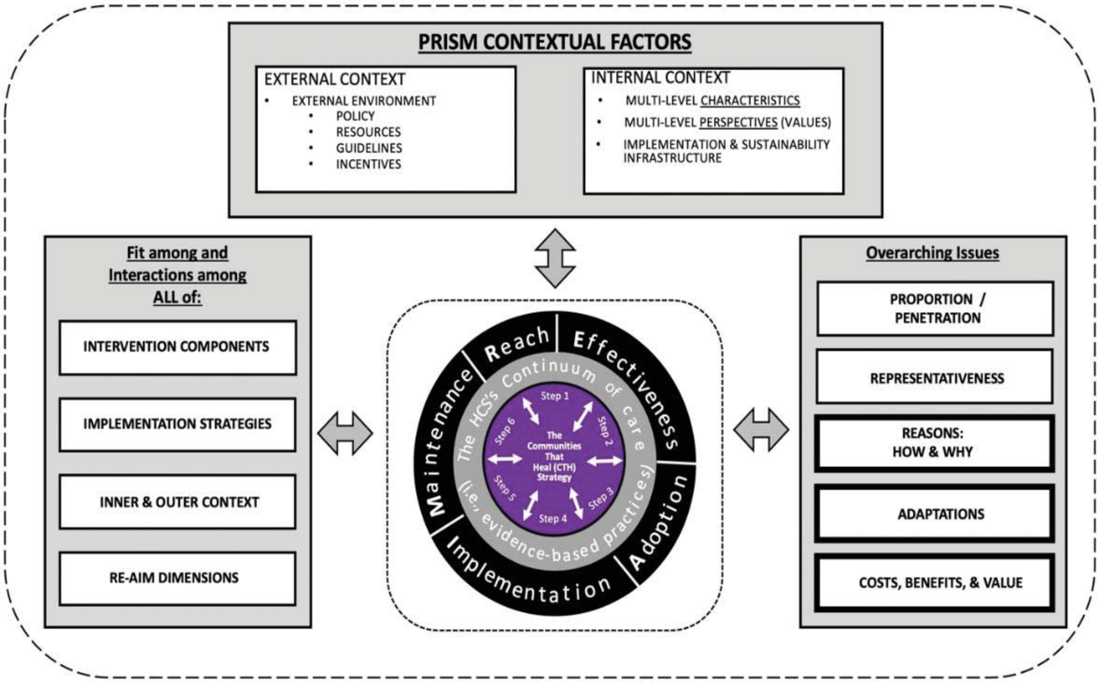

Source: Glasgow and colleagues.[65]

The purpose of the implementation science data collection is to measure coalition members' and key stakeholders' perspectives on the opioid epidemic in their communities, current responses to the opioid epidemic, and factors in the internal context (i.e., coalition perspectives and characteristics) and external context (i.e., community perspectives and characteristics) that may facilitate or impede the reduction in overdose deaths in these 67 communities when the CTH intervention is deployed. The implementation science assessments will rely on observational research methods, including qualitative semi-structured interviews and quantitative surveys to measure key components relevant to the RE-AIM/PRISM model. In addition to baseline data collection, similar data will be collected annually in all 67 communities at three additional time points during implementation of the CTH.

6.3.1 Study Participants

Study participants will be (1) members of community coalitions or (2) key stakeholders (e.g., representatives from treatment organizations, criminal justice organizations, medical and public health organizations, emergency services, faith-based organizations) in the 67 HCS communities. All participants will be at least 18 years of age. The number of participants per community will vary based on the size of the community, the size of the community coalition, and the plans of each RS, but a total of up to 2,010 participants across the 67 communities (approximately 30 per community) are anticipated to participate in baseline and three follow-up timepoints of data collection activities (approximately 15 months, 30 months, and 48 months). In Massachusetts and New York, human subjects will also include members of a statewide CAB that represents all communities and key state stakeholders.

Participants will be excluded if they

- show evidence of significant psychiatric or cognitive impairment as confirmed during written informed consent, or
- are not fluent in English as determined during written informed consent (we estimate that fewer than 5% of potential participants will be excluded for lack of fluency in English).

6.3.2 Recruitment Procedures

Subject recruitment for baseline data collection will vary among RSs because in some communities, a community coalition exists, whereas in other communities, a coalition will be formed in the early phases of the HCS. Hence, key stakeholders will be recruited for data collection through existing relationships, snowball sampling, or purposive sampling. Regardless of whether recruitment begins via email, telephone, or in person, the study procedures will be explained, and potential participants will be given an opportunity to ask questions. Research staff from each RS will then obtain informed consent from all interested people; consent procedures will be based on the mode of data collection (e.g., in person, REDCap web survey, telephone, videoconference such as Zoom). For self-administered surveys where participants directly input their responses into REDCap, consent will be obtained via an introductory screen that provides relevant information for consent. For telephone interviews, verbal consent will be obtained. When data are collected via hard copy surveys (self-administered by the participant or via an in-person interview), written informed consent will be obtained. Each potential participant will be assigned an ID number by the local RS so that rates of participation can be calculated. All raw data files will be transferred from the research sites to the Data Coordinating Center (DCC). All research participants recruited into the study must be able to read and understand the English language.

6.3.2.1 Recruitment Procedures for the Qualitative Interviews With Coalition Members/Key Stakeholders

The following recruitment procedures will be used for the qualitative interviews with coalition members/key stakeholders:

- RSs will use purposive sampling to select coalition members or key stakeholders for a given community (if no coalition exists) from those identified for the *Baseline Coalition/Stakeholder Survey*. When selecting potential participants for the qualitative semi-structured interviews, RSs will consider the role of the individual (e.g., coalition chair, community coordinator, health commissioner, “champion” if the HCS intervention is active at the time of the interview), sector (e.g., MOUD, harm reduction, criminal justice, person with lived experience), and geographic diversity (e.g., if county includes more than one town, if more than one town is in the coalition). At follow-up, sites will use purposive sampling from recent coalition rosters or recommendations from key stakeholders (if no coalition exists in a given Wave 2 community).
- Initial contact with selected individuals may be made in person or via video/web conferencing (e.g., at a regularly scheduled meeting), by email, or by telephone to describe the purpose of the study, to describe compensation associated with participating, and to gauge potential interest in participating. Selected individuals may be sent an email invitation (see *Coalition Survey & Qualitative Recruitment*

Subject Facing Materials), which will describe the purpose of the interview and any compensation associated with participating in the interview. If there is no response to the initial email invitation in 2 days, the email invitation will be re-sent. Repeating the invitation will occur once more if the potential participant does not reply to the second invitation. If the participant's telephone number is available, the [RS staff role] may also follow up with those who do not respond to the initial email invitation by telephone (see *Coalition Survey & Qualitative Recruitment Subject Facing Materials*).

- Informed consent, including consent for audio-recording, will be obtained from participants before any interview data are collected. Individuals who do not consent to audio-recording may not participate in the interview because verbatim transcripts are needed for data analysis.

These recruitment procedures for the qualitative interviews with coalition members/key stakeholders will be repeated at three additional time points.

6.3.2.2 Recruitment Procedures for the Surveys With Coalition Members/Key Stakeholders

The following recruitment procedures will be used for the surveys with coalition members/key stakeholders:

- RSs will invite all coalition members (if coalitions exist) or will work with key contacts to identify key stakeholders (if no coalition already exists) for the survey. At baseline, in communities without coalitions, RSs may work to quickly analyze the initial responses to the social network section of the survey (which also identifies key stakeholders in a given community) to determine whether additional individuals should be invited to participate in the survey.
- RSs may work to identify Survey Champions who can introduce the baseline survey to coalition members/key stakeholders. The Survey Champion may be the key government official for an RS, a local or state government official, a member of the research team who is well known to the communities, and/or the chairperson of the community coalition if a community coalition already exists. Ideally, no less than 3 business days before individuals are invited to participate in the baseline survey (via an email invitation or during an in-person Phase 0 orientation meeting), the Survey Champion(s) will engage the community about the survey via email. The purpose of the Survey Champion email is to grant legitimacy to the HCS survey and to inform individuals that data collection may occur at the in-person HCS community coalition orientation session and/or they may be sent an email invitation with a link to the HCS survey, which will be sent from the RS's account (i.e., University of Kentucky, Ohio State University, Columbia University, or Boston Medical Center email account; e.g., HCS_KY@uky.edu) on a specified date. The email will also provide a brief introduction to the study's purpose and describe the target respondent's importance to the study and may be co-signed by multiple Survey Champions (see *Coalition Survey & Qualitative Recruitment Subject Facing Materials*).
- A 4-week recruitment process will be used for the survey at baseline:
  - In week 1, an email invitation (see *Coalition Survey & Qualitative Recruitment Subject Facing Materials*) will be sent, which will describe the purpose of the study and any compensation associated with participating in the study. This

email invitation will include information about how to access the survey via REDCap and may include information about completing the survey after the Phase 0 orientation meeting via computer/tablet or hard copy. This email may also include a PDF of the survey, so the participant can download the survey to print, complete, and mail or fax back to the HCS RS along with a hard copy version of the consent form.

- In weeks 2–4, if there has been no response to the previous invitation, an email reminder will be sent as well as instructions about how to contact the RS if the individual prefers to complete the survey by telephone or in person (see *Coalition Survey & Qualitative Recruitment Subject Facing Materials*). This email may also include a PDF of the survey, so the participant can download the survey to print, complete, and mail or fax back to the HCS RS along with a hard copy version of the consent form. In addition, at week 2, the Survey Champion may send a separate reminder email (see *Coalition Survey & Qualitative Recruitment Subject Facing Materials*). During weeks 2–4, the RS may follow up with non-respondents with a telephone call, text, letter, or fax to ensure that the email has been received and to answer any questions. At week 4, non-respondents will also be mailed a packet that includes a letter on the RS's institutional letterhead requesting participation, a paper version of the survey, and a prepaid addressed envelope (see *Coalition Survey & Qualitative Recruitment Subject Facing Materials*).

These recruitment procedures for the surveys with coalition members/key stakeholders, as relevant, will be repeated at three additional time points. It is anticipated that by the time of these future time points, coalitions will be established, so all coalition members at the time of the follow-up will be invited to participate. For the three additional follow-up surveys, an eight-week recruitment process will be used. Sites may choose to contact non-respondents using the methods described for the baseline survey (e.g., telephone call, text, letter, or fax) for up to 8 weeks. At week 8, sites may mail a hard copy survey to non-respondents, but this is a site-optional data collection strategy. Sites may also choose to announce the survey data collection at coalition meetings.

6.3.3 Compensation

Kentucky, Massachusetts, and New York propose to compensate all participants $50 (cash/gift card) for the community coalition and key stakeholder survey and $50 (cash/gift card) for the coalition and key stakeholder semi-structured qualitative interview, consistent with agreements in place with their community partners. Ohio will not compensate participants.

6.3.4 Instruments

Semi-Structured Qualitative Interviews With Coalition Members/Key Stakeholders (Core)

Semi-structured qualitative interviews are planned to be conducted at baseline in the 67 HCS communities with (1) community coalition members or (2) key stakeholders who may be considered for future coalitions in communities where there are no existing community coalitions addressing the opioid epidemic. For communities with existing coalitions at baseline that are partnering in the HCS, at least one member of the coalition will be interviewed; the leader for the

coalition will be prioritized if such a position exists. RSs may choose to interview additional members of the coalition, using site-specific processes to identify additional interviewees. For communities that do not have existing coalitions at baseline, the RS will identify at least one key community stakeholder to be interviewed. Additional interviews with stakeholders may be conducted, using site-specific processes to identify these interviewees. Although we anticipate that sites will conduct an average of 4–12 interviews per community, it is important to note that interviewees will be drawn from the same population as the survey, so most of these participants are included in the estimated survey sample (n=2,010).

Baseline interviews will be conducted in person to observe and record non-verbal cues when feasible; if this is not possible, they will be conducted via videoconference or telephone. Due to the COVID-19 pandemic, interviews at the follow-up time points will be conducted via videoconference or telephone. With participants' consent, interviews will be audio-recorded; in the event that an individual consents to be interviewed but not audio-recorded, the interview will be terminated, because notes would be insufficient for qualitative analysis. Interviews will explore key components of the implementation science conceptual framework and will be conducted ideally with the selected community coalition member at baseline, which is defined as before the start of the intervention phase, and again at three additional time points. For baseline data collection, respondents will follow one of two paths through the interview (see *Coalition and Key Stakeholder Semi-Structured Qualitative Interview Guide*). The attachment contains a version of the guide for coalition members and another version for those without an existing opioid coalition. The guide contains items measuring the community context (i.e., outer context regarding other efforts to address the opioid epidemic, support to expand EBPs) and measures of coalition history and current activities (i.e., the inner context of the coalition). For follow-up data collection, respondents will follow one of two paths through the interview based on whether they represent a Wave 1 or Wave 2 community (see *Follow-Up Coalition Member and Key Stakeholder Semi-Structured Interview Guide*). If the individual has not already completed the *Community Coalition and Key Stakeholder Survey* at baseline (see next section) or has not previously participated (i.e., person is a new participant recruited at follow-up time-point), demographic information (using the same questions from the survey) will be collected at the end of the interview. The *Locator Form* may be collected at the end of the interview but is not required.

Qualitative interview data collection will occur via a similar process at three additional time points after the baseline data collection. A future amendment will be submitted if there are changes to the specific items included in the interview guide at follow-up.

For HCS-related qualitative interviews (core interviews), the following procedures regarding transcription, data sharing, and analysis will be used. With participant consent, recordings of the qualitative interviews will be transcribed verbatim. Transcription will happen locally, using professional transcription services or a combination of transcription software with review by research staff for accuracy. Participants will be given a unique participant ID number that will link their data across sites and time points; if the participant has already completed the *Community Coalition and Key Stakeholder Survey*, the ID number issued for the survey will be used for the qualitative interview. A linking log connecting the ID number with the name will be stored in a locked file on a secure system available to only researchers at the RS.

In line with current IRB and National Institutes of Health ethical standards, recordings will be destroyed after transcription validation. Transcripts will be manipulated and analyzed using

qualitative software (e.g., NVivo, ATLAS.ti). To code the data, we will analyze each interview transcript in two ways: initially, we will code the data using factors in the study conceptual framework, identifying and creating codes in each of the core constructs. In coding, passages referring to the different conceptual categories of the framework and other areas will be marked and identified by cover terms that reflect the conceptual category. The marked passages will be compared to enable the identification of similarities and differences across the sites and the program models. Second, using the general procedures of inductive coding, we will also be able to identify additional themes that emerge organically from the data. The initial sets of transcripts will be coded by two researchers from each site skilled in qualitative analysis in order to create a codebook. After consensus coding, the remaining transcripts will be coded by two individuals. Constant comparative coding will be employed, so all transcripts are coded with the full set of codes that emerges. According to this method, the initial conceptual categories are then applied to new data, and the categories are revised to reflect the addition of the new data.

Surveys With Coalition Members/Key Stakeholders (Core)

Surveys will be conducted with community coalition members or key stakeholders who have been identified for future community coalitions before the start of the intervention, with the goal of collecting data from all coalition members or key stakeholders who consent to provide these data. The preferred method for distributing surveys is via REDCap's web-based electronic data capture survey module software. REDCap is housed in the Data Coordinating Center's (DCC's) and the RSs' secure, web-based services, and all appropriate measures will be taken to ensure the security of the system and the data collected. To ensure high rates of participation, RS staff may also distribute paper copies of the survey to coalition members in a group setting (e.g., the Phase 0 orientation meeting), may distribute laptops/computers in a group setting so that participants can directly input their responses into REDCap, may distribute a PDF of the survey via email, and may collect the survey data via telephone/videoconference. If coalition members complete the survey in a group setting, research study staff will ensure sufficient space between participants to ensure privacy (i.e., that participants cannot see each other's responses). In instances where data are collected on paper forms, PDFs, or by telephone, it is the responsibility of the RS to enter the data into REDCap.

Surveys will measure key components of the implementation science framework (see Figure 10). Survey measures include (1) the inner context of the coalition, such as coalition characteristics (e.g., trust and communication quality in the coalition, coalition leadership) and coalition perspectives (e.g., coalition readiness to change related to EBPs); (2) the outer context of the community (e.g., perceptions regarding the community's needs for addressing the opioid epidemic, perceptions of the community climate for expanding OEND and MOUD); (3) perceived characteristics of EBPs; and (4) coalition member/key stakeholder characteristics (e.g., demographics, the community sector they represent).

Surveys will be conducted with coalition members and key stakeholders from the 67 communities before the start of the intervention. Survey data collection will occur via a similar process at three additional time points after the baseline data collection. A future amendment will be submitted if there are changes to the specific items included in the survey at follow-up.

At the end of the *Community Coalition and Key Stakeholder Survey*, the *Locator Form* will be administered at baseline. If the participant is self-administering the survey via REDCap, the participant will directly input the *Locator Form* information. For all other modes of survey

administration, the *Locator Form* will be completed on hard copy, then entered into REDCap by RS staff. At follow-up data collection, the *Locator Form* is optional.

All survey participants will be asked to provide informed consent before beginning the survey. The consent process will emphasize the voluntary nature of participation, the rights of interviewees to decline to answer any question and to stop the interview at any time, and that de-identified data may be used by researchers in the future, because all HCS data will be archived for secondary analysis by non-HCS researchers once the primary study has been completed.

Statistical analyses will be performed on these implementation science survey data. Descriptive statistics (e.g., frequencies, means, standard deviations) will be calculated for all variables, and regression analyses may be performed. Individual responses will also be aggregated to community-level measures.

Community Advisory Board Member Survey

Members of the implementation science team in Massachusetts and New York will conduct a survey (see the *HEALing Communities CAB Member Survey*) with all CAB members to understand CAB structures and processes and their feelings about the work of the CAB. They will also collect basic demographic information on the CAB members. The conduct of this survey will use the same procedures as described for the other surveys described for core measures mentioned earlier. This tool will be used with each CAB—the estimated number is up to 30 participants in Massachusetts and 50 participants in New York. In addition to baseline data collection, we will conduct surveys annually with Massachusetts and New York CABs. At baseline, the *HEALing Communities CAB Member Survey* will include a tool that addresses trust in the research team conducting the intervention called the Partnership Trust Tool. The goal of this tool is to understand their relationship and level of trust with each other. This tool will be used with all CAB members in Massachusetts and New York.

Document Review

Members of the implementation science teams will review documents to better understand the communities. Examples of the types of documents to be reviewed include needs assessments, community profiles, meeting agendas/notes, TA logs, community information regarding different programs, department of health/public health initiatives, and other community resources. The *Document Review Guide* will be used to structure and standardize data collection. After data collection, the guides will be stored using the same procedures for security as described earlier for all survey tools. Data will be analyzed qualitatively using the concepts from the conceptual framework to guide analysis.

Ethnographic Analysis

Members of the implementation science team in Massachusetts will attend coalition and CAB meetings as ethnographic observers. The *Ethnographic Guide for Field Notes* template will be used to structure observations collected during these meetings. Data collection will focus on understanding interactions between participants, the interactions with the facilitation (CE team), and the meeting process. After data collection, the guides will be stored using the same procedures for security as described earlier for all survey tools. Data will be analyzed

qualitatively using the principles from the conceptual framework to guide analysis with a focus on understanding the role of the facilitation process.

Community Advisory Board Member Interview Guide

Members of the implementation science team in Massachusetts and New York will conduct semi-structured qualitative interviews with members of the statewide CAB. All members of the CAB (up to 30 in Massachusetts and 50 in New York) will be interviewed. Interviews will be conducted in person to observe and record non-verbal cues; if this is not possible, interviews will be conducted via videoconference or telephone. Interviews will explore key components of the implementation science conceptual framework. Interviews will be conducted at baseline, before the intervention begins (see the *Community Advisory Board (CAB) Member Interview Guide*). Procedures for CAB qualitative interviews will be identical to those described earlier for community coalition interviews but will occur only once.

6.4 Toxicology Survey

Toxicologists in Kentucky, Massachusetts, New York, and Ohio will be administered a survey (see *Toxicology Survey for Labs*) to gather data on the characteristics of postmortem toxicology testing for suspect drug overdose deaths and other characteristics of the medicolegal death investigation and certification of drug overdose deaths. The toxicology survey will be administered to all four states using REDCap. REDCap is housed in the DCC's secure, web-based services, and all appropriate measures will be taken to ensure the security of the system and the data collected. Descriptive statistics will be performed on the resulting data, including tabular frequencies, means, minimum, and maximum by overall data and by state and community. Listings and graphical displays will also be used as appropriate.

In addition, a brief REDCap survey (see *Medical Examiner Survey* [NY site-specific] and *Toxicology Survey for Medical Examiners & Coroners* [KY, MA, and OH site-specific]) will be distributed to one respondent per county to obtain an understanding of the procedures in each county related to investigating and determining opioid overdose-related deaths. Depending on the county or community, this respondent may be the county health or mental health commissioner themselves, a key staff member they identify, or a medical examiner or coroner. These surveys will be programmed and administered remotely by the site-specific research teams. The research teams will share the survey link via email with county-level partners and follow-up to ensure completion. Findings will be analyzed by the research teams.

6.5 Measures to Minimize Bias: Randomization and Blinding

HCS communities will be the unit of randomization and the unit of analysis. Randomization will be stratified by RS (i.e., Kentucky, Massachusetts, New York, and Ohio). In each RS, we will use a technique referred to as covariate-constrained randomization[21,22] in order to ensure balance between intervention and control arms on three key community characteristics: baseline opioid overdose death rate, population size, and urban/rural status. This technique will remove these variables as potential confounders and improve the power to detect the effect of the CTH intervention on the opioid overdose death rate. For the continuous covariates, opioid overdose death rate and population size, balance constraints will be set at <0.2 standardized difference between arms. For the dichotomous variable, urban/rural status, randomization will be constrained to require equal numbers between arms among RSs with even numbers of

urban/rural communities (i.e., Kentucky, Massachusetts, and New York), and a difference of no more than 1 among RSs with odd numbers (i.e., Ohio). In each RS, one allocation will be selected at random among all possible allocations that meet the aforementioned criteria.

The HCS will not be blinded. The communities will know which are in Wave 1 of the CTH intervention because they will start to receive the CTH process 2 years and 6 months before the wait-listed Wave 2 communities. Also, the RS members, including the Principal Investigators, will know the assignments of communities to Waves 1 and 2 because they will be working with the communities to implement the CTH. However, it is not expected that this will cause a significant chance for bias in the study. The HCS research staff will be at arm’s length from the service venues, and the professionals working in therein, where OUD patients are seen and receive services. Although the HCS will not be blinded to communities or the HCS team, the statisticians performing the analysis will be blinded. Once the analyses are completed, the wave assignments will be associated with the analytic results.

7. STUDY INTERVENTION DISCONTINUATION AND PARTICIPANT DISCONTINUATION/WITHDRAWAL

7.1 Discontinuation of Study Intervention

The HEALing Communities Study (HCS) could be discontinued for several reasons. Examples include the following:

- The National Institutes of Health (NIH) decides to withdraw funding and cancel the study.
- The Data and Safety Monitoring Board recommends, and NIH agrees, that the study is unsafe to continue due to adverse events or futile to continue due to lack of participation.
- A state decides to withdraw critical support for the conduct of the study and the study is discontinued in that state.
- A community coalition decides to withdraw critical support for the conduct of the study or decides to no longer participate and the study is discontinued in the associated community.
- A key political leader determines, however unlikely, that they do not want their community participating because they perceive that it could stigmatize their community or have some other negative impact on their community.

The study team will work closely with all HCS communities to ensure that issues, such as the aforementioned examples, are promptly addressed with the aim of achieving mutually beneficial outcomes. Additionally, the HCS Steering Committee (SC) will discuss any community-specific issues that arise that may adversely affect the study outcomes.

A specific evidence-based practice (EBP) strategy could be discontinued or eliminated from the package for a variety of reasons. For example, a practice could be discontinued if:

- its legal status changes in a state or local community, or
- it is determined to be unsafe (e.g., a medication presently approved for the treatment of opioid use disorder is withdrawn by the Food and Drug Administration).

7.2 Participant Discontinuation/Withdrawal From the Study

Individuals are not being enrolled into care by the HCS. Rather, the HCS is facilitating communities to enroll individuals into treatment, retention in treatment, enhancing provision of overdose prevention education and naloxone distribution, and other prevention strategies. If a community coalition or service venue chooses to cease participation in the HCS, they can do so. Efforts will be made to maximize retention in the study.

Community coalitions and participating service venues could be discontinued from participation if the SC determines that they are not delivering the EBPs as part of their agreement (e.g., an opioid treatment program closes). The study team will work with community coalitions and venues to problem solve and help facilitate improvement before discontinuation.

Individuals recruited for surveys and interviews could decide to discontinue.

7.3 Lost to Follow-Up

It is unlikely that a community coalition or community service venue will cease operation without notice. Efforts will be made to retain community coalitions and service venues throughout the intervention. Some professionals working in service venues will participate in data collection through surveys, interviews, and so on concerning the opioid-related services currently being offered, their ability to offer new services, attitudes that might affect the success of service offerings, and the cost of offering services. If a participating professional decides to no longer participate or is no longer working at the venue, efforts will be made to understand and document the reasons for discontinuation. Most of the desired information is not about the specific participant but about the venue where he or she works. In such situations, a replacement respondent will be sought.

8. INSTRUMENTS AND PROCEDURES

8.1 Instruments

This section describes the instruments and approaches that will be used to collect data for the HEALing Communities Study (HCS). Data collection will include surveys and interviews with coalition and Community Advisory Board (CAB) members, key stakeholders, and organizational representatives (e.g., in jails, emergency departments, and recovery support programs) to measure outcomes. Research staff will conduct focus groups, individual interviews, and workshops to test and refine messages for the communication campaign. Coalition and CAB members will provide cost information on the implementation of the Communities That HEAL (CTH) to estimate cost-effectiveness. Finally, we will collect secondary data via a Landscape Analysis to characterize the communities and contextualize the study findings. See Table 9 for a list of instruments linked to informed consent.

Table 9 provides a list of the instruments, guides, and templates that will be used to collect quantitative and qualitative data. The table links the instruments to their associated informed consent forms (ICFs). For coalition and CAB members, who will be interviewed more than once, we will use a general written consent form that seeks consent for all survey activities (from baseline through study end). Data collections that are conducted in multiple modes (e.g., web and telephone) have multiple ICFs tailored to the mode of administration.

Table 10 consists of one table for each data collection instrument. Each table provides detailed instrument summaries that describe how each data collection will be conducted. Among other items, these tables indicate the data collection's purpose, respondent, mode, use of incentives, and frequency.

Table 9: Instrument name and associated informed consent form name

Instrument Name	Informed Consent Form Name
**Brandeis Payer Pre-Interview Survey & Interview Guide**	HCS_ICF_MA_Brandeis Payer Pre-Interview Survey & Interview Guide (In-Person)<br>HCS_ICF_MA_Brandeis Payer Pre-Interview Survey & Interview Guide (Verbal)<br>HCS_ICF_MA_Brandeis Payer Pre-Interview Survey & Interview Guide (Web)
**Campaign Evaluation Questionnaire**	HCS_ICF_Master_Campaign Evaluation Questionnaire (Web)
**Coalition Meeting Minute Template**	N/A
**Communication Design Workshop Guide**	HCS_ICF_Master_Communication Design Workshop (In Person)
**Communications Campaign Costing**	HCS_ICF_Master_Cost Template Information (Verbal)<br>HCS_ICF_Master_Cost Template Information (Web)<br>HCS_ICF_MA NY & KY_General Consent for Coalitions (In Person)<br>HCS_ICF_OH_General Consent for CAB and Coalitions (In Person)
**Community Advisory Board (CAB) Demographics Survey**	N/A
**Community Advisory Board (CAB) Member Interview Guide**	HCS_ICF_NY&MA_CAB Member Interview Guide (Verbal)
**Community Coalition Member and Key Stakeholder Semi-Structured Qualitative Interview Guide (Baseline and Follow-Up)**	HCS_ICF_Master_Community Coalition Member and Key Stakeholder Semi-Structured Qualitative Interview Guide (In Person) – *used from Nov. 2019 – Jan. 2020*<br>HCS_ICF_Master_Community Coalition Member and Key Stakeholder Semi-Structured Qualitative Interview Guide (Verbal) – *used from Nov. 2019 – Jan. 2020*<br>HCS_ICF_MA NY & KY_General Consent for Coalitions (In Person)<br>HCS_ICF_OH_General Consent for CAB and Coalitions (In Person)<br>HCS_ICF_Master_Follow-Up Community Coalition Member and Key Stakeholder Semi-Structured Qualitative Interview Guide (Verbal)

Instrument Name	Informed Consent Form Name
**Community Coalition and Key Stakeholder Survey (Baseline and Follow-Up)**	HCS_ICF_Master_Community Coalition Member and Key Stakeholder Survey (In Person) – *used from Nov. 2019 – Jan. 2020*<br>HCS_ICF_Master_Community Coalition Member and Key Stakeholder Survey (Verbal) – *used from Nov. 2019 – Jan. 2020*<br>HCS_ICF_Master_Community Coalition Member and Key Stakeholder Survey (Web) – *used from Nov. 2019 – Jan. 2020*<br>HCS_ICF_MA NY & KY_General Consent for Coalitions (In Person)<br>HCS_ICF_OH_General Consent for CAB and Coalitions (In Person)<br>HCS_ICF_Master_KY MA OH_Follow-Up Community Coalition Member and Key Stakeholder Survey (In-Person)<br>HCS_ICF_Master_NY_Follow-Up Community Coalition Member and Key Stakeholder Survey (In-Person)<br>HCS_ICF_Master_KY MA OH_Follow-Up Community Coalition Member and Key Stakeholder Survey (Verbal)<br>HCS_ICF_Master_NY_Follow-Up Community Coalition Member and Key Stakeholder Survey (Verbal)<br>HCS_ICF_Master_KY MA OH_Follow-Up Community Coalition Member and Key Stakeholder Survey (Web)<br>HCS_ICF_Master_NY_Follow-Up Community Coalition Member and Key Stakeholder Survey (Web)
**Community Engagement Costing (Coalition Meetings)**	HCS_ICF_Master_Cost Template Information (Verbal)<br>HCS_ICF_Master_Cost Template Information (Web)<br>HCS_ICF_MA NY & KY_General Consent for Coalitions (In Person)<br>HCS_ICF_OH_General Consent for CAB and Coalitions (In Person)
**Community Engagement Facilitator – Community Assessment Tool (CAT)**	N/A
**Costing the Evidence Based Practices Interview Guide**	HCS_ICF_Master_KY MA NY_Template for Costing the Evidence Based Practices (Verbal)
**CTH Milestone and Benchmark Checklist**	N/A
**Demographic Form**	HCS_ICF_Master_Community Coalition Member and Key Stakeholder Semi-Structured Qualitative Interview Guide (In Person)<br>HCS_ICF_Master_Community Coalition Member and Key Stakeholder Semi-Structured Qualitative Interview Guide (Verbal)

Instrument Name	Informed Consent Form Name
	HCS_ICF_Master_Community Coalition Member and Key Stakeholder Survey (In-Person)<br>HCS_ICF_Master_Community Coalition Member and Key Stakeholder Survey (Web)<br>HCS_ICF_Master_Community Coalition Member and Key Stakeholder Survey (Verbal)
**Document Review Guide**	N/A
**Ethnographic Guide for Field Notes**	N/A
**Focus Group CAB, Coalition, and Partner Organizations Guide**	HCS_ICF_Master_Focus Group with CAB, Coalition and Partner Organization (In Person)<br>HCS_ICF_Master_Focus Group with CAB, Coalition and Partner Organization (Verbal)
**Focus Group for Persons with OUD**	HCS_ICF_Master Focus Group Person with OUD (In Person)
**Focus Group Testing of Launch Messages**	HCS_ICF_Master_Focus Group Testing of Launch Messages (In Person)<br>HCS_ICF_Master_Focus Group Testing of Launch Messages (Verbal)
**General Template Costing the Community Engagement Process (CAB Members)**	HCS_ICF_Master_Cost Template Information (Verbal)<br>HCS_ICF_Master_Cost Template Information (Web)<br>HCS_ICF_OH_General Consent for CAB and Coalitions (In Person)<br>HCS_ICF_MA NY & KY General Consent for CABs (In Person)
**Grant Writing Needs Assessment**	N/A
**Group Model Building**	HCS_ICF_MA NY & KY_General Consent for Coalitions (In Person)<br>HCS_ICF_MA NY & KY General Consent for CABs (In Person)
**Group Model Building (GMB) Session Feedback Form**	N/A
**HEALing Communities CAB Member Survey**	HCS_ICF_NY&MA_HEALing Communities CAB Survey (In Person)<br>HCS_ICF_NY&MA_HEALing Communities CAB Survey (Verbal)<br>HCS_ICF_NY&MA_HEALing Communities CAB Survey (Web)
**HCS Annual Jail Survey**	HCS_ICF_Master_Annual Jail Survey (In-Person)<br>HCS_ICF_Master_Annual Jail Survey (Verbal)

Instrument Name	Informed Consent Form Name
	HCS_ICF_Master_Annual Jail Survey (Web)
**HCS Community Data Dashboards Survey & Portal Group Interview Guide**	HCS_ICF_Master_HCS Community Data Dashboards Survey & Portal Group Interview Guide (Verbal)
**HCS Community Grant Documentation**	N/A
**HCS Staff Activity Costing Instrument**	N/A
**HCS Staff Tracker**	N/A
Interview Guide Message Testing	HCS_ICF_Master_Interview Guide Message Testing Hcare Providers Comm Leaders Persons wOUD and Family Mbrs (Verbal)
**Interview Guide Message Testing Persons with OUD**	HCS_ICF_Master_Interview Guide Message Testing Hcare Providers Comm Leaders Persons wOUD and Family Mbrs (Verbal)
**Landscape Analysis 1 (Wave 1)/Landscape Analysis (Wave 2)**	N/A
**Landscape Analysis 2 (Wave 1)/Landscape Analysis Organizational Questionnaire (Wave 2)**	HCS_ICF_KY&OH_Landscape Analysis 2 for Data Collection (Verbal)<br>HCS_ICF_KY&OH_Landscape Analysis 2 for Data Collection (Web)<br>HCS_ICF_KY&OH_Landscape Analysis 2 for Data Collection (In Person)<br>HCS_ICF_KY_Landscape Analysis 2 for Data Collection-Pharmacy Module (Verbal)
**Learning Collaborative Evaluation Survey**	N/A
**Medical Examiner Survey**	HCS_ICF_Master_NY Medical Examiner Survey (Web)
**Medication Disposal Drop Box Sustainability Interview Guide**	HCS_ICF_Master_KY Medication Disposal Program Pharmacy Interviews (Verbal)
**Mobile MOUD Interview Guide**	HCS_ICF_Master_Mobile MOUD Interview Guide for MA, NY & OH (Verbal)
**Monthly Coalition Work Group or Subcommittee Meeting Log**	N/A
**MOUD Organization Interview Guide**	HCS_ICF_Master_KY MOUD Organization Interview Guide (Verbal)
**Municipal Drug Policies Interview Guide**	HCS-ICF_Master_MA Municipal Policies Interview Guide (Verbal)
**ORCCA Tracker (ORCCAT)**	N/A
**PARTNER Tool**	HCS_ICF_Master_OH Partner Tool for Wave 2 (Verbal)

Instrument Name	Informed Consent Form Name
	HCS_ICF_Master_OH Partner Tool for Wave 2 (Web)
**Pharmacy Study Interview Guide**	HCS_ICF_Master_NY Pharmacy Study Interview Guide for Community Member (Verbal)<br>HCS_ICF_Master_NY Pharmacy Study Interview Guide for Pharmacist (Verbal)
**Pharmacy Study Survey**	HCS_ICF_Master_NY Pharmacy Study Survey PHARMACIST ONLY (Verbal)<br>HCS_ICF_Master_NY Pharmacy Study Survey PHARMACIST ONLY (Web)<br>HCS_ICF_Master_NY Pharmacy Study Survey PHARMACY ONLY (Verbal)<br>HCS_ICF_Master_NY Pharmacy Study Survey PHARMACY ONLY (Web)
**Photovoice Focus Group Interview Guide & Demographics Survey**	HCS_ICF_Master_Photovoice Focus Group for KY, MA & OH (In-Person)<br>HCS_ICF_Master_Photovoice Focus Group for KY, MA & OH (Verbal)
**Policy Community Report**	N/A
**Policy Environmental Scan**	N/A
**Post Coalition Meeting Feedback Form**	N/A
**Qualitative Assessment Form for Campaign 4**	N/A
**Race and Ethnicity Data Collection Readiness Survey**	HCS_ICF_Master_NY Race and Ethnicity Data Collection Readiness Survey (In-Person)<br>HCS_ICF_Master_NY Race and Ethnicity Data Collection Readiness Survey (Verbal)<br>HCS_ICF_Master_NY Race and Ethnicity Data Collection Readiness Survey (Web)
**Reach Tracker**	N/A
**State Grant Funding**	HCS_ICF_Master_State Grant Funding (Verbal)
**Sustainability Partner Organization Interview Guide**	HCS_ICF_Master_Sustainability Partner Organization Interview Guide (Verbal)
**Toxicology Survey for Labs**	HCS_ICF_Master_Toxicology Survey for Labs (Verbal)<br>HCS_ICF_Master_Toxicology Survey for Labs (Web)
**Toxicology Survey for Medical Examiners & Coroners**	HCS_ICF_Master_KY MA OH Toxicology Survey for Medical Examiners & Coroners (Verbal)<br>HCS_ICF_Master_KY MA OH Toxicology Survey for Medical Examiners & Coroners (Web)

Instrument Name	Informed Consent Form Name
**Training and Technical Assistance Tracking (TTAT) Form**	N/A

Table 10: Instrument Summaries

Brandeis Payer Pre-Interview Survey & Interview Guide		
**Main Study Data Collection**		
1	**Purpose**	To gather information about the extent to which payers currently fund or otherwise encourage activities that communities may adopt as part of the HCS intervention, such as medications for opioid use disorder (MOUD), distribution of naloxone, or initiatives in schools, pharmacies and law enforcement settings.
2	**Respondent**	Officials at MassHealth (the state Medicaid Program) and at commercial insurers that serve Massachusetts customers.
3	**How will respondents be selected?**	Respondents will include officials from 13 commercial insurers that were identified by the Massachusetts Division of Insurance. Respondents may also include officials from other organizations that manage substance use disorder treatment under subcontracts with these insurers.
4	**Sample size**	Up to 20
5	**Repeated (Y/N)**	Yes
	**If yes: frequency (e.g., annually, monthly)**	Annually
6	**Mode (telephone, web, in person)**	The Brandeis Payer Pre-Interview Survey will be collected via the web. The Brandeis Payer Interview Guide will be administered either over the telephone or in-person.
7	**Self-administered or interviewer administered**	The Brandeis Payer Pre-Interview Survey will be self-administered, and the Brandeis Payer Interview Guide will be interviewer-administered.
8	**Informed consent (verbal, written, digital)**	Verbal, written, or digital
9	**Incentives (Y/N)**	No
	**If yes: incentive type (e.g., gift card, cash, check)**	N/A
10	**Seeking Waiver of Documentation of Informed Consent (Y/N)**	Yes
11	**Cross-site or site specific (specify site)**	Site specific (Massachusetts)

12	**Data collection platform (e.g., paper, REDCap, video)**	The Brandeis Payer Pre-Interview Survey will be administered via REDCap. The Brandeis Payer Interview Guide will be audio recorded. All recordings will be password protected and archived on a secure server. All transcripts will be de-identified.
13	**Audio-recording (Y/N)**	Yes
14	**Other respondent-facing material (e.g., email invitation to participate) (specify)**	Email invitation to participate

**Campaign Evaluation Questionnaire**		
**Main Study Data Collection**		
1	**Purpose**	Survey data for evaluation of the health communication campaigns within and across all four HCS sites
2	**Respondent**	Community members
3	**How will respondents be selected?**	Via targeted Facebook advertisements seeking respondents in their respective communities to take the survey evaluating the campaign
4	**Sample size**	In total, across all 4 sites and all campaigns of the HCS, approximately 30,000 surveys will be completed
5	**Repeated (Y/N)**	Yes
	**If yes: frequency (e.g., annually, monthly)**	3 times for each campaign (as a pre-test, intermediate, and post-test) for 6 campaigns (Wave 1 and Wave 2) plus additional baseline and post-tests * 4 sites = 84 times repeated
6	**Mode (telephone, web, in person)**	Web
7	**Self-administered or interviewer administered**	Self-administered. Interested respondents will click a Facebook advertisement that will take them to the REDCap survey where they will read and consent to participate, then they will begin the survey and complete it online via REDCap
8	**Informed consent (verbal, written, digital)**	Digital
9	**Incentives (Y/N)**	Yes
	**If yes: incentive type (e.g., gift card, cash, check)**	A raffle of a $100 Amazon e-gift card for each survey time point for each county or community participating in the survey at that time
10	**Seeking Waiver of Documentation of Informed Consent (Y/N)**	Yes
11	**Cross-site or site specific (specify site)**	Cross-site

12	**Data collection platform (e.g., paper, REDCap, video)**	REDCap
13	**Audio-recording (Y/N)**	No
14	**Other respondent-facing material (e.g., email invitation to participate) (specify)**	N/A

**Coalition Meeting Minute Template**		
**Main Study Data Collection**		
1	**Purpose**	To assess adherence and quality of implementing the CTH intervention
2	**Respondent**	HCS research staff
3	**How will respondents be selected?**	HCS research staff hired by the study
4	**Sample size**	67
5	**Repeated (Y/N)**	Yes
	**If yes: frequency (e.g., annually, monthly)**	Will vary; dependent on how often the coalition meets
6	**Mode (telephone, web, in person)**	In person or web
7	**Self-administered or interviewer administered**	Self-administered
8	**Informed consent (verbal, written, digital)**	N/A
9	**Incentives (Y/N)**	No
	**If yes: incentive type (e.g., gift card, cash, check)**	N/A
10	**Seeking Waiver of Documentation of Informed Consent (Y/N)**	No
11	**Cross-site or site specific (specify site)**	Cross-site
12	**Data collection platform (e.g., REDCap, video)**	REDCap
13	**Audio-recording (Y/N)**	No
14	**Other respondent-facing material (e.g., email invitation to participate) (specify)**	N/A

**Communication Design Workshop Guide**		
**Main Study Data Collection**		
1	**Purpose**	To understand how people in the community perceive the opioid problem and potential solutions and to test messages about naloxone, OUD, MOUD, and stigma

2	**Respondent**	Respondents will be members of the community and people who work in organizations that offer services that address the opioid crisis in that community
3	**How will respondents be selected?**	Potential participants will be solicited at coalition meetings and through public notices on social media and print outlets. Coalition members will help identify respondents from partner or implementation agencies in the community.
4	**Sample size**	Total up to 1,000 across 33 Wave 1 communities
5	**Repeated (Y/N)**	Yes
	**If yes: frequency (e.g., annually, monthly)**	These workshops may be repeated for subsequent communication campaigns
6	**Mode (telephone, web, in person)**	In person
7	**Self-administered or interviewer administered**	Interviewer administered
8	**Informed consent (verbal, written, digital)**	Written
9	**Incentives (Y/N)**	No
	**If yes: incentive type (e.g., gift card, cash, check)**	N/A
10	**Seeking Waiver of Documentation of Informed Consent (Y/N)**	No
11	**Cross-site or site specific (specify site)**	Cross-site
12	**Data collection platform (e.g., REDCap, video)**	Observers’ notes, participant-generated content to exercises
13	**Audio-recording (Y/N)**	No
14	**Other respondent-facing material (e.g., email invitation to participate) (specify)**	Email invitation, confirmation, and meeting logistics information

**Communications Campaign Costing**		
**Main Study Data Collection**		
1	**Purpose**	To estimate the cost of the communication campaign in the preparation and planning phases for health economic analysis
2	**Respondent**	RS staff and community members assigned to work on the communication campaign
3	**How will respondents be selected?**	RSs and the DCC will identify respondents who are involved with the communication campaign

Communications Campaign Costing		
4	**Sample size**	N=221−489<br>(3–7 coalition members per community) × (67 communities) + 20 DCC and RS staff
5	**Repeated (Y/N)**	Yes
	**If yes: frequency (e.g., annually, monthly)**	Monthly
6	**Mode (telephone, web, in person)**	In person or by telephone
7	**Self-administered or interviewer administered**	Interviewer administered
8	**Informed consent (verbal, written, digital)**	General written consent or verbal consent for community members<br>No consent for study staff
9	**Incentives (Y/N)**	No
	**If yes: incentive type (e.g., gift card, cash, check)**	N/A
10	**Seeking Waiver of Documentation of Informed Consent (Y/N)**	Yes
11	**Cross-site or site specific (specify site)**	Cross-site
12	**Data collection platform (e.g., REDCap, video)**	REDCap
13	**Audio-recording (Y/N)**	No
14	**Other respondent-facing material (e.g., email invitation to participate) (specify)**	There will be an email or telephone-based invitation to participate

Community Advisory Board (CAB) Demographics Survey		
Main Study Data Collection		
1	**Purpose**	To capture demographic characteristics of the Community Advisory Board (CAB) for Wave 1 and Wave 2 communities.
2	**Respondent**	Community Advisory Board (CAB) members
3	**How will respondents be selected?**	Respondents are members of the state-specific Community Advisory Boards. Survey responses will be anonymous.
4	**Sample size**	Up to 175 per data collection period
5	**Repeated (Y/N)**	Yes
	**If yes: frequency (e.g., annually, monthly)**	Annually

Community Advisory Board (CAB) Demographics Survey		
6	**Mode (telephone, web, in person)**	Web
7	**Self-administered or interviewer administered**	Self-administered
8	**Informed consent (verbal, written, digital)**	N/A
9	**Incentives (Y/N)**	No
	**If yes: incentive type (e.g., gift card, cash, check)**	N/A
10	**Seeking Waiver of Documentation of Informed Consent (Y/N)**	No
11	**Cross-site or site specific (specify site)**	Cross-site
12	**Data collection platform (e.g., REDCap, video)**	REDCap
13	**Audio-recording (Y/N)**	No
14	**Other respondent-facing material (e.g., email invitation to participate) (specify)**	N/A

Community Advisory Board (CAB) Member Interview Guide		
**Main Study Data Collection**		
1	**Purpose**	To measure CAB members' perspectives on the role of the CAB in the study and the structure and processes of the CAB
2	**Respondent**	CAB members
3	**How will respondents be selected?**	Potential participants will be identified based on CAB membership
4	**Sample size**	80 (up to 30 in Massachusetts; up to 50 in New York)
5	**Repeated (Y/N)**	Yes
	**If yes: frequency (e.g., annually, monthly)**	Annually
6	**Mode (telephone, web, in person)**	Telephone
7	**Self-administered or interviewer administered**	Interviewer administered
8	**Informed consent (verbal, written, digital)**	Verbal
9	**Incentives (Y/N)**	Yes

Community Advisory Board (CAB) Member Interview Guide		
	**If yes: incentive type (e.g., gift card, cash, check)**	The participant will receive $75 cash, check, or gift card after completing the interview.
10	**Seeking Waiver of Documentation of Informed Consent (Y/N)**	Yes
11	**Cross-site or site specific (specify site)**	Site specific (Massachusetts and New York)
12	**Data collection platform (e.g., REDCap, video)**	Audio-recording. All recordings will be password protected and archived on a secure server. All transcripts will be de-identified.
13	**Audio-recording (Y/N)**	Yes
14	**Other respondent-facing material (e.g., email invitation to participate) (specify)**	See *HCS Email Invitation and Follow Up Reminders for CAB Survey & Interview*

Community Coalition Member and Key Stakeholder Semi-Structured Qualitative Interview Guide (for Baseline and Follow-Up Interview)		
**Main Study Data Collection**		
1	**Purpose**	To qualitatively measure coalition members' and key stakeholders' perspectives on the opioid epidemic in their communities, current community responses to the opioid epidemic, and factors in the internal context (i.e., coalition perspectives and characteristics) and external context (i.e., community perspectives and characteristics) that may facilitate or impede the successful implementation of HCS efforts to reduce opioid-related mortality by 40%
2	**Respondent**	Coalition members and key stakeholders in Wave 1 and Wave 2 HCS communities
3	**How will respondents be selected?**	Potential participants will be identified based on membership of a given community coalition or key stakeholders for a given community (if no coalition exists). A purposive sampling strategy will be used, with the goals of including coalition leadership and maximizing variability and diversity. Initial contact with potential participants may be made in person (e.g., at a regularly scheduled meeting), by email, or by telephone to describe the purpose of the study, to describe compensation associated with participating, and to gauge potential interest in participating.

Community Coalition Member and Key Stakeholder Semi-Structured Qualitative Interview Guide (for Baseline and Follow-Up Interview)		
4	**Sample size**	4–12 interviews per community
5	**Repeated (Y/N)**	Yes
	**If yes: frequency (e.g., annually, monthly)**	Three additional follow-up time points (approximately 15 months, 30 months, and 42 months)
6	**Mode (telephone, web, in person)**	Telephone/video conference or in-person for baseline interview; telephone/video conference for follow-up interview
7	**Self-administered or interviewer administered**	Interviewer administered
8	**Informed consent (verbal, written, digital)**	Verbal or written for baseline interview; verbal for follow-up interview
9	**Incentives (Y/N)**	Yes (for KY, MA, NY); OH will not offer an incentive.
	**If yes: incentive type (e.g., gift card, cash, check)**	$50 cash, check, or gift card
10	**Seeking Waiver of Documentation of Informed Consent (Y/N)**	Yes
11	**Cross-site or site specific (specify site)**	Cross-site
12	**Data collection platform (e.g., REDCap, video)**	Telephone or video conferencing
13	**Audio-recording (Y/N)**	Yes
14	**Other respondent-facing material (e.g., email invitation to participate) (specify)**	See *HCS Follow-Up Community Coalition Member and Key Stakeholder Interview Guide Subject Facing Materials*

Community Coalition and Key Stakeholder Survey (for Baseline and Follow-Up Interview)		
**Main Study Data Collection**		
1	**Purpose**	To measure coalition members' and key stakeholders' perspectives on factors in the internal context (e.g., coalition characteristics, coalition functioning, coalition readiness to promote expansion of MOUD and OEND) and external context (e.g., community need and stigma) that may facilitate or impede the successful implementation of HCS efforts to reduce opioid-related mortality by 40%
2	**Respondent**	Coalition members and key stakeholders
3	**How will respondents be selected?**	Potential participants will be identified based on coalition membership and rosters (if a coalition exists). If a coalition does not exist, potential

Community Coalition and Key Stakeholder Survey<br>(for Baseline and Follow-Up Interview)		
		participants will also be identified through the state department of health, appropriate county official, or other key contacts in the state or county. Participants will be selected to ensure diversity of stakeholder types. Additional participants may be identified using a snowball sampling strategy based on the PARTNER section of the survey.
4	**Sample size**	Approximately 2,010 coalition members and key stakeholders across communities (30 per community)
5	**Repeated (Y/N)**	Yes
	**If yes: frequency (e.g., annually, monthly)**	Three additional follow-up time points (approximately 15 months, 30 months, and 42 months
6	**Mode (telephone, web, in person)**	Web, telephone, in person
7	**Self-administered or interviewer administered**	Self-administered or interviewer administered
8	**Informed consent (verbal, written, digital)**	Digital, verbal, or written
9	**Incentives (Y/N)**	Yes (for KY, MA, NY); OH will not offer an incentive.
	**If yes: incentive type (e.g., gift card, cash, check)**	$50 cash, check, or gift card
10	**Seeking Waiver of Documentation of Informed Consent (Y/N)**	Yes
11	**Cross-site or site specific (specify site)**	Cross-site
12	**Data collection platform (e.g., REDCap, video)**	REDCap or hard copy/paper version of the survey
13	**Audio-recording (Y/N)**	No
14	**Other respondent-facing material (e.g., email invitation to participate) (specify)**	See *HCS Follow-Up Community Coalition Member and Key Stakeholder Survey Subject Facing Materials*

Community Engagement Costing (Coalition Meetings)		
Main Study Data Collection		
1	**Purpose**	To estimate the cost of community coalition meetings and the cost of community coalition member activities in support of the CE process
2	**Respondent**	Community coalition members

Community Engagement Costing (Coalition Meetings)		
3	**How will respondents be selected?**	All coalition members attending coalition meetings will be surveyed
4	**Sample size**	1,005–1,340 coalition members<br>(15–20 coalition members per community) * (67 communities)
5	**Repeated (Y/N)**	Yes
	**If yes: frequency (e.g., annually, monthly)**	Monthly (or when coalition meetings are held)
6	**Mode (telephone, web, in person)**	In person, web, or by telephone
7	**Self-administered or interviewer administered**	Interviewer administered
8	**Informed consent (verbal, written, digital)**	General written, verbal, or digital consent for coalition members
9	**Incentives (Y/N)**	No
	**If yes: incentive type (e.g., gift card, cash, check)**	N/A
10	**Seeking Waiver of Documentation of Informed Consent (Y/N)**	Yes
11	**Cross-site or site specific (specify site)**	Cross-site
12	**Data collection platform (e.g., REDCap, video)**	REDCap
13	**Audio-recording (Y/N)**	No
14	**Other respondent-facing material (e.g., email invitation to participate) (specify)**	Email invitation and follow-up reminders

Community Engagement Facilitator - Community Assessment Tool (CAT)		
Main Study Data Collection		
1	**Purpose**	The Ohio research site will develop a community genogram model to track connections among organizations, agencies, and providers involved in relevant HCS work. As part of this process, the Community Engagement (CE) Facilitators will use the CE Facilitator - Community Assessment Tool (CAT) to detail their process and work in Ohio's participating HCS communities.
2	**Respondent**	Ohio HCS research staff (Community Engagement Facilitators)
3	**How will respondents be selected?**	HCS research staff hired by the study

Community Engagement Facilitator - Community Assessment Tool (CAT)		
4	**Sample size**	18
5	**Repeated (Y/N)**	Yes
	**If yes: frequency (e.g., annually, monthly)**	As needed
6	**Mode (telephone, web, in person)**	The CE Facilitator's interactions with coalition leaders/community members can occur face-to-face, by telephone or email.
7	**Self-administered or interviewer administered**	Self-administered
8	**Informed consent (verbal, written, digital)**	N/A
9	**Incentives (Y/N)**	No
	**If yes: incentive type (e.g., gift card, cash, check)**	N/A
10	**Seeking Waiver of Documentation of Informed Consent (Y/N)**	No
11	**Cross-site or site specific (specify site)**	Site specific (Ohio)
12	**Data collection platform (e.g., REDCap, video)**	REDCap
13	**Audio-recording (Y/N)**	No
14	**Other respondent-facing material (e.g., email invitation to participate) (specify)**	N/A

Costing the Evidence Based Practices Interview Guide		
Main Study Data Collection		
1	**Purpose**	To understand the costs of implementing the Evidence-Based Practice (EBP) Strategies. Specifically, the interviews will gather information about start-up and operational costs, such as time spent on activities required to stand up the EBP strategies and provide ongoing services, as well as additional resources that are required to implement and sustain each strategy.
2	**Respondent**	Key Informants at implementing organizations in the HCS communities.

3	**How will respondents be selected?**	The HCS research teams will identify the key informant(s) at each institution most likely to have knowledge about start-up and operational costs.
4	**Sample size**	Up to 90 (up to 30 at each research site)
5	**Repeated (Y/N)**	Yes
	**If yes: frequency (e.g., annually, monthly)**	Annually
6	**Mode (telephone, web, in person)**	Telephone or Videoconference
7	**Self-administered or interviewer administered**	Interviewer Administered
8	**Informed consent (verbal, written, digital)**	Verbal
9	**Incentives (Y/N)**	No
	**If yes: incentive type (e.g., gift card, cash, check)**	N/A
10	**Seeking Waiver of Documentation of Informed Consent (Y/N)**	Yes
11	**Cross-site or site specific (specify site)**	Site Specific (Kentucky, Massachusetts, New York)
12	**Data collection platform (e.g., REDCap, video)**	REDCap
13	**Audio-recording (Y/N)**	No
14	**Other respondent-facing material (e.g., email invitation to participate) (specify)**	Yes, see *HCS Template for Costing the Evidence Based Practices Subject Facing Materials (KY, MA, NY)*

**CTH Milestone and Benchmark Checklist**		
**Main Study Data Collection**		
1	**Purpose**	To assess adherence to the CTH CE SOP
2	**Respondent**	HCS research staff
3	**How will respondents be selected?**	HCS CE staff hired by the study
4	**Sample size**	67
5	**Repeated (Y/N)**	Yes

CTH Milestone and Benchmark Checklist		
	**If yes: frequency (e.g., annually, monthly)**	Monthly basis for the first 6 months, then quarterly until all six phase activities are completed
6	**Mode (telephone, web, in-person)**	Web
7	**Self-administered or interviewer administered**	Self-administered
8	**Informed consent (verbal, written, digital)**	N/A
9	**Incentives (Y/N)**	No
	**If yes: incentive type (e.g., gift card, cash, check)**	N/A
10	**Seeking Waiver of Documentation of Informed Consent (Y/N)**	No
11	**Cross-site or site specific (specify site)**	Cross-site
12	**Data collection platform (e.g., REDCap, video)**	REDCap
13	**Audio-recording (Y/N)**	No
14	**Other respondent-facing material (e.g., email invitation to participate) (specify)**	N/A

Document Review Guide		
**Main Study Data Collection**		
1	**Purpose**	To assess adherence to the CTH CE SOP and the quality of implementing the CTH intervention
2	**Respondent**	HCS research staff
3	**How will respondents be selected?**	HCS research staff hired by the study
4	**Sample size**	67
5	**Repeated (Y/N)**	Yes
	**If yes: frequency (e.g., annually, monthly)**	As needed
6	**Mode (telephone, web, in person)**	Web
7	**Self-administered or interviewer administered**	Self-administered
8	**Informed consent (verbal, written, digital)**	N/A
9	**Incentives (Y/N)**	No
	**If yes: incentive type (e.g., gift card, cash, check)**	N/A

Document Review Guide		
10	**Seeking Waiver of Documentation of Informed Consent (Y/N)**	No
11	**Cross-site or site specific (specify site)**	Site specific (Massachusetts and New York)
12	**Data collection platform (e.g., REDCap, video)**	REDCap
13	**Audio-recording (Y/N)**	No
14	**Other respondent-facing material (e.g., email invitation to participate) (specify)**	N/A

Ethnographic Guide for Field Notes		
Main Study Data Collection		
1	**Purpose**	To observe coalition meetings for quality and adherence of delivering the CTH intervention
2	**Respondent**	HCS research staff
3	**How will respondents be selected?**	HCS research staff from the implementation science team
4	**Sample size**	To be determined
5	**Repeated (Y/N)**	Yes
	**If yes: frequency (e.g., annually, monthly)**	Will vary; dependent on how often the coalition meets
6	**Mode (telephone, web, in person)**	In person
7	**Self-administered or interviewer administered**	Self-administered
8	**Informed consent (verbal, written, digital)**	N/A
9	**Incentives (Y/N)**	No
	**If yes: incentive type (e.g., gift card, cash, check)**	N/A
10	**Seeking Waiver of Documentation of Informed Consent (Y/N)**	No
11	**Cross-site or site specific (specify site)**	Site specific (Massachusetts and New York)
12	**Data collection platform (e.g., REDCap, video)**	Data from paper forms will be entered into REDCap
13	**Audio-recording (Y/N)**	No
14	**Other respondent-facing material (e.g., email invitation to participate) (specify)**	N/A

Focus Group CAB, Coalition, and Partner Organizations Guide		
**Main Study Data Collection**		
1	**Purpose**	To understand how people who are involved in addressing various aspects of the local community response to the opioid crisis respond to messages about naloxone, OUD, MOUD, and stigma
2	**Respondent**	Coalition members, CAB members, and senior staff from local agencies implementing programs and services related to naloxone, OUD, MOUD, and stigma reduction
3	**How will respondents be selected?**	Potential participants will be solicited at coalition and CAB meetings. These coalition and CAB members will help identify respondents from partner or implementation agencies in the community.
4	**Sample size**	Total up to 400 across 33 Wave 1 communities
5	**Repeated (Y/N)**	Yes
	**If yes: frequency (e.g., annually, monthly)**	These focus groups may be repeated for subsequent campaigns
6	**Mode (telephone, web, in person)**	In person or telephone
7	**Self-administered or interviewer administered**	Interviewer administered
8	**Informed consent (verbal, written, digital)**	Verbal or written
9	**Incentives (Y/N)**	Yes
	**If yes: incentive type (e.g., gift card, cash, check)**	$50–$75 gift card to be determined by the RS
10	**Seeking Waiver of Documentation of Informed Consent (Y/N)**	Yes
11	**Cross-site or site specific (specify site)**	Cross-site
12	**Data collection platform (e.g., REDCap, video)**	Audio-recording. Records will be maintained in a locked file cabinet and destroyed after analysis is complete.
13	**Audio-recording (Y/N)**	Yes
14	**Other respondent-facing material (e.g., email invitation to participate) (specify)**	Email invitation, confirmation, and meeting logistics

Focus Group for Persons with OUD		
Main Study Data Collection		
1	**Purpose**	Message testing to ensure relevance and lower risk of unintended effects of the communication campaign
2	**Respondent**	People who self-identify as having OUD or using injection opioids
3	**How will respondents be selected?**	Potential participants will be recruited through print and social media advertisements. We anticipate that some recruitment will also occur through word of mouth in the social and professional networks of coalition members. Participants will be screened to be (1) between ages 18 and 75, (2) a resident of the town or county where the groups will take place, and (3) having a self-reported opioid use disorder or self-reporting use of injection opioids (i.e., heroin, fentanyl) or had an opioid overdose in the past year.
4	**Sample size**	N=300 across 33 communities; groups will have between 3 and 8 participants each
5	**Repeated (Y/N)**	Yes
	**If yes: frequency (e.g., annually, monthly)**	Yes, may be repeated for formative research in subsequent communication campaigns
6	**Mode (telephone, web, in person)**	In-person or web conferencing, to be determined by each RS
7	**Self-administered or interviewer administered**	Interviewer administered
8	**Informed consent (verbal, written, digital)**	Written
9	**Incentives (Y/N)**	Yes
	**If yes: incentive type (e.g., gift card, cash, check)**	$50–$75 gift card to be determined by the RS
10	**Seeking Waiver of Documentation of Informed Consent (Y/N)**	No
11	**Cross-site or site specific (specify site)**	Cross-site
12	**Data collection platform (e.g., REDCap, video)**	Audio-recording
13	**Audio-recording (Y/N)**	Yes. Recordings will be saved on secure project servers and deleted after analysis is complete.
14	**Other respondent-facing material (e.g., email invitation to participate) (specify)**	Email invitation, confirmation, and meeting logistics

Focus Group Testing of Launch Messages		
**Main Study Data Collection**		
1	**Purpose**	To understand how people who are aware of the opioid crisis in the community respond to messages about naloxone, OUD, MOUD, and stigma
2	**Respondent**	Residents in each community
3	**How will respondents be selected?**	Potential participants may be approached by a coalition member or will respond to an open solicitation via social media and newspaper items. They will be screened for (1) being between ages 18 and 75 and (2) being a resident of the town or county where the groups will take place. The RSs will attempt to have a broad mix of demographic backgrounds and people from different occupation groups in each group (e.g., not all working in the health sector but also representing connections to public safety, law enforcement, business/retail, farming, and other sectors).
4	**Sample size**	Total up to 400 across 33 Wave 1 communities
5	**Repeated (Y/N)**	Yes
	**If yes: frequency (e.g., annually, monthly)**	These focus groups may be repeated for subsequent communication campaigns but will not include the same participants
6	**Mode (telephone, web, in person)**	In person or telephone, to be determined by each RS
7	**Self-administered or interviewer administered**	Interviewer administered
8	**Informed consent (verbal, written, digital)**	Verbal or written
9	**Incentives (Y/N)**	Yes
	**If yes: incentive type (e.g., gift card, cash, check)**	$50–$75 gift card to be determined by the RS
10	**Seeking Waiver of Documentation of Informed Consent (Y/N)**	Yes
11	**Cross-site or site specific (specify site)**	Cross-site
12	**Data collection platform (e.g., REDCap, video)**	Audio-recording. These recordings will be saved on secure project servers and deleted after analysis is complete.
13	**Audio-recording (Y/N)**	Yes
14	**Other respondent-facing material (e.g., email invitation to participate) (specify)**	Email invitation, confirmation, and meeting logistics information

General Template Costing the Community Engagement Process (CAB Members)		
**Main Study Data Collection**		
1	**Purpose**	To collect the resources (e.g., labor, time, space, equipment) spent on CE activities by CAB members
2	**Respondent**	CAB members and other key informants
3	**How will respondents be selected?**	All CAB members attending the CAB meetings will be invited to participate
4	**Sample size**	60–80 CAB members (15–20 members per state * 4)
5	**Repeated (Y/N)**	Yes
	**If yes: frequency (e.g., annually, monthly)**	Monthly (or when CAB meetings are held)
6	**Mode (telephone, web, in person)**	In person, web, or telephone
7	**Self-administered or interviewer administered**	Interviewer administered
8	**Informed consent (verbal, written, digital)**	General written, verbal, or digital consent for CAB members
9	**Incentives (Y/N)**	No
	**If yes: incentive type (e.g., gift card, cash, check)**	N/A
10	**Seeking Waiver of Documentation of Informed Consent (Y/N)**	Yes
11	**Cross-site or site specific (specify site)**	Cross-site
12	**Data collection platform (e.g., REDCap, video)**	REDCap
13	**Audio-recording (Y/N)**	No
14	**Other respondent-facing material (e.g., email invitation to participate) (specify)**	Email invitation and follow-up reminders

Grant Writing Learning Collaborative Needs Assessment	
**Main Study Data Collection**	
**Purpose**	For HCS community members to provide input and feedback regarding their grant writing training needs.
**Respondent**	HCS community members planning to attend the grant writing Learning Collaborative
**How will respondents be selected?**	Respondents self-select to participate after being asked to provide input and feedback regarding their grant writing training needs.
**Sample size**	70
**Repeated (Y/N)**	No
**If yes: frequency (e.g., annually, monthly)**	N/A
**Mode (telephone, web, in person)**	Web
**Self-administered or interviewer administered**	Self-administered
**Informed consent (verbal, written, digital)**	N/A
**Incentives (Y/N)**	No
**If yes: incentive type (e.g., gift card, cash, check)**	N/A
**Seeking Waiver of Documentation of Informed Consent (Y/N)**	N/A
**Cross-site or site specific (specify site)**	Site-specific (Ohio)
**Data collection platform (e.g., REDCap, video)**	Qualtrics
**Audio-recording (Y/N)**	No
**Other respondent-facing material (e.g., email invitation to participate) (specify)**	N/A

Group Model Building		
**Main Study Data Collection**		
**1**	**Purpose**	To develop causal loop diagrams (CLDs) that elucidate the implementation challenges of opioid-related prevention and treatment strategies in each county. CLDs are learning and CE tools used for subsequent simulation modeling efforts and the development/adaptation of community actions.
**2**	**Respondent**	Key stakeholders drawn from community coalitions
**3**	**How will respondents be selected?**	Participants should be leaders or influencers from different sectors in each community. In small communities, key stakeholders could be the entire coalition if its membership is small. In communities with larger or multiple coalitions, key stakeholders will be selected based on their leadership role in different sectors.
**4**	**Sample size**	12–20 participants per community
**5**	**Repeated (Y/N)**	No
	**If yes: frequency (e.g., annually, monthly)**	N/A
**6**	**Mode (telephone, web, in person)**	In person
**7**	**Self-administered or interviewer administered**	Interviewer administered
**8**	**Informed consent (verbal, written, digital)**	General written consent for coalition members
**9**	**Incentives (Y/N)**	Yes
	**If yes: incentive type (e.g., gift card, cash, check)**	$50 gift card for key stakeholders in New York
**10**	**Seeking Waiver of Documentation of Informed Consent (Y/N)**	No
**11**	**Cross-site or site specific (specify site)**	Site specific (New York)
**12**	**Data collection platform (e.g., REDCap, video)**	Stella for visualizing the CLDs. Handwritten notes of discussion.
**13**	**Audio-recording (Y/N)**	No
**14**	**Other respondent-facing material (e.g., email invitation to participate) (specify)**	Email or telephone invitation and a brief describing the session

Group Model Building (GMB) Session Feedback Form		
Main Study Data Collection		
1	**Purpose**	To elicit anonymous feedback from Group Model Building Workshop participants to evaluate the quality of the workshop's facilitation, content, clarity, and perceived utility
2	**Respondent**	Workshop participants
3	**How will respondents be selected?**	Respondents will be participants from the Group Model Building Workshop
4	**Sample size**	To be determined
5	**Repeated (Y/N)**	Yes
	**If yes: frequency (e.g., annually, monthly)**	As needed
6	**Mode (telephone, web, in person)**	In person
7	**Self-administered or interviewer administered**	Self-administered
8	**Informed consent (verbal, written, digital)**	N/A
9	**Incentives (Y/N)**	No
	**If yes: incentive type (e.g., gift card, cash, check)**	N/A
10	**Seeking Waiver of Documentation of Informed Consent (Y/N)**	No
11	**Cross-site or site specific (specify site)**	Site specific (New York)
12	**Data collection platform (e.g., REDCap, video)**	Will be completed on paper, then data from forms will be entered into REDCap
13	**Audio-recording (Y/N)**	No
14	**Other respondent-facing material (e.g., email invitation to participate) (specify)**	N/A

HEALing Communities CAB Member Survey		
**Main Study Data Collection**		
**1**	**Purpose**	To understand CAB member structures and processes and CAB members' opinions about the CAB's work
**2**	**Respondent**	CAB members
**3**	**How will respondents be selected?**	Potential participants will be identified based on CAB membership
**4**	**Sample size**	80 (up to 30 in Massachusetts; up to 50 in New York)
**5**	**Repeated (Y/N)**	Yes
	**If yes: frequency (e.g., annually, monthly)**	Annually
**6**	**Mode (telephone, web, in person)**	In-person, web, or telephone
**7**	**Self-administered or interviewer administered**	Self-administered
**8**	**Informed consent (verbal, written, digital)**	Written, verbal, or digital
**9**	**Incentives (Y/N)**	Yes
	**If yes: incentive type (e.g., gift card, cash, check)**	Massachusetts will not provide an incentive; New York will provide $50 after the participant completes the survey.
**10**	**Seeking Waiver of Documentation of Informed Consent (Y/N)**	Yes
**11**	**Cross-site or site specific (specify site)**	Site specific (Massachusetts and New York)
**12**	**Data collection platform (e.g., REDCap, video)**	REDCap
**13**	**Audio-recording (Y/N)**	No
**14**	**Other respondent-facing material (e.g., email invitation to participate) (specify)**	Email invitation and follow-up reminders

HCS Annual Jail Survey		
**Main Study Data Collection**		
**1**	**Purpose**	To assess the provision of opioid-related services by jails associated with communities participating in the HCS
**2**	**Respondent**	Key informants
**3**	**How will respondents be selected?**	Key employees who are knowledgeable about opioid-related services provided by jails in communities participating in the HCS
**4**	**Sample size**	67–200 jails
**5**	**Repeated (Y/N)**	Yes
	**If yes: frequency (e.g., annually, monthly)**	Annually
**6**	**Mode (telephone, web, in person)**	Telephone, web, or in person
**7**	**Self-administered or interviewer administered**	Self-administered
**8**	**Informed consent (verbal, written, digital)**	Verbal, written, or digital
**9**	**Incentives (Y/N)**	No
	**If yes: incentive type (e.g., gift card, cash, check)**	N/A
**10**	**Seeking Waiver of Documentation of Informed Consent (Y/N)**	Yes
**11**	**Cross-site or site specific (specify site)**	Cross-site
**12**	**Data collection platform (e.g., REDCap, video)**	REDCap
**13**	**Audio-recording (Y/N)**	No
**14**	**Other respondent-facing material (e.g., email invitation to participate) (specify)**	Email/letter invitation and follow-up reminders via email/letter/phone scripts

HCS Community Data Dashboards Survey & Portal Group Interview Guide		
**Main Study Data Collection**		
1	**Purpose**	To gather information on how the HCS dashboards have been useful in CTH decision making, whether they are easy to use and understand, whether the HCS dashboards will continue to be used and for what purpose, and whether and in what capacity landscape data will continue to be used to populate the HCS dashboards.
2	**Respondent**	HCS community members and research staff
3	**How will respondents be selected?**	Respondents will have actively utilized the community portals and data dashboards as part of the HCS.
4	**Sample size**	Up to 130
5	**Repeated (Y/N)**	No
	**If yes: frequency (e.g., annually, monthly)**	N/A
6	**Mode (telephone, web, in person)**	The HCS Community Data Dashboards Survey will be collected via the web. The Portal Group Interview Guide will be collected via video conference.
7	**Self-administered or interviewer administered**	The HCS Community Data Dashboards Survey will be self-administered, and the Portal Group Interview Guide will be interviewer-administered.
8	**Informed consent (verbal, written, digital)**	Verbal
9	**Incentives (Y/N)**	No
	**If yes: incentive type (e.g., gift card, cash, check)**	N/A
10	**Seeking Waiver of Documentation of Informed Consent (Y/N)**	Yes
11	**Cross-site or site specific (specify site)**	Cross-Site
12	**Data collection platform (e.g., paper, REDCap, video)**	The HCS Community Data Dashboards Survey will be administered via REDCap. The Portal Group Interview Guide will be conducted via video conference.
13	**Audio-recording (Y/N)**	Yes (Portal Group Interview Guide)
14	**Other respondent-facing material (e.g., email invitation to participate) (specify)**	Yes, see *Subject Facing Materials for the HCS Portal Group Interview Guide*

HCS Community Grant Documentation		
**Main Study Data Collection**		
1	**Purpose**	To document grants or similarly funded projects in HCS communities related to the CTH implementation or EBPs.
2	**Respondent**	HCS staff will document information after receiving information from key informants/coalition members in HCS communities.
3	**How will respondents be selected?**	Additional grants and/or similarly funded projects will be discussed and identified during coalitions meetings or other meetings where coalition members are present. Key informants/coalition members will be asked to clarify basic details about the addition grant/funded project. HCS staff will document these details.
4	**Sample size**	Unknown; it is unknown how many grants and/or similarly funded projects are occurring in the HCS communities. This information will be collected for each HCS community.
5	**Repeated (Y/N)**	Yes
	**If yes: frequency (e.g., annually, monthly)**	Ongoing. These discussions will be a set agenda item during coalition meetings in three of the research sites (KY, MA, NY). The fourth research site (OH) will train the community engagement facilitators to stay up to date on grants and/or similarly funded projects in their HCS communities.
6	**Mode (telephone, web, in person)**	Interactions with key informants/coalition members can occur face-to-face, by telephone or email.
7	**Self-administered or interviewer administered**	Self-administered
8	**Informed consent (verbal, written, digital)**	N/A
9	**Incentives (Y/N)**	No
	**If yes: incentive type (e.g., gift card, cash, check)**	N/A
10	**Seeking Waiver of Documentation of Informed Consent (Y/N)**	No
11	**Cross-site or site specific (specify site)**	Cross-Site
12	**Data collection platform (e.g., REDCap, video)**	Word document
13	**Audio-recording (Y/N)**	No
14	**Other respondent-facing material (e.g., email invitation to participate) (specify)**	N/A

HCS Staff Activity Costing Instrument		
**Main Study Data Collection**		
**1**	**Purpose**	Collect the time spent by project staff facilitating the CTH intervention
**2**	**Respondent**	HCS project staff
**3**	**How will respondents be selected?**	Any staff funded in part by the HCS grants and who perform intervention activities that are not for the purposes of research
**4**	**Sample size**	40
**5**	**Repeated (Y/N)**	Yes
	**If yes: frequency, (e.g., annually, monthly)**	Monthly, with an option to decrease to quarterly
**6**	**Mode (telephone, web, in person)**	Telephone, web, and in person
**7**	**Self-administered or interviewer administered**	Can be self-administered after the initial interview
**8**	**Informed consent (verbal, written, digital)**	N/A
**9**	**Incentives (Y/N)**	No
	**If yes: incentive type (e.g., gift card, cash, check)**	N/A
**10**	**Seeking Waiver of Documentation of Informed Consent (Y/N)**	No
**11**	**Cross-site or site specific (specify site)**	Cross-site
**12**	**Data collection platform (e.g., REDCap, video)**	REDCap
**13**	**Audio-recording (Y/N)**	No
**14**	**Other respondent-facing material (e.g., email invitation to participate) (specify)**	N/A

HCS Staff Tracker		
**Main Study Data Collection**		
**1**	**Purpose**	To document the labor resources of HCS staff hired to support the CTH implementation in HCS communities.
**2**	**Respondent**	Health economists and collaborating staff from the research sites.
**3**	**How will respondents be selected?**	Data will be collected on all HCS staff hired to support the CTH implementation.
**4**	**Sample size**	Up to 205
**5**	**Repeated (Y/N)**	Yes
	**If yes: frequency (e.g., annually, monthly)**	The data will be updated quarterly to reflect changes in staffing over the course of the project.
**6**	**Mode (telephone, web, in person)**	An excel spreadsheet template will be completed by a responsible person(s) (RPs) at each research site. The KY research site has the option of completing the information via REDCap (see *KY-Staff Research Effort Survey*). RPs may use email, phone, and/or face-to-face interactions when completing the excel spreadsheet/REDCap survey.
**7**	**Self-administered or interviewer administered**	Self-administered
**8**	**Informed consent (verbal, written, digital)**	N/A
**9**	**Incentives (Y/N)**	No
	**If yes: incentive type (e.g., gift card, cash, check)**	N/A
**10**	**Seeking Waiver of Documentation of Informed Consent (Y/N)**	No
**11**	**Cross-site or site specific (specify site)**	Cross-site
**12**	**Data collection platform (e.g., REDCap, video)**	An excel spreadsheet template will be provided to research site RPs. The KY research site has the option to use REDCap to facilitate data entry. All data will ultimately be complied in spreadsheets for systematic analysis.
**13**	**Audio-recording (Y/N)**	No
**14**	**Other respondent-facing material (e.g., email invitation to participate) (specify)**	N/A

Interview Guide Message Testing		
**Main Study Data Collection**		
**1**	**Purpose**	To understand how people who are aware of the opioid crisis in the community respond to messages about naloxone, OUD, MOUD stigma
**2**	**Respondent**	Community leaders and health care providers in each community
**3**	**How will respondents be selected?**	Potential participants may be approached by a coalition member or will respond to an open solicitation via social media and newspaper items. They will be screened for (1) being between ages 18 and 75 and (2) being a resident of the town or county where the groups will take place. The RSs will attempt to have a broad mix of demographic backgrounds and people from different occupation groups in each group (e.g., not all working in the health sector but also representing connections to public safety, law enforcement, business/retail, farming, and other sectors).
**4**	**Sample size**	Total up to 400 across 33 Wave 1 communities
**5**	**Repeated (Y/N)**	Yes
	**If yes: frequency (e.g., annually, monthly)**	These focus groups may be repeated for subsequent communication campaigns but will not include the same participants
**6**	**Mode (telephone, web, in person)**	Telephone
**7**	**Self-administered or interviewer administered**	Interviewer administered
**8**	**Informed consent (verbal, written, digital)**	Verbal
**9**	**Incentives (Y/N)**	Yes
	**If yes: incentive type (e.g., gift card, cash, check)**	$50 or $200 gift card (dependent on respondent type)
**10**	**Seeking Waiver of Documentation of Informed Consent (Y/N)**	Yes
**11**	**Cross-site or site specific (specify site)**	Cross-site
**12**	**Data collection platform (e.g., REDCap, video)**	Audio-recording. These recordings will be saved on secure project servers and deleted after analysis is complete.
**13**	**Audio-recording (Y/N)**	Yes
**14**	**Other respondent-facing material (e.g., email invitation to participate) (specify)**	Email invitation, confirmation, and meeting logistics information

Interview Guide Message Testing Persons with OUD		
**Main Study Data Collection**		
1	**Purpose**	Message testing to ensure relevance and lower risk of unintended effects of the communication campaign
2	**Respondent**	People who self-identify as having OUD or using injection opioids
3	**How will respondents be selected?**	Potential participants will be recruited through print and social media advertisements. We anticipate that some recruitment will also occur through word of mouth in the social and professional networks of coalition members. Participants will be screened to be (1) between ages 18 and 75, (2) a resident of the town or county where the groups will take place, and (3) having a self-reported OUD or self-reporting use of injection opioids (i.e., heroin, fentanyl) or had an opioid overdose in the past year.
4	**Sample size**	N=300 across 33 communities; groups will have between 3 and 8 participants each
5	**Repeated (Y/N)**	Yes
	**If yes: frequency (e.g., annually, monthly)**	Yes, may be repeated for formative research in subsequent communication campaigns
6	**Mode (telephone, web, in person)**	In-person or web conferencing, to be determined by each RS
7	**Self-administered or interviewer administered**	Interviewer administered
8	**Informed consent (verbal, written, digital)**	Verbal
9	**Incentives (Y/N)**	Yes
	**If yes: incentive type (e.g., gift card, cash, check)**	$50–$75 gift card to be determined by the RS
10	**Seeking Waiver of Documentation of Informed Consent (Y/N)**	Yes
11	**Cross-site or site specific (specify site)**	Cross-site
12	**Data collection platform (e.g., REDCap, video)**	Audio-recording
13	**Audio-recording (Y/N)**	Yes. Recordings will be saved on secure project servers and deleted after analysis is complete.
14	**Other respondent-facing material (e.g., email invitation to participate) (specify)**	Email invitation, confirmation, and meeting logistics information to be developed

Landscape Analysis 1 (Wave 1)/Landscape Analysis (Wave 2)		
**Main Study Data Collection**		
**1**	**Purpose**	The Landscape Analysis is a collection of secondary data to describe the external setting in which the HCS will occur.
**2**	**Respondent**	HCS researchers (i.e., study staff), community informants
**3**	**How will respondents be selected?**	N/A
**4**	**Sample size**	One Landscape Analysis will be performed for each community.
**5**	**Repeated (Y/N)**	Yes
	**If yes: frequency (e.g., annually, monthly)**	Data collected at baseline (Phase 0 through Phase 1 of the intervention) and updated as needed throughout the intervention
**6**	**Mode (telephone, web, in person)**	Information will be collected via web searches (i.e., publicly available data), administrative reports, and via conversations with community informants
**7**	**Self-administered or interviewer administered**	N/A
**8**	**Informed consent (verbal, written, digital)**	N/A
**9**	**Incentives (Y/N)**	N/A
	**If yes: incentive type (e.g., gift card, cash, check)**	
**10**	**Seeking Waiver of Documentation of Informed Consent (Y/N)**	N/A
**11**	**Cross-site or site specific (specify site)**	Cross-site
**12**	**Data collection platform (e.g., REDCap, video)**	REDCap
**13**	**Audio-recording (Y/N)**	No
**14**	**Other respondent-facing material (e.g., email invitation to participate) (specify)**	No

Landscape Analysis 2 (Wave 1)/Landscape Analysis Organizational Questionnaire (Wave 2)		
**Main Study Data Collection**		
**1**	**Purpose**	Wave 1: The Asset Classification (AC) is Phase 2 of the Landscape Analysis. The AC will be conducted to capture prevention, treatment, recovery support services, and infrastructure in health care, behavioral health, and criminal justice organizations, along with other key community features. Results of the AC will provide community context and directly inform CE efforts of the HCS. The roster of community-based organizations (assets) for the AC is generated from publicly available data sources gathered during Landscape Analysis Phase 1. The AC will be conducted via telephone, online, or in person with key facility contacts, or those best able to answer questions about opioid use treatment and opioid overdose prevention services at the facility.<br>Wave 2: The Organizational questionnaires will be conducted to capture prevention, treatment, recovery support services, and infrastructure in health care, behavioral health, and criminal justice organizations, along with other key community features. Results of the questionnaires will provide community context and directly inform CE efforts of the HCS. The roster of community-based organizations (assets) is generated from publicly available data sources gathered during Landscape Analysis process (web searching, use of extant data sources and conversations with community informants). The questionnaires will be conducted via telephone, online, or in person with key facility contacts, or those best able to answer questions about opioid use treatment and opioid overdose prevention services at the facility.
**2**	**Respondent**	Organization employees
**3**	**How will respondents be selected?**	A roster of organizations will be generated by LA. The primary respondents for the survey will be employees of the organization/agency that provides opioid-use disorder related services as identified by LA.
**4**	**Sample size**	Sample size is dependent on the number of assets identified by the LA process and difficult to estimate a priori.
**5**	**Repeated (Y/N)**	Yes
	**If yes: frequency (e.g., annually, monthly)**	Data collected at baseline (Phase 0 through 1 of the intervention) and updated as needed throughout the intervention period
**6**	**Mode (telephone, web, in person)**	Agency/organization employee—web based, in person, telephone
**7**	**Self-administered or interviewer administered**	Agency employee—self-administered (if web based) and interviewer administered (if by telephone or in person)

Landscape Analysis 2 (Wave 1)/Landscape Analysis Organizational Questionnaire (Wave 2)		
8	**Informed consent (verbal, written, digital)**	Verbal, written, or digital
9	**Incentives (Y/N)**	No
	**If yes: incentive type (e.g., gift card, cash, check)**	N/A
10	**Seeking Waiver of Documentation of Informed Consent (Y/N)**	Yes
11	**Cross-site or site specific (specify site)**	Site Specific (Kentucky and Ohio)
12	**Data collection platform (e.g., REDCap, video)**	REDCap
13	**Audio-recording (Y/N)**	No
14	**Other respondent-facing material (e.g., email invitation to participate) (specify)**	There will be an email invitation to participate with a follow-up reminder from a data collector, if necessary

Learning Collaborative Evaluation Survey		
**Main Study Data Collection**		
1	**Purpose**	For Learning Collaborative training attendees to rate how well the training session(s) met learning objectives, as well as satisfaction with the training.
2	**Respondent**	Community members from HCS communities
3	**How will respondents be selected?**	Each learning collaborative training attendee will be asked to complete a post-event evaluation survey immediately following the completion of the learning collaborative training.
4	**Sample size**	To be determined.
5	**Repeated (Y/N)**	No. An attendee will complete the evaluation survey for each unique Learning Collaborative training they attend.
	**If yes: frequency (e.g., annually, monthly)**	N/A
6	**Mode (telephone, web, in person)**	Web
7	**Self-administered or interviewer administered**	Self-Administered
8	**Informed consent (verbal, written, digital)**	N/A
9	**Incentives (Y/N)**	No

Learning Collaborative Evaluation Survey		
	**If yes: incentive type (e.g., gift card, cash, check)**	N/A
10	**Seeking Waiver of Documentation of Informed Consent (Y/N)**	N/A
11	**Cross-site or site specific (specify site)**	Site-specific (Ohio)
12	**Data collection platform (e.g., REDCap, video)**	Qualtrics
13	**Audio-recording (Y/N)**	No
14	**Other respondent-facing material (e.g., email invitation to participate) (specify)**	N/A

Medical Examiner Survey		
Main Study Data Collection		
1	**Purpose**	To understand the procedures in each New York county related to determining opioid overdose-related deaths
2	**Respondent**	Medical examiners in New York State
3	**How will respondents be selected?**	Medical examiners in New York's communities
4	**Sample size**	16
5	**Repeated (Y/N)**	No
	**If yes: frequency (e.g., annually, monthly)**	N/A
6	**Mode (telephone, web, in person)**	Web
7	**Self-administered or interviewer administered**	Self-administered
8	**Informed consent (verbal, written, digital)**	Digital
9	**Incentives (Y/N)**	Yes
	**If yes: incentive type (e.g., gift card, cash, check)**	Participants will receive $50 after completing the survey.
10	**Seeking Waiver of Documentation of Informed Consent (Y/N)**	Yes
11	**Cross-site or site specific (specify site)**	Site specific (New York)
12	**Data collection platform (e.g., REDCap, video)**	REDCap
13	**Audio-recording (Y/N)**	No

Medical Examiner Survey		
14	**Other respondent-facing material (e.g., email invitation to participate) (specify)**	Email invitation

Medication Disposal Drop Box Sustainability Interview Guide		
**Main Study Data Collection**		
1	**Purpose**	To gather information about satisfaction with the experience, barriers to implementation and maintenance, readiness for sustainment of drop boxes, training or technical needs related to drop box maintenance, and the inner construct of the pharmacy.
2	**Respondent**	Pharmacies who participated in the HCS medication disposal program.
3	**How will respondents be selected?**	Potential participants will be identified based on participation in the HCS medication disposal program.
4	**Sample size**	64
5	**Repeated (Y/N)**	No
	**If yes: frequency (e.g., annually, monthly)**	N/A
6	**Mode (telephone, web, in person)**	Telephone or Videoconferencing
7	**Self-administered or interviewer administered**	Interviewer administered
8	**Informed consent (verbal, written, digital)**	Verbal
9	**Incentives (Y/N)**	No
	**If yes: incentive type (e.g., gift card, cash, check)**	N/A
10	**Seeking Waiver of Documentation of Informed Consent (Y/N)**	Yes
11	**Cross-site or site specific (specify site)**	Site specific (Kentucky)
12	**Data collection platform (e.g., REDCap, video)**	Video conference (Zoom)
13	**Audio-recording (Y/N)**	Yes
14	**Other respondent-facing material (e.g., email invitation to participate) (specify)**	See *HCS_Medication Disposal Program Pharmacy Interview Guide Subject Facing Materials (KY Site-Specific)*

Mobile MOUD Interview Guide		
**Main Study Data Collection**		
1	**Purpose**	To understand the facilitators of and barriers to implementing mobile MOUD interventions (e.g., interventions that provide mobile access to clinicians who prescribe buprenorphine, naltrexone, or methadone). The interviews will gather information about services offered, barriers and facilitators to standing up such programs, as well as additional factors that are required to implement and sustain each strategy.
2	**Respondent**	Key informants from organizations in HCS Wave 1 communities implementing mobile MOUD interventions.
3	**How will respondents be selected?**	The HCS research teams will identify the key informant(s) at each organization most likely to have knowledge about implementation planning and operations of the mobile MOUD programs.
4	**Sample size**	Up to 33 (1-2 key informants per organization)
5	**Repeated (Y/N)**	No
	**If yes: frequency (e.g., annually, monthly)**	N/A
6	**Mode (telephone, web, in person)**	Telephone or videoconference
7	**Self-administered or interviewer administered**	Interview administered
8	**Informed consent (verbal, written, digital)**	Verbal
9	**Incentives (Y/N)**	Yes
	**If yes: incentive type (e.g., gift card, cash, check)**	$50 gift card or pre-paid debit card (ClinCard)
10	**Seeking Waiver of Documentation of Informed Consent (Y/N)**	Yes
11	**Cross-site or site specific (specify site)**	Site-specific (MA, NY, and OH)
12	**Data collection platform (e.g., REDCap, video)**	Telephone or videoconference (Zoom)
13	**Audio-recording (Y/N)**	Yes
14	**Other respondent-facing material (e.g., email invitation to participate) (specify)**	Yes, see *HCS Mobile MOUD Interview Guide Subject Facing Materials.*

Monthly Coalition Work Group or Subcommittee Meeting Log		
**Main Study Data Collection**		
1	**Purpose**	To assess number, length and type of different CTH subcommittee/workgroup committees that occur in wave 1 communities on a monthly basis
2	**Respondent**	HCS research staff will collect data from CE facilitators
3	**How will respondents be selected?**	HCS research sites will identify HCS staff in each community to complete the form
4	**Sample size**	67
5	**Repeated (Y/N)**	Yes
	**If yes: frequency (e.g., annually, monthly)**	Monthly
6	**Mode (telephone, web, in person)**	Telephone, Web, In Person
7	**Self-administered or interviewer administered**	Self-administered and interviewer administered
8	**Informed consent (verbal, written, digital)**	N/A
9	**Incentives (Y/N)**	No
	**If yes: incentive type (e.g., gift card, cash, check)**	N/A
10	**Seeking Waiver of Documentation of Informed Consent (Y/N)**	N/A
11	**Cross-site or site specific (specify site)**	Cross-site
12	**Data collection platform (e.g., REDCap, video)**	REDCap
13	**Audio-recording (Y/N)**	No
14	**Other respondent-facing material (e.g., email invitation to participate) (specify)**	N/A

MOUD Organization Interview Guide		
**Main Study Data Collection**		
**1**	**Purpose**	To gather information about the barriers to access and retention in MOUD, as well as the impacts of COVID on the delivery of MOUD within the Kentucky HCS communities.
**2**	**Respondent**	Staff working in organizations providing MOUD, which includes, but is not limited to, opioid treatment programs (OTPs), non-OPT specialty substance use disorder programs, and office-based medical practices.
**3**	**How will respondents be selected?**	To select potential respondents, the team will draw upon information provided by the community's HCS coalition, individuals in MOUD organizations that have already implemented fast-track overdose education and naloxone distribution, and the team's professional networks, the Drug Enforcement Agency's list of waivered providers, and MOUD organizations' websites. Approximately 2-3 staff members from a given organization will be asked to participate in the interview.
**4**	**Sample size**	Up to 160
**5**	**Repeated (Y/N)**	No
	**If yes: frequency (e.g., annually, monthly)**	N/A
**6**	**Mode (telephone, web, in person)**	Telephone or Videoconferencing
**7**	**Self-administered or interviewer administered**	Interviewer administered
**8**	**Informed consent (verbal, written, digital)**	Verbal
**9**	**Incentives (Y/N)**	No
	**If yes: incentive type (e.g., gift card, cash, check)**	N/A
**10**	**Seeking Waiver of Documentation of Informed Consent (Y/N)**	Yes
**11**	**Cross-site or site specific (specify site)**	Site specific (Kentucky)
**12**	**Data collection platform (e.g., REDCap, video)**	Video conference (Zoom)
**13**	**Audio-recording (Y/N)**	Yes
**14**	**Other respondent-facing material (e.g., email invitation to participate) (specify)**	See *HCS_KY_Subject Facing Materials for MOUD Organization Interview*

Municipal Drug Policies Interview Guide		
**Main Study Data Collection**		
**1**	**Purpose**	To identify and develop an understanding of municipal policies that may impact the implementation of evidence-based practice (EBP) strategies to prevent opioid overdose in HCS communities.
**2**	**Respondent**	HCS MA community facing staff, HCS MA Community Advisory Board members, HCS MA coalition members, HCS community municipal leadership and staff
**3**	**How will respondents be selected?**	MA research staff will develop a list of potential participants based on HCS staff lists, participant recommendations made by HCS MA staff/CAB members/coalition members, and public facing contact information for municipal leadership and staff
**4**	**Sample size**	50
**5**	**Repeated (Y/N)**	Yes
	**If yes: frequency (e.g., annually, monthly)**	Annually
**6**	**Mode (telephone, web, in person)**	Telephone or Videoconference
**7**	**Self-administered or interviewer administered**	Interviewer administered
**8**	**Informed consent (verbal, written, digital)**	Verbal
**9**	**Incentives (Y/N)**	Yes
	**If yes: incentive type (e.g., gift card, cash, check)**	Gift Card ($25)
**10**	**Seeking Waiver of Documentation of Informed Consent (Y/N)**	Yes
**11**	**Cross-site or site specific (specify site)**	MA Site-Specific
**12**	**Data collection platform (e.g., REDCap, video)**	Telephone or Videoconference
**13**	**Audio-recording (Y/N)**	Yes

14	**Other respondent-facing material (e.g., email invitation to participate) (specify)**	Yes, see *HCS Municipal Drug Policies Interview Guide Subject Facing Materials*

**ORCCA Tracker (ORCCAT)**		
**Main Study Data Collection**		
1	**Purpose**	To track the ORCCA EBP strategies participating HCS communities are selecting and implementing.
2	**Respondent**	Community Coordinators, Program Managers, Others familiar with HCS communities' day-to-day practices.
3	**How will respondents be selected?**	Research site leads will select the appropriate respondents
4	**Sample size**	1 per community
5	**Repeated (Y/N)**	Yes
	**If yes: frequency (e.g., annually, monthly)**	Monthly
6	**Mode (telephone, web, in person)**	Web
7	**Self-administered or interviewer administered**	Self-administered
8	**Informed consent (verbal, written, digital)**	N/A
9	**Incentives (Y/N)**	No
	**If yes: incentive type (e.g., gift card, cash, check)**	N/A
10	**Seeking Waiver of Documentation of Informed Consent (Y/N)**	No
11	**Cross-site or site specific (specify site)**	Cross site
12	**Data collection platform (e.g., REDCap, video)**	REDCap
13	**Audio-recording (Y/N)**	No
14	**Other respondent-facing material (e.g., email invitation to participate) (specify)**	N/A

PARTNER Tool		
**Main Study Data Collection**		
1	**Purpose**	The purpose of the analysis is to better understand the role coalition members (i.e., agencies) play within the coalition, what resources each agency brings to the table, identify activity levels of agencies in the coalition and determine how these agencies interact to address the opioid epidemic in their community(ies).
2	**Respondent**	Respondents will be coalition members from Wave 2 HCS communities.
3	**How will respondents be selected?**	Respondents will be selected from coalition rosters with help from coalition leaders for proper network selection.
4	**Sample size**	The sample size will depend on the size of community coalitions (which vary); there are 9 Wave 2 community coalitions, for a total of 9 networks.
5	**Repeated (Y/N)**	Yes
	**If yes: frequency (e.g., annually, monthly)**	Once more at the end of the study period.
6	**Mode (telephone, web, in person)**	Web or telephone
7	**Self-administered or interviewer administered**	Self-administered
8	**Informed consent (verbal, written, digital)**	Digital or verbal
9	**Incentives (Y/N)**	No
	**If yes: incentive type (e.g., gift card, cash, check)**	N/A
10	**Seeking Waiver of Documentation of Informed Consent (Y/N)**	Yes
11	**Cross-site or site specific (specify site)**	OH Site-Specific
12	**Data collection platform (e.g., REDCap, video)**	Visible Network Labs online instrument website
13	**Audio-recording (Y/N)**	No
14	**Other respondent-facing material (e.g., email invitation to participate) (specify)**	See *HCS PARTNER Tool for Wave 2 Subject Facing Materials (OH Site-Specific)*

Pharmacy Study Interview Guide		
**Main Study Data Collection**		
**1**	**Purpose**	To assess racial/ethnic disparities in Medication for Opioid Use Disorder (MOUD) and Naloxone availability at pharmacies in HCS communities, and to examine the perspectives of People With Opioid Use Disorder (PWOUD) with respect to barriers to accessing MOUD and Naloxone services in pharmacies.
**2**	**Respondent**	There are two types of respondents: 1) Pharmacists who work for pharmacies in New York's HCS communities and 2) People With Opioid Use Disorder (PWOUD).
**3**	**How will respondents be selected?**	Pharmacists will be selected because they work for pharmacies in New York's HCS communities. The NYS Pharmacy Association will provide recruitment assistance to the NY research team.<br><br>People With Opioid Use Disorder (PWOUD) will be selected/recruited from three types of programs serving PWOUD at sites in NY's HCS counties, with diverse communities: a) Methadone Maintenance treatment programs; b) primary care clinics; c) syringe exchange programs. Recruitment will ensure that the sample includes minority representation across non-Hispanic Blacks and Hispanics.
**4**	**Sample size**	20 Pharmacists; 20 People With Opioid Use Disorder
**5**	**Repeated (Y/N)**	Yes
	**If yes: frequency (e.g., annually, monthly)**	Annually
**6**	**Mode (telephone, web, in person)**	Telephone/Video Conference
**7**	**Self-administered or interviewer administered**	Interviewer administered
**8**	**Informed consent (verbal, written, digital)**	Verbal
**9**	**Incentives (Y/N)**	Yes
	**If yes: incentive type (e.g., gift card, cash, check)**	$50
**10**	**Seeking Waiver of Documentation of Informed Consent (Y/N)**	Yes
**11**	**Cross-site or site specific (specify site)**	Site Specific (New York)
**12**	**Data collection platform (e.g., REDCap, video)**	Video Conference

Pharmacy Study Interview Guide		
**13**	**Audio-recording (Y/N)**	Yes
**14**	**Other respondent-facing material (e.g., email invitation to participate) (specify)**	See *NY Pharmacy Study Survey & Interview Guide Subject Facing Materials.*

Pharmacy Study Survey		
**Main Study Data Collection**		
**1**	**Purpose**	To assess racial/ethnic disparities in Medication for Opioid Use Disorder (MOUD) and Naloxone availability at pharmacies in HCS communities., The surveys (one for pharmacy staff or pharmacists and one specifically for licensed pharmacists) will include questions about access to Narcan/Naloxone, Buprenorphine, Naltrexone, Methadone and COVID-19 services in pharmacies located within NY's HCS Communities.
**2**	**Respondent**	Pharmacists, pharmacy technicians and/or other pharmacy staff who work for a pharmacy in one of NY's HCS communities.
**3**	**How will respondents be selected?**	The NYS Pharmacy Association will provide recruitment assistance to the NY research team that will also conduct an online search for pharmacy contact information. The pharmacist, pharmacy technician and/or other pharmacy staff will work for pharmacies in NY's HCS communities.
**4**	**Sample size**	Up to 700
**5**	**Repeated (Y/N)**	Yes
	**If yes: frequency (e.g., annually, monthly)**	Annually
**6**	**Mode (telephone, web, in person)**	Telephone and Web
**7**	**Self-administered or interviewer administered**	Self-administered
**8**	**Informed consent (verbal, written, digital)**	Verbal and digital
**9**	**Incentives (Y/N)**	Yes
	**If yes: incentive type (e.g., gift card, cash, check)**	$15 for Pharmacy Only Survey and $50 for Pharmacist Only Survey
**10**	**Seeking Waiver of Documentation of Informed Consent (Y/N)**	Yes
**11**	**Cross-site or site specific (specify site)**	Site Specific (New York)

Pharmacy Study Survey		
12	**Data collection platform (e.g., REDCap, video)**	REDCap
13	**Audio-recording (Y/N)**	No
14	**Other respondent-facing material (e.g., email invitation to participate) (specify)**	See *NY Pharmacy Study Survey & Interview Guide Subject Facing Materials.*

Photovoice Focus Group Interview Guide		
**Main Study Data Collection**		
1	**Purpose**	To gather community members' perspectives (via shared photographs and focus group discussions) regarding barriers and facilitators that impact efforts to prevent opioid-related overdose deaths in their HCS community and to identify ways to address the challenges; HCS communities will be able to hone the focus of their inquiry to the local context.
2	**Respondent**	Community member or key stakeholder that can provide insight about the state of the opioid epidemic in their HCS community, as well as information about local community resources and responses.
3	**How will respondents be selected?**	Purposeful Selection
4	**Sample size**	Up to 16 individuals per HCS community
5	**Repeated (Y/N)**	No, though one Photovoice project may involve up to 6 sessions
	**If yes: frequency (e.g., annually, monthly)**	N/A
6	**Mode (telephone, web, in person)**	In person or by video conference
7	**Self-administered or interviewer administered**	Focus group is interviewer administered. Brief (5-minute) demographic survey is self-administered.
8	**Informed consent (verbal, written, digital)**	Written or verbal consent
9	**Incentives (Y/N)**	Yes
	**If yes: incentive type (e.g., gift card, cash, check)**	Gift cards (MA will compensate $25 per session, up to $150 per participant; KY and OH will compensate $50 per session, up to $300 per participant)
10	**Seeking Waiver of Documentation of Informed Consent (Y/N)**	Yes, for verbal consent
11	**Cross-site or site specific (specify site)**	Site-Specific (KY, MA and OH)

Photovoice Focus Group Interview Guide		
12	**Data collection platform (e.g., REDCap, video)**	Interviews will be audio-recorded and transcribed. Transcripts coded and saved in Box.com folder. Communities may choose to enter/share some photos via EpiCollect5.
13	**Audio-recording (Y/N)**	Yes
14	**Other respondent-facing material (e.g., email invitation to participate) (specify)**	See *HCS Photovoice Focus Group Subject Facing Materials (MA & OH), HCS Abbreviated Subject Facing Materials for Photovoice Focus Group (KY, MA & OH)* and *HCS Permission to Use Image(s) from Photovoice Process.*

Policy Community Report		
**Main Study Data Collection**		
1	**Purpose**	To document policies reported by the coalition members and research team staff that may facilitate or impede the CTH intervention and track the HCS responses to them.
2	**Respondent**	Research staff
3	**How will respondents be selected?**	Policies that HCS communities encounter or become aware of will be recorded when the research team is notified.
4	**Sample size**	To be determined
5	**Repeated (Y/N)**	No but individual records will be updated as needed
	**If yes: frequency (e.g., annually, monthly)**	N/A
6	**Mode (telephone, web, in person)**	Direct entry into REDCap by research team member
7	**Self-administered or interviewer administered**	Self-administered
8	**Informed consent (verbal, written, digital)**	N/A
9	**Incentives (Y/N)**	No
	**If yes: incentive type (e.g., gift card, cash, check)**	N/A
10	**Seeking Waiver of Documentation of Informed Consent (Y/N)**	N/A
11	**Cross-site or site specific (specify site)**	Cross-site
12	**Data collection platform (e.g., REDCap, video)**	REDCap
13	**Audio-recording (Y/N)**	No

Policy Community Report		
**Main Study Data Collection**		
**14**	**Other respondent-facing material (e.g., email invitation to participate) (specify)**	N/A

Policy Environmental Scan		
**Main Study Data Collection**		
**1**	**Purpose**	To document policies that may facilitate or impede the CTH intervention.
**2**	**Respondent**	Research staff
**3**	**How will respondents be selected?**	An environmental scan will be conducted to identify federal and state level policies.
**4**	**Sample size**	To be determined
**5**	**Repeated (Y/N)**	No but individual records will be updated as needed
	**If yes: frequency (e.g., annually, monthly)**	N/A
**6**	**Mode (telephone, web, in person)**	Direct entry into REDCap by research team member
**7**	**Self-administered or interviewer administered**	Self-administered
**8**	**Informed consent (verbal, written, digital)**	N/A
**9**	**Incentives (Y/N)**	No
	**If yes: incentive type (e.g., gift card, cash, check)**	N/A
**10**	**Seeking Waiver of Documentation of Informed Consent (Y/N)**	N/A
**11**	**Cross-site or site specific (specify site)**	Cross-site
**12**	**Data collection platform (e.g., REDCap, video)**	REDCap
**13**	**Audio-recording (Y/N)**	No
**14**	**Other respondent-facing material (e.g., email invitation to participate) (specify)**	N/A

Post Coalition Meeting Feedback Form		
**Main Study Data Collection**		
1	**Purpose**	To elicit anonymous feedback from coalition members on CTH coalition meeting minutes
2	**Respondent**	Coalition members
3	**How will respondents be selected?**	Will be an active member of the coalition; evaluation form to be completed after coalition meetings
4	**Sample size**	To be determined
5	**Repeated (Y/N)**	Yes
	**If yes: frequency (e.g., annually, monthly)**	As needed
6	**Mode (telephone, web, in person)**	In person
7	**Self-administered or interviewer administered**	Self-administered
8	**Informed consent (verbal, written, digital)**	N/A
9	**Incentives (Y/N)**	No
	**If yes: incentive type (e.g., gift card, cash, check)**	N/A
10	**Seeking Waiver of Documentation of Informed Consent (Y/N)**	No
11	**Cross-site or site specific (specify site)**	Site specific (New York)
12	**Data collection platform (e.g., REDCap, video)**	Will be completed on paper, then data from forms will be entered into REDCap
13	**Audio-recording (Y/N)**	No
14	**Other respondent-facing material (e.g., email invitation to participate) (specify)**	N/A

Qualitative Assessment Form for Campaign 4		
**Main Study Data Collection**		
**1**	**Purpose**	To learn what HCS communication campaign materials and distribution methods worked well and what could be improved.
**2**	**Respondent**	Community staff or Coalition members
**3**	**How will respondents be selected?**	Person(s) in each community primarily responsible for campaign implementation
**4**	**Sample size**	Will vary, 1-3 per community site
**5**	**Repeated (Y/N)**	N
	**If yes: frequency (e.g., annually, monthly)**	
**6**	**Mode (telephone, web, in person)**	Web
**7**	**Self-administered or interviewer administered**	Self-Administered; HCS staff will follow-up if there are questions about the respondent’s answers.
**8**	**Informed consent (verbal, written, digital)**	N/A
**9**	**Incentives (Y/N)**	N/A
	**If yes: incentive type (e.g., gift card, cash, check)**	N/A
**10**	**Seeking Waiver of Documentation of Informed Consent (Y/N)**	N/A
**11**	**Cross-site or site specific (specify site)**	Cross-site
**12**	**Data collection platform (e.g., REDCap, video)**	N/A
**13**	**Audio-recording (Y/N)**	No
**14**	**Other respondent-facing material (e.g., email invitation to participate) (specify)**	Yes, see *HCS Campaign 4 Qualitative Assessment Subject Facing Materials*

Race and Ethnicity Data Collection Readiness Survey		
**Main Study Data Collection**		
**1**	**Purpose**	To identify the process that organizations are using to collect and monitor demographic data in NY's HCS communities.
**2**	**Respondent**	Key informants working at NY's HCS partner organizations.
**3**	**How will respondents be selected?**	The NY HCS research team will develop a list of potential survey participants from internal study records. To gauge interest, potential participants will be contacted by the research team via email.
**4**	**Sample size**	Between 60 and 70 Wave 1 organizations will be recruited to participate in the survey.
**5**	**Repeated (Y/N)**	No
	**If yes: frequency (e.g., annually, monthly)**	N/A
**6**	**Mode (telephone, web, in person)**	Mail, Web or via video conference/phone.
**7**	**Self-administered or interviewer administered**	Self-administered if conducted by mail or web and interviewer administered if conducted by video conference/phone.
**8**	**Informed consent (verbal, written, digital)**	In-Person, Verbal or web consent
**9**	**Incentives (Y/N)**	No
	**If yes: incentive type (e.g., gift card, cash, check)**	N/A
**10**	**Seeking Waiver of Documentation of Informed Consent (Y/N)**	Yes, for verbal and web consent
**11**	**Cross-site or site specific (specify site)**	Site-Specific (NY)
**12**	**Data collection platform (e.g., REDCap, video)**	REDCap Survey
**13**	**Audio-recording (Y/N)**	No
**14**	**Other respondent-facing material (e.g., email invitation to participate) (specify)**	See *HCS Subject Facing Materials for NY Race and Ethnicity Data Collection Readiness Survey*

Reach Tracker		
**Main Study Data Collection**		
1	**Purpose**	To track aggregate reach data for the Overdose Reduction Continuum of Care Approach (ORCCA) evidence-based practices (EBP) strategies HCS communities are implementing.
2	**Respondent**	HCS program or research staff
3	**How will respondents be selected?**	RSs will identify HCS program or research staff from each community who will be responsible for filling out the tracker for each community.
4	**Sample size**	Variable, up to 264 across all 4 sites (8 per community for 33 communities across 4 sites)
5	**Repeated (Y/N)**	Y
	**If yes: frequency (e.g., annually, monthly)**	Once data collection begins (estimated start date: December 2021), the Reach Tracker will be administered 45 days after close of the month, and data are submitted on the 15th of the month. For example, January 2022 data are due March 15.
6	**Mode (telephone, web, in person)**	Web (REDCap instrument)
7	**Self-administered or interviewer administered**	Self-administered
8	**Informed consent (verbal, written, digital)**	N/A
9	**Incentives (Y/N)**	No
	**If yes: incentive type (e.g., gift card, cash, check)**	N/A
10	**Seeking Waiver of Documentation of Informed Consent (Y/N)**	N/A
11	**Cross-site or site specific (specify site)**	Cross-site
12	**Data collection platform (e.g., REDCap, video)**	REDCap
13	**Audio-recording (Y/N)**	No
14	**Other respondent-facing material (e.g., email invitation to participate) (specify)**	N/A

State Grant Funding		
**Main Study Data Collection**		
**1**	**Purpose**	Collect information on federal resources going to communities in the HCS states
**2**	**Respondent**	State employee key informant working in the relevant state agency
**3**	**How will respondents be selected?**	Research staff will reach out to a contact at each state who will help us identify the individual who can best extract data for our tool
**4**	**Sample size**	4 (one from each state)
**5**	**Repeated (Y/N)**	Yes
	**If yes: frequency (e.g., annually, monthly)**	Annually
**6**	**Mode (telephone, web, in person)**	Telephone introduction followed by emailed spreadsheet for data entry
**7**	**Self-administered or interviewer administered**	This is a collection of secondary (administrative) data. The key informant will work with the state's administrative records to enter data on programs into the spreadsheet.
**8**	**Informed consent (verbal, written, digital)**	Verbal
**9**	**Incentives (Y/N)**	No
	**If yes: incentive type (e.g., gift card, cash, check)**	N/A
**10**	**Seeking Waiver of Documentation of Informed Consent (Y/N)**	Yes
**11**	**Cross-site or site specific (specify site)**	Cross-site
**12**	**Data collection platform (e.g., REDCap, video)**	Emailed spreadsheet along with telephone support as necessary
**13**	**Audio-recording (Y/N)**	No
**14**	**Other respondent-facing material (e.g., email invitation to participate) (specify)**	Telephone introduction

Sustainability Partner Organization Interview Guide		
**Main Study Data Collection**		
1	**Purpose**	To expand knowledge regarding experiences of Wave 1 partner organizations in implementing ORCCA Menus 1 and 2 and to learn about the period of early sustainment.
2	**Respondent**	Staff from Wave 1 organizations that partnered with the HCS across all four HCS research sites.
3	**How will respondents be selected?**	Purposive sample that includes a range of organizations, including those located in rural and urban communities, those that are or are not represented on the coalition, and those in the three primary sectors of HCS (health care, behavioral health, and criminal justice).
4	**Sample size**	Approximately 450 participants
5	**Repeated (Y/N)**	No
	**If yes: frequency (e.g., annually, monthly)**	N/A
6	**Mode (telephone, web, in person)**	Videoconference or telephone
7	**Self-administered or interviewer administered**	Interviewer administered
8	**Informed consent (verbal, written, digital)**	Verbal
9	**Incentives (Y/N)**	Yes
	**If yes: incentive type (e.g., gift card, cash, check)**	$50 (check, cash, gift card)
10	**Seeking Waiver of Documentation of Informed Consent (Y/N)**	Yes
11	**Cross-site or site specific (specify site)**	Cross-site
12	**Data collection platform (e.g., REDCap, video)**	Videoconference or telephone
13	**Audio-recording (Y/N)**	Yes
14	**Other respondent-facing material (e.g., email invitation to participate) (specify)**	Yes, see *Subject Facing Materials for Sustainability Partner Organization Interview Guide*

Toxicology Survey for Labs		
**Main Study Data Collection**		
1	**Purpose**	To gather data on the characteristics of post-mortem toxicology testing for suspected drug overdose deaths. Information will also be collected on the processes and barriers related to toxicology testing.
2	**Respondent**	Toxicology laboratory staff
3	**How will respondents be selected?**	Staff who work at a lab providing post-mortem toxicology testing in HCS study communities.
4	**Sample size**	Up to 200
5	**Repeated (Y/N)**	No
	**If yes: frequency (e.g., annually, monthly)**	N/A
6	**Mode (telephone, web, in person)**	Web or telephone
7	**Self-administered or interviewer administered**	Both; Self-administered (web) or interviewer-administered (telephone)
8	**Informed consent (verbal, written, digital)**	Digital and verbal
9	**Incentives (Y/N)**	No
	**If yes: incentive type (e.g., gift card, cash, check)**	N/A
10	**Seeking Waiver of Documentation of Informed Consent (Y/N)**	Yes
11	**Cross-site or site specific (specify site)**	Cross-site
12	**Data collection platform (e.g., REDCap, video)**	REDCap
13	**Audio-recording (Y/N)**	No
14	**Other respondent-facing material (e.g., email invitation to participate) (specify)**	Yes, see *Toxicology Survey for Labs Subject Facing Materials*

Toxicology Survey for Medical Examiners & Coroners		
**Main Study Data Collection**		
**1**	**Purpose**	To gather information about the processes and barriers affecting the completion of death certificates for suspected drug overdose deaths.
**2**	**Respondent**	Medical examiners and coroners
**3**	**How will respondents be selected?**	Medical Examiners or coroners in one of the HCS communities.
**4**	**Sample size**	Up to 200
**5**	**Repeated (Y/N)**	No
	**If yes: frequency (e.g., annually, monthly)**	N/A
**6**	**Mode (telephone, web, in person)**	Web or telephone
**7**	**Self-administered or interviewer administered**	Both; Self-administered (web) or interviewer-administered (telephone)
**8**	**Informed consent (verbal, written, digital)**	Digital and verbal
**9**	**Incentives (Y/N)**	No
	**If yes: incentive type (e.g., gift card, cash, check)**	N/A
**10**	**Seeking Waiver of Documentation of Informed Consent (Y/N)**	Yes
**11**	**Cross-site or site specific (specify site)**	Site Specific (Kentucky, Massachusetts, and Ohio)
**12**	**Data collection platform (e.g., REDCap, video)**	REDCap
**13**	**Audio-recording (Y/N)**	No
**14**	**Other respondent-facing material (e.g., email invitation to participate) (specify)**	Yes, see *Toxicology Survey for Medical Examiners Subject Facing Materials for KY MA OH*

Training and Technical Assistance Tracking (TTAT) Form		
**Main Study Data Collection**		
1	**Purpose**	To assess cost, dosage, and adherence of training or technical assistance service for CE or ORCCA activity as an implementation strategy
2	**Respondent**	HCS project staff
3	**How will respondents be selected?**	HCS project staff from each RS
4	**Sample size**	To be determined
5	**Repeated (Y/N)**	Yes
	**If yes: frequency (e.g., annually, monthly)**	Monthly
6	**Mode (telephone, web, in person)**	In person
7	**Self-administered or interviewer administered**	Self-administered
8	**Informed consent (verbal, written, digital)**	N/A
9	**Incentives (Y/N)**	No
	**If yes: incentive type (e.g., gift card, cash, check)**	N/A
10	**Seeking Waiver of Documentation of Informed Consent (Y/N)**	No
11	**Cross-site or site specific (specify site)**	Cross-site
12	**Data collection platform (e.g., REDCap, video)**	Completed on paper, then data will be entered into REDCap
13	**Audio-recording (Y/N)**	No
14	**Other respondent-facing material (e.g., email invitation to participate) (specify)**	N/A

8.2 Safety Assessments

As described in Section 2.3, the risks associated with the CTH intervention are minimal. The CTH intervention does not directly treat members of the communities. Rather, it assists the communities and their service venues in selecting evidence-based practices (EBPs) that the communities will implement, and which were previously demonstrated as safe and effective. Thus, safety outcomes are not the driving issue for our monitoring assessments, and we do not expect to find adverse impacts of the HCS intervention. However, recognizing that the study will

collect data on opioid-related mortality and morbidity, the HCS will monitor adverse events (AEs) and serious adverse events (SAEs). It is very unlikely that opioid-related overdose events will be directly associated with the study; nevertheless, Wave 1 and Wave 2 communities will be monitored, and monthly reports of AEs and SAEs will be prepared and reviewed as described below.

The primary outcome for the HCS is opioid-related overdose fatalities; however, accurate fatality data are not available in a timely manner (i.e., the data lag is 6 months or more). Therefore, monitoring of opioid-related overdose fatalities is not a feasible measure that is available rapidly and reliably. For the HCS, we will monitor emergency medical services (EMS) runs for suspected opioid-related overdoses where naloxone was administered. The four HCS Research Sites (RSs) already have access to EMS runs that could be requested monthly, with a 30–90-day lag after the end of each month. The HCS will estimate the rate of suspected opioid-related overdose events by month in a community with the rate being the number of events per 1,000 community members.[72] In this manner, EMS runs provide the HCS with an early warning sign of an increase in opioid-related overdose events in HCS communities.

To determine if there is a situation where AEs or SAEs might be related to the HCS, we will employ a two-step AE/SAE review process (Figure 10). Step one involves distinguishing whether the observed AE or SAE from monthly monitoring reports is a “safety signal”. If it is, then we will proceed with step two which involves assessing if the AE or SAE is related to the HCS.

Figure 10: Two-Step AE/SAE Review Process

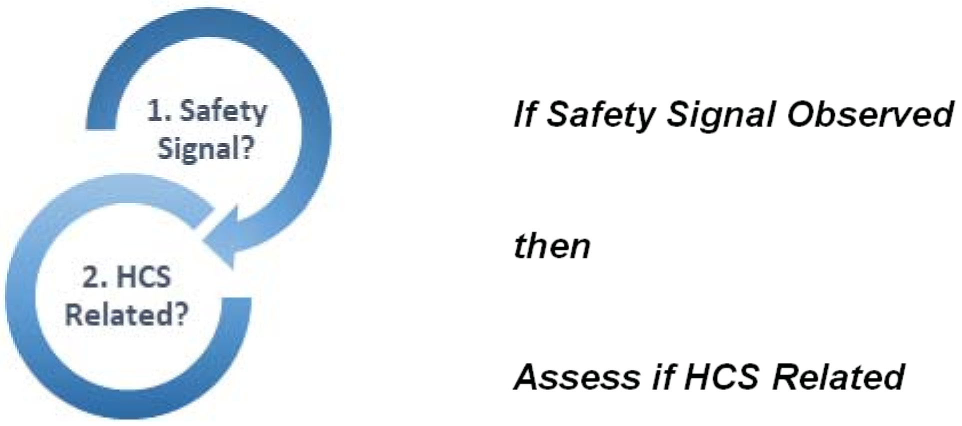

We consider the following criteria when assessing if an AE or SAE is a safety signal:

1) *Has the community had a previous AE/SAE?* This would depict some repeatable pattern within a community rather than one isolated event.

2) *Do AEs/SAEs repeat across adjacent months in a community?* Two or more continuous months with an AE/SAE are more indicative of a building concern.

3) *Is the observed increase in the number of EMS runs for a month meaningful?* In small communities with low counts of EMS runs per month (often with zero or one EMS run in a month), an increase of a few runs could result in an AE or SAE due to a small 12-

month running standard deviation. Therefore, we only want to flag instances with meaningfully large observed increases.

4) *Do several communities have AEs/SAEs in the same month?* We are interested in whether there is a widely prevalent occurrence of AEs/SAEs.

If one or more of these criteria are met for an AE or SAE, and it is thus deemed a safety signal, we will conduct a relatedness analysis which might include examining if there are non-HCS events within a community that might increase opioid related events, if there are state/national events or trends related to increased opioid related events, if HCS and non-HCS populations within each state differ in opioid related events, and if Wave 1 and Wave 2 communities within each state differ in opioid related events. This AE/SAE review process will allow the HCS to use a managed process to determine safety signals and relatedness to the HCS.

8.3 Adverse Events

8.3.1 Definition of Adverse Events and Serious Adverse Events

The HCS will use EMS opioid-related overdose runs to monitor opioid-related overdose events as an AE and an SAE. These events provide important knowledge about the opioid crisis that the HCS should ethically investigate and report to the community coalitions involved, the National Institute on Drug Abuse (NIDA), the Substance Abuse and Mental Health Services Administration SAMHSA, the Institutional Review Board (IRB), and the Data and Safety Monitoring Board (DSMB). The HCS does not collect any data attributable to specific individuals who received services from the HCS intervention. Only aggregate date for an entire community will be available.

- AEs will be identified by the following criterion:
  - The opioid-related overdose rate for a single month increases **more than three standard deviations** above the moving average of the previous 3 months. For example, the rate in a community for April 2020 is more than three standard deviations greater than the average rate for January, February, and March 2020.
- SAEs will be identified by the following criterion:
  - The opioid-related overdose rate for a single month increases **more than four standard deviations** above the moving average of the previous 3 months. For example, the rate in a community for April 2020 is more than four standard deviations greater than the average rate for January, February, and March 2020.

The standard deviation will be determined as the rolling standard deviation calculated from the 12 months prior to the target month.

Opioid-related overdoses are expected events in the HCS communities because, to be eligible for being selected into the HCS, NIDA required communities to being highly affected by the opioid epidemic with minimum numbers and rates per 100,000 of opioid-related overdose fatalities in 2016.

We will follow a metric-based approach to help identify safety signals and evaluate relatedness to the HCS. Table 11 describes both the monthly and cross-month metrics that serve as triggers

for further analyses. If one of these events occurs, we will consider it a safety signal and will proceed with conducting a relatedness analysis. This analysis will help determine whether observed AE/SAEs are directly related to the HCS intervention and inform further action if necessary.

Table 11: Metrics Used to Declare a Safety Signal in AE/SAE Review Process

Monthly Metrics	Cross-Month Metrics
• 5 Adverse Events<br>• 2 Serious Adverse Events<br>• 1 Serious Adverse Event + 3 Adverse Events<br>• Steering Committee Direction<br>• DSMB Direction	• 3 Consecutive Adverse Events within the Same Community<br>• Consecutive Adverse Event and Serious Adverse Event<br>• Steering Committee Direction<br>• DSMB Direction

8.3.2 Time Period and Frequency for Event Assessment and Follow-Up

The RSs will provide monthly reports of the estimated number of opioid-related overdoses from the EMS runs in each of their communities to the Data Coordinating Center (DCC). These data will be available approximately by the end of the month after a target month for the Kentucky and Ohio sites, two months after a target month for the Massachusetts site, and on a quarterly schedule from the New York site. As part of the DCC's regular processing of the data, AEs and SAEs will be determined within 9 days of the receipt of the monthly data from all communities to allow for data cleaning and validation to minimize erroneous AEs or SAEs.

8.3.2.1 Adverse Event Reports to the Steering Committee

A monthly report of AEs will be sent to the Steering Committee (SC). This report will include all AEs that have occurred since the last report. The SC will review the report and determine what actions should be taken, including ongoing AE monitoring.

8.3.2.2 Adverse Event Reports to the DSMB, NIDA, and SAMHSA

A monthly and semi-annual report of AEs will be sent to the DSMB, NIDA, and SAMHSA. This report will include all AEs that have occurred since the last report. The DSMB will recommend any additional actions it deems appropriate to the NIDA Director.

8.3.2.3 Serious Adverse Event Reports and Follow-Up

Once an SAE is identified, the DCC will email a report to the SC, NIDA, and SAMHSA within 24 hours. The SC will meet within 24 hours of notification to determine whether the SAE is related to the CTH intervention and assign it to one of three categories:

1. Definitely related to the CTH intervention

3. Possibly related to the CTH intervention
5. Unrelated to the CTH intervention

Because the CTH intervention is directed toward community coalitions, it is unlikely that SAEs will be related to the CTH intervention. The DSMB will be notified within 24 hours of the SC determination. If the SC determines the SAE is related to the CTH intervention, the IRB will be notified within 24 hours. The IRB or the DSMB may request additional information or recommend actions for the SC to take.

If the SC determines that follow-up action is needed, then the course of action will depend on whether the affected community is in Wave 1 or Wave 2. When a community is participating in the CTH intervention, it will incorporate this new information into the implementation of the CTH intervention (e.g., coalition members will be notified). When a community is *not* participating in the CTH intervention, it will be informed of the SAE and may elect to address the event without facilitation from the HCS team.

8.4 Unanticipated Problems

8.4.1 Definition of Unanticipated Problems

This protocol uses the definition of unanticipated problems (UPs) as defined by the U.S. Department of Health and Human Services Office for Human Research Protections (OHRP). OHRP considers UPs involving risks to participants or others to include, in general, any incident, experience, or outcome that meets ***all*** the following criteria:

- Unexpected in terms of nature, severity, or frequency given (1) the research procedures that are described in the protocol-related documents, such as the IRB-approved research protocol and informed consent document; and (2) the characteristics of the participant population being studied
- Related or possibly related to participation in the research ("possibly related" means there is a reasonable possibility that the incident, experience, or outcome may have been caused by the CTH intervention)
- Suggests that the research places participants or others at a greater risk of harm (including physical, psychological, economic, or social harm) than was previously known or recognized

8.4.2 Unanticipated Problem Reporting

The following steps will be used to review and report an UP:

- The contact Principal Investigators at the RSs and the DCC will be responsible for identifying any event, incident, experience, or outcome that is a potential UP.
- A potential UP will be discussed by the SC within 1 workday to determine whether it meets the criteria in Section 8.4.1, and it will be reported to the IRB (as needed), NIDA, SAMHSA, and the DSMB by the next workday.
- The procedures in Section 8.4.1 will be followed to assess a potential UP and recommend any corrective actions that might be appropriate.

- The SC will make the final determination of whether the event, incident, experience, or outcome is a UP or not.
- The findings of the SC and the associated report will be submitted to NIDA, SAMHSA, the DSMB, and the IRB (if determined to be a UP). The report will include the following:
  - A detailed description of the event, incident, experience, or outcome
  - An explanation of the basis for determining that the event, incident, experience, or outcome represents an UP or not
  - A description of any changes to the protocol or other corrective actions that have been taken or are proposed in response to the UP

8.4.3 Reporting Unanticipated Problems to HCS Communities

The HCS Communications team, in conjunction with the HCS SC and the NIDA Office of Science Policy and Communications, will be responsible for reporting UPs or AEs. The steps will include the following:

- Monitoring emerging issues
- Assessing the potential for a situation to develop into a crisis
- Identifying appropriate communications strategies and actions
- Briefing spokespeople
- Developing materials to respond to the situation
- Engaging media and community channels as necessary and appropriate
- Keeping partners and stakeholders informed of the situation
- Evaluating responses and adjusting strategies as needed

9. STATISTICAL CONSIDERATIONS

9.1 Statistical Hypotheses

The HEALing Communities Study (HCS) has one primary hypothesis (H1) and three secondary hypotheses (H2, H3, H4). Compared with Wave 2 communities, we hypothesize the following:

H1: Wave 1 communities will reduce opioid overdose deaths.

H2: Wave 1 communities will increase naloxone distribution.

H3: Wave 1 communities will expand utilization of buprenorphine for opioid use disorder.

H4: Wave 1 communities will reduce high-risk opioid prescribing.

9.2 Sample Size Determination

A total of 67 clusters or communities will be randomized and analyzed in an intention-to-treat (ITT) approach based on a negative binomial regression model, as described in Section 9.4.2. Detailed as follows, this study is designed to have >99% power to detect a 40% (i.e., relative

risk 0.60) reduction in opioid overdose deaths between Wave 1 and Wave 2 communities during the 12-month period of the HCS multi-site, parallel arm, cluster randomized, wait-list controlled trial. Our calculations are based on a simulation study driven by pilot data from all 67 communities, including actual community sizes and corresponding numbers of opioid overdose deaths.

Our simulation study was conducted using 20,000 replications to ensure very small error in estimated powers (i.e., 95% confidence intervals corresponding to our calculated power are no wider than 0.011 for power ≥0.80). This simulation study is advantageous relative to a basic power calculation approach due to the ability to account for the high variation in community sizes, as well as its ability to adapt to the analytical approach (negative binomial regression model) that will be used. To conduct our study, actual data from these 67 communities were used to empirically drive our population assumptions.

For each community, we took the average reported number of individuals in the given community and the average number of opioid overdose deaths. A negative binomial regression model was then fit to these data to extract estimates for the marginal parameters in the regression model, as well as the dispersion parameter, k, corresponding to between-community variation as expected in cluster trials. We note that this model did not include an intervention effect, because the pilot data are reflective of control conditions, and the desired intervention effect is dictated by the assumed risk reduction for which we calculate power. Furthermore, due to the unknown influence observed community-specific baseline opioid overdose death rates have on future rates, and more importantly, in order to provide conservative power calculations (greater power will potentially be achieved in our actual analysis via a reduction in unexplained variability due to the use of the observed baseline opioid overdose death rate as a covariate in the regression model), this variable was not included as a covariate in the regression model for the simulation study. In short, we obtained a value of 0.0431 for k, and with the resulting regression parameter estimates, marginal probabilities ranged from 0.000255 to 0.000436.

To conduct our simulation study, we assume that these population parameters just described will be reflective of the true parameters for the wait-list comparison population at the time of the 12-month parallel-arm trial period. The analyses were conducted as described in Section 9.4.2, with the exception that observed baseline opioid overdose death rate for the community was not incorporated into the model as a covariate, thus providing conservative power estimates. Table 12 provides power estimates for a variety of reductions in opioid overdose deaths for the Wave 1 and Wave 2 communities. As can be seen, we have greater than 99% power to detect a 40% (i.e., relative risk 0.60) reduction and at least 83% power for any reduction of 20% or more.

Table 12: Power calculation for underlying reduction in risk of opioid overdose fatalities between Wave 1 and Wave 2 communities

Intervention Effect	Relative Risk (Opioid Overdose Fatality)	Power
40%	0.60	0.999
25%	0.75	0.961
24%	0.76	0.943
23%	0.77	0.925

Intervention Effect	Relative Risk (Opioid Overdose Fatality)	Power
22%	0.78	0.899
21%	0.79	0.866
20%	0.80	0.832
19%	0.81	0.792
18%	0.82	0.748
0%	1.00	0.075

9.3 Populations for Analyses

Analyses of efficacy outcome measures and safety measures will be conducted using the ITT approach (i.e., including all randomized HCS communities according to their assigned group).

9.4 Statistical Analyses

9.4.1 General Approach

The HCS is a multi-site, parallel arm, cluster randomized, wait-list controlled trial of Communities That HEAL (CTH). All statistical computations will be performed by HCS biostatisticians. For summaries of study data, categorical measures will be summarized in tables listing the frequency and the percentage of participating communities; continuous data will be summarized by presenting mean, standard deviation, median, 95% confidence intervals, and range; and ordinal data will be summarized by only presenting median and range. The balance or imbalance of these characteristics will be studied and reported, particularly for analyses comparing the two study groups. Graphical displays will be used to show distributions (box plots, density curves). The reported p-values will be based on two-sided tests at an $\alpha=0.05$ unless otherwise specified and statistical models will generally be adjusted for the covariates in the randomization. No adjustment will be made for multiple testing for the primary analyses given there is a single primary outcome being compared between two arms. For the analysis and modeling of the data, general methodological standards will be followed throughout, including proper handling of missing data, assessing model assumptions, incorporating appropriate covariates, and conducting sensitivity analysis to assess robustness of findings. For continuous outcomes, checks of normality will be performed and if required, transformations or non-parametric tests will be employed. Additional details for potential covariate adjustments in secondary analyses or handling violations of analytic method assumptions will be detailed in the statistical analysis plan.

The analyses will be done using an ITT approach, including all randomized HCS communities according to assigned group. For the primary and secondary outcomes, every effort will be made to minimize missing data; however, in the event that missing data do exist, we will document the process that resulted in the missing data and consider model-based imputation methods to account for the missing data if needed.[73] In short, guidelines for missing data in clinical trials prescribed by a National Research Council report,[74] and guidelines for handling

missing data in cluster trials, will be followed. Additional details will be supplied in the statistical analysis plan.

Baseline counts and rates of opioid overdose deaths in HCS communities, and other outcome measures needed for the statistical analysis, will be collected based on the 24 months preceding the study initiation, where available. Additionally, baseline counts and rates will be used for dashboard visualization measures, where available. Baseline data may be used in additional state-specific analyses, including geospatial analyses using address information (e.g., from death certificate data, including address of death and address of residence) where permitted by site's state-specific data use agreements.

9.4.2 Analysis of the Primary Outcome

The primary outcome is the number of opioid overdose deaths that occur in the 12-month evaluation period of the HCS multi-site, parallel arm, cluster randomized, wait-list controlled trial. The evaluation period is defined as the last year of the waiting period for Wave 2 communities. The number of opioid overdose deaths is an aggregate outcome assessed at the community level. The primary comparison of interest is between Wave 1 communities compared with the Wave 2 communities during the 12-month evaluation period. The analysis will assess the primary hypothesis (H1 in Section 9.1) that CTH intervention will reduce opioid overdose deaths. The primary analysis will use an ITT approach as described above.

A marginal negative binomial regression model will be utilized to analyze the count outcomes. The model will include trial arm as the main independent variable. In addition, the model will control for the following covariates: the Research Site (RS) (i.e., Kentucky, Massachusetts, New York, or Ohio), the rural/urban status of the community, and the observed baseline opioid overdose death rate for the community. The reported natural log of the population size for each community will be utilized to offset the model, such that our proposed model is a model for the probability of an opioid overdose death in the population. The interpretation from the proposed model will therefore be with respect to changes in the population probabilities (i.e., risk ratios for opioid overdose death for Wave 1 vs. Wave 2 communities). We note that alternatively, the analysis can also be interpreted as a model for opioid overdose death rate per individual, evaluating changes in rates (i.e., rate ratios). RS, rural/urban status, baseline opioid overdose death rate, and population size are accounted for in the model in order to increase statistical power because each of these factors was included in the constrained randomization scheme.

The previously described marginal negative binomial regression model will be fit utilizing PROC GLIMMIX in SAS (version 9.4 or higher). We note that GLIMMIX will utilize maximum likelihood theory to estimate parameters. However, in general, this procedure utilizes unbiased estimating equations for regression parameter estimation; therefore, quasi-likelihood theory or generalized estimating equation (GEE) theory applies. Specifically, we will utilize small-sample adjusted empirical standard error estimates and degrees of freedom equal to the number of communities minus the number of regression parameters. Furthermore, as a sensitivity analysis, a permutation test at the 0.05 significance level will be conducted with respect to the impact of the intervention. This test will be based on our implementation of constrained randomization.

9.4.3 Analysis of the Secondary Outcomes

As with the primary outcome, secondary and structural outcomes will be modeled using an ITT approach as described above. The models will include trial arm as the main independent variable and will control for the following covariates: the RS (i.e., Kentucky, Massachusetts, New York, or Ohio), the rural/urban status of the community, baseline opioid overdose death rate, and the observed baseline level of the particular secondary outcome for the community. The secondary outcomes related to H2–H4 in Section 9.1 are counts or rates; therefore, a marginal negative binomial regression model estimated using GEE will be utilized to analyze the count outcomes, as with the primary analysis. For those outcomes that involve a rate, the implied denominator in the county during the 12-month parallel-arm trial period will be utilized to offset the model, such that the outcome is interpreted as a rate, and differences between treatment assignments would be rate ratios. A model-based approach will be used for hypothesis testing, as described in the primary analysis.

9.4.4 Baseline Descriptive Statistics

We will collect and describe the following baseline characteristics overall and by randomized group: demographics, community socio-economic characteristics, policy/behavioral characteristics, and baseline values of structural variables. As recommended in the CONSORT guideline,[75] these characteristics will not be statistically tested for differences by randomized group.

9.4.5 Subgroup Analyses

There are two planned subgroup analyses based on the categorical stratification factors in the covariate-constrained randomization: urban/rural and RS (i.e., Kentucky, Massachusetts, New York, or Ohio). Subgroup analyses will follow the same analysis plan as the primary and secondary analyses. However, to test for the subgroup effect, an interaction effect (e.g., subgroup identifier * trial arm condition) will be included. If this statistical test is not significant at the $\alpha=0.05$ level, then there will be no further examination of the subgroup. If the statistical test is significant at $\alpha=0.05$, then tests of significance of trial arm condition in each subgroup level will be examined, using an adjusted alpha level that accounts for the number of tests.[75] Analytic approaches for additional subgroups or subgroups involving subsets of populations in communities will be conducted as described in Section 9.4.6.

9.4.6 Exploratory Analyses

Subgroup analyses are planned (Section 9.4.5), but additional subgroup analyses by sex and race will also be conducted. This type of subgroup analysis subsets the population in communities and partitions the outcome into separate aggregate counts for each subgroup in a community. The statistical model will use GEE as described in primary and secondary analyses but will account for the additional nesting of subgroups in a community. Grouping or redefining subgroup identifiers may be necessary with small community-level counts. Therefore, these analyses will be considered exploratory. The statistical model will utilize the same general GEE approach as the primary and secondary analyses, utilizing a negative binomial distribution, including a population offset to estimate a rate, rather than a count. The initial test of subgroup

effect will examine the significance of the interaction of the subgroup indicator with the trial arm condition (see Section 9.4.5 for the conduct of subgroup analyses).

For Steering Committee approved non-hypothesis driven analyses, the comparison group may either be Wave 2 communities and/or the rest-of-the-state (non-Wave 1 communities) dependent upon aims and the statistical analysis plan. The RSs have access to the rest-of-the-state administrative data given approved security procedures, restricted access, and DUAs. In addition, the HCS has a full Health Insurance Portability and Accountability Act (HIPAA) waiver for use of secondary administrative data. The risk-benefits of using Wave 2 or the rest-of-the state administrative data as the comparison group is not changed.

RSs and/or the DCC will conduct Steering Committee approved adjacent analyses. These analyses will utilize state-specific data and/or additional data collected from site-specific research activities (approved by the sIRB). These analyses will adhere to the same rigorous data security processes as those used for hypothesis driven analyses.

10. SUPPORTING DOCUMENTATION AND OPERATIONAL CONSIDERATIONS

10.1 Regulatory, Ethical, and Study Oversight Considerations

10.1.1 Informed Consent

The cluster randomized trial design of the HEALing Communities Study (HCS) has structured the overall consent process in ways that significantly differ from a standard randomized controlled trial. It is important to note that the HCS is a study of the Communities That HEAL (CTH) intervention, the primary targets of which are 67 communities as a whole and their members. Data collected in the process of implementing the CTH intervention will involve surveys and qualitative interviews with state Community Advisory Board (CAB) and community coalition members and service venue staff. Data on community social and health outcomes will be gathered through administrative data rather than through direct interactions with members of the public. The study will not be collecting data directly from individuals receiving services.

10.1.1.1 Waiver of Consent for Secondary Data Review

The HCS requested a waiver of informed consent and a full Health Insurance Portability and Accountability Act (HIPAA) waiver for use of secondary administrative data. Primary and secondary outcomes will primarily be measured using a retrospective review of secondary administrative data. Individuals whose health care data are included in the secondary administrative data sets will not be contacted at any time during the project. Given the time frame of the study and estimated population sizes of the HCS communities, secondary data will likely include millions of individual health records. The Institutional Review Board (IRB) granted a waiver of consent and a full waiver of HIPAA authorization for secondary data analysis.

10.1.1.2 Informed Consent Process

Researchers will obtain consent from research participants before data collection. When collecting data during in-person interviews or focus groups, researchers will provide eligible subjects with a hard copy of the informed consent form and describe the study objectives and

what will be asked of them if they choose to participate. Enough time will be scheduled to allow them to read the informed consent form and to answer all questions. Participants will be given a copy of the consent form to keep that includes contact information for the local Principal Investigator (PI). Hard copy consent forms will be stored in a secured location. When data collection occurs via web interfaces (REDCap), researchers will contact individuals by email and direct them to an online form that explains the research, the consent process, and steps taken to ensure confidentiality. Individuals will then provide consent as instructed by the online process. Research will not begin until the participant completes the electronic informed consent form. If the participant is contacted by telephone or videoconference to complete an interview, researchers will administer a verbal consent using an approved script that provides all the key information about the study. When administering the verbal consent, study staff will record the respondent name, interviewer name, and interviewer signature on the consent form. Because study staff are working remotely due to the COVID pandemic and may not have access to a hard copy of the verbal consent, research sites have the option of programming these fields (respondent name, interviewer name, and interviewer signature) into REDCap. The data will be stored securely on the site-specific REDCap server, in the same manner as all REDCap data. The study staff's signature is stored as an image file on the same server. The IRB also approved a waiver of documentation of consent; therefore, participant signatures will not be required for verbal or online consent.

10.1.2 Certificate of Confidentiality

This research will be covered by a Certificate of Confidentiality from the National Institutes of Health (NIH). Researchers covered under this Certificate may not disclose personally identifiable information (PII) of any research participant in any federal, state, or local civil, criminal, administrative, legislative, or other action suit. Disclosure of this information is permitted only when:

- required by federal, state, or local laws (e.g., as required by the Federal Food, Drug, and Cosmetic Act, or state laws requiring the reporting of communicable diseases to state and local health departments), excluding instances of disclosure in any federal, state, or local civil, criminal, administrative, legislative, or other proceeding;
- necessary for the medical treatment of the individual to whom the information, document, or biospecimen pertains and made with the consent of such individual;
- made with the consent of the individual to whom the information, document, or biospecimen pertains; or
- made for the purposes of other scientific research that is compliant with applicable federal regulations governing the protection of human subjects in research.

The Certificate does not cover requests for information from personnel of the U.S. federal or state government agency sponsoring the project that is needed for auditing or program evaluation by the National Institute on Drug Abuse (NIDA) and the Substance Abuse and Mental Health Services Administration (SAMHSA). A Certificate of Confidentiality does not prevent an individual from voluntarily releasing information about himself or herself or his or her involvement in the research.

10.1.3 Confidentiality and Privacy

No data that contain identifiers will be shared with anyone outside of approved key personnel affiliated with the HCS. Research sites will assign a subject identification number to each participant and will securely transfer all raw data to the Data Coordinating Center (DCC). Every effort will be made to keep all research records confidential. Information will be combined with information from all study participants. Participants will not be identified in any study materials or reports. The results of this study may be published; however, results will be presented only in aggregate and will not allow for the identification of any individual participant.

The research teams will make every effort to prevent anyone who is not on the research team from accessing any PII. For example, participants' names will be kept separate from the information provided, and these two items will be stored in different places under lock and key. All hard copy research data will be kept in locked file cabinets; electronic data will be password protected and stored on secure servers. The RSs will assign a participant ID number to individual records and maintain the key linking PII with participant ID numbers in an encrypted and password-protected electronic file. Research data will be identified only by participant ID number.

10.1.4 Future Use of Stored Specimens and Data

This study intends to store, use, and share data from surveys and interviews for future research. Having information collected from many people helps researchers identify trends and discover better ways to implement interventions to reduce opioid overdoses. Researchers will use the stored information to research additional scientific questions. The study will prepare a data sharing plan that is described in Section 10.1.10.

10.1.5 Key Roles and Study Governance

The HCS Steering Committee (SC) comprises the PI, a state government official, and one CAB member from each of the four RSs, as well as project scientists from NIDA and SAMHSA, and a chairperson (Table 13). The responsibility of the SC is to give guidance and direction to the overall study design and execution.

Table 13: Key roles and study governance

DCC PIs
Emmanuel Oga, MD, MPH<br>RTI International<br>6110 Executive Boulevard, Suite 900<br>Rockville, MD 20852<br>301-230-4641<br>eoga@rti.org<br><br>Gary Zarkin, PhD<br>RTI International

3040 East Cornwallis Road<br>PO Box 12194<br>Research Triangle Park, NC 27709<br>919-541-5858<br>*gaz@rti.org*<br><br>LaShawn Glasgow, DrPH, MPH<br>RTI International<br>2987 Clairmont Road NE<br>Atlanta, GA 30329<br>770-407-4913<br>*lglasgow@rti.org*
**Kentucky PI**
Sharon Walsh, PhD<br>University of Kentucky<br>845 Angliana Avenue<br>Lexington, KY 40508<br>859-257-6485<br>*sharon.walsh@uky.edu*
**Massachusetts PI**
Jeffrey H. Samet, MD, MA, MPH<br>Boston Medical Center<br>801 Massachusetts Avenue, 2nd Floor<br>Boston, MA 02118<br>617-414-7288<br>*jsamet@bu.edu*
**New York PI**
Nabila El-Bassel, PhD<br>Columbia University School of Social Work<br>1255 Amsterdam Avenue<br>New York, NY 10027<br>212-851-2391<br>*ne5@columbia.edu*

**Ohio PI**
Bridget Freisthler, PhD<br>The Ohio State University<br>530 W. Spring Street, Suite 275<br>Columbus, OH 43215<br>614-292-2856<br>*freisthler.19@osu.edu*
**NIDA Project Scientist**
Redonna Chandler, PhD<br>National Institute on Drug Abuse<br>6001 Executive Boulevard, Room 5265<br>Rockville, MD 20892<br>301-402-1919<br>*redonna.chandler@nih.gov*
**SAMHSA Project Scientist**
Yngvild K. Olsen, MD, MPH<br>Substance Abuse and Mental Health Services Administration<br>5600 Fishers Lane<br>Rockville, MD 20857<br>240-276-0493<br>*yngvild.olsen@samhsa.hhs.gov*
**Chairperson**
Scott Walters, PhD<br>University of North Texas Health Science Center<br>3500 Camp Bowie Boulevard, EAD 709K<br>Fort Worth, TX 76107<br>817-735-2365<br>*scott.walters@unthsc.edu*

10.1.6 Safety Oversight

Safety oversight will be monitored by the Data and Safety Monitoring Board (DSMB), which is composed of individuals with the appropriate expertise, including community-based research, implementation science, epidemiology, biostatistics, bioethics, and opioid use disorder.

Members of the DSMB will be independent from the study conduct and free of conflicts of interest. The DSMB will meet at least semi-annually to assess safety and efficacy data from each arm of the study. The DSMB will operate under an approved Data and Safety Monitoring Plan. The DSMB will provide its recommendations about trial safety to the NIDA Director, who will determine any actions to be taken on the recommendations. The DSMB members are as follows (see Table 14):

Table 14: Data and Safety Monitoring Board members

Name/Title/Organization	Expertise	DSMB Role
Christine Grella, PhD<br>Professor, Department of Psychiatry and Biobehavioral Sciences, David Geffen School of Medicine at University of California, Los Angeles	Substance use disorder (SUD) treatment services, health services research, and longitudinal research on treatment utilization and outcomes	Chair
Celia Fisher, PhD<br>Professor and Director, Fordham University Center for Ethics Education	Ethical principles, racial/ethnic identity, cultural competence, and community-based participatory research	Member
Monica Taljaard, PhD<br>Senior Scientist, Clinical Epidemiology Program, Ottawa Hospital Research Institute	Biostatistics and epidemiology	Member
Vivian Go, PhD<br>Associate Professor, University of North Carolina at Chapel Hill	SUD treatment, implementation science, and opioid/human immunodeficiency virus (HIV)/hepatitis C virus research in rural communities	Member
Margarita Alegría, PhD<br>Chief, Disparities Research Unit, Massachusetts General Hospital	Implementation science, clinical trials, large multi-site studies, community-based participatory research, and qualitative methods	Member

10.1.7 Quality Assurance and Quality Control

Quality assurance (QA) and quality control (QC) are critical to ensure that data are generated, documented, and reported in compliance with the protocol, Good Clinical Practice (GCP), and all other regulatory requirements. QA is process oriented and includes planned, systematic reviews of procedures and standards used to manage and complete project deliverables. QA is prospective and intended to prevent mistakes and avoid problems. QC is product oriented and includes activities to ensure adherence to the protocol, GCP guidelines, and other regulatory requirements. QC occurs in real time and includes standards followed during the completion of project deliverables to ensure that work products are accurate and complete.

QA processes, QC standards, and the related roles and responsibilities of staff will be documented in the DCC and site-specific data management plans (DMPs) and standard operating procedures (SOPs). Specific QA procedures will include the following:

- Reviewing training and certification of staff in GCP and use of data systems
- Tracking study performance metrics
- Conducting internal audits for compliance with DMPs and SOPs

QC standards will be implemented and maintained according to SOPs that cover the following activities:

- Development of the data collection forms and data dictionaries
- Development of an electronic data capture system for measures collected by all sites that includes automated reporting and programmatic edit checks (range checks, logical inconsistencies, missing data) to identify potential errors at the point of entry
- Thorough testing of the data capture system capabilities and functioning, and establishment of change control processes, issue tracking, and load testing
- Development of the data management system following an approved software development life cycle in which requirements are specified, changes to code are controlled, defects are reported and resolved, and systems are tested throughout the life cycle
- Review of secondary data (e.g., from state databases) to ensure accuracy and completeness

10.1.8 Data Handling and Record Keeping

10.1.8.1 Data Collection and Management Responsibilities

Data Collection

Data used for the HCS are derived from primary (de novo) and secondary data collection efforts. The RSs and the DCC will collect the study data. The DCC will establish an electronic data capture system (e.g., REDCap) that all RSs will use for primary data collection activities. The sites will also collect secondary administrative data from various state agencies (e.g., Medicaid, vital records). These data (both primary and secondary administrative data) may include identifiable and protected health information. The RSs will work with one another and the DCC to ensure that data collection procedures, QA, and QC are harmonized across the HCS.

Data for the primary and most secondary study outcomes rely on administrative data, which will be requested directly from various state agencies in each of the RSs. Each RS will develop a study-specific data use agreement with state agencies, which will (1) ensure access to fully identified or limited-use data sets for site-specific analysis and development of a limited and de-identified data set, and (2) allow sites to submit this limited data set to the DCC for the purpose of statistical analysis and dashboard visualization. Brief descriptions of the variables and operational definitions for the calculations of the study outcome measures are included in Appendix A. The data sets required for the calculation of each measure are listed in Table 1 and in Appendix A. The RS research teams will develop data security protocols that will ensure the

collection, storage, and transfer of fully identified and/or limited-use data sets. The DCC will develop a protocol for secure data collection, storage, and transfer for limited data sets that will be received from the sites.

Data Management

The DCC is responsible for developing and maintaining an informatics environment to host and control access to data sets collected by the sites and the DCC. This environment will include a secure SQL-based server with a web interface and technologies (e.g., secure file transfer protocol [SFTP], application programming interfaces, Secure Sockets Layer [SSL] encryption) supporting data ingestion from REDCap and the sites. The development of this environment will be guided by in-house SOPs and follow an applicable software development life cycle framework.

The DCC will be responsible for performing active data management in the informatics environment to ensure the collection of high-quality data and support downstream activities including dashboard display, provision of quality metrics related to the quality management plan, and data analysis and sharing. The data management techniques, detailed in the DMP, will include automated and semi-automated programmatic data checks and production of data reports. As discrepancies are identified, the DCC will notify the sites of the need for corrective action. Audit trails will be retained for documentation purposes.

The sites will be responsible for establishing local, secure, and approved informatics environments to store site-specific primary and secondary data, and for performing relevant data management activities and processes. Each site will work with the DCC to establish a secure method for transmission of data sets to the DCC. State-specific plans are as follows:

- Kentucky: The Kentucky HCS team will provide a robust, secure, and user-friendly informatics platform to manage the data source flow and integration, data modeling and validation, data warehousing, and multiple reporting tools. These tools allow investigators to monitor and interact with project data, including generation of dashboards, reports, and query tools. We use enterprise class data warehousing software and tools including SQL Server, DataStage, and Tableau. The entire system will be housed in the University of Kentucky HealthCare Information Technology secure data center, which includes enterprise network firewalls, Dell SecureWorks Advanced Malware Protection and Detection service, and an annual risk assessment and security audit. Access to protected health information is supported through an encrypted access solution (e.g., virtual private network [VPN]) by qualified investigators with protected virtual machines to manage all sensitive data. (1) Data import. Data will be extracted, transformed, and imported into the Kentucky Health Data Trust staging area using validated SQL scripts and procedures to standardize the imports. (2) Data staging. Scripts will be developed to load the data files into the operational data store SQL Server database. A complete series of data validation and quality checks will be completed to finalize the production data and provide data quality monitoring and feedback. (3) Data repositories. Each specific intervention team will help develop implementation-specific use-case data marts to support implementation monitoring, evaluation, and analysis including the dissemination and sharing of results and data (in standardized formats) with community partners, NIDA, the DCC, the public, and the research community using a

suite of business analytics tools. The Kentucky HCS team will release to the DCC small counts normally subject to suppression rules for analysis purposes only, under the condition that they never be released publicly, including public-use versions of data. The release of Kentucky counts beyond the purpose of HCS statistical analysis (e.g., for publications, reports, dashboards) will follow the Kentucky data reporting policy and small count suppression rules as listed in the Kentucky HCS data use agreements with agencies' data owners.

- Massachusetts: The Massachusetts Department of Public Health has built substantial protections to prevent identification of individuals in the public health data set. Specifically, access to secondary individual-level data is restricted to only death information. For all other secondary state data, only summary statistics are provided, and information is suppressed if there is an insufficient number of individuals for a given query (between 1 and 10 individuals, variable dependent). Massachusetts study staff will obtain secondary state data, working in the virtual environment provided by the Massachusetts Department of Public Health. Primary data containing PII will be stored on virtual machines located in the premium secure environment of the Boston University Medical Campus Information Technology group. A Massachusetts informatics lead will provide usernames and passwords for authorized users to access the study data based on role. All PII will be encrypted on transfer using SSL encryption technology.
- New York: Only de-identified aggregate-level data will be transferred to servers under control of Columbia University Information Technology from partners at the New York State Department of Health. Small counts normally subject to suppression rules will be released for analysis purposes only, under the condition that they never be released publicly, including public-use versions of data. De novo data collection will be stored in REDCap. Both of these types of data will be stored in Columbia Universities' accounts using Google Cloud Platform and BigQuery, Google's serverless enterprise data warehouse. All data and metadata are stored in a multi-redundant, AES256-encrypted data store and leverage Google Cloud Platform infrastructure to manage encryption keys. All data access is logged, and Google Cloud Platform uses industry-leading electronic and physical security controls. Permissions and access to data and metadata will be managed by the front-end discovery platform, Redivis.
- Ohio: All data and analysis of protected health information from state agency sources will reside on and occur on the InnovateOhio Platform (IOP). The IOP is a secure government data environment administered and maintained by the Ohio Department of Administrative Services (DAS), the organization that provides all IT services for the state of Ohio. DAS will be responsible for obtaining and uploading data from all state agencies listed in the data use agreement, automating monthly refreshes, linking records across data sets, and de-identifying resulting records. Ohio state agencies will transfer raw identifiable data to the IOP through SFTP or a direct connection. DAS will link data across agencies and use a token system to generate unique ID numbers for each individual record. These individual-level de-identified data will be placed in a secured workspace on the IOP, behind a secured government firewall. HCS researchers will have access to this workspace through a secured virtual machine. Select study personnel will be issued government ID

numbers and passwords and be allowed to log in to the IOP workspace. No individual-level data will be hosted on Ohio State University servers. County aggregate, de-identified data will be transferred from the IOP to Ohio State University through SFTP. Small numbers, typically subject to suppression, will be released to Ohio State University and transferred to the DCC, but will not be released in public-use data sets. Primary data collected through the REDCap system, and any de-identified data transferred from the IOP, will be housed on the Ohio State University Wexner Medical Center internal network and protected by its enterprise firewall. Application servers and databases are kept physically separate to enhance security. All in-flight communication is secured by transport layer security under the SSL protocol. The databases will also be encrypted at rest to protect them from external attacks. Data accessibility will be controlled by an identity and access management service allowing only authorized users to view or process data.

Secondary data received from state partners consist of de-identified or limited data sets. Secondary data will be transferred from each RS to the DCC and will be stripped of protected health information (PHI). Primary data collection will include PII of coalition members and associated surveys, qualitative interviews, and study documentation (e.g., meeting minutes). Data will be collected by each RS, and data sets containing primary data will be encrypted and transferred from each RS to the DCC.

Each RS and the DCC has a data center that has physical security and enterprise class network firewalls, secure servers, encryption protocols, malware protection and detection services, role-based security protocols, hardware redundancy, data backup policies, and user logging and auditing. Each data center undergoes an annual risk assessment and security audit to ensure compliance. Access to PHI is supported through an encrypted access solution (VPN) by qualified investigators with protected virtual machines to manage all sensitive data.

The DCC has developed a secure environment and a data warehouse to store primary and secondary data that comply with FAIR (Findable, Accessible, Interoperable, Reusable) Guiding Principles and HIPAA. Specifically, collection of metadata will ensure that data and documentation are findable and that metadata, data, and documentation are accessible through the dashboard with links to data sources, documentation, and information of interest. Data will be stored in SQL format, and standard practice for assignment of common geographic identifiers (e.g., nested census-based geographies) and data model will be followed to facilitate interoperability. Metadata, data, and documentation will be reusable because data will be stored in native or raw format, and extract, transform, load logic and variable definitions for derived and analytic data sets will be available.

All HCS investigators and staff who are involved in the design, conduct, oversight, or management of this clinical trial have been or will be trained in GCP.

10.1.8.2 Study Records Retention

Data collected or transmitted to the DCC under this protocol will be maintained at the DCC for at least 3 years from the date of the last annual Federal Financial Report submission.

10.1.9 Protocol Deviations

This protocol defines a protocol deviation as any non-compliance with the clinical trial protocol or SOP requirements. The non-compliance may be on the part of the research participant, the PI, or the study site staff. As a result of deviations, corrective actions will be developed by the RSs and the DCC and implemented promptly.

It will be RS investigator's responsibility to use continuous vigilance to identify and report deviations within 5 working days of identification of the protocol deviation. All deviations will be addressed in study source documents and reported to the NIDA Program Official and the SC. The DCC will send protocol deviations to the single IRB, Advarra, per its policies. The DCC is responsible for knowing and adhering to the IRB's reporting requirements. Further details about the handling of protocol deviations will be included in the SOP.

10.1.10 Publication and Data Sharing Policy

We plan to make study methods, data, and results available to scientists, health policy experts, and members of the public who are interested in reducing the burden of the opioid crisis. We will develop a data sharing plan that describes the process by which study publications and the underlying primary data will be made broadly available to the public. The data sharing plan will comply with the NIH HEAL Initiative℠ Public Access and Data Sharing Policy, the NIH Data Sharing Policy, and the NIH Policy on Dissemination of NIH-Funded Clinical Trial Information, and the NIH Clinical Trial Registration and Results Information Submission rule. As such, this trial will be registered at *ClinicalTrials.gov*, and results information from this trial will be submitted to *ClinicalTrials.gov*.

The data sharing plan will be implemented through five key steps. First, a data management plan will be developed to describe the final data documentation and archival considerations for study data. Second, best practices will be followed for creating, maintaining, and documenting study data to ensure preservation of accurate and usable data and metadata while protecting the privacy of study participants. Third, de-identified data (as specified in the data use agreements) and accompanying documentation will be made readily available to researchers for secondary analysis through a repository selected by NIDA, such as the NIDA-funded National Addiction & HIV Data Archive Program (NAHDAP). NAHDAP is hosted by the Inter-university Consortium for Political and Social Research, the largest social science data archive in the world and part of the University of Michigan's Institute for Social Research. Fourth, NIDA and SAMHSA will be given access to all data generated under this award, subject to rules specified in any Certificates of Confidentiality obtained by the RSs. Fifth, the DCC and the SC will develop a publication policy consistent with the NIH Public Access Policy, which ensures that the public has access to the published results of NIH-funded research and is consistent with the following criteria:

1. Within 4 weeks of acceptance by a journal, electronic copies of publications will be deposited to the digital archive PubMed Central with no embargo period and with proper tagging of metadata to ensure online discoverability and accessibility.
2. Publications will be published under the Creative Commons Attribution 4.0 International generic license (CC BY 4.0) or an equivalent license.

3. To the extent feasible, de-identified data will be made accessible simultaneously with the publication and through NAHDAP.

The data sharing plan will follow NIH requirements for sharing data via the creation of public-use data sets and will apply established procedures to ensure that publicly released data satisfy all necessary requirements. A structured process will be followed to determine the risk of re-identification of people included in public-use data sets based on guidance documents from the U.S. Department of Health and Human Services[75] and HIPAA or related requirements for protecting participant identity. The risk of re-identification will also be quantitatively evaluated using accepted statistical methods.[76,77] Study data deposited into a repository will be de-identified or masked to minimize risks to study participant privacy. The following are examples of procedures typically followed to de-identify and mask the data:

- Replacing site numbers with randomly generated site ID numbers
- Replacing study ID numbers with randomly generated study ID numbers
- Removing or recoding distinguishing parameters, such as dates or specific locations
- Removing verbatim responses or sensitive variables
- Combining subgroups with low frequencies or truncating distributions to ensure a minimum number of observations per category or variation within cells
- Dropping categorical variables, combining levels, or otherwise modifying response categories with less than 5% prevalence or fewer than 10 observations
- Dropping continuous variables with fewer than 10 observations

We plan to share a variety of dissemination products that will emerge from this study including scientific manuscripts and conference presentations describing study methods, findings, content within and utility of supporting materials, and best practices. We plan to present results at national scientific meetings that focus on drug use, public health, and modeling methods (e.g., the Rx Drug Abuse & Heroin Summit, the College on Problems of Drug Dependence, and the Society for Medical Decision Making). Given the national attention and urgency of mitigating the opioid crisis, we aim to publish results in high-impact peer-reviewed medical (e.g., *New England Journal of Medicine*) and specialty (e.g., *Drug and Alcohol Dependence*, *Health Affairs*) journals. Publications resulting from this study will have authorship decided based on contribution to study design, conduct, analysis, and writing per HCS policy and journal guidelines. The HCS publication policy will follow the published policies of the International Committee of Medical Journal Editors and the appropriate use of federal disclaimer and acknowledgment of funding and disclosure statements.

In addition to sharing study data and publications, the HCS modeling tool will be made freely available to the scientific community and policy makers through the Internet (e.g., *www.opioidpolicysimulator.org*). Per the guidelines from the Second Panel on Cost-Effectiveness Analysis and the report on model transparency and validation by the Society for Medical Decision Making and the International Society for Pharmacoeconomics and Outcomes Research, we will present all modeling elements including technical details in future publications. Transparency will also be maintained, so key stakeholders can see how the model is built.

10.2 Abbreviations and Special Terms

AE	adverse event
API	application programming interface
CAB	Community Advisory Board
CBPR	community-based participatory research
CDC	Centers for Disease Control and Prevention
CE	community engagement
CTC	Communities That Care
CTH	Communities That HEAL
DAS	Department of Administrative Services (Ohio)
DCC	Data Coordinating Center
DEA	Drug Enforcement Administration
DMP	data management plan
DSMB	Data and Safety Monitoring Board
DSMP	data and safety monitoring plan
DUA	data use agreement
EBP	evidence-based practice
ED	emergency department
EMS	emergency medical services
FDA	U.S. Food and Drug Administration
GCP	Good Clinical Practice
GEE	general estimating equation
GMB	Group Model Building
HCS	HEALing Communities Study
HCV	hepatitis C virus
HHS	U.S. Department of Health and Human Services
HIPAA	Health Insurance Portability and Accountability Act of 1996
HIV	human immunodeficiency virus

ICD-10-CM	International Statistical Classification of Diseases and Related Health Problems, 10th Revision, Clinical Modification
ICF	informed consent form
ID	Identification
IOP	InnovateOhio Platform
IRB	Institutional Review Board
IS	Information Systems
ITT	intention-to-treat
KY-ASAP	Kentucky Agency for Substance Abuse Policy
LDS	limited data set
MME	morphine milligram equivalent
MOUD	medication for opioid use disorder
NAHDAP	National Addiction & HIV Data Archive Program
NEMSIS	National Emergency Medical Services Information System
NIDA	National Institute on Drug Abuse
NIH	National Institutes of Health
NSDUH	National Survey on Drug Use and Health
OBAT	office-based addiction treatment
OD	Overdose
OEND	overdose education and naloxone distribution
OHRP	Office for Human Research Protections
ORCCA	Opioid-Overdose Reduction Continuum of Care Approach
OTP	opioid treatment program
OUD	opioid use disorder
PI	Principal Investigator
PII	personally identifiable information
QA	quality assurance
QC	quality control
REDCap	Research Electronic Data Capture

RS	Research Site
SAE	serious adverse event
SAMHSA	Substance Abuse and Mental Health Services Administration
SAP	statistical analysis plan
SC	Steering Committee
SFTP	secure file transfer protocol
SOP	standard operating procedure
SSL	Secure Sockets Layer
UP	unanticipated problem

10.3 Protocol Amendment History

Protocol amendments will be recorded in Table 15, which is maintained by the DCC.

Table 15: Protocol Amendment History

Version	Date	Description of Change	Brief Rationale
1.1	10-28-2019	Landscape Analysis (LA) 1 revision	Updated the LA 1 instrument
1.2	11-1-2019	Protocol, informed consent form (ICF), instrument, and subject-facing material updates	Updated the protocol, the majority of the ICFs, and most of the instruments<br>Submitted new instruments, associated ICFs, and subject-facing materials<br>Moved and updated prior approved documents from Pro00037850 to Pro00038088
1.3	11-27-2019	Protocol, ICF, instrument, and subject-facing material updates	Updated the protocol, ICFs, and instruments related to the HCS data collection in jails<br>Updated the protocol and instruments related to the HCS fidelity measures
1.4	12-12-2019	Protocol, ICF, and instrument updates	Updated the protocol to reflect changes made to the health economics instruments and the LA 2 instrument revision<br>Made minor revisions to related ICFs and created a new online ICF for the health economics instruments

Version	Date	Description of Change	Brief Rationale
			Added information in Appendix D of the protocol about GetNaloxoneNow
1.5	1-10-2020	Protocol, ICF, instrument, and subject-facing material updates	Updated the protocol: Appendices A and C<br>Added information on new and revised instruments/ICFs and outcome measures<br>Added information about the evaluation of the communication campaign<br>Made minor revisions to the health economics section
1.6	3-6-2020	Protocol, ICF, instrument, and subject facing material updates	Light edits made throughout the protocol to fix formatting and grammatical errors<br>Updates made to Section 6.1.1 (study intervention)<br>Updates made to Section 6.2 (fidelity)<br>Submitted new subject facing materials for the communications campaign<br>Revisions made to the CEQ instrument and its associated ICFs
1.7	4-7-2020	Protocol, ICF and instrument updates	Edits made throughout the protocol to allow the DCC to receive PII/PHI from the research sites for de novo data<br>Protocol updates made related to the HCS Jail Survey, changing data collection from every six months to annually<br>Protocol updates made to remove information around Dynata, as the Consortium is no longer using Dynata to recruit participants for Campaign Evaluation Questionnaire<br>Protocol updates made to remove the TA and Training Participant Evaluation Form as this instrument is not being used at this time<br>Revisions made to the TTAT instrument<br>Revisions made to 5 web modality ICFs
1.8	5-15-2020	Protocol and subject facing material updates	Protocol updates made to revise hypothesis 3; revisions made to section

Version	Date	Description of Change	Brief Rationale
			3 (objectives and outcomes), section 9.3 (statistical hypotheses), and Appendix A<br>Protocol updates made to section 8.1 (instruments), 8.2 (safety assessments), 8.3 (adverse events), 8.4 (unanticipated problems)<br>Submission of two new generic phone scripts<br>Minor edits made to existing email templates/scripts
1.9	8-26-2020	Protocol updates	Protocol revisions made to section 3 (objectives and outcomes), section 6 (study interventions), section 9 (statistical considerations) and Appendix A<br>Added the MA Site-Specific Brandeis Payer Pre-Interview Survey & Interview Guide research activity to Appendix D<br>Added the KY Site-Specific Academic Detailing and CECentral Online Modules research activity to Appendix D
1.10	10-20-2020	Protocol updates	Protocol revisions made to section 6 (study interventions), section 8 (instruments and procedures) and Appendix D.<br>Added the KY Site-Specific MOUD Organization Interview Guide research activity to Appendix D.<br>Added the NY Site-Specific Pharmacy Study Interview Guide & Survey research activity to Appendix D.
1.11	11-23-2020	Protocol, instrument, and subject facing materials updates	Protocol revisions made to section 6.3.2 (recruitment procedures), section 8 (instruments) and section 10.1.1.2 (informed consent process).<br>Revisions made to the Campaign Evaluation Questionnaire<br>Submission of a MA site-specific recruitment flyer.

Version	Date	Description of Change	Brief Rationale
1.11	01-14-2021	Protocol updates	Protocol revisions made throughout the protocol to capture study timeline changes.
1.12	04-05-2021	Protocol, ICF, instrument, and subject facing material updates	Protocol revisions made to section 6 (study interventions), section 8 (instruments and procedures), and Appendix D.<br>Added the OH Site-Specific CE Facilitator Community Genogram Model research activity to Appendix D.<br>Revisions made to the NY Site-Specific Pharmacy Study research activity in Appendix D.<br>Submission of the OH Site-Specific Community Engagement Facilitator Community Assessment Tool.<br>Submission of the HCS Community Data Dashboards Survey & Portal Group Interview Guide, and their associated verbal ICF and subject facing materials.
1.13	06-17-2021	Protocol and instrument updates	Protocol revisions made to section 6 (study interventions), section 8 (instruments and procedures), and Appendix D.<br>Added the Policy Barriers and Facilitators research activity to Appendix D.<br>Submission of the Policy Community Report and Policy Environmental Scan instruments.
1.14	11-08-2021	Protocol updates	Protocol revisions made throughout the protocol to capture Wave 2 study timeline changes, section 8 (instruments and procedures), and Appendix D.<br>Added Costing the Evidence Based Practices to Appendix D.<br>Added Identifying the Municipal Policies that Impact the Implementation of the EBP Strategies to Appendix D.
1.15	12-10-2021	Protocol and instrument updates	Protocol revisions made to section 6 (study interventions), section 8

Version	Date	Description of Change	Brief Rationale
			(instruments and procedures), and Appendix D.<br>Added Photovoice Participatory Research Technique to Appendix D.<br>Added Race and Ethnicity Data Collection Readiness Survey to Appendix D.<br>Submission of new and revised data collection instruments.
1.16	4-14-2022	Protocol and instrument updates	Protocol revisions made throughout the protocol to capture Wave 2 research activities, section 6 (study interventions), section 8 (instruments and procedures), section 9 (statistical considerations), and Appendix D.<br>Added PARTNER Tool research activity to Appendix D.<br>Added Medication Disposal Program Pharmacy Interviews to Appendix D.<br>Added Mobile Interventions for Increasing Access to Medications for Opioid Use Disorder to Appendix D.
1.17	11-7-2022	Protocol and instrument updates	Protocol revisions made to section 2 (introduction), section 3 (objections and outcomes), section 6 (study interventions), section 8 (instruments and procedures), section 10 (supporting documentation and operational considerations), Appendix A and Appendix D.<br>Added Cost Analysis of Peer Recovery Support Services research activity to Appendix D.<br>Added Wave 1 Partner Organization Sustainability Interview research activity to Appendix D.
1.18	3-30-2023	Protocol updates	Protocol revisions made to the cover page and section 10 (supporting documentation and operational considerations).

Version	Date	Description of Change	Brief Rationale
1.19	8-17-2023	Protocol updates	Protocol revisions were made to add secondary outcome 3.4 and update the OSU research site address in Table 13.
1.20	10-9-2023	Protocol Updates	Minor revisions made to section 6 (study interventions).
1.21	3-6-2024	Protocol Updates	Protocol updates were made to extend the study end date through March 2025 in section 1 (protocol summary) and section 4 (study design).

11. REFERENCES

1. Gostin LO, Hodge JG Jr, Noe SA. Reframing the opioid epidemic as a national emergency. *JAMA*. 2017;318(16):1539-1540. doi:10.1001/jama.2017.13358.

2. Pitt AL, Humphreys K, Brandeau ML. Modeling health benefits and harms of public policy responses to the US opioid epidemic. *Am J Public Health*. 2018;108(10):1394-1400. doi:10.2105/AJPH.2018.304590.

3. Seth P, Scholl L, Rudd RA, Bacon S. Overdose deaths involving opioids, cocaine, and psychostimulants—United States, 2015-2016. *Am J Transplant*. 2018;18(6):1556-1568. doi:10.1111/ajt.14905.

4. National Institute on Drug Abuse (NIDA). Overdose Death Rates. National Institute on Drug Abuse website. https://www.drugabuse.gov/related-topics/trends-statistics/overdose-death-rates. January 29, 2019. Accessed June 18, 2019.

5. Hedegaard H, Minino A, Warner M. Drug overdose deaths in the United States, 1999-2017. NCHS Data Brief. 2018;(329):1-8.

6. Volkow ND, Wargo EM. Overdose prevention through medical treatment of opioid use disorders. *Ann Intern Med*. 2018;169(3):190-192. doi:10.7326/M18-1397.

7. Kolodny A, Courtwright DT, Hwang CS, et al. The prescription opioid and heroin crisis: a public health approach to an epidemic of addiction. *Annu Rev Public Health*. 2015;36:559-574. doi:10.1146/annurev-publhealth-031914-122957.

8. Substance Abuse and Mental Health Services Administration (SAMHSA). Key substance use and mental health indicators in the United States: results from the 2016 National Survey on Drug Use and Health (HHS Publication No. SMA 17-5044, NSDUH Series H-52). https://www.samhsa.gov/data/sites/default/files/NSDUH-FFR1-2016/NSDUH-FFR1-2016.htm. Published September 2017. Accessed February 13, 2020.

9. Substance Abuse and Mental Health Services Administration (SAMHSA). Key substance use and mental health indicators in the United States: results from the 2017 National Survey on Drug Use and Health (HHS Publication No. SMA 18-5068, NSDUH Series H-53). https://www.samhsa.gov/data/sites/default/files/cbhsq-

reports/NSDUHFFR2017/NSDUHFFR2017.pdf. Published September 2018. Accessed February 13, 2020.

10. Wu LT, Zhu H, Swartz MS. Treatment utilization among persons with opioid use disorder in the United States. *Drug Alcohol Depend*. 2016;169:117-127. doi:10.1016/j.drugalcdep.2016.10.015.

11. Larochelle MR, Bernson D, Land T, et al. Medication for opioid use disorder after nonfatal opioid overdose and association with mortality: a cohort study. *Ann Intern Med*. 2018;169(3):137-145. doi:10.7326/M17-3107.

12. Ali MM, Teich JL, Mutter R. The role of perceived need and health insurance in substance use treatment: implications for the Affordable Care Act. *J Subst Abuse Treat*. 2015;54:14-20. doi:10.1016/j.jsat.2015.02.002.

13. Olsen Y, Sharfstein JM. Confronting the stigma of opioid use disorder—and its treatment. *JAMA*. 2014;311(14):1393-1394. doi:10.1001/jama.2014.2147.

14. Corrigan P, Schomerus G, Shuman V, et al. Developing a research agenda for understanding the stigma of addictions part I: lessons from the mental health stigma literature. *Am J Addict*. 2017;26(1):59-66. doi:10.1111/ajad.12458.

15. Jones CM, Campopiano M, Baldwin G, McCance-Katz E. National and state treatment need and capacity for opioid agonist medication-assisted treatment. *Am J Public Health*. 2015;105(8):e55-e63. doi:10.2105/AJPH.2015.302664.

16. Kraus ML, Alford DP, Kotz MM, et al. Statement of the American Society of Addiction Medicine Consensus Panel on the use of buprenorphine in office-based treatment of opioid addiction. *J Addict Med*. 2011;5(4):254-263. doi:10.1097/ADM.0b013e3182312983.

17. Knudsen HK, Havens JR, Lofwall MR, Studts JL, Walsh SL. Buprenorphine physician supply: Relationship with state-level prescription opioid mortality. *Drug Alcohol Depend*. 2017;173(Suppl 1):S55-S64. doi:10.1016/j.drugalcdep.2016.08.642.

18. Alderks CE. Trends in the use of methadone, buprenorphine, and extended-release naltrexone at substance abuse treatment facilities: 2003-2015 (update). The CBHSQ Report. https://www.samhsa.gov/data/sites/default/files/report_3192/ShortReport-3192.pdf. Published August 22, 2017. Accessed February 13, 2020.

19. Morgan JR, Schackman BR, Leff JA, Linas BP, Walley AY. Injectable naltrexone, oral naltrexone, and buprenorphine utilization and discontinuation among individuals treated for opioid use disorder in a United States commercially insured population. *J Subst Abuse Treat*. 2018;85:90-96. doi:10.1016/j.jsat.2017.07.001.

20. Xu J, Davis CS, Cruz M, Lurie P. State naloxone access laws are associated with an increase in the number of naloxone prescriptions dispensed in retail pharmacies. *Drug Alcohol Depend*. 2018;189:37-41. doi:10.1016/j.drugalcdep.2018.04.020.

21. Moulton LH. Covariate-based constrained randomization of group-randomized trials. *Clin Trials*. 2004;1(3):297-305. doi:10.1191/1740774504cn024oa.

22. Ivers NM, Halperin IJ, Barnsley J, et al. Allocation techniques for balance at baseline in cluster randomized trials: a methodological review. *Trials*. 2012;13:120. doi:10.1186/1745-6215-13-120.

23. Hawkins JD. Communities That Care: risk-focused prevention using the social development strategy. Seattle, WA: Developmental Research and Programs; 1993.

24. Hawkins JD, Catalano RF Jr. Communities That Care: action for drug abuse prevention. Jossey-Bass; 1992.

25. Hawkins JD, Catalano RF, Arthur MW, et al. Testing Communities That Care: the rationale, design and behavioral baseline equivalence of the community youth development study. *Prev Sci*. 2008;9(3):178-190. doi:10.1007/s11121-008-0092-y.

26. Newman SD, Andrews JO, Magwood GS, Jenkins C, Cox MJ, Williamson DC. Community advisory boards in community-based participatory research: a synthesis of best processes. *Prev Chronic Dis*. 2011;8(3):A70.

27. Robert Wood Johnson Foundation (RWJF). Data for Health initiative: learning what works. https://www.rwjf.org/en/library/research/2015/04/data-for-health-initiative.html. Published April 2, 2015. Accessed February 13, 2020.

28. Williams AR, Nunes EV, Bisaga A, Levin FR, Olfson M. Development of a cascade of care for responding to the opioid epidemic. *Am J Drug Alcohol Abuse*. 2019;45(1):1-10. doi:10.1080/00952990.2018.1546862.

29. Darke S, Ross J, Hall W. Overdose among heroin users in Sydney, Australia: I. Prevalence and correlates of non-fatal overdose. *Addiction*. 1996;91(3):405-411.

30. Darke S, Williamson A, Ross J, Teesson M. Non-fatal heroin overdose, treatment exposure and client characteristics: findings from the Australian Treatment Outcome Study (ATOS). *Drug Alcohol Rev*. 2005;24(5):425-432. doi:10.1080/09595230500286005.

31. McGregor C, Darke S, Ali R, Christie P. Experience of non-fatal overdose among heroin users in Adelaide, Australia: circumstances and risk perceptions. *Addiction*. 1998;93(5):701-711.

32. Kerr T, Fairbairn N, Tyndall M, et al. Predictors of non-fatal overdose among a cohort of polysubstance-using injection drug users. *Drug Alcohol Depend*. 2007;87(1):39-45. doi:10.1016/j.drugalcdep.2006.07.009.

33. Tsui JI, Evans JL, Lum PJ, Hahn JA, Page K. Association of opioid agonist therapy with lower incidence of hepatitis c virus infection in young adult injection drug users. *JAMA Intern Med*. 2014;174(12):1974-1981. doi:10.1001/jamainternmed.2014.5416.

34. MacArthur GJ, Minozzi S, Martin N, et al. Opiate substitution treatment and HIV transmission in people who inject drugs: systematic review and meta-analysis. *BMJ*. 2012;345:e5945. doi:10.1136/bmj.e5945.

35. Hagan H, Pouget ER, Des Jarlais DC. A systematic review and meta-analysis of interventions to prevent hepatitis C virus infection in people who inject drugs. *J Infect Dis*. 2011;204(1):74-83. doi:10.1093/infdis/jir196.

36. Turner KME, Hutchinson S, Vickerman P, et al. The impact of needle and syringe provision and opiate substitution therapy on the incidence of hepatitis C virus in injecting drug users: pooling of UK evidence. *Addiction*. 2011;106(11):1978-1988. doi:10.1111/j.1360-0443.2011.03515.x.

37. Powers KI, Anglin MD. A differential assessment of the cumulative versus stabilizing effect of methadone maintenance treatment. *Eval Rev*. 1998;22(2):175-206. doi:10.1177/0193841X9802200202.

38. Feelemyer JP, Jarlais DCD, Arasteh K, Phillips BW, Hagan H. Changes in quality of life (WHOQOL-BREF) and addiction severity index (ASI) among participants in opioid substitution treatment (OST) in low and middle income countries: an international systematic review. *Drug Alcohol Depend*. 2014;134:251-258. doi:10.1016/j.drugalcdep.2013.10.011.

39. Murphy SM, Polsky D. Economic evaluations of opioid use disorder interventions. *Pharmacoeconomics*. 2016;34(9):863-887. doi:10.1007/s40273-016-0400-5.

40. D'Onofrio G, Chawarski MC, O'Connor PG, et al. Emergency department-initiated buprenorphine for opioid dependence with continuation in primary care: outcomes during and after intervention. *J Gen Intern Med*. 2017;32(6):660-666. doi:10.1007/s11606-017-3993-2.

41. Liebschutz JM, Crooks D, Herman D, et al. Buprenorphine treatment for hospitalized, opioid-dependent patients: a randomized clinical trial. *JAMA Intern Med*. 2014;174(8):1369-1376. doi:10.1001/jamainternmed.2014.2556.

42. Proctor SL, Copeland AL, Kopak AM, Herschman PL, Polukhina N. A naturalistic comparison of the effectiveness of methadone and two sublingual formulations of buprenorphine on maintenance treatment outcomes: findings from a retrospective multisite study. *Exp Clin Psychopharmacol*. 2014;22(5):424-433. doi:10.1037/a0037550.

43. Schuman-Olivier Z, Weiss RD, Hoeppner BB, Borodovsky J, Albanese MJ. Emerging adult age status predicts poor buprenorphine treatment retention. *J Subst Abuse Treat*. 2014;47(3):202-212. doi:10.1016/j.jsat.2014.04.006.

44. Soeffing JM, Martin LD, Fingerhood MI, Jasinski DR, Rastegar DA. Buprenorphine maintenance treatment in a primary care setting: outcomes at 1 year. *J Subst Abuse Treat*. 2009;37(4):426-430. doi:10.1016/j.jsat.2009.05.003.

45. Proctor SL, Copeland AL, Kopak AM, Hoffmann NG, Herschman PL, Polukhina N. Predictors of patient retention in methadone maintenance treatment. *Psychol Addict Behav*. 2015;29(4):906-917. doi:10.1037/adb0000090.

46. Coulter A, Stilwell D, Kryworuchko J, Dolan Mullen P, Ng CJ, van der Weijden T. A systematic development process for patient decision aids. *BMC Med Inform Decis Mak*. 2013;13(Suppl 2):S2. doi:10.1186/1472-6947-13-S2-S2.

47. O'Conner AM, Jacobsen MJ. Workbook on developing and evaluating patient decision aids. www.ohri.ca/decisionaid. Published 2003. Accessed February 13, 2020.

48. Lefebvre RC. Social marketing and social change: strategies and tools for improving health, well-being, and the environment. San Francisco: Jossey-Bass; 2013.

49. McCombs, M. Setting the agenda: The mass media and public opinion (2nd ed). Malden, MA: Polity Press; 2014.

50. Snyder LB. Health communication campaigns and their impact on behavior. *J Nutr Educ Behav*. 2007;39(2, Suppl):S32-S40. doi:10.1016/j.jneb.2006.09.004.

51. Wakefield MA, Loken B, Hornik RC. Use of mass media campaigns to change health behaviour. *Lancet*. 2010;376(9748):1261-1271. doi:10.1016/S0140-6736(10)60809-4.

52. Huo J, Desai R, Hong YR, Turner K, Mainous AG, Bian J. Use of social media in health communication: findings from the Health Information National Trends Survey 2013, 2014, and 2017. *Cancer Control*. 2019. doi:10.1177/1073274819841442.

53. Ramo DE, Prochaska JJ. Broad reach and targeted recruitment using Facebook for an online survey of young adult substance use. *J Med Internet Res*. 2012;14(1):e28. doi:10.2196/jmir.1878

54. Batterham PJ. Recruitment of mental health survey participants using Internet advertising: content, characteristics and cost effectiveness. *Int J Methods Psychiatr Res*. 2014;23:184-191. doi:10.1002/mpr.1421.

55. Akers L, Gordon JS. Using Facebook for large-scale online randomized clinical trial recruitment: effective advertising strategies. *J Med Internet Res*. 2018;20(11):e290. doi:10.2196/jmir.9372.

56. Healthy People 2020. https://www.healthypeople.gov/. Accessed January 8, 2020.

57. Greenberg-Worisek AJ, Kurani S, Finney Rutten LJ, Blake KD, Moser RP, Hesse BW. Tracking Healthy People 2020 Internet, broadband, and mobile device access goals: an update using data from the Health Information National Trends Survey. *J Med Internet Res*. 2019;21(6):e13300. doi:10.2196/13300.

58. Slater MD, Kelly KJ. Testing alternative explanations for exposure effects in media campaigns: the case of a community-based, in-school media drug prevention project. *Commun Res*. 2002;29(4):367-389.

59. Brewer NT, Hall MG, Lee JG, Peebles K, Noar SM, Ribisl KM. Testing warning messages on smokers' cigarette packages: a standardised protocol. *Tob Control*. 2015. doi:10.1136/tobaccocontrol-2014-051661.

60. Livingston JD, Tugwell A, Korf-Uzan K, Cianfrone M, Coniglio C. Evaluation of a campaign to improve awareness and attitudes of young people towards mental health issues. *Soc Psychiatry Psychiatr Epidemiol*. 2013;48(6):965-973.

61. Griffiths KM, Christensen H, Jorm AF. Predictors of depression stigma. *BMC Psychiatry*. 2008;8(25). doi:10.1186/1471-244X-8-25.

62. Lefebvre RC, Squiers L, Adams E, Nyblade L, West S, Bann C. Stigma and prescription opioid addiction and treatment: a national survey. Annals of Behavioral Medicine, 2019; 53(Suppl 1), S401. https://doi.org/10.1093/abm/kaz007

63. Saunders RP, Pate RR, Felton G, et al. Development of questionnaires to measure psychosocial influences on children’s physical activity. *Prev Med*. 1997;26(2):241-247.

64. Zimmerman L, Lounsbury DW, Rosen CS, Kimerling R, Trafton JA, Lindley SE. Participatory system dynamics modeling: increasing stakeholder engagement and precision to improve implementation planning in systems. *Adm Policy Ment Health*. 2016;43:834-849.

65. Glasgow RE, Harden SM, Gaglio B, et al. RE-AIM planning and evaluation framework: adapting to new science and practice with a 20-year review. *Front Public Health*. 2019;7:64. doi:10.3389/fpubh.2019.00064.

66. Aarons GA, Hurlburt M, Horwitz SM. Advancing a conceptual model of evidence-based practice implementation in public service sectors. *Adm Policy Ment Health*. 2011;38(1):4-23.

67. Damschroder LJ, Aron DC, Keith RE, et al. Fostering implementation of health services research findings into practice: a consolidated framework for advancing implementation science. *Implement Sci*. 2009;4:50. doi:10.1186/1748-5908-4-50.

68. Greenhalgh T, Robert G, Macfarlane F, Bate P, Kyriakidou O. Diffusion of innovations in service organizations: systematic review and recommendations. *Milbank Q*. 2004;82:581-629. doi:10.1111/j.0887-378X.2004.00325.x

69. Kitson AL, Rycroft-Malone J, Harvey G, McCormack B, Seers K, Titchen A. Evaluating the successful implementation of evidence into practice using the PARiHS framework: theoretical and practical challenges. *Implement Sci*. 2008;3:1. doi:10.1186/1748-5908-3-1.

70. Raghavan R, Bright CL, Shadoin AL. Toward a policy ecology of implementation of evidence-based practices in public mental health settings. *Implement Sci*. 2008;3:26. doi:10.1186/1748-5908-3-26.

71. Tabak RG, Khoong EC, Chambers DA, Brownson RC. Bridging research and practice: models for dissemination and implementation research. *Am J Prev Med*. 2012;43(3):337-350. doi:10.1016/j.amepre.2012.05.024.

72. Rhode Island Department of Health. Rhode Island Enhanced State Opioid Overdose Surveillance (ESOOS) case definition for Emergency Medical Services (EMS) (updated). *http://health.ri.gov/publications/guidelines/ESOOSCaseDefinitionForEMS.pdf.* Published March 2019. Accessed August 14, 2019.

73. Schafer, J. L., & Graham, J. W. (2002). Missing data: our view of the state of the art. *Psychological Methods*, *7*(2), 147–177.

74. National Research Council (U.S.) Panel on Handling Missing Data in Clinical Trials. The prevention and treatment of missing data in clinical trials. Washington, DC: National Academies Press; 2010. doi:10.17226/12955.

75. Moher D, Hopewell S, Schulz KF, et al. CONSORT 2010 explanation and elaboration: updated guidelines for reporting parallel group randomised trials. *BMJ*. 2010;340:c869. doi:10.1136/bmj.c869.

76. U.S. Department of Health and Human Services, Office of Civil Rights. Guidance regarding methods for de-identification of protected health information in accordance with the Health Insurance Portability and Accountability Act (HIPAA) Privacy Rule. https://www.hhs.gov/sites/default/files/ocr/privacy/hipaa/understanding/coveredentities/De-identification/hhs_deid_guidance.pdf. Published November 2012. Accessed November 30, 2018.

77. Benitez K, Malin B. Evaluating re-identification risks with respect to the HIPAA privacy rule. *J Am Med Inform Assoc.* 2010;17(2):169-177. doi:10.1136/jamia.2009.000026.

78. Dillman DA, Smyth JD, Christian LM. *Internet, Phone, Mail and Mixed-Mode Surveys: The Tailored Design Method*. 4th ed. Hoboken, NJ: Wiley; 2014.

12. APPENDICES

12.1 Appendix A: Study Outcomes

12.1.1 Primary Outcomes

1	**Definition Number**	1
2	**Definition Name**	Number of opioid overdose deaths
3	**Population**	General population/all residents
4	**Data Sources**	The primary data source will be death certificate records. Additional data sources (e.g., medical examiner and/or coroner data) may be used to identify opioid involvement in certified drug overdose deaths when the death certificates do not list any specific drugs involved in the overdose death. Because of differences in the medicolegal death investigation systems across the jurisdictions, the data sources cannot be standardized, but the process for capturing opioid overdose deaths developed by each site or jurisdiction will ensure that high-quality measures for opioid overdose deaths will be captured consistently in each jurisdiction, allowing harmonization across the HEALing Communities Study (HCS) communities and sites.
5	**Other Available Data for Additional or State-Specific Analyses**	Other data sources (e.g., medical examiner and coroner reports, post-mortem toxicology records, law enforcement reports) will be used to supplement death certificate records for identification of opioid contribution to drug overdose deaths when needed
6	**Primary Outcome Measure**	Number (count) of HCS community resident overdose deaths (i.e., deaths with an underlying cause of drug poisoning) where opioids were determined to be contributing (alone or in combination with other drugs) to the drug poisoning
7	**Population Size Adjustment**	The population size estimate for each HCS community will be the mid-year population estimate as of July 1 for the calendar year under surveillance. This population size estimate will be utilized in the primary analysis as an offset (for details, see the study protocol)
8	**Covariates**	The Hypothesis 1 (H1) analysis will control for the following community-level covariates: rural/urban classification, HCS site, and baseline rates of opioid overdose deaths in HCS communities (based on the 12 months preceding the study initiation)

9	**Additional Analyses**	Stratified by intent of the death (unintentional, suicide, undetermined), gender (male, female), race (white, black, other), and drugs or drug classes contributing to the opioid overdose death (at minimum—heroin, fentanyl, methamphetamine, cocaine, stimulants, benzodiazepines) (drugs or drug classes are not mutually exclusive)

12.1.2 Secondary Outcomes

1	**Definition Number**	2.1
2	**Definition Name**	Number of drug overdose deaths
3	**Population**	General population/all residents
4	**Data Sources**	Death certificates
5	**Other Available Data for Additional or State-Specific Analyses**	Variety of data sources (including death certificates and medical examiner and/or coroner data) allowing accurate and timely capturing of overdose deaths for HCS community residents and harmonization across the HCS sites
6	**Primary Outcome Measure**	Number of overdose deaths
7	**Population Size Adjustment**	The denominator would be the mid-year population for the county or local community
8	**Subgroups**	In addition to the count of all drug overdose deaths, we would like to examine the counts of death by various drug subtypes, including opioids, heroin, fentanyl, cocaine, psychostimulants, benzodiazepines, stimulants, and others, alone or in combination

1	**Definition Number**	2.2
2	**Definition Name**	Number of non-fatal drug overdose events
3	**Population**	General population/all residents
4	**Data Sources**	A combination of state hospital inpatient billing claims and emergency department (ED) billing claims
5	**Other Available Data for Additional or State-Specific Analyses**	Syndromic surveillance, emergency medical services (EMS), and Medicaid data may be a common source across states; Massachusetts and New York have all-payer claims data
6	**Primary Outcome Measure**	Hospital or ED discharge records for HCS residents with a discharge status different from death and codes in the International Statistical Classification of Diseases and Related

		Health Problems, 10th Revision, Clinical Modification (ICD-10-CM) for drug overdose in any discharge diagnosis field
7	**Population Size Adjustment**	Mid-year population estimate

1	**Definition Number**	2.3
2	**Definition Name**	Number of non-fatal opioid overdose events
3	**Population**	General population/all residents
4	**Data Sources**	A combination of state hospital inpatient billing claims and ED billing claims
5	**Other Available Data for Additional or State-Specific Analyses**	Syndromic surveillance, EMS, and Medicaid data may be a common source across states; Massachusetts and New York have all-payer claims data
6	**Primary Outcome Measure**	Hospital or ED discharge records for HCS residents with a discharge status different from death and any ICD-10-CM codes for opioid-related overdose in any discharge diagnosis field
7	**Population Size Adjustment**	Mid-year population estimate

1	**Definition Number**	2.4
2	**Definition Name**	Number of individuals with OUD (prevalence)
3	**Population**	Medicaid beneficiaries
4	**Data Sources**	Medicaid claims
5	**Other Available Data for Additional or State-Specific Analyses**	Massachusetts and New York have all-payer claims databases; Ohio will combine Medicaid claims with claims from the Ohio Department of Mental Health and Addiction Services (OhioMHAS), the New York Office of Alcoholism and Substance Abuse Services (OASAS), and the Massachusetts Bureau of Substance Addiction Services (BSAS)
6	**Primary Outcome Measure**	Individuals with a claim during the 12-month period coded with an ICD-10-CM diagnosis code of opioid use disorder
7	**Population Size Adjustment**	Number of Medicaid beneficiaries enrolled during the 12-month study period for full benefit

1	**Definition Number**	2.5.1

2	**Definition Name**	Number of individuals receiving buprenorphine products that are approved by the Food and Drug Administration (FDA) for treatment of OUD
3	**Population**	General
4	**Data Sources**	Prescription drug monitoring program
5	**Other Available Data for Additional or State-Specific Analyses**	Claims (Medicaid in Kentucky; all-payer in Massachusetts and New York, combined Medicaid and OhioMHAS in Ohio; OASAS in New York)
6	**Primary Outcome Measure**	Count of number of unique individuals receiving buprenorphine MOUD during the measurement period
7	**Population Size Adjustment**	Mid-year population in the county or local community
8	**Covariates**	The H3 analysis will control for the following community-level covariates: rural/urban classification, HCS site, and baseline rates

1	**Definition Number**	2.5.2
2	**Definition Name**	Number of individuals receiving methadone
3	**Population**	Medicaid beneficiaries
4	**Data Sources**	Medicaid administrative data
5	**Other Available Data for Additional or State-Specific Analyses**	Claims (Medicaid in Kentucky, all-payer in Massachusetts and New York, combined Medicaid and OhioMHAS in Ohio; OASAS in New York; BSAS in Massachusetts
6	**Primary Outcome Measure**	Count of individuals receiving methadone as MOUD during the measurement period
7	**Population Size Adjustment**	Count of HCS residents who are Medicaid enrollees aged 18–64, with OUD receiving MOUD during the measurement period

1	**Definition Number**	2.5.3
2	**Definition Name**	Number of individuals receiving naltrexone
3	**Population**	Medicaid beneficiaries
4	**Data Sources**	Medicaid administrative data
5	**Other Available Data for Additional or**	All-payer claims (Massachusetts)

	**State-Specific Analyses**	
6	**Primary Outcome Measure**	Count of individuals receiving naltrexone as MOUD during the measurement period
7	**Population Size Adjustment**	Count of HCS residents who are Medicaid enrollees aged 18–64, with OUD receiving MOUD during the measurement period

1	**Definition Number**	2.5.4
2	**Definition Name**	Number of individuals with OUD receiving MOUD
3	**Population**	Medicaid beneficiaries
4	**Data Sources**	Medicaid administrative data
5	**Other Available Data for Additional or State-Specific Analyses**	All-payer claims (Massachusetts and New York); Ohio can combine claims from Medicaid and OhioMHAS; OASAS in New York; BSAS in Massachusetts
6	**Primary Outcome Measure**	Count of HCS residents who are Medicaid enrollees aged 18–64, with OUD receiving MOUD during the measurement period
7	**Population Size Adjustment**	Number of member-months enrolled during the 12-month study period for full-benefit Medicaid enrollees, aged 18–64, who were identified as individuals with OUD

1	**Definition Number**	2.6
2	**Definition Name**	Number of individuals with OUD receiving behavioral health treatment
3	**Population**	Medicaid beneficiaries
4	**Data Sources**	Medicaid administrative data
5	**Other Available Data for Additional or State-Specific Analyses**	All-payer claims (Massachusetts and New York); Ohio can combine claims from Medicaid and OhioMHAS; OASAS in New York; BSAS in Massachusetts
6	**Primary Outcome Measure**	2.6.1: Number of individuals with OUD receiving behavioral health treatment (inpatient, ASAM levels 3,4)<br>2.6.2: Number of individuals with OUD receiving behavioral health treatment (IOP, ASAM level 2)<br>2.6.3: Number of individuals with OUD receiving behavioral health treatment (outpatient, ASAM level 1)<br>2.6.4: Number of individuals with OUD receiving behavioral health treatment (any of ASAM levels 1–4)

		2:6:5 Number of individuals with OUD receiving behavioral health treatment (any of ASAM levels 1–4)<br>2.6.6: Number of individuals with OUD receiving behavioral health treatment (peer support)<br>2.6.7: Number of individuals with OUD receiving behavioral health treatment (any of case management, peer support)<br>2.6.8: Number of individuals with OUD receiving behavioral health treatment (screening)
7	**Population Size Adjustment**	Medicaid enrollees with a diagnosis of OUD and a claim for MOUD during the measurement period, recorded during any health service encounter (inpatient, outpatient, office visit, ED, etc.)

1	**Definition Number**	2.7.1
2	**Definition Name**	Number of individuals receiving buprenorphine/naloxone retained beyond 6 months
3	**Population**	General
4	**Data Sources**	Prescription drug monitoring programs
5	**Other Available Data for Additional or State-Specific Analyses**	Claims (Medicaid in Kentucky; all-payer in Massachusetts and New York, Medicaid/OhioMHAS in Ohio; OASAS in New York)
6	**Primary Outcome Measure**	Individuals who maintained continual MOUD for 6 months in the measurement period. Continuous treatment is calculated based on days' supply and dosage, and an individual can have a gap of no more than 7 days' treatment to be considered in continual treatment.
7	**Population Size Adjustment**	Mid-year population in the county or local community

1	**Definition Number**	2.7.2
2	**Definition Name**	Number of individuals receiving methadone retained beyond 6 months
3	**Population**	Medicaid beneficiaries
4	**Data Sources**	Medicaid beneficiaries
5	**Other Available Data for Additional or State-Specific Analyses**	All-payer claims in Massachusetts and New York; Ohio can combine claims from Medicaid and OhioMHAS; BSAS in Massachusetts

6	**Primary Outcome Measure**	Individuals who maintained continual methadone MOUD for 6 months in the measurement period. Continuous treatment is calculated based on days' supply and dosage, and an individual can have a gap of no more than 7 days' treatment to be considered in continual treatment.
7	**Population Size Adjustment**	Medicaid enrollees with a diagnosis of OUD and a methadone claim during the first half of the measurement period—allowing for a full 6 months of measurement to occur after the initial claim

1	**Definition Number**	2.7.3
2	**Definition Name**	Number of individuals receiving naltrexone retained beyond 6 months
3	**Population**	Medicaid beneficiaries
4	**Data Sources**	Medicaid beneficiaries
5	**Other Available Data for Additional or State-Specific Analyses**	All-payer data in Massachusetts
6	**Primary Outcome Measure**	Individuals who maintained continual naltrexone MOUD for 6 months in the measurement period. Continuous treatment is calculated based on days' supply and dosage, and an individual can have a gap of no more than 7 days' treatment to be considered in continual treatment.
7	**Population Size Adjustment**	Medicaid enrollees with a diagnosis of OUD and a naltrexone claim during the first half of the measurement period—allowing for a full 6 months of measurement to occur after the initial claim

1	**Definition Number**	2.7.4
2	**Definition Name**	Number of individuals with MOUD retained in treatment beyond 6 months
3	**Population**	Medicaid
4	**Data Sources**	Medicaid administrative data
5	**Other Available Data for Additional or State-Specific Analyses**	All-payer claims in Massachusetts; BSAS in Massachusetts
6	**Primary Outcome Measure**	Individuals who, by (1) prescription MOUD, (2) office-administered MOUD, or (3) a combination of both, maintained continual MOUD for 6 months in the measurement period.

		Continuous treatment is calculated based on days' supply and dosage, and an individual can have a gap of no more than 7 days' treatment to be considered in continual treatment.
7	**Population Size Adjustment**	Medicaid enrollees with an OUD diagnosis, an MOUD claim during the measurement period, and continuous eligibility for 6 months after the first MOUD, concluding before the end of the measurement period

1	**Definition Number**	2.7.5
2	**Definition Name**	Person-months in MOUD
3	**Population**	Medicaid
4	**Data Sources**	Medicaid administrative data
5	**Other Available Data for Additional or State-Specific Analyses**	All-payer claims in Massachusetts; OASAS in New York, Ohio can combine claims from Medicaid and OhioMHAS; BSAS in Massachusetts
6	**Primary Outcome Measure**	Identify all individuals with an OUD diagnosis who received MOUD during the measurement period.<br>For each individual, calculate the number of person-months during the measurement period that the individual maintained continuous MOUD by (1) prescription MOUD, (2) office-administered MOUD, or (3) a combination of both. Continuous treatment is calculated based on days' supply and dosage, and an individual can have a gap of no more than 7 days' treatment to be considered in continual treatment.<br>Outcome measure at the HCS community level is the total number of person-months in continuous MOUD.
7	**Population Size Adjustment**	Number of member-months enrolled during the 12-month study period for full-benefit Medicaid enrollees, aged 18–64, who were identified as individuals with OUD

1	**Definition Number**	2.8.1
2	**Definition Name**	Number of emergency medical services (EMS) naloxone administration events
3	**Population**	An emergency-response patient-EMS encounter
4	**Data Sources**	EMS run data, typically collected by the state's board of EMS as part of national reporting to NEMSIS (*https://nemsis.org/technical-resources/*)

5	**Other Available Data for Additional or State-Specific Analyses**	N/A
6	**Primary Outcome Measure**	Number of EMS runs that involve naloxone administration
7	**Population Size Adjustment**	Mid-year population estimate

1	**Definition Number**	2.8.2
2	**Definition Name**	Number of EMS runs for opioid-related incidents/overdoses
3	**Population**	Any emergency-response patient-EMS encounter
4	**Data Sources**	EMS run data, typically collected by the state's board of EMS as part of national reporting to NEMSIS (*https://nemsis.org/technical-resources/*)
5	**Other Available Data for Additional or State-Specific Analyses**	N/A
6	**Primary Outcome Measure**	Number of EMS runs for opioid-related incidents or overdoses
7	**Population Size Adjustment**	Mid-year population estimate

1	**Definition Number**	2.9
2	**Definition Name**	Number of individuals linked to MOUD after opioid overdose
3	**Population**	Medicaid beneficiaries
4	**Data Sources**	Medicaid claims
5	**Other Available Data for Additional or State-Specific Analyses**	All-payer claims in Massachusetts and New York; Ohio can combine claims from Medicaid and OhioMHAS; BSAS in Massachusetts; prescription drug monitoring programs; EMS and hospital discharge data for Massachusetts
6	**Primary Outcome Measure**	2.9.1.: Count of individuals with ≥1 claim for naltrexone, methadone maintenance treatment, or buprenorphine within 30 days of ED or inpatient discharge<br>2.9.2: Count of overdose events with ≥1 claim for naltrexone, methadone maintenance treatment, or buprenorphine within 30 days of ED or inpatient discharge

7	**Population Size Adjustment**	2.9.1: Number of full-benefit Medicaid enrollees with an opioid overdose<br>2.9.2: Total number of opioid overdose events among full-benefit Medicaid enrollees

1	**Definition Number**	2.10
2	**Definition Name**	Number of individuals linked to MOUD after release from prison
3	**Population**	Imprisoned inmates released to HCS communities
4	**Data Sources**	State departments of corrections linked to Medicaid claims
5	**Other Available Data for Additional or State-Specific Analyses**	Departments of corrections data linked to all-payer claims, where available; OASAS in New York; BSAS in Massachusetts; prescription drug monitoring program in Massachusetts
6	**Primary Outcome Measure**	Number of individuals who reside in HCS communities who receive a first dose of MOUD within 2 and 4 weeks of release from prison as identified by Medicaid claims
7	**Population Size Adjustment**	Imprisoned inmates released to HCS communities

1	**Definition Number**	2.11
2	**Definition Name**	Number of individuals provided MOUD while in jail
3	**Population**	Jailed (pre-trial and sentenced) inmates at jails associated with each HCS community (if more than one, limit to top three jails)
4	**Data Sources**	Survey of jails associated with each HCS community
5	**Other Available Data for Additional or State-Specific Analyses**	Linkage of Massachusetts jail releases to the prescription drug monitoring program in Massachusetts; all-payer claims data and BSAS data
6	**Primary Outcome Measure**	Number of individuals who received MOUD in community jails
7	**Population Size Adjustment**	Number of individuals were offenders in community jails

1	**Definition Number**	2.12
2	**Definition Name**	Number of individuals linked to MOUD after an opioid-related ED visit
3	**Population**	Medicaid beneficiaries
4	**Data Sources**	Medicaid claims
5	**Other Available Data for Additional or State-Specific Analyses**	All-payer claims in Massachusetts and New York; Ohio can combine claims from Medicaid and OhioMHAS; hospital/ED discharge data from Massachusetts; prescription drug monitoring data from Massachusetts; BSAS data from Massachusetts
6	**Primary Outcome Measure**	≥1 claim for naltrexone, methadone maintenance treatment, or buprenorphine within 30 days of ED or inpatient discharge
7	**Population Size Adjustment**	Medicaid beneficiaries with an opioid-related ED visit during the relevant study period

1	**Definition Number**	2.13
2	**Definition Name**	Incidents of high-risk opioid prescribing
3	**Population**	All residents
4	**Data Sources**	Prescription drug monitoring program data
5	**Other Available Data for Additional or State-Specific Analyses**	Claims (Medicaid in Kentucky; all-payer in Massachusetts and New York; combined Medicaid/OhioMHAS in Ohio)
6	**Primary Outcome Measure**	Count of individuals with one or more of the following during the 12-month study period, and not in a prior specified time window (3 months):<br>Risk of continued opioid use (new opioid episode lasting at least 31 days)<br>Initiating opioid treatment with an extended-release or long-acting opioid<br>Incident high dosage (average ≥90 mg morphine per day)<br>Incident overlapping opioid and benzodiazepine for ≥30 days<br>(Measures A–D will be tracked separately and aggregated for the H4 analysis to a total count of unique individuals during the 12-month measurement period)
7	**Population Size Adjustment**	The population size estimate for each HCS community will be the mid-year population estimate as of July 1 for the year under surveillance

8	**Covariates**	The H4 analysis will control for the following community-level covariates: rural/urban classification, HCS site, and baseline rates

1	**Definition Number**	2.14.3
2	**Definition Name**	Number of naloxone units distributed in communities
3	**Population**	General residents in HCS communities
4	**Data Sources**	Data sources include state administrative sources, HCS study records and pharmacy sales<br>1. Data from the office in each state's department of health or contracting agency that distributes naloxone to groups for community distribution (exclusive of pharmacies) and data from HCS for any naloxone distributed by the study<br>2. Naloxone dispensed by pharmacies will be obtained from the IQVIA pharmacy database Xponent® for all sites
5	**Other Available Data for Additional or State-Specific Analyses**	Massachusetts can expand the measure by using all-payer claims data. Medicaid may be a source in other states.
6	**Primary Outcome Measure**	Count of naloxone units distributed in the HCS communities during the measurement period as captured by the following submeasures:<br>2.14.1.: Count of naloxone units distributed by the state health agency (secondary data from state health agencies) and HCS study logs for naloxone distributed by the study.<br>2.14.2.: Count of dispensed naloxone units from pharmacies (IQVIA Dispensed Prescription Data)<br>2.14.3.: This is the sum of 2.14.1 and 2.14.2
7	**Population Size Adjustment**	The population size estimate for each HCS community will be the mid-year population estimate as of July 1 for the year under surveillance
8	**Covariates**	The H2 analysis will control for the following community-level covariates: rural/urban classification, HCS site, and baseline rates

1	**Definition Number**	2.15
2	**Definition Name**	Number of individuals with OUD who are screened, diagnosed, and treated for hepatitis C
3	**Population**	Medicaid beneficiaries residing in HCS communities

4	**Data Sources**	Medicaid administrative data
5	**Other Available Data for Additional or State-Specific Analyses**	All-payer claims in Massachusetts
6	**Primary Outcome Measure**	Count of HCS residents who are Medicaid enrollees with OUD who are screened, diagnosed, and treated for hepatitis C
7	**Population Size Adjustment**	Medicaid beneficiaries during the time period

1	**Definition Number**	2.16
2	**Definition Name**	Number of newly diagnosed HIV cases
3	**Population**	All residents
4	**Data Sources**	State-specific registry for HIV/AIDS reporting; funding provided by CDC to state health departments for surveillance (*http://www.cdc.gov/hiv/library/reports/surveillance/*)
5	**Other Available Data for Additional or State-Specific Analyses**	Medicaid claims data
6	**Primary Outcome Measure**	Count of newly diagnosed cases of HIV
7	**Population Size Adjustment**	The population size estimate for each HCS community will be the mid-year population estimate as of July 1 for the year under surveillance

	**Definition Number**	2.17
2	**Definition Name**	Number of opioid-related overdoses treated in EDs and captured by syndromic surveillance data
3	**Population**	This is a population measure and would refer to the count in the local community; thus, all demographic groups would be included
4	**Data Sources**	Syndromic surveillance records (accessed via CDC's NSSP-ESSENCE application or other state-based platforms)
5	**Other Available Data for Additional or State-Specific Analyses**	N/A
6	**Primary Outcome Measure**	Number of opioid-related overdoses treated in EDs in the HCS community

7	**Population Size Adjustment**	Mid-year population estimates

1	**Definition Number**	2.18
2	**Definition Name**	Number of new acute opioid prescriptions limited to a 7-day supply
3	**Population**	All residents
4	**Data Sources**	Prescription drug monitoring program; census data for population estimates
5	**Other Available Data for Additional or State-Specific Analyses**	Claims (Medicaid in Kentucky and Ohio; all-payer in Massachusetts and New York—these are inferior to Prescription Monitoring Program (PMP) because they lack cash payments but would allow for removal of patients with cancer or on hospice care)
6	**Primary Outcome Measure**	Count of individuals with a new opioid prescription (45-day washout period preceding opioid prescription with no opioids) with a supply for ≤7 days
7	**Population Size Adjustment**	The population size estimate for each HCS community will be the mid-year population estimate as of July 1 for the year under surveillance

12.1.3 Structural Outcomes

1	**Definition Number**	3.1
2	**Definition Name**	Opioid prescriptions from multiple prescribers or pharmacies
3	**Data Sources**	Prescription drug monitoring program; CDC standards for MME; census data for population estimates
4	**Other Available Data for Additional or State-Specific Analyses**	Claims (Medicaid in Kentucky, all-payer in Massachusetts and New York, Ohio can combine Medicaid and OhioMHAS data)—these are inferior to PMP because they lack cash payments)
5	**Primary Outcome Measure**	Count of individuals with an opioid prescription from ≥4 providers or ≥4 pharmacies in a quarter
6	**Population Size Adjustment**	Mid-year population estimate

1	**Definition Number**	3.2

2	**Definition Name**	Number of providers with a waiver under the Drug Addiction Treatment Act of 2000 (DATA 2000)
3	**Data Sources**	Drug Enforcement Administration's Active Controlled Substances Act (CSA) Registrants Database, which lists all provider types that meet the requirements to prescribe buprenorphine for the treatment of OUD; requires a purchased subscription from the National Technical Information Service (NTIS) (*https://classic.ntis.gov/products/dea/*)
4	**Other Available Data for Additional or State-Specific Analyses**	N/A
5	**Primary Outcome Measure**	Using information from the DEA's Active CSA Registrants Database, calculate the total number of buprenorphine for OUD treatment providers: (1) civilian physicians, (2) civilian nurse practitioners, and (3) civilian physician assistants
6	**Population Size Adjustment**	Mid-year population estimates
7	**Strata**	Measures will be stratified by level of prescribing

1	**Definition Number**	3.3
2	**Definition Name**	Number of providers with a DATA 2000 waiver who actively prescribe buprenorphine products that are FDA approved for OUD
3	**Data Sources**	The calculation of this measure will require linkage between the DEA Active CSA Registrants Database and the state prescription drug monitoring program records to identify waivered prescribers from the HCS communities and their volume of prescriptions for buprenorphine products approved by FDA for the treatment of OUD.<br>Drug Enforcement Administration's Active Controlled Substances Act (CSA) Registrants Database, which lists all physicians who meet the requirements to prescribe buprenorphine for the treatment of OUD; requires a purchased subscription from NTIS (*https://classic.ntis.gov/products/dea/*) state prescription drug monitoring program records
4	**Other Available Data for Additional or State-Specific Analyses**	N/A

5	**Primary Outcome Measure**	Using information from the DEA's Active CSA Registrants Database, identify waivered prescribers (see measure 3.2) from the HCS communities and their allowed capacity (number of patients that they can treat at a given point)<br>Using the state prescription drug monitoring program records, for each prescriber in step 1, identify the unique patients who had at least one dispensed prescription issued by this prescriber for an FDA-approved buprenorphine product for treatment of OUD during the period under surveillance.<br>Calculate the total number of prescribers who prescribed buprenorphine to at least one patient
6	**Population Size Adjustment**	The denominator would be the "number of providers with a DATA 2000 waiver" (measure 3.2)
7	**Strata**	Measures will be stratified by level of prescribing

1	**Definition Number**	3.4
2	**Definition Name**	Number of providers who actively prescribe buprenorphine products that are FDA approved for OUD
3	**Data Sources**	Prescription Drug Monitoring Program (PDMP) data
4	**Other Available Data for Additional or State-Specific Analyses**	None
5	**Primary Outcome Measure**	Number of dispensed prescriptions with National Drug Code (NDC) for buprenorphine products that are approved by the FDA for the treatment of OUD.

1	**Definition Number**	3.5
2	**Definition Name**	Number of jails initiating and linking people to MOUD
3	**Data Sources**	De novo
4	**Other Available Data for Additional or State-Specific Analyses**	None
5	**Primary Outcome Measure**	1. Number of jails associated with HCS communities that initiate buprenorphine/methadone/Vivitrol for inmates before release<br>2. Number of jails associated with HCS communities that link people to MOUD treatment after release<br>3. Number of jails that provide naloxone to people upon release

1	**Definition Number**	3.8
2	**Definition Name**	Number of take-back drug drop boxes
3	**Data Sources**	All states will use DEA records to get the number of current drop boxes.
4	**Other Available Data for Additional State-Specific Analyses**	None
5	**Primary Outcome Measure**	Count of drug take-back boxes in HCS Communities

1	**Definition Number**	4.1.1
2	**Definition Name**	Number of ED visits for BH (count visits)
3	**Population**	Medicaid beneficiaries
4	**Data Sources**	Medicaid administrative data
5	**Other Available Data for Additional or State-Specific Analyses**	Claims (Medicaid in Kentucky, all-payer in Massachusetts and New York, combined Medicaid and OhioMHAS in Ohio; OASAS in New York; BSAS in Massachusetts)
6	**Primary Outcome Measure**	Count the number of unique emergency department (ED) visits with a behavioral health (BH) diagnosis code in the measurement period
7	**Population Size Adjustment**	Count of HCS residents who are Medicaid enrollees aged 18–64 who are non-dual eligible during the measurement period

1	**Definition Number**	4.1.2
2	**Definition Name**	Number of ED visits for non-BH (count visits)
3	**Population**	Medicaid beneficiaries
4	**Data Sources**	Medicaid administrative data
5	**Other Available Data for Additional or State-Specific Analyses**	Claims (Medicaid in Kentucky, all-payer in Massachusetts and New York, combined Medicaid and OhioMHAS in Ohio; OASAS in New York; BSAS in Massachusetts)
6	**Primary Outcome Measure**	Count the number of unique emergency department visits that do not have a behavioral health diagnosis code in the measurement period

7	**Population Size Adjustment**	Count of HCS residents who are Medicaid enrollees aged 18–64 who are non-dual eligible during the measurement period

1	**Definition Number**	4.2.1
2	**Definition Name**	Number of hospital/inpatient nights for non-detox BH (count nights)
3	**Population**	Medicaid beneficiaries
4	**Data Sources**	Medicaid administrative data
5	**Other Available Data for Additional or State-Specific Analyses**	Claims (Medicaid in Kentucky, all-payer in Massachusetts and New York, combined Medicaid and OhioMHAS in Ohio; OASAS in New York; BSAS in Massachusetts)
6	**Primary Outcome Measure**	Count the number of inpatient nights with a behavioral health diagnosis code, excluding inpatient stays with a detox revenue or procedure code, in the measurement period
7	**Population Size Adjustment**	Count of HCS residents who are Medicaid enrollees aged 18–64 who are non-dual eligible during the measurement period

1	**Definition Number**	4.2.2
2	**Definition Name**	Number of hospital/inpatient nights for detox (count nights)
3	**Population**	Medicaid beneficiaries
4	**Data Sources**	Medicaid administrative data
5	**Other Available Data for Additional or State-Specific Analyses**	Claims (Medicaid in Kentucky, all-payer in Massachusetts and New York, combined Medicaid and OhioMHAS in Ohio; OASAS in New York; BSAS in Massachusetts)
6	**Primary Outcome Measure**	Count the number of detox inpatient nights in the measurement period
7	**Population Size Adjustment**	Count of HCS residents who are Medicaid enrollees aged 18–64 who are non-dual eligible during the measurement period

1	**Definition Number**	4.2.3
2	**Definition Name**	Number of hospital/inpatient nights for non-BH (count nights)
3	**Population**	Medicaid beneficiaries
4	**Data Sources**	Medicaid administrative data

5	**Other Available Data for Additional or State-Specific Analyses**	Claims (Medicaid in Kentucky, all-payer in Massachusetts and New York, combined Medicaid and OhioMHAS in Ohio; OASAS in New York; BSAS in Massachusetts)
6	**Primary Outcome Measure**	Count the number of inpatient nights that are NOT behavioral health in nature in the measurement period
7	**Population Size Adjustment**	Count of HCS residents who are Medicaid enrollees aged 18–64 who are non-dual eligible during the measurement period

1	**Definition Number**	4.3.1
2	**Definition Name**	Number of non-detox BH residential nights (count nights)
3	**Population**	Medicaid beneficiaries
4	**Data Sources**	Medicaid administrative data
5	**Other Available Data for Additional or State-Specific Analyses**	Claims (Medicaid in Kentucky, all-payer in Massachusetts and New York, combined Medicaid and OhioMHAS in Ohio; OASAS in New York; BSAS in Massachusetts)
6	**Primary Outcome Measure**	Count the number of residential non-detox nights, excluding the number of detox nights, in the measurement period; residential services by nature are assumed to be behavioral health related
7	**Population Size Adjustment**	Count of HCS residents who are Medicaid enrollees aged 18–64 who are non-dual eligible during the measurement period

1	**Definition Number**	4.3.2
2	**Definition Name**	Number of BH detox residential nights (count nights)
3	**Population**	Medicaid beneficiaries
4	**Data Sources**	Medicaid administrative data
5	**Other Available Data for Additional or State-Specific Analyses**	Claims (Medicaid in Kentucky, all-payer in Massachusetts and New York, combined Medicaid and OhioMHAS in Ohio; OASAS in New York; BSAS in Massachusetts)
6	**Primary Outcome Measure**	Count the number of residential detox nights in the measurement period; residential services by nature are assumed to be behavioral health related
7	**Population Size Adjustment**	Count of HCS residents who are Medicaid enrollees aged 18–64 who are non-dual eligible during the measurement period

1	**Definition Number**	4.4.1
2	**Definition Name**	Number of intensive BH outpatient visits (count nights)
3	**Population**	Medicaid beneficiaries
4	**Data Sources**	Medicaid administrative data
5	**Other Available Data for Additional or State-Specific Analyses**	Claims (Medicaid in Kentucky, all-payer in Massachusetts and New York, combined Medicaid and OhioMHAS in Ohio; OASAS in New York; BSAS in Massachusetts)
6	**Primary Outcome Measure**	Count the number of intensive outpatient visits in the measurement period; intensive outpatient visits by nature are assumed to be behavioral health related
7	**Population Size Adjustment**	Count of HCS residents who are Medicaid enrollees aged 18–64 who are non-dual eligible during the measurement period

1	**Definition Number**	4.5.1
2	**Definition Name**	Number of outpatient visits BH (count visits)
3	**Population**	Medicaid beneficiaries
4	**Data Sources**	Medicaid administrative data
5	**Other Available Data for Additional or State-Specific Analyses**	Claims (Medicaid in Kentucky, all-payer in Massachusetts and New York, combined Medicaid and OhioMHAS in Ohio; OASAS in New York; BSAS in Massachusetts)
6	**Primary Outcome Measure**	Count the number of outpatient visits with a behavioral health diagnosis code in the measurement period
7	**Population Size Adjustment**	Count of HCS residents who are Medicaid enrollees aged 18–64 who are non-dual eligible during the measurement period

1	**Definition Number**	4.5.2
2	**Definition Name**	Number of outpatient visits non-BH (count visits)
3	**Population**	Medicaid beneficiaries
4	**Data Sources**	Medicaid administrative data
5	**Other Available Data for Additional or State-Specific Analyses**	Claims (Medicaid in Kentucky, all-payer in Massachusetts and New York, combined Medicaid and OhioMHAS in Ohio; OASAS in New York; BSAS in Massachusetts)

6	**Primary Outcome Measure**	Count the number of outpatient visits that do NOT have a behavioral health diagnosis code in the measurement period
7	**Population Size Adjustment**	Count of HCS residents who are Medicaid enrollees aged 18–64 who are non-dual eligible during the measurement period

1	**Definition Number**	4.6.1
2	**Definition Name**	Number of non-pain buprenorphine days supplied (count days supply)
3	**Population**	Medicaid beneficiaries
4	**Data Sources**	Medicaid administrative data
5	**Other Available Data for Additional or State-Specific Analyses**	Claims (Medicaid in Kentucky, all-payer in Massachusetts and New York, combined Medicaid and OhioMHAS in Ohio; OASAS in New York; BSAS in Massachusetts)
6	**Primary Outcome Measure**	Count the number of days supplied of non-pain oral buprenorphine in the measurement period; this will come from two datasets: prescription drug claims and outpatient claims
7	**Population Size Adjustment**	Count of HCS residents who are Medicaid enrollees aged 18–64 who are non-dual eligible during the measurement period

1	**Definition Number**	4.6.2
2	**Definition Name**	Number of non-pain buprenorphine injections (count injections)
3	**Population**	Medicaid beneficiaries
4	**Data Sources**	Medicaid administrative data
5	**Other Available Data for Additional or State-Specific Analyses**	Claims (Medicaid in Kentucky, all-payer in Massachusetts and New York, combined Medicaid and OhioMHAS in Ohio; OASAS in New York; BSAS in Massachusetts)
6	**Primary Outcome Measure**	Count the number of non-pain buprenorphine injections in the measurement period; this will come from two datasets: outpatient claims and prescription drug claims.
7	**Population Size Adjustment**	Count of HCS residents who are Medicaid enrollees aged 18–64 who are non-dual eligible during the measurement period

1	**Definition Number**	4.6.3

2	**Definition Name**	Number of opioid-related oral naltrexone days supplied (count days supply)
3	**Population**	Medicaid beneficiaries
4	**Data Sources**	Medicaid administrative data
5	**Other Available Data for Additional or State-Specific Analyses**	Claims (Medicaid in Kentucky, all-payer in Massachusetts and New York, combined Medicaid and OhioMHAS in Ohio; OASAS in New York; BSAS in Massachusetts)
6	**Primary Outcome Measure**	Count the number of prescription days supplied that are opioid related oral naltrexone fills in the measurement period; the population in the denominator for this measure requires evidence (i.e., a diagnosis) of an OUD in the measurement period or in the past 12 months
7	**Population Size Adjustment**	Count of HCS residents who are Medicaid enrollees aged 18–64 who are non-dual eligible during the measurement period

1	**Definition Number**	4.6.4
2	**Definition Name**	Number of opioid-related naltrexone injections (count injections)
3	**Population**	Medicaid beneficiaries
4	**Data Sources**	Medicaid administrative data
5	**Other Available Data for Additional or State-Specific Analyses**	Claims (Medicaid in Kentucky, all-payer in Massachusetts and New York, combined Medicaid and OhioMHAS in Ohio; OASAS in New York; BSAS in Massachusetts)
6	**Primary Outcome Measure**	Count the number of opioid related naltrexone injections in the measurement period; this will come from two datasets: outpatient claims and prescription drug claims. The population in the denominator for this measure requires evidence (i.e., a diagnosis) of an OUD in the measurement period or in the past 12 months.
7	**Population Size Adjustment**	Count of HCS residents who are Medicaid enrollees aged 18–64 who are non-dual eligible during the measurement period

1	**Definition Number**	4.6.5
2	**Definition Name**	Number of methadone days supplied (count calculated days supply)
3	**Population**	Medicaid beneficiaries

4	**Data Sources**	Medicaid administrative data
5	**Other Available Data for Additional or State-Specific Analyses**	Claims (Medicaid in Kentucky, all-payer in Massachusetts and New York, combined Medicaid and OhioMHAS in Ohio; OASAS in New York; BSAS in Massachusetts
6	**Primary Outcome Measure**	Count the number of days that methadone has been supplied in the measurement period
7	**Population Size Adjustment**	Count of HCS residents who are Medicaid enrollees aged 18–64 who are non-dual eligible during the measurement period

1	**Definition Number**	4.7.1
2	**Definition Name**	Number of opioid pain medication days supplied (count days supply)
3	**Population**	Medicaid beneficiaries
4	**Data Sources**	Medicaid administrative data
5	**Other Available Data for Additional or State-Specific Analyses**	Claims (Medicaid in Kentucky, all-payer in Massachusetts and New York, combined Medicaid and OhioMHAS in Ohio; OASAS in New York; BSAS in Massachusetts
6	**Primary Outcome Measure**	Count the number of prescription days supplied that are opioid pain medication fills in the measurement period
7	**Population Size Adjustment**	Count of HCS residents who are Medicaid enrollees aged 18–64 who are non-dual eligible during the measurement period

1	**Definition Number**	4.7.2
2	**Definition Name**	Number of non-opioid pain medication days supplied (count days supply)
3	**Population**	Medicaid beneficiaries
4	**Data Sources**	Medicaid administrative data
5	**Other Available Data for Additional or State-Specific Analyses**	Claims (Medicaid in Kentucky, all-payer in Massachusetts and New York, combined Medicaid and OhioMHAS in Ohio; OASAS in New York; BSAS in Massachusetts)
6	**Primary Outcome Measure**	Count the number of prescription days supplied that are non-opioid pain medication fills in the measurement period
7	**Population Size Adjustment**	Count of HCS residents who are Medicaid enrollees aged 18–64 who are non-dual eligible during the measurement period

12.2 Appendix B: Health Message Testing Service Questions—Centers for Disease Control and Prevention

Core Questions

(Questions can be used for Central Location Intercept Interviews, Telephone Interviews, Individual In-depth Interviews [Cognitive Interviews], Focus Group Screeners, and Focus Groups.

Comprehension

1d. What is the main idea that this message is trying to get across, in your own words?

2d. How well do you think the main ideas come across?

3d. Is it trying to get people to do something?

- What action would the message prompt you to take?

4d. Were there any words that were unusual or unfamiliar?

5d. What other words can be used in their place? What would you say is the main idea or ideas they are trying to convey here?

6d. Is there anything confusing, unclear, or hard to understand?

Impressions

7d. How would you sum up in just a few words your first impression of this message? Do you like it? Not like it? What makes you say that?

8d. What feelings do you have in reaction to this message? Anything positive? Anything negative?

9d. Was your reaction to this positive or negative?

- What positive images do you associate with “[INSERT message/phrase]?”

- What negative images do you associate with "[INSERT message/phrase]"?

10d. Is this an appealing message?

- What makes the message appealing or unappealing?

11d. How does it make you feel?

12d. Was this a new idea or something that you've heard before?

13d. Do you strongly agree with any part of this message? If so, what?

14d. Do you strongly disagree with anything in this message? If so, what?

15d. Is this message believable or not? Why or why not?

16d. Is this message believable? GRID FORMAT, ROTATE LIST AS RELEVANT. (MESSAGES TO BE INSERTED BASED ON SPOTS.

- Yes
- No

A. INSERT message 1 from Ad

B. INSERT message 2 from Ad

C. INSERT message 3 from Ad

D. INSERT message 4 from Ad

17d. What additional information would you need in order to more strongly believe this message?

18d. Are any of the five advertisements memorable?

- Yes

- No

19d. Please rank these five advertisements by placing a 1 by the ad you felt was the most memorable, a 2 by your second-most memorable, etc. RANKING QUESTION UP TO TOP 5, FORCE EXACTLY ONE RESPONSE FOR EACH OF 1 THROUGH 5. ROTATE 1-5.

1. PICTURE/DESCRIPTION OF FIRST SPOT VIEWED
2. PICTURE/DESCRIPTION OF SECOND SPOT VIEWED
3. PICTURE/DESCRIPTION OF THIRD SPOT VIEWED
4. PICTURE/DESCRIPTION OF FOURTH SPOT VIEWED
5. PICTURE/DESCRIPTION OF FIFTH SPOT VIEWED

20d. Do you think you will talk about any of these ads with others?

- Yes
- No

21d. Please rank these five advertisements by placing a 1 by the ad you felt you are more likely to talk about with others, a 2 by your second-most likely to talk about, etc. RANKING QUESTION UP TO TOP 5, FORCE EXACTLY ONE RESPONSE FOR EACH OF 1 THROUGH 5. ROTATE 1-5.

1. PICTURE/DESCRIPTION OF FIRST SPOT VIEWED
2. PICTURE/DESCRIPTION OF SECOND SPOT VIEWED
3. PICTURE/DESCRIPTION OF THIRD SPOT VIEWED
4. PICTURE/DESCRIPTION OF FOURTH SPOT VIEWED
5. PICTURE/DESCRIPTION OF FIFTH SPOT VIEWED

22d. Did you think any of these ads was effective to motivate you or someone else to [INSERT health behavior/message/phrase]?

- Yes
- No

23d. Please rank these five advertisements by placing a 1 by the ad you felt was the most effective in motivating you or someone else to [INSERT health behavior/message/phrase], a 2 by the second-most effective in motivating you to [INSERT health behavior/message/phrase], etc. RANKING QUESTION UP TO TOP 5, FORCE EXACTLY ONE RESPONSE FOR EACH OF 1 THROUGH 5. ROTATE 1-5.

1. PICTURE/DESCRIPTION OF FIRST SPOT VIEWED
2. PICTURE/DESCRIPTION OF SECOND SPOT VIEWED
3. PICTURE/DESCRIPTION OF THIRD SPOT VIEWED
4. PICTURE/DESCRIPTION OF FOURTH SPOT VIEWED
5. PICTURE/DESCRIPTION OF FIFTH SPOT VIEWED

Audience and Personal Relevance

24d. Who would you say they are trying to reach?

- Does it seem like this message is talking to you, and people like you? Or someone else?
- What in the message suggested it was talking to you and people like you or someone else?

25d. Who would you say they are trying to reach? *Please check all that apply.*

1. You
2. People like you
3. Someone else
4. None of these

26d. Do you see yourself doing this? Or something like it? Why/why not?

27d. Who do you believe would benefit most from [INSERT health behavior]?

Content and Wording

28d. Do you like the way it is written? [Probe: tone, language/style, etc.] Is it easy to read?

29d. Is there anything you want to know that this item does not tell you?

30d. If you saw or heard this message, would it get your attention? Why or why not?

31d. Are there any words or phrases here that you think are especially attention-getting or appealing?

32d. Are there any words or phrases that bother you or that you think should be said differently?

33d. How could this message be improved?

34d. Is there a way to say this differently that would make you personally more likely to notice and think about the message?

35d. Thinking back to the information they are trying to convey, is there anything else you would add?

36d. [Underline/circle/highlight/cross out] phrases, sentences or images that you think are [important/confusing/unclear/inappropriate/offensive/should be deleted].

What did you indicate as [important/confusing/unclear/inappropriate/offensive/to be deleted]?

37d. How much of the [INSERT item] would you read? Please choose all that apply.

[Screen shows the responses below]

- I would only read the headline
- I would only look at the image/s
- I would only read the headline and look at the image/s
- I would read the whole thing

- I wouldn't read any of it

Efficacy

38d. Which actions, if any, sound doable to you? Why?

39d. How feasible is it that you would try to do this? Please explain.

40d. Would you consider doing this behavior?

41d. What makes it hard to do this?

42d. Who in your household would be against trying this? Why?

43d. Which descriptions, if any, sound do-able? Why? Why not?

44d. Which of these options would be the easiest to incorporate into your life? What makes that option the easiest?

45d. What do you think of this idea?

46d. How appealing is it to you as a way to control [INSERT health topic or behavior]?

47d. Do you think it could work in your home? Why or why not?

48d. Would you consider doing this at home?

49d. How would you try it out in your home?

50d. How feasible would this be to try at home? Explain.

51d. What would make this easier for you to do?

52d. How would you try this at work or when out of your home?

53d. Which of these would be most effective in your workplace?

54d. How easy are these guidelines for you to follow and understand?

55d. What, if anything, makes it difficult to follow? How might this be presented in an easier way?

56d. What are the good things about trying this tactic?

57d. What makes it hard to do this?

58d. Who in your household would be against trying this? Why?

59d. I’m going to pass around a sheet that gives you some descriptions for [INSERT health topic or behavior]. Please review these descriptions silently, and circle those that seem most doable. Cross out any that don’t seem do-able.

- After all tactics have been discussed, the moderator asks participants to identify the two or three tactics that they think are most important and that they have the greatest likelihood of doing. (Moderator takes hand count.)

- From the two or three top tactics, the participants are asked to choose the single tactic that they think is most important and that they have the greatest likelihood of implementing. The moderator explores some of the participants’ choices and their reasons for choosing.

- As you look at these strategies, does any one of the three stand out as the one that you would try first? Which one? Why?

- What are the one or two things we have discussed today — if any — that

seem most feasible to implement in your household?

- Is there any more information or tools that you would need that would help you implement some of these strategies?
- What are some of the things that would make it difficult for people in your area to [INSERT health topic or behavior]?
- What could, or has, been done in the local area to make it easier—to overcome some of the barriers you described?

Placement

60d. Do you like the idea of having material to take with you into a doctor's appointment or to take home?

61d. Do you think you would take information with you, or leave it in the waiting room? Why?

62d. Does this affect your likelihood to read the information? How?

63d. Does it affect your likelihood to ask your doctor? How?

64d. Where would you expect to see it?

65d. Where would it need to be so that you would pay attention to it?

66d. How do you prefer to see health information presented?

67d. In what form (probe: posters, brochures, fliers)?

68d. What kinds of promotional items would you use?

Channels

69d. Where do you get your information about [INSERT health topic or behavior]?

- Probe for sources such as media, family, friends, church

70d. Has your doctor talked with you about [INSERT health topic or behavior]?

- What did he/she tell you?

71d. What are some of the ways you have gotten information about [INSERT health topic or behavior] prior to today? [Probe: role of media, word-of-mouth, other.]

72d. Is [INSERT health topic or behavior] a topic on which you seek out information? If so, how?

- What type of information are you seeking?
- Where might you seek out information?
- Where would you turn first for information?

73d. How often, if at all, do you pick up information about [INSERT health topic or behavior]?

- What are the sources of this information?

74d. When it comes to [INSERT health topic or behavior], are there any organizations that you would really trust as a reliable source of information?

75d. What makes them a trusted source of [INSERT health topic or behavior] information?

76d. What do you think [INSERT organization name] needs to know about your community? How would you want them to be involved in your community? Do you see ways of partnering with them? How would you like to receive the information?

77d. How do you usually learn about environmental issues? Who or what is your main source of trusted information about community issues? Is there adequate information out there? How do these sources compare to [INSERT organization name]?

78d. How would you rank your level of knowledge and understanding of information you have received from [INSERT organization name]?

- 5: knowledge is extensive
- 4: above average
- 3: average
- 2: have some knowledge
- 1: no knowledge

79d. How could this information be conveyed more effectively?

80d. What types of information would you like to receive regarding environmental issues? What would be the most effective way or format to provide this information [INSERT health topic or behavior]? (Probes: Video? Educational pamphlets? Community/public meetings? Internet?)

81d. What are some places where you might notice messages like these?

82d. Are there some places in particular that you would be most likely to notice and pay attention to these messages?

Sources of Information

83d. If you are trying to put together a group of people in your community to deal with [INSERT health topic or behavior] in a comprehensive fashion, who are the people you want at the table?

- Probe: Community based organizations, health care practitioners, and policy makers
- Probe: Religious leaders/faith-based organizations
- Probe: What is it about these people that make you want them there?
- Probe: Once you get these people in the room, what is the conversation going to sound like?

84d. What topics do you want to cover? What will be the outcome of this conversation, what kinds of things are going to happen as a result?

85d. Who wrote or produced this item?

86d. Have you heard of them before?

87d. Are they a good source of information?

88d. Are they believable?

89d. Do they seem trustworthy?

90d. How do you feel about CDC as the source of this information?

Spokespeople/Sponsor

91d. Who do you think would be a good spokesperson to use to convince you and your friends to [INSERT health topic or behavior]?

92d. Who would have the ability to influence you?

- Would it be a physician, celebrity, religious or spiritual leader, or someone like you?

93d. If you were trying to make up your mind about [INSERT health topic or behavior], who would influence you?

94d. If you were trying to influence a friend to [INSERT health topic or behavior], what would you say?

- Probe for benefits and ways to overcome barriers

95d. Have you ever heard of [INSERT organization name]?

96d. What is [INSERT organization name]? Where did you learn about [INSERT organization name]?

97d. What could [INSERT organization name] do to make you feel better about the health risks in your community?

98d. Do any companies or organizations say something like this now? Which ones?

99d. What impact does [INSERT organization name] have on your community? On you and your friends and family's lives? What kind of impact should it have?

100d. What do you think about the work of [INSERT organization name]? Why?

101d. Have you ever heard of an organization called the Centers for Disease Control and Prevention or CDC?

102d. What if the CDC was to say something like this? Would that change the way you look at these statements? Would it make any of them more or less believable? More or less appealing? Motivating?

103d. What if the CDC and [INSERT partner name] said something like this? Would that change the way you look at these statements? Would it make any of them more or less believable? More or less appealing? Motivating?

Comparison of Concepts/Messages/Materials

104d. Do you think one is more appealing than the others? Which? Why/why not?

105d. Is one more likely than another to get your attention?

- To make you think about it afterwards?
- If yes, what is it about that one that "works" for you (or someone else you care for or take care of, such as a spouse/partner, child, parent, relative, or friend)?

106d. Now that you've seen all of these concepts, which one catches your attention the most?

107d. Now that you've seen all of these ads, rank which one catches your attention the most by placing a 1 by the ad you liked the most, a 2 by your (NEXT) favorite, etc. SINGLE RESPONSE. SCREEN CAPTURES FROM THE ADS WILL BE INSERTED FOR VISUAL RECALL OF THE ADS.

1. PICTURE/DESCRIPTION OF FIRST SPOT VIEWED
2. PICTURE/DESCRIPTION OF SECOND SPOT VIEWED
3. PICTURE/DESCRIPTION OF THIRD SPOT VIEWED
4. PICTURE/DESCRIPTION OF FOURTH SPOT VIEWED
5. PICTURE/DESCRIPTION OF FIFTH SPOT VIEWED

108d. Looking over all of the different messages we have discussed, which two or three are the most effective?

- Probe: What makes it most effective?

109d. Looking over all of the different messages, which two or three are the most effective? INSERT LIST OF MESSAGES VIEWED IN THE 5 SPOTS. MULTIPLE MENTION. *Please check up to three.*

110d. Which one was most inspiring or motivating for you personally?

111d. Which one is most believable?

112d. Now, looking at just the top two or three messages, which one message is the most important to you to help you [INSERT health topic or behavior] and that you have the greatest likelihood of doing?

113d. What makes this message most effective?

114d. What about this particular one is most engaging?

115d. What item is least effective?

116d. Which of these is the worst? Why? What, specifically, do you dislike about it? What do you mean by “worst?” What criteria did you use?

- Least likely to attract attention?

- Least likely to read?
- Least likely to act upon?

117d. Did any of the concepts not motivate you at all? Why not?

118d. Did any of the concepts turn you off? What was it about the statement/s that turned you off?

119d. Would any of the statements make you feel opposed [INSERT health topic or behavior]?

120d. What could be changed to make it more effective?

121d. What information would most motivate you to ask your doctor about [INSERT health topic or behavior]?

122d. What is the most motivational format for this information?

Campaign Awareness

123d. In the past month, do you remember seeing, hearing, or reading any TV, radio, newspaper, or online advertising about a [INSERT program, campaign, or web site description]?

- Yes
- No
- Don't Know/Not Sure
- Refused

124d. What can you tell me about this/these [INSERT format]? Please describe for me anything specific you saw, heard, or read in the [INSERT format].

- What was the [INSERT format] about?
- What was the name of the program mentioned in the [INSERT format]? Is there anything else you can recall?

[**Probe** for specifics/details they remember, for example who, if anyone, appeared in the message.]

125d. In general, are you aware of [INSERT description of program, campaign, or web site]?

- Yes
- No
- Don’t Know/Not Sure
- Refused

126d. What is the name of the [INSERT description of program, campaign, or web site] (Probe: Any others?)

127d. Now, I am going to describe an ad you may or may not have seen on TV. You may or may not have seen the following ad because it is NOT running in all parts of the country. But in the past month, have you happened to have seen an ad that shows [INSERT ad description].

- Yes
- No
- Don’t Know/Not Sure
- Refused

128d. n a scale from 1 to 10 where 1 is *not at all appealing* and 10 is *extremely appealing*, how would you rate this [INSERT type of communication, e.g., ad]?

	Not at All Appealing 1	2	3	4	5	6	7	8	9	Extremely Appealing 10	DK 88	RF 99
How would you rate this [INSERT type of communication, e.g., ad]?												

129d. Now I’m going to read a list of a few [INSERT type of communication, e.g., website, slogan, or program] names you may or may not have heard about. Which of the following [INSERT type of communication, e.g., website, slogan, or program] have you heard about? [REPEAT as necessary for other types of communication]

- Yes
- No
- Don’t Know/Not Sure
- Refused

12.3 Appendix C: Justice Community Opioid Innovation Network (JCOIN) and HCS Annual Jail Survey

As part of a separate federally funded (NIDA) grant, unrelated to the HCS, Dr. Chris Scott of the Lighthouse Institute of Chestnut Health Systems is conducting interviews with jail administrators. The survey (the Justice Community Opioid Innovation Network Jail Interview [JCOIN]) is designed to collect administrative data about the availability, accessibility, and utilization of interventions to treat opioid use disorder (OUD) in jails. Dr. Scott works with each sampled jail to determine who at the jail is best able to provide the institutional data she requires, and all the questions are about the facility and its procedures (i.e., no individual-level data are collected).

The data Dr. Scott is collecting are relevant for the HCS, and she will share data with the HCS team once the appropriate data sharing agreements are in place. Once she completes the JCOIN data collection, she will alert the HCS team in that state, and the HCS team can then reach out to specific jails in the JCOIN sample to request their involvement with the HCS. The HCS team will not know whether the jail participated, only that the jail was included in the sample for Dr. Scott's study. If a jail is willing to have its JCOIN data shared with the HCS team, it will be asked to complete an Authorization to Disclose form and will provide completed forms to Dr. Scott. The informed consent for the jail interview contains a statement regarding other NIDA projects. The statement says that if the jail or county is participating in other NIDA projects and would like for JCOIN to share the data obtained in the JCOIN to please provide a release of information that explicitly states with whom they would like to share their data.

Dr. Scott has expanded the JCOIN sample to include an additional 33 counties representing HCS communities. Principal Investigators from the HCS will be responsible for obtaining Authorization to Disclose forms from the facilities to share the interview data with the HCS. The form makes it clear that the identity of the facility will be linked to the disclosed data for the purpose of re-contacting the jail in the future to request permission to update a portion of the data collected. The revised JCOIN protocol, JCOIN survey, JCOIN ICF, and Authorization to Disclose have all been approved by the local IRB for JCOIN.

For those jails that authorize the release of their (baseline) data to the HCS, the HCS will utilize the jail names to re-contact the jails for two additional follow-up surveys over the course of the HCS study. A standardized email message will be sent by the PIs to the jails to serve as an introduction to the project, and informed consent will be obtained for each HCS follow-up survey completed.

Justice Community Opioid Innovation Network (JCOIN)

in collaboration with the HEALing Communities Study (HCS)

AUTHORIZATION TO DISCLOSE

IDENTIFYING INFORMATION

Name of Jail: ______________________________

Name of Authorized Representative: ______________________________

(*preferably, the same Authorized Representative who signed the previous consent to participant in JCOIN. If not available, the current authorized designee may sign.*)

Jail Address: ______________________________

By signing this **Authorization to Disclose**, the above-named individual, on behalf of the above-named jail ("Jail"), acting with sufficient authority to legally bind Jail, hereby authorizes Chestnut Health Systems, Inc. ("Chestnut") to release to the individuals or entities named below the Jail information described below.

WHAT MAY BE DISCLOSED:

Chestnut may disclose data duly collected from Jail's responses to the Justice Community Opioid Innovation Network (JCOIN) Jail Interview ("Jail Interview") under a grant from the National Institute on Drug Abuse ("NIDA"). The identity of Jail will be linked to the disclosed data for the purpose of re-contacting Jail in the future to update a portion of the data collected. This completes your participation in the JCOIN survey. However, as part of their on-going monitoring of the HEALing Communities Study (HCS), HCS staff may follow-up with additional data-collection requests over the next two years.

WHO MAY RECEIVE:

Chestnut may disclose the above-described data to the following entities that have received funding from NIDA for the HCS to learn more about the availability, accessibility, and utilization of interventions to treat opioid use disorder (OUD):

[Name HSC research site to received data] - will receive data linked to Jail's identity only for the purpose described above. This Research Site will in-turn provide a copy of the data, with Jail identifying information *removed*, to RTI International, the HCS Data Coordinating Center, for the purpose of statistical analysis and summary reports.

Chestnut is in no way responsible for the use or sharing of Jail Interview data by the above entities.

GENERAL:

Jail understands and agrees that Chestnut will in no way be liable to it or any third party for disclosing Jail Interview data in accordance with this Authorization to Disclose and that Chestnut is in no way responsible for the use or sharing of Jail Interview data by the entities listed above. Further, Jail understands that it may revoke this Authorization to Disclose at any time by providing written notification to Chestnut. However, any revocation will not cover disclosures that Chestnut has already made pursuant to the authority granted by Jail in this Authorization to Disclose prior to the date of such revocation.

______________________________ ______________________________

Signature of Authorized Representative Date

______________________________ ______________________________

Name and Title of Authorized Representative Email Address of Authorized Representative

Procedures for Recruitment and Improving the Response Rates for the HCS Annual Jail Survey

To improve recruitment and survey response rates, the following procedures will be used. These procedures include jails that do not complete the JCOIN Baseline Survey but who consent to the HCS Annual Jail Survey at baseline. Jail Administrative Directors (i.e., jailers) will receive a pre-recruitment letter or email from a Jail Survey Champion(s) no less than 10 business days before the survey invitation is sent. The Jail Survey Champion may be the key government official for an RS, a local or state government official, and/or a local or state influencer (e.g., from the state's jailer's association or sheriff's association). The Jail Survey Champion letter or email may be co-signed by multiple stakeholders. The purpose of the Jail Survey Champion letter or email is to grant legitimacy to the HCS Jail Annual Survey and provide a brief introduction to the study's purpose. In addition, the goal is to request that the Jail Administrative Director identify a key jail employee who is knowledgeable about opioid-related services in his or her jail and describe this person's importance to the study (see *Attachment: Jail Survey Champion*). It is requested that the Jail Administrative Director send the name, title, telephone number, and email of the key jail employee to the RS's account (i.e., University of Kentucky, Ohio State University, Columbia University, or Boston Medical Center email account, e.g., HCS_KY@uky.edu). The RS may also reach out directly to the jail via telephone, email, or in person to request a key jail employee or alternate and not go through the Jail Survey Champion.

All key jail employees will be sent an email invitation with a link to the Jail Annual Survey, which will be sent from the RS's account (i.e., University of Kentucky, Ohio State University, Columbia University, or Boston Medical Center email account, e.g., HCS_KY@uky.edu) on a specified date. The email will also provide a brief introduction to the study's purpose and describe the target respondent's importance to the study (see *Attachments: Jail Survey Invitation and the Alternate Version for Ohio*, *Sheriff Email and Phone Script*). This email will contain the HCS graphic icon to establish rapport and verify authenticity. If target participants have questions, they will be able to respond to this email address, and additional contact information for the research team will be provided in the email. The email notifications include a unique URL link assigned to each participant, which allows for duplicated submissions to be automatically eliminated. Only the first completed submission from a URL link will be included in the data set; however, it should be noted that participants do not have to complete the survey in one sitting. The REDCap survey can be completed at the individuals' convenience, on various platforms (smartphone, laptop, desktop, tablet), and in multiple sittings (e.g., it can be started one day and finished at a later date). Targeted participants will be given the option to complete the survey online via REDCap, in person, or over the telephone. No compensation is offered for participation.

Following established procedures based on Dillman and colleagues' Tailored Design Method,[78] a weekly email reminder will be sent to non-responding target participants for up to 4 weeks at optimal times (e.g., not on a Friday afternoon). This email will include a link to provide consent and complete the REDCap survey as well as instructions on how to complete the survey by telephone or in person (see *Attachment: Weekly Reminder*). This email reminder will also include an attached PDF of the informed consent and survey for participants to fax or mail in. If telephone numbers are available, target participants may also be contacted by telephone to highlight the importance of the study for the specific RS's state and for reducing opioid related

overdose deaths, as well as to reiterate that all responses are confidential (see *Attachment: Phone Script*).

At 4 weeks after the initial contact, non-respondents may be sent a packet that includes a letter on the RS's institutional letterhead requesting participation, a paper version of the consent form and survey, and a pre-paid addressed envelope (see *Attachment: 4-Week Follow-Up*). The packet will also provide instructions for completing the survey via REDCap or in person. During these contacts, participants may be given the choice of completing the survey online via REDCap, over the telephone, or in person with research staff. In addition, research staff also have the option of conducting in-person visits with non-respondents in their respective jails. Research staff will bring a hard copy of the consent form and survey as well as information on how to complete the survey online. HCS research staff will have up to nine months to collect data from the HCS Annual Jail Survey.

12.4 Appendix D: Additional Site-Led HCS Research Activities

HCS RSs may seek approval from the Steering Committee and NIDA for site-led research activities. Steering Committee and NIDA approved protocols, once approved by the IRB, may be implemented by a single site, two to three sites, or as many as all sites, based on local preference.

12.4.1 Group Model Building Workshop

The New York RS will conduct a group model building (GMB) workshop (see *Group Model Building Workshop Facilitation Manual*) that aims to advance the site's understanding of how community coalitions conceptualize the organizational dynamics that affect OUD and how the CTH is implemented in their communities. GMB is a qualitative method used to develop a causal loop diagram or systems dynamic model, which are constructions of explicit models, or representations, of a system's behavior, considering multiple actors, factors, levels, and the interconnections and feedbacks among them. Systems models can help test the potential effect of interventions and identify the points of maximum leverage in the system.

Community coalition members and other local stakeholders will be asked to participate in the GMB workshop. During the GMB workshop, participants will present their understanding of the driving factors and etiology of OUD in the context of their community's organizational dynamics. Based on this information, a causal loop diagram or systems dynamic model will be developed.

Participants will not be forced to disclose or discuss any information they do not feel comfortable sharing, and the confidentiality of study participants' information will be protected throughout the general study protocols. The GMB process focuses on documenting participants' perceptions and ideas about the opioid crisis and potential solutions at the community level. No individual health information will be collected. In addition, participant names or identifying information will not be used in research reports or presentations.

12.4.2 GetNaloxoneNow

Overdose education is part of the ORCCA EBP selection for communities. All four RSs will provide HCS communities with the option of using www.GetNaloxoneNow.org. GetNaloxoneNow is an online resource to train people to respond effectively to an opioid overdose emergency. The content of the GetNaloxoneNow module draws on years of scholarship and contributions of many experts in overdose prevention including public health researchers, harm-reduction specialists, physicians, nurses, police officers, firefighters, emergency medical technicians, case managers, outreach workers, graphic designers, professional actors, and educators. Funding to develop the training module was provided by NIH/NIDA, Grant #1R43D033746-01 and Grant #1R43DA029358-01A1 (PI: Dr. Janie Simmons). This online overdose education is an evidence-based practice that includes a bystander (i.e., general public) module and a first responder module. The GetNaloxoneNow bystander training module is about 20 minutes, and the first responder training module is approximately 45 minutes.

The four HCS RSs will obtain monthly data analytics on the number of individuals who start and complete the bystander and the first responder training module by county and state through Co-Investigator Dr. Simmons. As part of the registration process for this existing training, demographics are collected, including race/ethnicity, preferred language, gender, age range,

self-report of overdose, or witnessed overdose, as well as other questions related to how they heard about the training. There are also post-training questions to assess the ability to intervene in the case of overdose.

HCS sites will receive only de-identified aggregate data describing participants; therefore, informed consent is not required.

De-identified data will include the following:

- Demographics including race/ethnicity, preferred language, gender, age range, self-report of overdose, or witnessed overdose
- County, state of residence, and ZIP Code
- Questions related to how they heard about the training
- Post-training module questions to assess ability to intervene in case of overdose

Data transfers will occur monthly during the period from January 1, 2020, through March 31, 2023. Data will be stored in local, approved informatics environments for at least 3 years.

12.4.3 Brandeis University Collaboration to Conduct Interviews with Health Care Payers

Brandeis University is collaborating with the Massachusetts RS, and Brandeis University staff will conduct semi-structured interviews with health care payers in Massachusetts, as part of the HEALing Communities Study (HCS). This activity is site specific to Massachusetts and is supervised by the HCS's Implementation Science Core at Boston Medical Center.

The interviews are intended to gather information about the extent to which payers currently fund or otherwise encourage activities that communities may adopt as part of the HCS intervention, such as medications for opioid use disorder (MOUD), distribution of naloxone, or initiatives in schools, pharmacies and law enforcement settings. There is also interest in the extent to which payers would be willing to fund those activities in the future, and how they make decisions about which activities to fund or otherwise support. The resulting information may be helpful to determine the long-term sustainability of the system interventions that are being tested in the HCS communities.

The Brandeis University team will seek to interview officials at MassHealth (the state Medicaid program) and at commercial insurers that serve Massachusetts customers. In 2018, the Massachusetts Division of Insurance identified 13 commercial insurers serving Massachusetts. The team will identify the official at each institution most likely to have knowledge about policies toward opioid use, MOUD, and other topics of interest. For insurers that contract out the management of substance use disorder treatment to an external vendor, the team will also seek to interview that vendor (e.g., for MassHealth: Beacon Health Options, and the Medicaid managed care plans). A total of up to 20 interviews will be conducted. In some cases, these persons are already known to the team from prior research with local health plans. In other cases, the team will use other information to identify the best respondent, using contacts from professional networks and information from company websites and elsewhere.

The team will contact potential participants by email, to explain the HCS study and ask if they are willing to participate in an interview. If an official agrees to an interview, the team will schedule the interview. The team will ask the respondent to complete a survey (via REDCap) that includes a few pre-interview questions on factual issues, such as whether their plan covers certain specific opioid use disorder-related services, whether it provides incentive payments to providers, etc.

The interview will last up to one hour and will be an open-ended discussion. Each respondent will be asked a series of open-ended questions about their institutional policies and activities in relation to MOUD or naloxone, including both use of reimbursement/incentives and other forms of encouragement to providers. The interview will not ask respondents about their own beliefs or their own experience with health care. The interviews will be conducted annually for the duration of the study. After baseline, the team will ask about changes since baseline, and what effect, if any, the HCS initiative played in any resulting policy changes.

Neither the pre-interview survey nor the interview will collect PHI/PII from the respondent.

The interview will be audio recorded. The recording is for the use of the Brandeis University research team in transcribing the interviews and will not be shared with anyone outside of the research team. The recording may include the respondent's name, but it will be removed from the transcribed interview form and a study ID number substituted in its place. The audio files and transcription will be saved on a secure server at Brandeis University. Audio files will be destroyed within 6 months following transcription; transcription files will be kept for 3 years following project completion and then destroyed.

Brandeis University will analyze the interview transcripts using qualitative data techniques. The team will summarize the results in annual reports to the Boston Medical Center's HCS leadership team and may use the results in future publications.

12.4.4 Academic Detailing and CECentral Online Modules

To facilitate and operationalize ORCCA EBP Requirement 3: Safer Opioid Prescribing and Dispensing at the service provider level, and address barriers for rural healthcare providers, the Kentucky RS has developed Academic Detailing and On-Demand Online Continuing Education modules through CECentral. Components include Academic Detailing (in-person training) as well as online modules for Pain Management for Pharmacy, MOUD for Pharmacy, Safer Opioids and Overdose Risk Reduction, and Naloxone Training. Audience will be dentists, nurse practitioners (primary care), pharmacist, pharmacy technicians, and physicians (primary care). Educational content meet accreditation standards, compliance documentation, and outcomes assessment for AMA PRA Category 1 Credits™, ACPE contact hours/CEUs, Dentistry credit, and participation certificate, as well as House Bill 1 requirements for dentists, nurse practitioners, and physicians. Both Academic Detailing and On-demand Online CE modules will be provided free of charge.

As part of the registration process, CECentral requires pre-activity evaluation questions and post-activity evaluation questions. To assess the impact of Academic Detailing and On-Demand Online Education on safer opioid prescribing and dispensing, the Kentucky RS will receive de-identified data describing registrants. Because data received will be de-identified, informed consent is not required.

De-identified data may include the following:

- Demographics (profession)
- Practice Information (county of primary practice)
- Pre-activity questions to assess knowledge, attitudes, and current practice standards/processes
- Post-activity questions to evaluate knowledge, change in attitudes, and impact on practice standards/processes
- Post-activity questions to assess the delivery and content of educational modules and speakers

Data transfers will occur quarterly (March 31, June 30, September 30, December 31) beginning September 30, 2020, through March 31, 2023. Data will be stored in secure, local, approved informatics environments for at least 3 years.

12.4.5 MOUD Organization Interview Guide

University of Kentucky research staff will conduct small group semi-structured interviews with staff working in organizations that provide medication for opioid use disorder (MOUD) in Kentucky, as part of the HEALing Communities Study (HCS). This activity is site-specific to Kentucky and is supervised by the HCS's Implementation Science and Treatment teams at the University of Kentucky.

The interviews are intended to gather information about barriers to access and retention in MOUD as well as the impacts of COVID on the delivery of MOUD within the Kentucky HCS communities. The resulting information may be helpful in explaining the potential impacts of efforts to implement aspects of Menu 2 in the Opioid-overdose Reduction Continuum of Care Approach (ORCCA).

The University of Kentucky team will seek to conduct small group semi-structured interviews with staff working in organizations providing MOUD, which include, but are not limited to, opioid treatment programs (OTPs), non-OTP specialty substance use disorder programs, and office-based medical practices. It is anticipated that approximately 2-3 staff from a given organization will participate in the interview, but some interviews may be conducted with a single individual. To identify potential interviewees, the team will draw upon information provided by the community's HCS coalition, individuals in MOUD organizations that have already implemented fast-track overdose education and naloxone distribution, the team's professional networks, the Drug Enforcement Agency's list of waivered providers, and MOUD organizations' websites. It is anticipated that efforts will be made to conduct interviews with all agencies that have been identified for Menu 2 implementation within the eight Wave 1 communities; similar interviews will be conducted once HCS moves into Wave 2. Up to 250 individuals will be interviewed.

The team will contact potential participants by email or telephone to explain the HCS study and ask if they are willing to participate in a small group interview. Each small group interview will only include individuals from a single organization. If individual(s) from the organization agree to an interview, the team will schedule the interview. During the interview, participants will be asked open-ended questions about the organization's MOUD census, resources needed to increase the patient census, barriers to MOUD access and retention, differences in barriers

between types of MOUD (if the organization offers more than one type of MOUD), the impact of the COVID-19 pandemic on MOUD delivery and use of telehealth for MOUD. Because of the semi-structured nature of the interview, interviewers may probe for clarity or greater detail. The interview guide will not ask individuals about their personal beliefs or their personal experience with health care. A brief demographic REDCap survey, using the IRB approved *HCS Demographic Form* will be used to collect information on race, ethnicity, gender, age, and education after the interview.

Neither the interview nor post-interview demographic survey will collect PHI/PII from the respondent.

The interview will be audio-recorded and professionally transcribed. Identifying information will not be shared with anyone outside of the research team. The recording may include the names of individuals or organizations but identifying information will be removed from the interview transcript and a study ID number substituted in its place. The audio files and transcription will be saved on a secure server at the University of Kentucky and destroyed 3 years following project completion. Demographic data will be stored within REDCap on secure servers.

The University of Kentucky team will analyze the interview transcripts using qualitative data techniques. Findings may be reported in peer-reviewed manuscripts, but confidentiality will be maintained by not identifying any individuals or organizations in publications.

12.4.6 Pharmacy Study Interview Guide & Survey

The New York research site will conduct a survey with selected pharmacies and Pharmacists, as well as interviews with Pharmacists and community members as part of the HEALing Communities Study (HCS). This activity is site-specific to New York and is supervised by Columbia University School of Social Work Associate Research Scientist Dawn Goddard-Eckrich, Ed.D.

The survey and interviews are intended to assess racial and ethnic disparities in Medication for Opioid Use Disorder (MOUD) and Naloxone availability at pharmacies in HCS communities, and to examine the perspectives of People with Opioid Use Disorder (PWOUD) with respect to barriers to accessing MOUD and Naloxone services in pharmacies. There is also interest in using these findings to inform the use of pharmacies to promote MOUD and Naloxone as part of the HCS intervention and to increase engagement, participation, and leadership of more ethnic minorities in the community coalitions and overall HCS study.

Members of the New York research team will seek to survey (via two different surveys [one for pharmacy staff or pharmacists and one specifically for licensed pharmacists]) a sample of Pharmacists, pharmacy technicians and/or other pharmacy staff who work for pharmacies in New York’s HCS communities. The NYS Pharmacy Association will provide recruitment assistance to the research team that will also conduct an online search for pharmacy contact information. The surveys will be programmed in REDCap and will include questions about access to Narcan/Naloxone, Buprenorphine, Naltrexone, Methadone and COVID-19 testing in pharmacies in the HCS communities. The surveys will take between 20 and 30 minutes to complete. Respondents can choose to participate via a REDCap link sent to their email or to complete the survey by phone with a NY research site staff member.

Members of the New York research team will also conduct semi-structured one-on-one interviews with a sample of community members (including African Americans and Hispanics) with a goal of interviewing 20 People with Opioid Use Disorder (PWOUD) and 20 pharmacists in HCS communities.

Through the assistance of its community coalitions, the New York team will recruit individuals and receive referrals from three types of programs serving PWOUD at sites in NY's HCS counties with diverse communities: (a) one methadone maintenance treatment program (MMTP); (b) one primary care clinic; and (c) one syringe exchange program (SEP). Recruitment will ensure that the sample includes minority representation across non-Hispanic Blacks and Hispanics.

New York research staff will explain the study to potential PWOUD participants and verify their age and drug use experience. To be eligible, PWOUD study participants must be: 18 years of age or older, have used heroin, cocaine, and/or methamphetamine within the past 12 months, and/or received, or currently receive services from an opioid treatment, HIV primary care, or a syringe exchange program.

The research team will contact potential participants by email to explain the HCS study and ask if they are willing to participate in an interview. If the potential participant agrees to participate, the team will schedule the interview. The interview will last approximately 45 minutes and will be an open-ended discussion. Each respondent will be asked a series of questions about key facilitators, barriers to availability and overlap in MOUD services (e.g., Narcan/Naloxone, Buprenorphine, and Naltrexone) and COVID services (e.g., testing, vaccine, medication. [e.g., Remdesivir], availability, etc.) provided in pharmacies, as well as access to and distribution (e.g., mail order, drive thru, in person) of different types of Naloxone, Buprenorphine, Naltrexone and other Medications for Opioid use Disorder (MOUD) in pharmacies in NY HCS communities.

The interview will be audio recorded. The recording is for the use of the New York research team in transcribing the interviews and will not be shared with anyone outside of the research team. The recording may include the respondent's name, but it will be removed from the transcribed interview form and a study ID number substituted in its place. The audio files and transcription will be saved on a secure server at Columbia University. Audio files will be destroyed within 6 months following transcription; transcription files will be kept for 3 years following project completion and then destroyed.

The New York research site will analyze the interview transcripts using qualitative data techniques. The team will summarize the results in a report to the New York Research Site's HCS leadership team and may also be included in future publications.

12.4.7 CE Facilitator Community Genogram Model

The Ohio research site will use a community genogram model that aims to advance the site's understanding of how community organizations, agencies, and providers are related to and connected with one another. The process of completing the genogram and the visualization of these relationships will inform the community-facing and research staff in understanding the resources and challenges within communities. The genogram is an engagement tool for the field staff and assists with understanding the changing landscapes in the HCS counties.

Community Engagement Facilitators (CE-Fs) will engage in a listening tour throughout their community starting with coalition leadership. This process will allow the CE-F to learn about the relevant community members, organizations, and systems. As a part of the listening tour, CE-F's will transcribe information gathered from the listening tour into the Community Engagement Facilitator – Community Assessment Tool (CAT).

Coalition leaders and other community members will not be forced to disclose or discuss any information they do not feel comfortable sharing, and the confidentiality of the gathered information will be protected. No personal health information will be collected as part of the listening tour. The Ohio research site will use the Community Genogram information to inform CTH implementation and assist in strategies to leverage engagement throughout the study. Findings may be reported in presentations, reports, and manuscripts, but confidentiality will be maintained.

12.4.8 Policy Barriers and Facilitators Tracking

The HEALing Communities Study (HCS) will carry out a policy tracking activity through the following 4 aims.

Aims

Aim 1) Data collection: Document policy issues (e.g., policy facilitators, barriers or misunderstandings) that could impact the implementation or effectiveness of the Communities That HEAL (CTH) intervention including identification of which components may be impacted (e.g., evidence-based practices known to reduce opioid overdose deaths and strategies to implement these evidence-based practices).

Aim 2) Technical Assistance: Through training and technical assistance, engage key stakeholders and community partners to identify, leverage, and address policy issues impacting the use of evidence-based practices to reduce opioid overdose deaths.

Aim 3) Feedback: Provide feedback to policymakers and regulators through our Key Government Officials, federal partners (NIDA/SAMHSA), and our state Community Advisory Boards about policies that may impact implementation of the CTH intervention.

Aim 4) Analysis: Anticipate the probable effects of identified policies on study outcomes, and whether they are likely to mediate or moderate the impact of the CTH intervention, in order to address the policies appropriately in evaluating the effect of CTH.

While each HCS research site will pursue each aim, the relative emphasis may differ across the research sites.

Methods

The overall goal of this activity is to collect information on governmental and organizational policies relevant to the implementation of the HCS (Aim 1). This includes policies that could modify the impact of the Communities That Heal (CTH) intervention, as well as other policies that could affect the outcomes being targeted (e.g., opioid overdose deaths; risky prescribing). Study team members at the research sites will document and collect information on policies that facilitate or impede the CTH intervention in a REDCap tracking system. Research sites may also document in the tracking system the support(s) they provide communities on overcoming these barriers and/or by sharing information with communities (Aim 2).

The HCS research team will pursue two strategies to identify policies that are relevant to the implementation of evidence-based practices and evaluation of their outcomes for the CTH intervention. Research team members will systematically conduct a contextual analysis of policy changes using an environmental scan of publicly available sources (e.g., government websites, press releases). In addition, the policy tracking team will seek community reports of policy issues from study staff or others, such as community advisory board members or coalition members.

For this research activity, policy is defined as a set of principles intended to guide decisions and behavior within an organization or larger system, which includes formal laws, but also the rules, guidelines, practices, and protocols guiding organizations and jurisdictions. Examples include the following: laws of federal and state legislatures; guidelines, rules, regulations of federal agencies and national associations; organizational policies of health insurers; and rules and regulations of state and local health departments.

Information on policies collected by the HCS research team will be shared with communities in order to support them in overcoming any barriers or to facilitate implementation of the CTH intervention. The research team will also provide feedback to policymakers and regulators through our Key Government Officials, federal partners (NIDA/SAMHSA), and our state Community Advisory Boards about policies that may impact implementation of the CTH intervention (Aim 3).

We are seeking a waiver of informed consent for these activities as we are not collecting identifiable information about an individual's behavior or health. Data will be collected from public websites and documents as well as from research team members. More than one person may also be involved in providing information for the community reports.

This activity will be conducted throughout the study period.

Analysis

Data collected as part of this research activity will be available to use in study analyses to account for the probable effects of identified policies on study outcomes, and whether they are likely to mediate or moderate the impact of the CTH intervention (Aim 4). This will afford researchers a better understanding of how the policy landscape may affect the HCS. Descriptive reports on policies and data collection activities may also be published to disseminate information to broader audiences.

Risks/benefits

There are no risks to the community or individual study participants. Benefits for the community include receiving support to overcome policy barriers and more easily access information about policies.

12.4.9 Costing the Evidence Based Practices

HEALing Communities Study (HCS) staff on the Health Economics Core in Kentucky, Massachusetts and New York will conduct semi-structured interviews with key informants from implementing organizations to understand the costs of implementing Evidence Based Practice (EBP) Strategies. The interviews are intended to gather information about start-up and operational costs, such as time spent on activities required to stand up the EBP strategies and provide

ongoing services, as well as additional resources (e.g., volunteer time, non-HCS funds, and donated items) that are required to implement and sustain each strategy. The resulting information will be used to determine costs of the EBP Strategies in the HCS communities in Kentucky, Massachusetts, and New York.

The research teams will seek to interview 1-2 key informants at implementing organizations in their Wave 1 HCS Communities. The teams will identify the key informant(s) at each institution most likely to have knowledge about start-up and operational costs. A total of up to thirty interviews per research site will be conducted. In most cases, these persons are already known to the HCS team members who work directly with the implementing organizations.

Research staff from the Health Economics Core will contact potential participants by email to explain the HCS study and ask if they are willing to participate in an interview via phone or video conference. The interview may last between 10-45 minutes depending on the role and knowledge of the key informant. If a key informant agrees to participate, the team will schedule the interview. The research team will document the key informant's name, role, and contact information in REDCap before the interview. During the interview, key informants will be asked a series of questions to estimate the start-up and operational costs of the EBP strategy their organization is implementing. The research staff member will document the responses on the "Template for Costing the Evidence Based Practices" data collection instrument in REDCap. Following the first interview, the research team may contact the key informants again to invite them to participate in a follow-up interview to ask about changes in operational costs over time.

The interview will not collect PHI/PII from the respondent.

12.4.10 Identifying the Municipal Policies that Impact the Implementation of the EBP Strategies

At the HEALing Communities Study (HCS) Massachusetts' research site, community implementation has revealed a number of municipal level factors that may have important implications for the translation of local Evidence Based Practice (EBP) strategies. For example, zoning ordinances and codes have impacted implementation plans involving Opioid Use Disorder (OUD) treatment facilities and mobile health services in MA HCS communities. Understanding municipal policies that influence implementation as well as effective strategies for working with municipal key players may inform local efforts to translate EBP strategies. Just as important, engaging with local stakeholders may help to facilitate the sustainability of these strategies over time. The HCS MA research site will aim to develop an understanding of local policies that may impact the implementation of EBP strategies in their HCS communities.

To develop an understanding of the local policies, the MA research site will conduct interviews with key informants and a document review. Key informants include HCS MA community staff supporting Wave 1 communities (e.g., community coordinators), Wave 1 coalition members, Community Advisory Board (CAB) members, municipal stakeholders in Massachusetts (for example mayors, city council members, public health officials, etc.), and leaders from communities with existing drug strategies. For this research activity, drug strategies are defined as municipal policies and programs enacted to respond to the opioid epidemic. The interviews will last an hour to an hour and a half depending on the role of the key informant (interviews with leaders from communities with existing drug strategies will last an hour and a half).

No more than 50 key informants will be recruited and interviewed. The MA research team will develop a list of potential interviewees from internal study records, their knowledge of the

communities, and public searches. To gauge interest, potential participants will be contacted by the research team through email, no more than three times. If participants do not respond to one of the three emails, then they will receive a follow-up phone call or text. HCS MA staff who participate in the interview will be asked for their feedback on who in the coalition could be a potential interviewee. Over the next two years, participants who participated in the first interview may be contacted to participate in a follow-up interview, if needed, or asked to participate in community engagement activities around municipal drug policies. Participation in the interviews or activities is always voluntary.

MA HCS study staff will not be compensated as they will complete interviews during their working hours. Community members (CAB and Coalition members) and municipal leaders within the HCS MA communities and other communities with drug strategies will be offered a $25 gift card for their participation in the interviews.

There are no direct benefits in participating in these interviews. Potential community benefits include advancing our understanding of the role municipal policies and practices have in supporting the implementation and sustainability of the role of the EBP strategies that were introduced as part of the HCS.

There are no direct risks associated with participation in these interviews beyond potential loss of confidentiality for low-risk information related to the implementation of EBP strategies. No identifiable information will be disclosed in reports or manuscripts.

The interview will be audio and/or video recorded. The recording is for the use of the MA research site in transcribing the interviews and will not be shared with anyone outside of the research team. The recording may include the respondent's name but identifying information will be removed from the recording and transcriptions will be deidentified. The deidentified recordings and transcriptions will be saved on a secure server at the Boston University School of Social Work. Recordings will be destroyed within 6 months following transcription; transcription files will be kept for 3 years following project completion and then destroyed.

In addition to conducting interviews, the MA research site will qualitatively analyze documents including the HCS implementation plans, media and policy scans conducted by the HCS communications team and the policy workgroup, documents related to municipal drug policies and strategies, and community related news. Documents will be collected through community alerts/notifications, implementation plans, and a scan of the municipality website.

Data will be collected on an ongoing basis between October 2021-July 2022. Interviews and documents will be analyzed using both content and thematic analysis to understand what is known or being done related to municipal drug policies. They will also be used to identify themes that can be translated into recommendations for communities in Massachusetts to support community-driven design and enforcement of municipal drug policies. Findings may be used in future reports and manuscripts.

12.4.11 Photovoice Participatory Research Technique

The HEALing Communities Study (HCS) Kentucky, Massachusetts and Ohio research sites will use the Photovoice participatory research technique to identify community-specific strengths and barriers along the continuum of care to prevent opioid overdose deaths in HCS communities. Community implementation of the Communities that Heal Intervention (CTH) has

revealed a number of both unique and common facilitators and barriers to the continuum of care across participating communities (e.g., existence of services, distance to services, stigma, cultural beliefs, transportation, homelessness). Understanding the opioid epidemic as experienced by community members, including perceptions of existing resources, strengths, and barriers, can inform local strategic efforts to prevent overdose deaths.

Photovoice is a participatory research technique that invites participants to take photos related to a chosen challenge in their community. The photos and descriptions are then shared and discussed during focus group sessions to explore ways to address the challenge. Over the course of the HCS Photovoice sessions in participating communities, participants 1) develop photo assignments related to the opioid epidemic, 2) take representative photos to explore barriers and strengths related to how their communities are responding to the opioid epidemic, 3) use photos as a trigger for a focus group discussion, and 4) participate in a focus group session focused on each photo assignment. Focus group discussions will be guided by the SHOWeD method which uses selected photos as triggers to collectively explore what is observed in the photo, reflect on what the photo represents, interpret how it came to be and currently affects their lives and the community, and propose actions to address the issue. Results will then be disseminated more broadly with community members. At minimum, we will encourage sharing results with the community's HCS coalition to discuss ways to combat the opioid epidemic locally. HCS staff, facilitators, and participants will also discuss other means of dissemination, potentially including but not limited to presentation(s) at HCS meeting(s), presentations to larger community or external forums, community photo displays, and billboards.

While the overarching goal of the Photovoice projects in HCS communities is to understand barriers and facilitators that impact efforts to prevent opioid-related overdose deaths, HCS communities will be able to hone the focus of their inquiry to the local context and decide on specific photo topics to prepare for each focus group session. For example, a rural community with no public transport options may be interested in focusing one session on exploring transportation, while an urban community with a geographically distant opioid treatment program may be interested in using a session to explore how to expand access to medicines for opioid use disorder within their community and surrounding areas.

The Photovoice projects will be conducted with key community informants, which include community members who can provide insight about the state of the opioid epidemic and local community resources and responses. Key informants for this effort may include current and former opioid users, behavioral health providers, addiction treatment providers, public health and social service staff, harm reduction service providers, first responders, members of cultural or faith-based institutions, and others with experience, insight, and knowledge of the community.

The HCS community facing team will develop a list of potential key informants. To gauge interest, key informants will be contacted by the research or community teams through email or phone no more than three times, or via in person communication during meetings in the community. No more than 16 key informants will be recruited from each individual community. To keep group size and conversation manageable, individual focus group sessions will be limited to 8 participants. Focus groups may be specific to an individual community, or they may be cross-community/county and include individuals from Wave 1 and/or Wave 2 communities/counties.

The proposed Photovoice projects in the HCS communities will include up to 6 sessions. Participants will be offered site-specific compensation up to $50 per session:

**Session**	**Activities**	**Time Commitment**	**Incentive**
Orientation	• What is Photovoice?<br>• Photovoice process<br>• Ethics of Photography<br>• Informed consent<br>• Photo topic development	1.5 hours	up to $50
Session 1	• Focus group: Discuss photos deemed by participants to be most representative of the chosen topic; collaboratively develop proposed actions<br>• 2nd photo topic development	1.5-2 hours	up to $50
OPTIONAL: Session 2	• Focus group<br>• 3rd photo topic development	1.5-2 hours	up to $50
OPTIONAL: Session 3	• Focus group<br>• 4th photo topic development	1.5-2 hours	up to $50
OPTIONAL: Session 4	• Focus group	1.5-2 hours	up to $50
Final session	Review results and develop community dissemination plan	1.5 hours	up to $50
**Total**		**4.5 – 11 hours**	**Up to $300**

Depending on access to smartphones, photos and brief descriptions of photos may be submitted via the HIPAA compliant app EpiCollect5. This app also offers a GIS function where location of photos could be included if desired by facilitators. Otherwise, photos will be captured on digital cameras provided to participants.

If they agree to participate in the Photovoice project, key informants will be asked to complete a brief (5-minute) demographics survey so the study team can gather background characteristics of the participants. The survey will ask demographic questions including age, gender, race/ethnicity, education, county/community represented, and personal connection to substance use disorder. In order to keep the demographic information confidential and de-identified, the participant's responses will not be connected to their name.

Photovoice sessions may happen in person or via Zoom; they will be audio recorded. The recordings will not be shared with anyone outside of the HCS research team. Audio recordings will be transcribed. The recording may include the respondent's name but identifying information will be removed from the recording and transcriptions will be de-identified. The de-identified recordings and transcriptions will be saved on a secure server at the HCS research site.

Recordings will be destroyed within 6 months following transcription; transcription files will be kept for 3 years following project completion and then destroyed.

Data will be analyzed in two stages. Following each session, focus group facilitators will code barriers and facilitators impacting the community's efforts to address the opioid epidemic and strengthen the continuum of care in their community. The analysis will be reported back to participants at the start of the next session as a form of quality control. A thematic outcome analysis will be completed by HCS researchers and interested participants at project conclusion. Findings may be used in future reports and manuscripts.

Each HCS community will have the opportunity to consider whether they are interested in implementing a Photovoice project. Participation in the Photovoice project is voluntary, and communities and participants may decline or discontinue participation at any time. There is no cost to participate, and there are no direct risks associated with participation in this project beyond potential loss of confidentiality for low-risk information related to the opioid epidemic in their community. No identifiable information will be disclosed in reports or manuscripts. Participants will sign an informed consent form, and any individuals depicted in the photos or audio recordings will sign a Permission to Use Photographs form and indicate approved use of photos in community dissemination or research activities.

There are no direct benefits for the participant involved in this project. Potential community benefits include advancing our understanding of local barriers and strengths in preventing opioid overdose deaths to inform strategic decision making, and results can be used in communications and dissemination activities.

Photovoice groups may elect to evaluate efficacy and/or impact of Photovoice and dissemination activities as reflected in responses to programmatic surveys. HCS RS may receive deidentified data collected during the course of these Photovoice “administrative” activities.

PROCESS:

- Point person, through their administrative role in Photovoice operations, will remove identifying information.
- Point person will then transfer deidentified data to HCS research team. If available, deidentified data may include impact of the Photovoice exhibit (i.e., understanding community strengths and concerns, community experience with opioid epidemic, impact of images, takeaway message, experience with Photovoice, among other relevant topics), the individual's role or connection to Photovoice and/or substance use disorder as well as demographics. County served is a programmatic requirement for HCS, and if available, will be retained.
- Point person on HCS research team will store these data on a secure, local, approved informatics environment with access limited to delegated members of the HCS research team.
- Delegated members of the HCS research team will extract and/or aggregate information as necessary to evaluate the efficacy of Photovoice and dissemination

research activities.

The HCS research team will not be able to identify individuals surveyed. Data will be stored in secure, local, approved informatics environments for 3 years after the conclusion of the study.

12.4.12 Race and Ethnicity Data Collection Readiness Survey

At the HEALing Communities Study (HCS) New York research site, community implementation has revealed a difference in racial and demographic data collection across partner organizations. Understanding the process that an organization is using to collect and monitor demographic data may inform us on identifying challenges and successes of our community partners gathering demographic information. To develop an understanding of demographic data collection, the NY HCS research site will conduct a survey with key informants working at our HCS partner organizations. From this survey, the team plans to learn about the perceived and actual barriers preventing our county partners from obtaining race, ethnicity, gender, sex, language and/or age data from the population their organization serves. The aim of this survey is to gain a deeper understanding of health inequity in these community settings and address them through targeted education and learning collaboratives. Between 60 to 70 Wave 1 organizations will be recruited to participate in the survey. The NY HCS research team will develop a list of potential survey participants from the internal study records. To gauge interest, potential participants will be contacted by the research team through email, no more than three times. If participants do not respond to one of the three emails, then they will receive a follow-up phone call or text. Participants will be asked to complete this survey once; there are no additional follow-up surveys.

Participation in this survey is voluntary. There will be no compensation for participating in the survey. There are no direct benefits in participating in these surveys. Potential community benefits include advancing our understanding of perceived and physical barriers preventing our county partners from obtaining race, ethnicity, gender, sex, language and or age data from their patient population.

There are no direct risks associated with participation in these surveys beyond potential loss of confidentiality for low-risk information related to an organizations system of collecting demographic data. Participants will be asked to provide their name, email address, name of their organization, job title, and their organization’s zip code. This information will be stored separately from the information collected about the process their organization uses to collect and monitor demographics data. All collected information will be stored in secure, encrypted to protect confidentiality. We will not share name and contact information with anyone outside of the research team. All data will be assigned a unique, coded participant ID. No identifiable information will be disclosed in reports or manuscripts.

12.4.13 PARTNER Tool

The HEALing Communities Study (HCS) Ohio research site’s community engagement team will conduct a social network analysis by administering the PARTNER Tool to Wave 2 county coalitions. The PARTNER tool is a 24-item survey that will be given to HCS community coalition members which asks about 1) their views on the coalition as a whole and 2) their interactions with other coalition members. The purpose of this research activity is to better understand the role coalition members (i.e., agencies) play within the coalition, what resources each agency

brings to the table, identify activity levels of agencies in the coalition, and determine how these agencies interact to address the opioid epidemic in their communities/counties.

HCS Ohio study staff will work with coalition leaders to build a list of potential participants for each Wave 2 county/community. Potential participants will be sent an introductory email, explaining the PARTNER Tool survey. This email will explain to potential participants that they can either complete the survey online or over the phone. If the potential participant wants to complete the survey over the phone, the email provides contact information for a study staff member. This staff member will verbally consent the participant prior to beginning the survey. If the potential participant chooses to complete the survey online, they will review the informed consent information (provided in the email) prior to completing the online survey. Participants can contact Ohio study staff if they have questions or concerns about the study and are given time to decide if they would like to participate. The survey will be repeated once, at the end of the study period.

The survey, data, and analysis tools will be housed online through the Visible Network Labs (VNL) group who own the PARTNER Tool. Participant survey data will be collected and stored on VNL's HTTPS encrypted servers. The data is only accessible through the VNL web interface and requires a login. Ohio HCS study staff will be given a 'manager-level' login where they can access participant data and the analysis tool which can generate network maps and scores. Study staff will be able to track the number of started and completed surveys which will be used to track how many individuals declined to participate (i.e., those that are listed as "Not Started" on the VNL website). Only HCS Ohio staff will communicate directly with participants (e.g., reminder emails, phone call, etc.).

Using the data and analysis tool from the online VNL portal, study staff will generate summary reports for each county/community coalition. These reports will only describe network-level outcomes such as centralization or density and will not report on specific coalition member scores. The Ohio HCS Community Engagement team will work with coalition leaders to present the results to the coalition and discuss implications (e.g., what sectors are underrepresented in the coalition, which resources are being contributed or not contributed). Ohio HCS Community Engagement Facilitators will also use the results as a tool to better understand coalition connections and function.

12.4.14 Medication Disposal Program Pharmacy Interviews

To monitor ORCCA EBP Requirement 3: Safer opioid prescribing and dispensing, the Kentucky Research Site (RS) will conduct individual or small group semi-structured interviews with staff at community pharmacies who partnered with HCS to increase access to safe medication disposal through installation of a medication disposal drop box. This activity is site-specific to Kentucky.

The interviews are intended to gather information about satisfaction with the experience, barriers to implementation and maintenance, readiness for sustainment of drop boxes, training or technical needs related to drop box maintenance, and the inner construct of the pharmacy. The resulting information may be helpful in explaining how overcoming previously identified barriers to pharmacy-based medication disposal can increase safe medication disposal options in a community and potentially decrease the amount of medication, including prescription opioids that are available for misuse or accidental poisoning. The information may also identify new barriers and facilitators.

HCS Disposal Program Coordinators will seek to conduct one-on-on or small group semi-structured interviews with staff working in pharmacies who participated in the HCS medication disposal program. It is anticipated that the pharmacy owner or manager will participate in the interview, but some interviews may include other pharmacy staff. Efforts will be made to conduct interviews with all pharmacies within HCS communities that have installed a medication disposal drop box.

The team will contact potential participants by email no more than 2 times and by telephone no more than 3 times to explain the purpose of the interview and ask if they are willing to participate. If the pharmacy declines the interview, an offer to discuss only sustainability will be made. In the event that the pharmacy is unable to schedule any type of interview, a one-page sustainability summary will be sent to the pharmacy for review.

Each small group interview will typically include individuals from a single pharmacy, but in the case of a small pharmacy chain or an individual owning multiple pharmacies, more than one pharmacy may be represented. If individual(s) from the organization agree to an interview, they will be given a link to schedule the interview, or the research team will schedule the interview. During the interview, participants will be asked open-ended questions. Because of the semi-structured nature of the interview, interviewers may probe for clarity or greater detail. The interview guide will not ask individuals about their personal beliefs or their personal experience with health care.

The interview will be audio-recorded and transcribed. Identifying information will not be shared with anyone outside of the research team. The recording may include the names of individuals but identifying information will be removed from the interview transcript and a pharmacy ID number substituted in its place. The audio files and transcription will be saved on a secure server at the HCS Kentucky research site. Recordings will be destroyed within 6 months following transcription; transcription files will be kept for 3 years following project completion and then destroyed.

12.4.15 Mobile Interventions for Increasing Access to Medications for Opioid Use Disorder (MOUD)

The HEALing Communities Study (HCS) Massachusetts, New York and Ohio research sites will conduct semi-structured interviews with key informants from implementing organizations in HCS communities to understand the facilitators of and barriers to implementing mobile MOUD interventions (e.g., interventions that provide mobile access to clinicians who prescribe buprenorphine, naltrexone, or methadone). The interviews are intended to gather information about services offered, barriers and facilitators to standing up such programs, as well as additional factors that are required to implement and sustain each strategy. The resulting information will be used to develop a set of best practices for other organizations seeking to implement mobile MOUD programs.

The research team will seek to interview 1-2 key informants from implementing organizations in Wave 1 HCS communities that have created or are creating mobile MOUD interventions, including five communities in Massachusetts, five in New York, and one in Ohio. The team will identify the key informant(s) at each organization most likely to have knowledge about implementation planning and operations of the mobile MOUD programs. Up to thirty-three interviews will be conducted. In most cases, these key informants are people already known to the HCS research team.

Research staff will contact potential participants by email to explain the HCS study and ask if they are willing to participate in an interview via phone or video conference (Zoom). The interview may last up to an hour, depending on the role and knowledge of the key informant. If a key informant agrees to participate, the team will schedule the interview. The research team will document the key informant's name, role, and contact information in REDCap before the interview. During the interview, key informants will be asked a series of questions to explore the experiences of standing up mobile MOUD interventions. Key informants will be offered a $50 gift card or a pre-paid debit card (ClinCard) for their participation.

The interviews will be audio recorded. The recordings will not be shared with anyone outside of the HCS research team. Audio recordings will be transcribed. The recording may include the respondent's name but identifying information will be removed from the recording and transcriptions will be de-identified. The audio files and transcriptions will be saved on a secure server at the research site. Audio files will be destroyed within 6 months following transcription; transcription files will be kept for up to 3 years following project completion and then destroyed.

Aside from participants' names and organizational affiliation, the interview will not collect PHI/PII from the respondent. No identifiable information will be included in presentations or publications of results.

12.4.16 A Cost Analysis of Peer Recovery Support Services

Opioid Use Disorder (OUD) is a chronic brain disease which has historically been treated through a process more appropriate for acute conditions. This short-term care, which has largely been focused on stabilizing a patient's emergent condition, has failed to lead to optimal longer-term remission and recovery outcomes. Due to the lack of effective ongoing treatment, as many as 3-9% individuals with a prior opioid overdose are readmitted for opioid dependence or another overdose within one year. Additionally, beginning a treatment program is often just one step in a complex recovery process as substance use disorders (SUDs) are often accompanied by additional mental, social, legal, occupational, and other medical needs.

Peer Recovery Services are growing in popularity throughout the nation as a means of assisting individuals with SUDs by providing emotional, informational, and instrument support. Numerous studies have proven the effectiveness of these services in improving care and recovery outcomes for individuals with SUDs. However, a common theme among these analyses is that services are often not standardized, and the term peer is often applied generally and does not specify training or services provided by an individual. As a result of these findings, systematic reviews suggest identifying a clearer description of peer recovery support roles and responsibilities. Additionally, while these reviews mention that the use of peer recovery services may result in cost savings through decreased emergency and hospital services, none of the studies reported on the cost of peer recovery itself.

To operationalize and build capacity for ORCCA EBP Requirement 2: Effective delivery of MOUD, including agonist/partial agonist medication and outreach and delivery to high-risk populations, the Kentucky (KY) HEALing Communities research site (RS) has partnered with a nonprofit community recovery organization, Voice of Hope, which trains and manages numerous peer recovery specialists to provide MOUD treatment linkage and retention services. Because HCS provides funding support for peer recovery specialists, this partnership offers a unique opportunity to assess the effectiveness of peer support services in the delivery

of MOUD as well as the impact of implementing Evidence Based Practices (EBPs) in partnering organizations. Obtaining accurate cost information for peer recovery services is critical for evaluating program effectiveness and informing future policy decisions. Due to the current lack of studies in this field, the goals of this HCS research activity are to build a set of standard service categories for OUD peer recovery support services which are provided by one agency in Kentucky. Once completed, associated time and resource costs for each component will be calculated. Understanding the costs of this intervention targeting OUD within a highly impacted state like Kentucky, and the impact of peer support services in the delivery of MOUD in highly impacted HCS communities, will provide greater insight into its economic viability and support researchers, practitioners, and policymakers in determining future funding allocations for combatting this epidemic.

The KY HCS Research Site (RS) will answer the following research questions (RQ) through this new HCS research activity:

- RQ1: What are the common peer recovery service categories used to provide support for OUD recovery?
- RQ2: What roles and resources are needed for operating a peer recovery support organization?
- RQ3: What are the unit costs for specific services provided by peer recovery support organizations?

The KY HCS RS will examine peer recovery support services provided by our partners at Voices of Hope (VOH). Data collection will be limited to information on organizational costing. Because no human subjects are being studied and no PII or PHI is being collected, informed consent is not required, and Waiver of Consent is requested.

A two-pronged approach will be used to assess organizational costing for peer recovery services:

1. The KY RS will work with VOH to classify a set of standard service categories and the types of staffing required to provide these services. Using these data, the Substance Abuse Services Cost Analysis Program (SASCAP) Labor Module has been adapted for Peer Recovery Services Agencies and will be administered to staff at VOH. The SASCAP Labor Module is a validated instrument that will be used to identify the amount of time spent among the different job types on the standard set of peer recovery services. This instrument will be administered by the HCS KY Health Economics team through an electronic survey with follow-up conversations with VOH as needed to clarify survey responses. The SASCAP Labor Module has been submitted as part of this modification.
2. The KY RS will use the SASCAP Cost Module to estimate agency-level annual operating costs. The SASCAP Labor Module is designed to do activity-based costing and is combined with the SASCAP Cost Module to do program/agency-level costing. Together these provide an estimate of total annual operating costs of the agency plus the cost per specific service. The SASCAP Cost Module has been submitted as part of this modification.

Data will be collected with the SASCAP instruments, which are Word or PDF documents that can be edited. The KY RS team will work directly with a key informant from VOH agency (CEO or similar that has access to financial records and other pertinent information on resources to deliver services). These data will be transferred to an Excel spreadsheet. Once collected, cost data will be organized in a statistical software program (e.g., STATA) and will be analyzed using algorithms designed by RTI to produce cost analysis results. Results will be reported as total annual agency cost, average cost per client served, and average cost per service type.

Data collection will be limited to organizational costing. All data collected from these research activities will be stored in KY's local, secure, approved informatics environment for 3 years after the conclusion of the study.

12.4.17 Wave 1 Partner Organization Sustainability Interview

To expand knowledge regarding the experiences of Wave 1 partner organizations in implementing ORCCA (Opioid-overdose Reduction Continuum of Care Approach) Menus 1 and 2 and to learn about the period of early sustainment (approximately 6 months after the end of Wave 1), HCS staff from all four research sites will conduct small group and/or individual semi-structured interviews with staff working in organizations that partnered with the HEALing Communities Study (HCS). The interviews are intended to gather information about internal, external, and intervention-related factors that facilitated or impeded implementation as well as how these factors are impacting the ability of organizations to sustain these evidence based practices (EBPs). The resulting information may be helpful in contextualizing the efforts to implement aspects of Menus 1 and 2 as well as broaden our understanding of barriers to and facilitators of sustainment.

Trained HCS Research Site (RS) staff will conduct small group and/or individual semi-structured interviews with staff working in Wave 1 partner organizations that implemented EBPs from Menu 1 and/or Menu 2. Approximately 50 partner organizations per HCS site will be asked to participate in the small group semi-structured interviews (approximately 200 total across all four RSs). It is anticipated that 2-3 staff from a given organization will participate in the interview (some interviews may be conducted with a single individual depending on interest within the organization) for an estimated sample size of about 450 participants.

To identify potential interviewees, the team will draw upon internal site databases to identify a purposive sample that includes a range of organizations, including those located in rural and urban communities, those that are or are not represented on the coalition, and those in the three primary sectors of HCS (health care, behavioral health, and criminal justice). Efforts will be made to conduct interviews with all Wave 1 communities, recognizing that the distribution of organizations between communities is likely to vary based on the strategies chosen by the respective HCS coalitions.

Trained HCS RS staff will contact potential participants by email or telephone to explain the purpose of this data collection and ask if they are willing to participate in a small group or individual interview. Interviews will be conducted by video conference or by telephone. If conducted in a small group format, the interview will only include individuals from a single organization. If individual(s) from the organization agree to an interview, the team will schedule the interview. After providing verbal informed consent, participants will be asked open-ended questions (see Wave 1 Partner Organization Sustainability Interview Guide). Because of the semi-structured nature of the interview, interviewers may probe for clarity or greater detail. The

interview guide will not ask individuals about their personal beliefs or their personal experience with health care. At the end of the interview, using the IRB approved *HCS Demographic Form,* information on race, ethnicity, gender, age, and education will be collected. Each individual who participates will receive $50 (in the form of check, cash card, or gift card), unless state, government, and employer regulations or policies do not permit employees to receive compensation for participating in studies.

The interview will be audio-recorded and transcribed. Identifying information will not be shared with anyone outside of the research team. The recording may include the names of individuals or organizations but identifying information will be removed prior to inclusion in any study-related report. The audio files and transcripts will be saved on a secure server at each RS and destroyed no later than 3 years following project completion.

STATISTICAL ANALYSIS PLAN

Do communities implementing the CTH intervention have significantly lower overdose death rates?

Proposal Number: MP0057

SAP VERSION : Version 6.0

SAP DATE : November 29, 2023

PI/SPONSOR: Sharon Walsh (University of Kentucky)
Nabila El-Basel (Columbia University)
Jeffrey Samet (Boston Medical Center)
Bridget Freisthler (Ohio State University)
LaShawn Glasgow (RTI International)
Emmanuel Oga (RTI International)
Gary Zarkin (RTI International)
Redonna Chandler (NIDA)
Jennifer Villani (NIDA)

PREPARED BY: RTI International and the HCS Statistics and Data Analysis Work Group (StaDA-WG)

AUTHOR (S): Rouba Chahine, Lauren D'Costa, and Sylvia Tan for the HEALing Communities Study Data Coordinating Center Statistical Operations (DCC StatOps) group.

SAP REVIEWER (S):
Philip Westgate (University of Kentucky), Debbie Cheng (Boston University), Daniel Feaster (University of Miami), Soledad Fernandez (Ohio State University) for the HCS Statistics and Data Analysis Work Group (StaDA-WG).

Contents

1 ADMINISTRATIVE INFORMATION 3

1.1 SAP REVISION HISTORY 3

1.2 LIST OF ABBREVIATIONS 4

2 INTRODUCTION 5

2.1 OBJECTIVES 5

2.2 HYPOTHESES 5

3 STUDY METHODS 6

3.1 TRIAL DESIGN 7

3.2 RANDOMIZATION 7

3.3 STATISTICAL INTERIM ANALYSIS AND STOPPING GUIDANCE 7

3.4 TIMING OF ANALYSIS 7

4 STATISTICAL PRINCIPLES 7

4.1 CONFIDENCE INTERVALS AND P-VALUES 7

4.2 ANALYSIS POPULATIONS 8

5 STATISTICAL ANALYSIS 8

5.1 OUTCOME DEFINITIONS 8

5.2 ANALYSIS METHODS 10

5.2.1 Statistical Methods 10

5.2.2 Assumption Checks 12

5.2.3 Alternate Analysis Methods 12

5.2.4 Sensitivity Analysis 12

5.2.5 Subgroup Analysis, ITT 13

5.3 MISSING DATA 14

5.4 ADDITIONAL ANALYSES 14

5.4.1 ORCCA 15

5.5 STATISTICAL SOFTWARE 16

5.6 LIST OF POTENTIAL DISPLAYS 16

6 REFERENCES 17

7 APPENDICES 18

7.1 APPENDIX 1: STUDY RANDOMIZATION 18

1 ADMINISTRATIVE INFORMATION

1.1 SAP Revision History

Provide revision history for SAPs that have been approved (i.e., no outstanding comments or questions) only. For instance, each row should correspond to Version 1.0, 2.0, 3.0, etc. that includes all changes that were made to the SAP from the previous version.

Ver.	Justification for change	Date
1.0	Initial version (approved by StaDA-WG)	2022-12-07
2.0	• Indicated that adjusted rates within each wave will be reported<br>• Clarified reporting of sub-group analyses<br>• Revised analysis of tertiary measures<br>• Revised list of potential displays<br>• Provided a description of the modeling approach that will be used to address missing data if such issue arises	2023-03-10
3.0	• Changed model analysis language and FDR adjustment plan<br>• Moved measures 1.3 & 1.4 to secondary and 1.5 to tertiary. Request made by WT and approved by Dan Feaster and StaDA-WG<br>• Updated Tables language	2023-05-12
4.0	• Added language to section 5.2.5 to indicate that individual point estimates will be provided for all subgroups for descriptive purposes at the urging of the HCS SC who made this request at their meeting 2023-06-30	2023-07-05
5.0	• Added language to section 5.2.4 to indicate post-hoc sensitivity analyses that will be conducted.<br>• Revised language in section 5.2.5 to describe reporting of individual point estimates and confidence intervals for subgroups.<br>• Added language to section 5.4 to indicate post-hoc analyses that will be conducted.	2023-08-18
6.0	• Added language to section 5.4 to indicate post-hoc estimation methods for “death averted” calculation.	2023-11-29

1.2 List of Abbreviations

Abbreviation:	Definition:
$	Dollar
%	Percent
±	Plus or Minus
≤	Less than or equal to
<	Less than
=	Equals
≥	Greater than or equal to
>	Greater than
CTH	Communities That HEAL
DCC	Data Coordinating Center
HCS	HEALing Communities Study
ITT	Intent-to-treat
KY	Kentucky
MA	Massachusetts
N/A	Not Applicable
NIDA	National Institute on Drug Abuse
NY	New York
OH	Ohio
RS	Research site
SAP	Statistical analysis plan

2 INTRODUCTION

The HEALing Communities Study (HCS)[1] is a multi-site, parallel group, cluster randomized wait-list comparison trial to test the immediate impact of implementing an integrated set of evidence-based practices from the Communities That HEAL (CTH) framework. The CTH will be delivered across healthcare, behavioral health, justice, and other community-based settings in highly affected communities. The goal of the HCS is to reduce opioid overdose deaths by 40% in three years.

This document outlines the statistical analysis plan that will be followed to address the research objective that are of interest in MP0057.

2.1 Objectives

MP0057 has the following research objectives:

Primary Objective

1. To compare the rate of drug overdose deaths in an adult during the evaluation period between Waves 1 and 2.

Secondary Objectives

2. To compare the rate of opioid overdose deaths involving any opioid and any psychostimulant (excluding cocaine) in an adult during the evaluation period between Waves 1 and 2.
3. To compare the rate of opioid overdose deaths involving any opioid and cocaine in an adult during the evaluation period between Waves 1 and 2.
4. To compare the rate of opioid overdose deaths involving any opioid and any benzodiazepine in an adult during the evaluation period between Waves 1 and 2.

Tertiary Objectives

5. To describe the rate of opioid overdose deaths involving heroin in an adult during the evaluation period between Waves 1 and 2.
6. To describe the rate of opioid overdose deaths involving synthetic opioids except methadone in an adult during the evaluation period between Waves 1 and 2.
7. To describe the rate of opioid overdose deaths involving any opioid and any psychostimulant (including cocaine) in an adult during the evaluation period between Waves 1 and 2.
8. To describe the rate of drug overdose deaths involving any psychostimulant (excluding cocaine) in an adult during the evaluation period between Waves 1 and 2.
9. To describe the rate of drug overdose deaths involving cocaine in an adult during the evaluation period between Waves 1 and 2.
10. To describe the rate of drug overdose deaths involving any psychostimulant (including cocaine) in an adult during the evaluation period between Waves 1 and 2.
11. To describe the rate of drug overdose deaths involving any benzodiazepine in an adult during the evaluation period between Waves 1 and 2.

2.2 Hypotheses

MP0057 has the following primary research hypothesis:

1. The rate of drug overdose deaths in an adult during the evaluation period in Wave 1 is lower than Wave 2.

   The null (H_0) and alternative (H_A) statistical hypotheses are:

   > H_0: The rate of drug overdose deaths in an adult during the evaluation period in Wave 1 is equal to Wave 2.
   >
   > H_A: The rate of drug overdose deaths in an adult during the evaluation period in Wave 1 is not equal to Wave 2.

Secondary research hypotheses:

2. The rate of opioid overdose deaths involving any opioid and any psychostimulant (excluding cocaine) in an adult during the evaluation period in Wave 1 is lower than Wave 2.

   The null (H_0) and alternative (H_A) statistical hypotheses are:

   > H_0: The rate of opioid overdose deaths involving any opioid and any psychostimulant (excluding cocaine) in an adult during the evaluation period in Wave 1 is equal to Wave 2
   >
   > H_A: The rate of opioid overdose deaths involving any opioid and any psychostimulant (excluding cocaine) in an adult during the evaluation period in Wave 1 is not equal to Wave 2.

3. The rate of opioid overdose deaths involving any opioid and cocaine in an adult during the evaluation period in Wave 1 is lower than Wave 2.

   The null (H_0) and alternative (H_A) statistical hypotheses are:

   > H_0: The rate of opioid overdose deaths involving any opioid and cocaine in an adult during the evaluation period in Wave 1 is equal to Wave 2
   >
   > H_A: The rate of opioid overdose deaths involving any opioid and cocaine in an adult during the evaluation period in Wave 1 is not equal to Wave 2.

4. The rate of opioid overdose deaths involving any opioid and any benzodiazepine in an adult during the evaluation period in Wave 1 is lower than Wave 2.

   The null (H_0) and alternative (H_A) statistical hypotheses are:

   > H_0: The rate of opioid overdose deaths involving any opioid and any benzodiazepine in an adult during the evaluation period in Wave 1 is equal to Wave 2.
   >
   > H_A: The rate of opioid overdose deaths involving any opioid and any benzodiazepine in an adult during the evaluation period in Wave 1 is not equal to Wave 2.

3 STUDY METHODS

This section contains information about the study design and statistical analysis that will be performed to assess the research objectives and statistical hypotheses outlined in Section 2.

3.1 Trial design

The HCS is a multi-site, parallel group, cluster randomized wait-list comparison trial composed of 67 communities from four Research Sites (RS): Kentucky (KY), Massachusetts (MA), New York (NY), and Ohio (OH). Communities were assigned to receive the CTH intervention group (Wave 1, N = 34 communities) or the wait-list comparison group (Wave 2, N = 33 communities).

3.2 Randomization

The 67 HCS communities were randomly assigned to Wave 1 (CTH intervention) or Wave 2 (wait-list control) according to Appendix 1. Randomization was stratified by RS. For each RS, covariate-constrained randomization (Moulton, 2004)[2] was used to ensure balance between Wave 1 and Wave 2 communities on three key community characteristics at baseline:

1. opioid overdose death rate averaged over 2016 and 2017
2. population size
3. urban versus rural status.

Given the nature of the research, no blinding was performed in this study.

3.3 Statistical Interim Analysis and Stopping Guidance

There are no interim efficacy analyses, safety analyses, or interim stopping rules that must be considered for this SAP.

3.4 Timing of Analysis

The evaluation period for the CTH intervention is from July 1, 2021 through June 30, 2022. Data collection for the evaluation period is expected to be complete within 6-9 months from the end of the evaluation period. Subsequent data cleaning and analysis will be completed within 3-6 months and dissemination of results will take place immediately after.

4 STATISTICAL PRINCIPLES

4.1 Confidence Intervals and P-values

All statistical computations will be performed by HCS biostatisticians from the Data Coordinating Center Statistical Operations group (DCC StatOps). For descriptive summaries of study data, the following will be presented:

- Nominal/categorical measures will be summarized using frequencies and percentages;
- Interval or ratio scale measures will be summarized using means, standard deviations, medians, 95% confidence intervals, 25th and 75th percentiles, and ranges;
- Ordinal measures will be summarized depending on the number of levels. An ordinal measure with five levels or less will be summarized as a nominal measure. An ordinal measure with more than five levels will be summarized as an interval or ratio scale measure.

The balance or imbalance of these characteristics will be studied and reported, particularly for analyses comparing the two study arms. Graphical displays will be used to show distributions (box plots, density curves). The reported p-values will be based on two-sided tests at an $\alpha=0.05$ unless otherwise specified. When p-value correction is appropriate, Benjamini-Hochberg (1995)[3] False Discovery Rate adjustments will be used to address multiplicity and preserve Type I error rate.

Unless required otherwise by a journal, the following rules are standard:

- Moment statistics including mean and standard deviation will be reported at 1 more significant digit than the precision of the data.
- Order statistics including median, minimum, and maximum values will be reported to the same level of precision as the original observations. If any values are calculated to have more significant digits, then the value should be rounded so that it is the same level of precision as the original data.
- Following SAS rules, the median will be reported as the average of the two middle numbers (by order) if the dataset contains an even number of observations.
- Test statistics including t and z test statistics will be reported to two decimal places.
- P-values will be reported to 3 decimal places if > 0.001. If less than 0.001, p-values will be reported as '<0.001'. P-values will be reported as 0.05 rather than .05.
- No preliminary rounding should be performed; rounding should only occur after analysis. To round, consider digit to right of last significant digit: if < 5 round down, if ≥ 5 round up.

4.2 Analysis Populations

The analyses of each outcome will be performed using an intention-to-treat (ITT) framework, defined as the inclusion of all communities randomized into the HCS and analyzed according to the trial arm to which the community was randomized.

If specified, a sensitivity analysis of an outcome will also be performed using a Per Protocol (PP) framework, defined as the inclusion all randomized communities who complete the HCS protocol with no major deviations and analyzed according to the trial arm to which the community was randomized.

5 STATISTICAL ANALYSIS

5.1 Outcome Definitions

Table 1 provides the definitions for the outcomes that will be used to address the statistical hypotheses listed in Section 2.2.

Table 1. Outcome Definitions

Objective	Outcome	Technical Specifications Ref.	Source
1 (Primary)	Number of drug overdose deaths	2.1	Death certificates data
2 (Secondary)	Number of opioid overdose deaths involving any opioid and any psychostimulant (excluding cocaine)	1.3	Death certificates data
3 (Secondary)	Number of opioid overdose deaths involving any opioid and cocaine	1.4	Death certificates data
4 (Secondary)	Number of opioid overdose deaths involving any opioid and any benzodiazepine	1.6	Death certificates data
5	Number of opioid overdose deaths involving heroin	1.1	Death certificates data
6	Number of opioid overdose deaths involving synthetic opioids except methadone	1.2	Death certificates data
7	Number of opioid overdose deaths involving any opioid and any psychostimulant (including cocaine)	1.5	Death certificates data
8	Number of drug overdose deaths involving any psychostimulant (excluding cocaine)	2.1.1	Death certificates data
9	Number of drug overdose deaths involving cocaine	2.1.2	Death certificates data
10	Number of drug overdose deaths involving any psychostimulant (including cocaine)	2.1.3	Death certificates data
11	Number of drug overdose deaths involving any benzodiazepine	2.1.4	Death certificates data

5.2 Analysis Methods

5.2.1 Statistical Methods

The models used to evaluate the statistical hypotheses in Section 2.2 will test the effect of the CTH intervention on each outcome between Wave 1 and Wave 2 communities using methods described in Westgate (2022)[4]. To address each objective, a negative binomial regression model will be fit to the corresponding outcome in Section 5.1 as the dependent variable with trial arm (Wave 1 versus Wave 2) included as the main independent variable.

Table 2 provides a description of the dependent variable that will be used to address each objective, the statistical model that will be fit, the offset term that will be included in the model, and a list of covariates that will be included as fixed effects in the model. Small sample adjusted empirical standard error estimates will also be applied using the average of the small-sample corrected empirical estimators proposed by Ford and Westgate (2017)[2] (SAS options given by "empirical=FIRORES" and "empirical=root" respectively) and degrees of freedom equal to the number of communities minus the number of regression parameters. In the unlikely event this estimator is computationally infeasible, one of the following SAS options will be used: "empirical=FIRORES", "empirical=FIROEEQ", or "empirical=DF". If needed, a similar test can be conducted for each of the individual parameters described above.

The results of this model for each outcome will be used to estimate and report:

1. The adjusted relative rate (and 95% CI and p-value) of the outcome in the population of interest during the evaluation period from July 1, 2021 to June 30, 2022 between Wave 1 and Wave 2.
2. The adjusted rate (and 95% CI) of the outcome in the population of interest during the evaluation period from July 1, 2021 to June 30, 2022 within Wave 1.
3. The adjusted rate (and 95% CI) of the outcome in the population of interest during the evaluation period from July 1, 2021 to June 30, 2022 within Wave 2.

Note that adjusted rates and 95% CIs will be calculated using least squares means.

Table 2 provides a description of the dependent variable that will be used to address each hypothesis, the statistical model that will be fit, the offset term that will be included in the model, and a list of covariates that will be included as fixed effects in the model.

We note that secondary outcomes will be analyzed and reported similar to the approach used for primary outcomes. However, secondary outcomes should be interpreted differently in the manuscript with less focus, regardless of results, than primary outcomes.

Table 3 provides description of tertiary variables. Tertiary outcomes will only be reported using descriptive statistics. Descriptive statistics include the means and SDs of the number of events and population for each outcome as well as of the raw sum of the number of events population for each outcome during the evaluation period by wave using ITT.

Table 2*. Statistical Models

Hypothesis	Dependent Variable[1]	Model	Offset[1]	Covariates[2]
1	Number of drug overdose deaths during the evaluation period	Negative Binomial	Natural log of the community population size of individuals 18 years of age or older during the evaluation period	A,B,C,D
2	Number of opioid overdose deaths involving any opioid and any psychostimulant (excluding cocaine) during the evaluation period	Negative Binomial	Natural log of the community population size of individuals 18 years of age or older during the evaluation period	A,B,C,D
3	Number of opioid overdose deaths involving any opioid and cocaine during the evaluation period	Negative Binomial	Natural log of the community population size of individuals 18 years of age or older during the evaluation period	A,B,C,D
4	Number of opioid overdose deaths involving any opioid and any benzodiazepine during the evaluation period	Negative Binomial	Natural log of the community population size of individuals 18 years of age or older during the evaluation period	A,B,C,D

1. The population that will be used for the offset:
   - P.1.1: County-defined community population denominator, 18+
   - P.2.1: Zip code–defined community population denominator, 18+
2. The covariates that will be included in the model:
   A. State (KY, MA, NY, OH)
   B. Rural/Urban
   C. Natural log of the observed baseline community level opioid overdose death rate as measured from January 2019 to December 2019. The observed baseline community level opioid overdose death rate will be calculated using the ratio of the observed number of opioid overdose deaths as measured from January 2019 to December 2019 to the observed community population size of individuals 18 years of age or older measured during 2020 (county-defined) or 2021 (zip-code defined). If there are any communities where the baseline community level opioid overdose death rate is 0, the log transformation cannot be applied and so the baseline community level opioid overdose death rate will be used instead.
   D. Natural log of the observed baseline rate of the dependent variable from January 2019 to December 2019 within a community. If there are any communities where the observed baseline rate of the dependent variable from January 2019 to December 2019 is 0, a log transformation cannot be applied and so the observed baseline rate of the dependent variable will be used instead

Table 3. Tertiary Outcomes

Objective	Dependent Variable[1]
5	Number of opioid overdose deaths involving heroin during the evaluation period

Objective	Dependent Variable[1]
6	Number of opioid overdose deaths involving synthetic opioids except methadone during the evaluation period
7	Number of opioid overdose deaths involving any opioid and any psychostimulant (including cocaine) during the evaluation period
8	Number of drug overdose deaths involving any psychostimulant (excluding cocaine) during the evaluation period
9	Number of drug overdose deaths involving cocaine during the evaluation period
10	Number of drug overdose deaths involving any psychostimulant (including cocaine) during the evaluation period
11	Number of drug overdose deaths involving any benzodiazepine during the evaluation period

1. Population that will be used as denominator:
   - P.1.1: 2020 county-defined community population denominator, 18+
   - P.2.1: 2021 zip code-defined community population denominator, 18+

5.2.2 Assumption Checks

It is possible that for some outcomes, a Poisson model could be used in lieu of a negative binomial model. To evaluate this, the overdispersion parameter, k, of the negative binomial model will first be estimated using PROC GLIMMIX and its 95% confidence interval will be estimated by refitting the model using PROC GENMOD as PROC GLIMMIX does not generate a confidence interval for k. If the negative binomial is appropriate for the data collected, the value of k should reflect overdispersion and be a positive value, but if k is close to zero then GLIMMIX may fail to converge. If PROC GLIMMIX fails to converge or k itself is less than 0 in the PROC GENMOD fit, then k will be set to 0 and a working Poisson model will be fit to the data in PROC GLIMMIX.

5.2.3 Alternate Analysis Methods

A negative binomial response is assumed (overdispersion parameter, $k > 0$). If this assumption does not hold ($k \leq 0$), then a Poisson response may be adequate. If the negative binomial model does not fit adequately, as mentioned in Section 4.2.2, then k will be set to 0 and a Poisson model will be fit to the data.

5.2.4 Sensitivity Analysis

Primary and secondary models will be replicated using a PP framework (as defined in Section 4.2)

The following sensitivity analyses were conducted after Version 4.0 of the SAP was implemented and results disseminated.

1. To assess the impact of using negative binomial or Poisson regression to model the primary and secondary outcomes during the evaluation period, for each outcome, we will compare the following:
    a. The effect of the CTH intervention on the outcome during the evaluation period under a GEE-type negative binomial regression model.
    b. The effect of the CTH intervention on the outcomes during the evaluation period under a GEE-type Poisson regression model.
2. To assess the impact of assuming that the effect of urban/rural status on the rate of drug overdose deaths during the evaluation period is the same across research sites, we will compare the following:
    a. The effect of the CTH intervention on the rate of drug overdose deaths during the evaluation period under a model that does not include an interaction between urban/rural status and research site.
    b. The effect of the CTH intervention on the rate of drug overdose deaths during the evaluation period under a model that includes an interaction between urban/rural status and research site.

5.2.5 Subgroup Analysis, ITT

For Hypotheses 1 the following subgroup analyses will be performed:

- RS (KY, MA, NY, OH)
- Rural/urban status of the community
- age (18–34, 35–54, 55+)
- sex (male, female, missing)
- race/ethnicity (Hispanic, Non-Hispanic White, Non-Hispanic Black, Non-Hispanic Other, missing).

For Hypotheses 2 through 4, the following subgroup analyses will be performed:

- RS (KY, MA, NY, OH)
- Rural/urban status of the community

For each hypothesis, a separate model will be fit for each stratification variable using an ITT framework. Each model will be similar to that used for the primary analysis described in Section 5.2.1, but will also include fixed effects for the stratification variable as well as an interaction between intervention and the stratification variable.

To account for multiple comparisons arising from multiple interaction tests within a manuscript and multiple comparisons from pairwise comparisons within a manuscript, a two-stage approach will be used as follows:

- Stage 1 – All p-values from all interaction tests will be gathered together and the Benjamini-Hochberg (1995)3 procedure will be applied to obtain FDR adjusted p-values.

• Stage 2 – Amongst all interaction tests where the FDR adjusted p-value is statistically significant at a 0.05 level, all p-values from all corresponding pairwise tests will be gathered together and the Benjamini-Hochberg (1995)[4] procedure will be applied to obtain FDR adjusted p-values.

For each test of effect modification, adjusted rates within Wave 1 and 2 and the adjusted relative rate between Wave 1 and 2 within each level of a stratification variable (as well as 95% simultaneous CI using a Bonferroni correction that were added after Version 4.0 of the SAP was implemented and results disseminated) will be reported, in addition to the FDR-adjusted p-value associated with the interaction test.

- If the test for effect modification is not statistically significant at the 0.05 level, no further reporting will be performed.
- If the test for effect modification is statistically significant at the 0.05 level, the following will be reported:
    - estimate of the ratio of the relative rate between Wave 1 and 2 (as well as 95% simultaneous CI using a Bonferroni correction) between any 2 subgroups; and
    - pairwise FDR-adjusted p-value between any 2 subgroups.

5.3 Missing data

Missing data may arise when the number of events and population for an outcome are reported in separate levels of a stratification variable (e.g., race/ethnicity, sex, age) and one of the levels of the stratification variable is 'missing' because there is a subset of individuals for which the stratification variable is missing. A missing level of a stratification variable will be removed from the subgroup analyses described in Section 5.2.5. However, the missing level of a stratification variable will be summarized using descriptive means by wave (Table 2 series) and descriptive sums by wave (Table 3 series).

Missing data may also arise when outcome data is suppressed at a research site. For suppressed data at the community-level, multiple imputation for missing data (Rubin 19875) will be performed using SAS PROC MI (SAS/STAT® 15.2 User's Guide6). Twenty imputations will be used for analysis (Allison 20127). SAS PROC MIANALYZE (SAS/STAT® 15.2 User's Guide6) will be used to combine results across imputations. Multiple imputation will not be performed to account for suppressed data at the subgroup level within a community (e.g., race/ethnicity, sex, age). In this case, subgroups with suppressed data will be excluded from the analysis. Regardless of the approach taken, the amount of suppressed data will be summarized.

5.4 Additional Analyses

After Version 4.0 of the SAP was implemented and results were disseminated, one additional analysis was conducted to estimate the effect of the CTH intervention of the relative rate of drug overdose deaths between the evaluation and baseline periods. In this case, a repeated measures marginal model (similar to those described in Section 5.2.5 for age, sex, and race/ethnicity subgroup analyses) was fit to the number of opioid overdose deaths during the baseline and evaluation periods. Fixed effects included wave, research site, urban/rural status, time period (baseline or evaluation period), and a two-way interaction between wave and time period as well as an offset

term given by the natural log of the community population size during the most recent year from available data. A residual covariance structure was specified to account for correlation among outcomes measured at baseline and evaluation periods within the same community. In this case, the effect of the CTH intervention was estimated using the ratio (and 95% CI and p-value) between the following two estimates:

- The adjusted relative rate (and 95% CI) of opioid overdose deaths between the evaluation and baseline periods in Wave 1 communities.
- The adjusted relative rate (and 95% CI) of opioid overdose deaths between the evaluation and baseline periods in Wave 2 communities.

Another analysis was requested after the SAP was implemented and results disseminated, the study team requested an estimate of the number of “deaths averted” from implementing the CTH intervention in Wave 1. Two methods were used to estimate the number of deaths averted: (1) a crude method that does not account for overdispersion or covariates, and (2) a model-based average marginal effects method (Greene, 2000) using the estimated parameters from the negative binomial used for the primary analysis.

1. Crude method: apply the observed rate in Wave 2 communities (number of deaths/population, the Wave 1 counterfactual) to the Wave 1 population. We then subtract the actual observed deaths from the Wave 1 counterfactual to get the estimated deaths averted.
2. Negative binomial based method: use the parameter estimates from the negative binomial model used for the primary analysis to estimate Wave 1 predicted deaths using Wave 2 parameters (Wave 1 counterfactual). We then used the Wave 1 parameters to estimate Wave 1 predicted deaths (Wave 1 as Wave 1). We subtracted the Wave 1 as Wave 1 from the Wave 1 counterfactual to get the estimated deaths averted.

5.4.1 ORCCA

The Opioid-overdose Reduction Continuum of Care Approach ORCCA consists of three menus of evidence-based practices (EBPs) that were implemented in Wave 1 communities. Within each menu, EBPs were implemented in different combinations of strategies (e.g., active OEND at high-risk venues), sectors (e.g., healthcare), and venues (e.g., healthcare-emergency department). Each strategy-sector-venue combination is referred to as a triad. A triad is the primary identifier for a strategy that is planned and then implemented within a community. For each triad that is selected by a community, each community answered questions relating to:

- intent to reach special populations;
- development of an implementation plan for the strategy;
- initiation of the implementation plan;
- the number of partner organizations/practices that are implementing the strategy; and
- a brief description of the strategy.

To summarize the implemented strategies from the ORCCAT across Wave 1 communities, Table 6 will provide frequencies and percentages of strategy and sector for each of Menu 1: Overdose Education and Naloxone Distribution (OEND), Menu 2: Medication for Opioid Use Disorder (MOUD), and Menu 3: Safer Prescribing. Summaries will be presented by research site (KY, MA,

NY, OH), by geographic location (urban, rural), and overall. The table will only include summary statistics on menus that are relevant to the manuscript.

Depending on the scope of the manuscript, frequencies and percentages for specific strategy-sector-venue strategies may also be included. Inclusion will be informed by 1) the ORCCA menu, which maps outcome measures to ORCCA menus and strategies, and 2) the sector and venue definitions provided in Appendix D of the ORCCAT SOP.

5.5 Statistical Software

The SAS statistical package (version 9.4 or higher) will be utilized for all analyses. The R software package may be used to create figures and other graphical displays.

5.6 List of Potential Displays

- Table 1. Baseline Demographic Characteristics of N=67 Communities Participating in the HEALing Communities study by Wave (January 2019 – December 2019)
- Table 2a. Descriptive Means of Number of Drug Overdose Deaths During the Evaluation Period (July 1, 2021 - June 30, 2022) Using the Intention-to-Treat Population
- Table 2b. Descriptive Means of Number of Opioid Overdose Deaths Involving any Opioid and any Psychostimulant (Excluding Cocaine) During the Evaluation Period (July 1, 2021 - June 30, 2022) Using the Intention-to-Treat Population
- Table 2c. Descriptive Means of Number of Opioid Overdose Deaths Involving any Opioid and Cocaine During the Evaluation Period (July 1, 2021 - June 30, 2022) Using the Intention-to-Treat Population
- Table 2d. Descriptive Means of Number of Opioid Overdose Deaths Involving any Opioid and any Benzodiazepine During the Evaluation Period (July 1, 2021 - June 30, 2022) Using the Intention-to-Treat Population
- Table 3a. Descriptive Sums of Number of Drug Overdose Deaths During the Evaluation Period (July 1, 2021 - June 30, 2022) Using the Intention-to-Treat Population
- Table 3b. Descriptive Sums of Number of Opioid Overdose Deaths Involving any Opioid and Psychostimulant (Excluding Cocaine) During the Evaluation Period (July 1, 2021 - June 30, 2022) Using the Intention-to-Treat Population
- Table 3c. Descriptive Sums of Number of Opioid Overdose Deaths Involving any Opioid and Cocaine During the Evaluation Period (July 1, 2021 - June 30, 2022) Using the Intention-to-Treat Population
- Table 3d. Descriptive Sums of Number of Opioid Overdose Deaths Involving any Opioid and Benzodiazepine During the Evaluation Period (July 1, 2021 - June 30, 2022) Using the Intention-to-Treat Population
- Table 4a. Adjusted Rate of Each Outcome Within Waves and Adjusted Relative Rate of Each Outcome Between Wave 1 to Wave 2 Communities in the Intention-to-Treat Population
- Table 4b. Adjusted Rate of Each Outcome Within Waves and Adjusted Relative Rate of Each Outcome Between Wave 1 to Wave 2 Communities in the Per-Protocol Population
- Table 5a. Sub-Group Analyses of Number of Drug Overdose Deaths During the Evaluation Period (July 1,2021 – June 30,2022) Using the Intention-to-treat Population

- Table 5b. Subgroup Analyses of Number of Opioid Overdose Deaths Involving any Opioid and any Psychostimulant (Excluding Cocaine) During the Evaluation Period (July 1, 2021 - June 30, 2022) Using the Intention-to-Treat Population
- Table 5c. Subgroup Analyses of Number of Opioid Overdose Deaths Involving any Opioid and Cocaine During the Evaluation Period (July 1, 2021 - June 30, 2022) Using the Intention-to-Treat Population
- Table 5d. Subgroup Analyses of Number of Opioid Overdose Deaths Involving any Opioid and any Benzodiazepine During the Evaluation Period (July 1, 2021 - June 30, 2022) Using the Intention-to-Treat Population
- Table 6. Implemented Strategies from ORCCAT by Study Site for N=33 Wave 1 Communities Participating in the HEALing Communities Study
    - Summary statistics of the 3 menus can be included in this table. Choice of menus to include can vary by manuscript.
        - Menu 1: Overdose Education and Naloxone Distribution (OEND)
        - Menu 2: Medication for Opioid Use Disorder (MOUD)
        - Menu 3: Safer Prescribing
- Table 7a. Descriptive Means of Tertiary Outcomes During the Evaluation Period (July 1, 2021 - June 30, 2022) Using the Intention-to-Treat Population
- Table 7b. Descriptive Sums of Tertiary Outcomes During the Evaluation Period (July 1, 2021 - June 30, 2022) Using the Intention-to-Treat Population
- Version Information
    - This table will be used to provide information regarding the data used in the analysis (date of site data lock per measure; date of DCC data lock per measure; date of data freeze, date of table generation, SAP version used to generate tables).

6 REFERENCES

1. Westgate PM, Cheng DM, Feaster DJ, Fernández S, Shoben AB, Vandergrift N (2022). Marginal modeling in community randomized trials with rare events: Utilization of the negative binomial regression model. Clinical Trials, 19(2): 162-171.
2. Ford WP and Westgate PM (2017). Improved standard error estimator for maintaining the validity of inference in cluster randomized trials with a small number of clusters. Biometrical Journal, 59: 478-495.
3. Benjamini Y and Hochberg Y (1995). Controlling the False Discovery Rate: A Practical and Powerful Approach to Multiple Testing. Journal of the Royal Statistical Society: Series B (Methodological), 57: 289-300.
4. Rubin DB (1987). Multiple Imputation for Nonresponse in Surveys. John Wiley & Sons Inc., New York.
5. SAS/STAT® 15.2 User's Guide
    - PROC MI: https://documentation.sas.com/doc/en/statug/15.2/statug_mi_toc.htm
    - PROC MIANALYZE: https://documentation.sas.com/doc/en/statug/15.2/statug_mianalyze_toc.htm
6. Allison P (2012). “Why You Probably Need More Imputations Than You Think.” Accessed February 20, 2015. http://www.statisticalhorizons.com/more-imputations
7. Greene, W. H. (2000), Econometric Analysis, Fourth edition, Prentice Hall, 307–312.

7 APPENDICES

7.1 Appendix 1: Study Randomization

Table A1: HCS Wave 1 Communities

Wave	RS	CID	Community Name	Rural/Urban
1	KY	02	BOYD	Urban
1	KY	03	BOYLE	Rural
1	KY	06	CLARK	Urban
1	KY	07	FAYETTE	Urban
1	KY	08	FLOYD	Rural
1	KY	09	FRANKLIN	Rural
1	KY	13	KENTON	Urban
1	KY	15	MADISON	Rural
1	MA	18	BROCKTON	Urban
1	MA	19	PLYMOUTH	Urban
1	MA	20	GLOUCESTER	Urban
1	MA	22	SALEM	Urban
1	MA	23	HOLYOKE	Urban
1	MA	25	LOWELL	Urban
1	MA	28	BARNSTABLE (BOURNE/SANDWICH)	Rural
1	MA	32	MIDDLESEX (SHIRLEY/TOWNSEND)	Rural
1	NY	34	CAYUGA	Rural
1	NY	36	COLUMBIA	Rural
1	NY	38	ERIE	Urban
1	NY	40	GREENE	Rural
1	NY	41	LEWIS	Rural
1	NY	44	PUTNAM	Urban
1	NY	45	SUFFOLK	Urban
1	NY	47	ULSTER	Urban
1	OH	50	ASHTABULA	Rural
1	OH	51	ATHENS	Rural
1	OH	53	CUYAHOGA	Urban
1	OH	54	DARKE	Rural
1	OH	56	GREENE	Urban
1	OH	57	GUERNSEY	Rural
1	OH	58	HAMILTON	Urban
1	OH	61	LUCAS	Urban
1	OH	62	MORROW	Urban
1	OH	64	SCIOTO	Rural

Table A2: HCS Wave 2 Communities

Wave	RS	CID	Community Name	Rural/Urban
2	KY	01	BOURBON	Urban
2	KY	04	CAMPBELL	Urban
2	KY	05	CARTER	Rural
2	KY	10	GREENUP	Urban
2	KY	11	JEFFERSON	Urban
2	KY	12	JESSAMINE	Urban
2	KY	14	KNOX	Rural
2	KY	16	MASON	Rural
2	MA	17	NORTH ADAMS	Urban
2	MA	21	LAWRENCE	Urban
2	MA	24	SPRINGFIELD	Urban
2	MA	26	PITTSFIELD	Urban
2	MA	27	WEYMOUTH	Urban
2	MA	29	BRISTOL (BERKELEY/DIGHTON/FREETOWN)	Rural
2	MA	30	FRANKLIN (GREENFIELD/MONTAGUE/ATHOL/ORANGE)	Rural
2	MA	31	HAMPSHIRE (BELCHERTOWN/WARE)	Rural
2	NY	33	BROOME	Urban
2	NY	35	CHAUTAUQUA	Rural
2	NY	37	CORTLAND	Rural
2	NY	39	GENESEE	Rural
2	NY	42	MONROE	Urban
2	NY	43	ORANGE	Urban
2	NY	46	SULLIVAN	Rural
2	NY	48	YATES	Urban
2	OH	49	ALLEN	Urban
2	OH	52	BROWN	Urban
2	OH	55	FRANKLIN	Urban
2	OH	59	HURON	Rural
2	OH	60	JEFFERSON	Urban
2	OH	63	ROSS	Rural
2	OH	65	STARK	Urban
2	OH	66	WILLIAMS	Rural
2	OH	67	WYANDOT	Rural
