# Supplementary material for: Communities That HEAL Intervention and Mortality Including Polysubstance Overdose Deaths: A Randomized Clinical Trial
Source: JAMA Netw Open. 2024 Oct 21;7(10):e2440006. doi: 10.1001/jamanetworkopen.2024.40006 (PMC11581668; doi:10.1001/jamanetworkopen.2024.40006)
Supplement: Supplement 2. — eTable 1. Descriptive Sums of Primary and Secondary Outcomes During the Baseline Period (January 2019-December 2019) Using the Intention-to-Treat Population eTable 2. Descriptive Means of Primary and Secondary Outcomes During the Evaluation Period (July 1, 2021-June 30, 2022) Using the Intention-to-Treat Population eTable 3. Descriptive Sums of Tertiary Outcomes During the Evaluation Period (July 1, 2021-June 30, 2022) Using the Intention-to-Treat Population eTable 4. Calculation of Proportions of Opioid Deaths Involving Specific Drugs eTable 5. Adjusted Rate of Each Outcome Within Trial Groups and Adjusted Relative Rate of Each Outcome Between Intervention to Control Communities During the Evaluation Period Using the Per-Protocol Population eTable 6. Sensitivity Analyses [file jamanetwopen-e2440006-s002.pdf]

Supplemental Online Content

Freisthler B, Chahine RA, Villani J, et al. Effect of the Communities That HEAL Intervention on Mortality Including Polysubstance Overdose Deaths. *JAMA Netw Open.* 2024;7(10):e2440006. doi:10.1001/jamanetworkopen.2024.40006

eTable 1. Descriptive Sums of Primary and Secondary Outcomes During the Baseline Period (January 2019-December 2019) Using the Intention-to-Treat Population

eTable 2. Descriptive Means of Primary and Secondary Outcomes During the Evaluation Period (July 1, 2021-June 30, 2022) Using the Intention-to-Treat Population

eTable 3. Descriptive Sums of Tertiary Outcomes During the Evaluation Period (July 1, 2021-June 30, 2022) Using the Intention-to-Treat Population

eTable 4. Calculation of Proportions of Opioid Deaths Involving Specific Drugs

eTable 5. Adjusted Rate of Each Outcome Within Trial Groups and Adjusted Relative Rate of Each Outcome Between Intervention to Control Communities During the Evaluation Period Using the Per-Protocol Population

eTable 6. Sensitivity Analyses

This supplemental material has been provided by the authors to give readers additional information about their work.

© 2024 Freisthler B et al. *JAMA Network Open.*

eTable 1. Descriptive Sums of Primary and Secondary Outcomes During the Baseline Period (January 2019 – December 2019) Using the Intention-to-Treat Population

Outcome	Group	Intervention Communities			Control Communities			Overall Rate Ratio[3]
		Total Events[1]	Total Population[1]	Overall Raw Rate[2]	Total Events[1]	Total Population[1]	Overall Raw Rate[2]	
**Drug Overdose Deaths**	**Overall**	2,086	4,439,170	47.0	1,803	3,772,336	47.8	0.98
	**Research Site**							
	Kentucky	313 (15.0%)	617,841 (13.9%)	50.7	425 (23.6%)	815,764 (21.6%)	52.1	0.97
	Massachusetts	182 (8.7%)	359,314 (8.1%)	50.7	231 (12.8%)	356,545 (9.5%)	64.8	0.78
	New York	311 (14.9%)	1,101,497 (24.8%)	28.2	373 (20.7%)	976,069 (25.9%)	38.2	0.74
	Ohio	1,280 (61.4%)	2,360,518 (53.2%)	54.2	774 (42.9%)	1,623,958 (43.0%)	47.7	1.14
	**Urban/Rural Classification**							
	Urban	1,803 (86.4%)	3,793,353 (85.5%)	47.5	1,595 (88.5%)	3,242,663 (86.0%)	49.2	0.97
	Rural	283 (13.6%)	645,817 (14.5%)	43.8	208 (11.5%)	529,673 (14.0%)	39.3	1.12
	**Age**							
	18-34 Years	597 (28.6%)	1,334,880 (30.1%)	44.7	565 (31.3%)	1,178,210 (31.2%)	48.0	0.93
	35-54 Years	980 (47.0%)	1,353,341 (30.5%)	72.4	869 (48.2%)	1,180,392 (31.3%)	73.6	0.98
	55+ Years	509 (24.4%)	1,750,949 (39.4%)	29.1	369 (20.5%)	1,413,734 (37.5%)	26.1	1.11
	**Sex**							
	Male	1,431 (68.6%)	2,133,827 (48.1%)	67.1	1,218 (67.6%)	1,825,776 (48.4%)	66.7	1.01
	Female	655 (31.4%)	2,305,343 (51.9%)	28.4	585 (32.4%)	1,946,560 (51.6%)	30.1	0.95
	Missing	0 (0.0%)	NA	NA	0 (0.0%)	NA	NA	NA
	**Race/Ethnicity**							
	Non-Hispanic White	1,591 (76.3%)	3,229,233 (72.7%)	49.3	1,301 (72.2%)	2,750,369 (72.9%)	47.3	1.04
	Non-Hispanic Black	349 (16.7%)	728,037 (16.4%)	47.9	331 (18.4%)	545,357 (14.5%)	60.7	0.79

© 2024 Freisthler B et al. *JAMA Network Open.*

	Non-Hispanic Other	16 (0.8%)	200,571 (4.5%)	8.0	25 (1.4%)	153,956 (4.1%)	16.2	0.49
	Hispanic	127 (6.1%)	281,329 (6.3%)	45.1	144 (8.0%)	322,654 (8.6%)	44.6	1.01
	Missing	3 (0.1%)	NA	NA	2 (0.1%)	NA	NA	NA
**Overdose Deaths Involving any Opioid and any Psychostimulant (Other than Cocaine)**	**Overall**	240	4,439,170	5.4	235	3,772,336	6.2	0.87
	**Research Site**							
	Kentucky	63 (26.3%)	617,841 (13.9%)	10.2	105 (44.7%)	815,764 (21.6%)	12.9	0.79
	Massachusetts	8 (3.3%)	359,314 (8.1%)	2.2	2 (0.9%)	356,545 (9.5%)	0.6	3.97
	New York	12 (5.0%)	1,101,497 (24.8%)	1.1	24 (10.2%)	976,069 (25.9%)	2.5	0.44
	Ohio	157 (65.4%)	2,360,518 (53.2%)	6.7	104 (44.3%)	1,623,958 (43.0%)	6.4	1.04
	**Urban/Rural Classification**							
	Urban	178 (74.2%)	3,793,353 (85.5%)	4.7	212 (90.2%)	3,242,663 (86.0%)	6.5	0.72
	Rural	62 (25.8%)	645,817 (14.5%)	9.6	23 (9.8%)	529,673 (14.0%)	4.3	2.21
**Overdose Deaths Involving any Opioid and Cocaine**	**Overall**	519	4,439,170	11.7	461	3,772,336	12.2	0.96
	**Research Site**							
	Kentucky	28 (5.4%)	617,841 (13.9%)	4.5	33 (7.2%)	815,764 (21.6%)	4.0	1.12
	Massachusetts	59 (11.4%)	359,314 (8.1%)	16.4	83 (18.0%)	356,545 (9.5%)	23.3	0.71
	New York	99 (19.1%)	1,101,497 (24.8%)	9.0	113 (24.5%)	976,069 (25.9%)	11.6	0.78
	Ohio	333 (64.2%)	2,360,518 (53.2%)	14.1	232 (50.3%)	1,623,958 (43.0%)	14.3	0.99
	**Urban/Rural Classification**							
	Urban	495 (95.4%)	3,793,353 (85.5%)	13.0	425 (92.2%)	3,242,663 (86.0%)	13.1	1.00
	Rural	24 (4.6%)	645,817 (14.5%)	3.7	36 (7.8%)	529,673 (14.0%)	6.8	0.55
**Overdose Deaths Involving any Opioid and any Benzodiazepine**	**Overall**	272	4,439,170	6.1	228	3,772,336	6.0	1.01
	**Research Site**							
	Kentucky	37 (13.6%)	617,841 (13.9%)	6.0	72 (31.6%)	815,764 (21.6%)	8.8	0.68
	Massachusetts	49 (18.0%)	359,314 (8.1%)	13.6	44 (19.3%)	356,545 (9.5%)	12.3	1.11

© 2024 Freisthler B et al. *JAMA Network Open.*

	New York	73 (26.8%)	1,101,497 (24.8%)	6.6	57 (25.0%)	976,069 (25.9%)	5.8	1.13
	Ohio	113 (41.5%)	2,360,518 (53.2%)	4.8	55 (24.1%)	1,623,958 (43.0%)	3.4	1.41
	**Urban/Rural Classification**							
	Urban	242 (89.0%)	3,793,353 (85.5%)	6.4	191 (83.8%)	3,242,663 (86.0%)	5.9	1.08
	Rural	30 (11.0%)	645,817 (14.5%)	4.6	37 (16.2%)	529,673 (14.0%)	7.0	0.66

[1]Sum of all events/ population in that group and trial group.

[2]Raw rate calculated as 100,000 multiplied by the sum of all events in that group and trial groupwave divided by the sum of the population in that group and trial group.

[3]Overall rate ratio calculated as the overall raw rate in Intervention Communities divided by the overall raw rate in Control Communities.

© 2024 Freisthler B et al. *JAMA Network Open.*

eTable 2. Descriptive Means of Primary and Secondary Outcomes During the Evaluation Period (July 1, 2021 - June 30, 2022) Using the Intention-to-Treat Population

Outcome	Group	Intervention Communities			Control Communities			Overall Rate Ratio[3]
		Events[1]	Population[1]	Raw Rate[2]	Events[1]	Population[1]	Raw Rate[2]	
**Drug Overdose Deaths**	**Overall**	75.9 (115.6)	130,563.8 (200,088.0)	57.6 (30.3)	80.0 (161.3)	114,313.2 (201,417.3)	61.2 (24.5)	0.94
	**Research Site**							
	Kentucky	56.3 (49.2)	77,230.1 (80,938.9)	81.7 (25.9)	84.1 (185.3)	101,970.5 (202,045.3)	68.2 (24.2)	1.20
	Massachusetts	28.0 (24.8)	44,914.3 (26,559.3)	54.1 (29.3)	33.1 (28.0)	44,568.1 (33,628.8)	71.3 (24.5)	0.76
	New York	67.9 (84.1)	137,687.1 (140,779.9)	38.5 (17.4)	76.6 (83.6)	122,008.6 (106,012.1)	52.2 (25.3)	0.74
	Ohio	136.5 (186.0)	236,051.8 (322,922.0)	56.5 (32.8)	121.0 (251.2)	180,439.8 (325,173.2)	54.0 (22.7)	1.05
	**Urban/Rural Classification**							
	Urban	115.9 (142.5)	199,650.2 (248,385.2)	59.5 (22.5)	122.7 (203.8)	170,666.5 (252,614.9)	63.5 (23.3)	0.94
	Rural	25.3 (21.4)	43,054.5 (19,075.4)	55.3 (38.7)	22.1 (16.3)	37,833.8 (23,733.0)	58.1 (26.7)	0.95
	**Age**							
	18-34 Years	19.3 (27.5)	39,261.2 (60,440.2)	51.1 (26.0)	21.3 (45.0)	35,703.3 (69,802.6)	58.8 (28.7)	0.87
	35-54 Years	37.1 (56.9)	39,804.1 (60,321.8)	92.8 (58.4)	38.5 (78.2)	35,769.5 (65,163.6)	96.1 (40.8)	0.97
	55+ Years	19.6 (32.5)	51,498.5 (79,908.4)	37.2 (21.3)	20.1 (39.3)	42,840.4 (67,485.8)	37.5 (25.7)	0.99
	**Sex**							
	Male	52.1 (79.3)	62,759.6 (94,370.9)	80.5 (46.7)	55.1 (109.3)	55,326.5 (96,853.5)	87.6 (37.8)	0.92
	Female	23.8 (36.6)	67,804.2 (105,743.9)	36.5 (20.1)	24.9 (52.2)	58,986.7 (104,579.4)	36.2 (19.0)	1.01
	**Race/Ethnicity**							
	Non-Hispanic White	53.7 (70.5)	94,977.4 (128,763.1)	59.1 (33.3)	53.8 (102.1)	83,344.5 (135,601.7)	60.1 (26.5)	0.98
	Non-Hispanic Black	16.4 (38.7)	21,412.9 (55,089.6)	101.8 (146.2)	18.8 (52.8)	16,526.0 (45,296.8)	156.0 (228.4)	0.65
	Non-Hispanic Other	1.1 (2.5)	5,899.1 (9,757.0)	14.9 (50.9)	1.3 (3.2)	4,665.3 (11,990.9)	30.6 (110.7)	0.49
	Hispanic	4.5 (9.0)	8,274.4 (13,482.1)	27.3 (39.7)	5.8 (11.2)	9,777.4 (17,743.5)	54.5 (82.9)	0.50
	Missing	0.3 (0.8)	NA	NA	0.4 (1.0)	NA	NA	NA

© 2024 Freisthler B et al. *JAMA Network Open.*

**Overdose Deaths Involving any Opioid and any Psychostimulant (Other than Cocaine)**	**Overall**	11.6 (17.4)	130,563.8 (200,088.0)	11.1 (14.3)	16.2 (40.4)	114,313.2 (201,417.3)	13.0 (12.8)	0.86
	**Research Site**							
	Kentucky	16.3 (13.6)	77,230.1 (80,938.9)	24.0 (13.6)	33.1 (73.6)	101,970.5 (202,045.3)	26.8 (11.3)	0.90
	Massachusetts	1.1 (2.1)	44,914.3 (26,559.3)	1.4 (2.6)	1.1 (1.2)	44,568.1 (33,628.8)	2.4 (2.6)	0.56
	New York	5.1 (7.0)	137,687.1 (140,779.9)	3.1 (1.8)	10.1 (12.3)	122,008.6 (106,012.1)	9.5 (8.3)	0.33
	Ohio	21.6 (25.6)	236,051.8 (322,922.0)	14.9 (17.1)	19.8 (33.1)	180,439.8 (325,173.2)	13.1 (12.7)	1.13
	**Urban/Rural Classification**							
	Urban	15.1 (21.2)	199,650.2 (248,385.2)	7.9 (10.8)	23.8 (52.2)	170,666.5 (252,614.9)	11.8 (11.9)	0.67
	Rural	7.3 (10.0)	43,054.5 (19,075.4)	15.1 (17.3)	5.8 (6.9)	37,833.8 (23,733.0)	14.5 (14.2)	1.04
**Overdose Deaths Involving any Opioid and Cocaine**	**Overall**	21.9 (41.0)	130,563.8 (200,088.0)	11.5 (10.8)	23.8 (56.5)	114,313.2 (201,417.3)	14.9 (16.1)	0.77
	**Research Site**							
	Kentucky	7.1 (11.5)	77,230.1 (80,938.9)	6.2 (5.0)	12.0 (30.7)	101,970.5 (202,045.3)	5.3 (4.8)	1.17
	Massachusetts	11.6 (14.3)	44,914.3 (26,559.3)	19.4 (15.9)	13.5 (12.9)	44,568.1 (33,628.8)	28.0 (21.0)	0.69
	New York	26.5 (39.4)	137,687.1 (140,779.9)	11.8 (9.9)	29.5 (50.3)	122,008.6 (106,012.1)	17.8 (16.6)	0.66
	Ohio	38.4 (64.0)	236,051.8 (322,922.0)	9.4 (7.0)	38.3 (95.2)	180,439.8 (325,173.2)	9.2 (9.0)	1.02
	**Urban/Rural Classification**							
	Urban	37.3 (50.2)	199,650.2 (248,385.2)	16.4 (11.8)	38.1 (71.7)	170,666.5 (252,614.9)	19.4 (18.5)	0.85
	Rural	2.5 (2.3)	43,054.5 (19,075.4)	5.4 (4.6)	4.4 (5.2)	37,833.8 (23,733.0)	8.8 (9.8)	0.61
**Overdose Deaths Involving any Opioid and any Benzodiazepine**	**Overall**	8.6 (13.9)	130,563.8 (200,088.0)	6.9 (5.6)	8.2 (15.6)	114,313.2 (201,417.3)	6.9 (6.0)	1.01
	**Research Site**							
	Kentucky	5.6 (4.1)	77,230.1 (80,938.9)	9.6 (7.3)	11.3 (24.2)	101,970.5 (202,045.3)	10.1 (8.4)	0.95
	Massachusetts	5.5 (5.7)	44,914.3 (26,559.3)	9.9 (5.6)	3.0 (2.6)	44,568.1 (33,628.8)	7.8 (6.0)	1.28
	New York	10.8 (14.4)	137,687.1 (140,779.9)	5.4 (3.7)	8.9 (11.9)	122,008.6 (106,012.1)	6.1 (4.3)	0.89

© 2024 Freisthler B et al. *JAMA Network Open.*

	Ohio	11.6 (21.8)	236,051.8 (322,922.0)	3.7 (3.3)	9.7 (16.9)	180,439.8 (325,173.2)	4.0 (3.7)	0.92
	**Urban/Rural Classification**							
	Urban	13.7 (17.0)	199,650.2 (248,385.2)	8.0 (5.8)	12.2 (19.7)	170,666.5 (252,614.9)	6.6 (5.1)	1.21
	Rural	2.1 (1.8)	43,054.5 (19,075.4)	5.6 (5.3)	2.9 (2.8)	37,833.8 (23,733.0)	7.3 (7.3)	0.77

[1]**Mean (Standard Deviation) of the community-level events or population in that group and trial group.**

[2]**Mean (Standard Deviation) of the community-level rates expressed per 100,000 residents 18 years of age or older in that group and trial group.**

[3]**Overall rate ratio calculated as the raw rate in Intervention Communities divided by the raw rate in Control Communities.**

© 2024 Freisthler B et al. *JAMA Network Open.*

eTable 3. Descriptive Sums of Tertiary Outcomes During the Evaluation Period (July 1, 2021 - June 30, 2022) Using the Intention-to-Treat Population

Outcome	Group	Intervention Communities			Control Communities			Overall Rate Ratio[3]
		Total Events[1]	Total Population[1]	Overall Raw Rate[2]	Total Events[1]	Total Population[1]	Overall Raw Rate[2]	
**Opioid Overdose Deaths Involving Heroin**	**Overall**	93	4,439,170	2.1	125	3,772,336	3.3	0.63
	**Research Site**							
	Kentucky	10 (10.8%)	617,841 (13.9%)	1.6	19 (15.2%)	815,764 (21.6%)	2.3	0.69
	Massachusetts	13 (14.0%)	359,314 (8.1%)	3.6	43 (34.4%)	356,545 (9.5%)	12.1	0.30
	New York	28 (30.1%)	1,101,497 (24.8%)	2.5	54 (43.2%)	976,069 (25.9%)	5.5	0.46
	Ohio	42 (45.2%)	2,360,518 (53.2%)	1.8	9 (7.2%)	1,623,958 (43.0%)	0.6	3.21
	**Urban/Rural Classification**							
	Urban	75 (80.6%)	3,793,353 (85.5%)	2.0	94 (75.2%)	3,242,663 (86.0%)	2.9	0.68
	Rural	18 (19.4%)	645,817 (14.5%)	2.8	31 (24.8%)	529,673 (14.0%)	5.9	0.48
**Overdose Deaths Involving Synthetic Opioids Except Methadone**	**Overall**	2,075	4,439,170	46.7	2,165	3,772,336	57.4	0.81
	**Research Site**							
	Kentucky	350 (16.9%)	617,841 (13.9%)	56.6	571 (26.4%)	815,764 (21.6%)	70.0	0.81
	Massachusetts	193 (9.3%)	359,314 (8.1%)	53.7	232 (10.7%)	356,545 (9.5%)	65.1	0.83
	New York	433 (20.9%)	1,101,497 (24.8%)	39.3	507 (23.4%)	976,069 (25.9%)	51.9	0.76
	Ohio	1,099 (53.0%)	2,360,518 (53.2%)	46.6	855 (39.5%)	1,623,958 (43.0%)	52.6	0.88
	**Urban/Rural Classification**							
	Urban	1,799 (86.7%)	3,793,353 (85.5%)	47.4	1,912 (88.3%)	3,242,663 (86.0%)	59.0	0.80
	Rural	276 (13.3%)	645,817 (14.5%)	42.7	253 (11.7%)	529,673 (14.0%)	47.8	0.89
**Overdose Deaths Involving Any**	**Overall**	1,059	4,439,170	23.9	1,218	3,772,336	32.3	0.74
	**Research Site**							

© 2024 Freisthler B et al. *JAMA Network Open.*

**Opioid and Any Psychostimulant (Including Cocaine)**	Kentucky	176 (16.6%)	617,841 (13.9%)	28.5	324 (26.6%)	815,764 (21.6%)	39.7	0.72
	Massachusetts	97 (9.2%)	359,314 (8.1%)	27.0	114 (9.4%)	356,545 (9.5%)	32.0	0.84
	New York	237 (22.4%)	1,101,497 (24.8%)	21.5	301 (24.7%)	976,069 (25.9%)	30.8	0.70
	Ohio	549 (51.8%)	2,360,518 (53.2%)	23.3	479 (39.3%)	1,623,958 (43.0%)	29.5	0.79
	**Urban/Rural Classification**							
	Urban	917 (86.6%)	3,793,353 (85.5%)	24.2	1,085 (89.1%)	3,242,663 (86.0%)	33.5	0.72
	Rural	142 (13.4%)	645,817 (14.5%)	22.0	133 (10.9%)	529,673 (14.0%)	25.1	0.88
**Overdose Deaths Involving Any Psychostimulant (Other than Cocaine)**	**Overall**	482	4,439,170	10.9	638	3,772,336	16.9	0.64
	**Research Site**							
	Kentucky	155 (32.2%)	617,841 (13.9%)	25.1	303 (47.5%)	815,764 (21.6%)	37.1	0.68
	Massachusetts	14 (2.9%)	359,314 (8.1%)	3.9	12 (1.9%)	356,545 (9.5%)	3.4	1.16
	New York	52 (10.8%)	1,101,497 (24.8%)	4.7	97 (15.2%)	976,069 (25.9%)	9.9	0.48
	Ohio	261 (54.1%)	2,360,518 (53.2%)	11.1	226 (35.4%)	1,623,958 (43.0%)	13.9	0.79
	**Urban/Rural Classification**							
	Urban	347 (72.0%)	3,793,353 (85.5%)	9.1	545 (85.4%)	3,242,663 (86.0%)	16.8	0.54
	Rural	135 (28.0%)	645,817 (14.5%)	20.9	93 (14.6%)	529,673 (14.0%)	17.6	1.19
**Overdose Deaths Involving Cocaine**	**Overall**	891	4,439,170	20.1	924	3,772,336	24.5	0.82
	**Research Site**							
	Kentucky	65 (7.3%)	617,841 (13.9%)	10.5	105 (11.4%)	815,764 (21.6%)	12.9	0.82
	Massachusetts	105 (11.8%)	359,314 (8.1%)	29.2	122 (13.2%)	356,545 (9.5%)	34.2	0.85
	New York	250 (28.1%)	1,101,497 (24.8%)	22.7	267 (28.9%)	976,069 (25.9%)	27.4	0.83
	Ohio	471 (52.9%)	2,360,518 (53.2%)	20.0	430 (46.5%)	1,623,958 (43.0%)	26.5	0.75

© 2024 Freisthler B et al. *JAMA Network Open.*

	**Urban/Rural Classification**							
	Urban	845 (94.8%)	3,793,353 (85.5%)	22.3	853 (92.3%)	3,242,663 (86.0%)	26.3	0.85
	Rural	46 (5.2%)	645,817 (14.5%)	7.1	71 (7.7%)	529,673 (14.0%)	13.4	0.53
**Overdose Deaths Involving Any Psychostimulant (Including Cocaine)**	**Overall**	1,277	4,439,170	28.8	1,449	3,772,336	38.4	0.75
	**Research Site**							
	Kentucky	207 (16.2%)	617,841 (13.9%)	33.5	370 (25.5%)	815,764 (21.6%)	45.4	0.74
	Massachusetts	113 (8.8%)	359,314 (8.1%)	31.4	131 (9.0%)	356,545 (9.5%)	36.7	0.86
	New York	284 (22.2%)	1,101,497 (24.8%)	25.8	346 (23.9%)	976,069 (25.9%)	35.4	0.73
	Ohio	673 (52.7%)	2,360,518 (53.2%)	28.5	602 (41.5%)	1,623,958 (43.0%)	37.1	0.77
	**Urban/Rural Classification**							
	Urban	1,102 (86.3%)	3,793,353 (85.5%)	29.1	1,294 (89.3%)	3,242,663 (86.0%)	39.9	0.73
	Rural	175 (13.7%)	645,817 (14.5%)	27.1	155 (10.7%)	529,673 (14.0%)	29.3	0.93
**Overdose Deaths Involving Benzodiazepine**	**Overall**	317	4,439,170	7.1	304	3,772,336	8.1	0.89
	**Research Site**							
	Kentucky	52 (16.4%)	617,841 (13.9%)	8.4	100 (32.9%)	815,764 (21.6%)	12.3	0.69
	Massachusetts	46 (14.5%)	359,314 (8.1%)	12.8	28 (9.2%)	356,545 (9.5%)	7.9	1.63
	New York	92 (29.0%)	1,101,497 (24.8%)	8.4	76 (25.0%)	976,069 (25.9%)	7.8	1.07
	Ohio	127 (40.1%)	2,360,518 (53.2%)	5.4	100 (32.9%)	1,623,958 (43.0%)	6.2	0.87
	**Urban/Rural Classification**							
	Urban	282 (89.0%)	3,793,353 (85.5%)	7.4	257 (84.5%)	3,242,663 (86.0%)	7.9	0.94
	Rural	35 (11.0%)	645,817 (14.5%)	5.4	47 (15.5%)	529,673 (14.0%)	8.9	0.61

[1]Sum of all events/ population in that group and trial group.

© 2024 Freisthler B et al. *JAMA Network Open.*

[2]Raw rate calculated as 100,000 multiplied by the sum of all events in that group and trial groupwave divided by the sum of the population in that group and trial group.
[3]Overall rate ratio calculated as the overall raw rate in Intervention Communities divided by the overall raw rate in Control Communities.

© 2024 Freisthler B et al. *JAMA Network Open.*

eTable 4. Calculation of Proportions of Opioid Deaths Involving Specific Drugs

First Outcome	Second Outcome	Intervention Communities			Control Communities		
		Total Events in First Outcome[1]	Total Events in Second Outcome[1]	Proportion[2]	Total Events in First Outcome[1]	Total Events in Second Outcome[1]	Proportion[2]
Number of Opioid Overdose Deaths Involving Synthetic Opioids Except Methadone	Number of Opioid Overdose Deaths[3]	2,075	2,220	0.93	2,165	2,297	0.94
Number of Opioid Overdose Deaths Involving Heroin	Number of Opioid Overdose Deaths[3]	93	2,220	0.04	125	2,297	0.05
Number of Opioid Overdose Deaths Involving Any Opioid and Any Psychostimulant (Including Cocaine)	Number of Opioid Overdose Deaths[3]	1,059	2,220	0.48	1,218	2,297	0.53
Number of Opioid Overdose Deaths Involving Any Opioid and Any Psychostimulant (Including Cocaine)	Number of Drug Overdose Deaths Involving Any Psychostimulant (Including Cocaine)	1,059	1,277	0.83	1,218	1,449	0.84

[1]Sum of events in that trial group.

[2]Proportion calculated as the total events in first outcome divided by the total events in second outcome.

[3] Samet et al. [21]

© 2024 Freisthler B et al. *JAMA Network Open.*

eTable 5. Adjusted Rate of Each Outcome Within Trial Groups and Adjusted Relative Rate of Each Outcome Between Intervention to Control Communities During the Evaluation Period Using the Per-Protocol Population[1]

Outcome	Intervention Communities	Control Communities	Adjusted Rate Ratio (95% CI)[3]	P-value
	Adjusted Rate (95% CI)[2]	Adjusted Rate (95% CI)[2]		
**Drug Overdose Deaths[4]**	56.21 (50.20, 62.94)	61.00 (54.14, 68.72)	0.92 (0.79, 1.08)	0.294
**Overdose Deaths Involving any Opioid and any Psychostimulant (Other than Cocaine)[5]**	6.60 (5.06, 8.60)	10.54 (7.75, 14.33)	0.63 (0.43, 0.90)	0.013
**Overdose Deaths Involving any Opioid and Cocaine[6]**	10.40 (8.38, 12.92)	10.97 (8.31, 14.50)	0.95 (0.69, 1.30)	0.739
**Overdose Deaths Involving any Opioid and any Benzodiazepine[7]**	6.62 (5.27, 8.31)	6.58 (4.94, 8.77)	1.01 (0.74, 1.36)	0.971
**[1]Results obtained from a negative binomial model adjusting for research site (KY, MA, NY, OH), urban/rural classification, baseline opioid overdose death rate, and baseline rate of the outcome. The natural log of the observed community population size of individuals 18 years of age or older measured from the 2020 Bridged-Race Population Estimates or the 2017-2021 American Community Survey 5-Year Averages is used as an offset.**				
**[2]Model estimated event rate expressed as per 100,000 residents aged 18 and older.**				
**[3]Adjusted relative rate of outcome during the evaluation period between Intervention and Control communities.**				
**[4]Estimated dispersion parameter, k: 0.0439; 95%CI: (0.0217, 0.0888)**				
**[5]Estimated dispersion parameter, k: 0.2136; 95%CI: (0.1094, 0.4170)**				
**[6]Estimated dispersion parameter, k: 0.1454; 95%CI: (0.0762, 0.2774)**				
**[7]Estimated dispersion parameter, k: 0.0847; 95%CI: (0.0261, 0.2747)**				

© 2024 Freisthler B et al. *JAMA Network Open.*

eTable 6. Sensitivity Analyses

Sensitivity Post-Hoc	Outcome	Distribution	Weight/ offset	Covariates	Subgroup	Adjusted rate in Intervention Communities	Adjusted rate in Control Communities	Adjusted rate ratio between Intervention and Control Communities	P-Value
**Primary Outcome**									
**Assess Impact of Community Size**	Drug overdose deaths during the comparison period	Poisson	Offset: Log of population size	Trial group, research site, urban/rural, baseline rate of M1, baseline rate of outcome	Overall	55.14 (49.39, 61.56)	64.93 (57.87, 72.87)	0.85 (0.72, 1.00)	0.045
**Assess Impact of Assuming Common Effect of Urban/Rural Status Across Research Sites**	Drug overdose deaths during the comparison period	Negative binomial	Offset: Log of population size	Trial group, research site, urban/rural, baseline rate of M1, baseline rate of outcome, research site*urban/rural	Overall	55.15 (49.13, 61.90)	62.21 (54.90, 70.48)	0.89 (0.76, 1.04)	0.127
**Secondary Outcomes**									
**Assess Impact of Community Size**	Overdose deaths involving any opioid and any psychostimulant (other than cocaine) during the comparison period	Poisson	Offset: Log of population size	Trial group, research site, urban/rural, baseline rate of M1, baseline rate of outcome	Overall	7.19 (5.76, 8.98)	10.57 (8.14, 13.73)	0.68 (0.55, 0.85)	<0.001
**Assess Impact of Community Size**	Overdose deaths involving any opioid and cocaine during the comparison period	Poisson	Offset: Log of population size	Trial group, research site, urban/rural, baseline rate of M1, baseline rate of outcome	Overall	11.01 (8.51, 14.23)	12.42 (9.46, 16.31)	0.89 (0.63, 1.25)	0.482

© 2024 Freisthler B et al. *JAMA Network Open.*

**Assess Impact of Community Size**	Overdose deaths involving any opioid and any benzodiazepine during the comparison period	Poisson	Offset: Log of population size	Trial group, research site, urban/rural, baseline rate of M1, baseline rate of outcome	Overall	6.56 (5.17, 8.33)	7.00 (5.37, 9.14)	0.94 (0.72, 1.22)	0.618
**Post-Hoc**						**Adjusted rate ratio between comparison and baseline periods in Intervention Communities (95% CI)**	**Adjusted rate ratio between comparison and baseline periods in Control Communities (95% CI)**	**Ratio between Intervention and Control Communities (95% CI)**	**P-value from interaction**
**Comparing the Change in Drug Overdose Death Rate Between the Baseline and Comparison Periods Between Trial Groups**	Drug overdose deaths	Negative binomial	Offset: Log of population size	Trial group, research site, urban/rural, time, trial group*time	Overall	1.30 (1.15, 1.47)	1.40 (1.26, 1.57)	0.93 (0.79, 1.09)	0.372
**Comparing the Change in Drug Overdose Death Rate Between the Baseline and Comparison Periods Between Trial Groups**	Drug overdose deaths	Poisson	Offset: Log of population size	Trial group, research site, urban/rural, time, trial group*time	Overall	1.24 (1.08, 1.42)	1.46 (1.33, 1.61)	0.85 (0.72, 1.00)	0.046

© 2024 Freisthler B et al. *JAMA Network Open.*
