# Supplementary material for: Communities That HEAL Intervention and Mortality Including Polysubstance Overdose Deaths: A Randomized Clinical Trial
Source: JAMA Netw Open. 2024 Oct 21;7(10):e2440006. doi: 10.1001/jamanetworkopen.2024.40006 (PMC11581668; doi:10.1001/jamanetworkopen.2024.40006)
Supplement: Supplement 3. — Data Sharing Statement [file jamanetwopen-e2440006-s003.pdf]

Data Sharing Statement

Freisthler. Communities That HEAL Intervention and Mortality Including Polysubstance Overdose Deaths. *JAMA Netw Open*. Published October 21, 2024.
doi:10.1001/jamanetworkopen.2024.40006

Data
Additional Information: ClinicalTrials.gov Identifier: NCT04111939
Data available: Yes
Data types: Deidentified participant data, Data dictionary
How to access data: University of Michigan's Inter-university Consortium for Political and Social Research (ICPSR) will archive HCS data. ICPSR home page: https://www.icpsr.umich.edu/web/pages/
When available: beginning date: 03-31-2025

Supporting Documents
Document types: None

Additional Information
Who can access the data: Researchers whose proposed use of the data has been approved
Types of analyses: For any purpose
Mechanisms of data availability: With a signed data access agreement
